# Supplementary material for: Gene Structure Evolution of the Short-Chain Dehydrogenase/Reductase (SDR) Family
Source: Genes (Basel). 2022 Dec 30;14(1):110. doi: 10.3390/genes14010110 (PMC9859523; doi:10.3390/genes14010110)
Supplement: Supplementary file 1 [file genes-14-00110-s001.zip › Online Resources 1/Online Resources 1.pdf]

STRUCTURE AND CATALYSIS CONSENSUSES OF THE HUMAN SDR FAMILY VARIANTS.

The variants of the human SDR family have two highly conserved consensuses: the structure and the catalysis consensus (Kallberg et al. 2002 [1]). The structure consensus is responsible for the formation of the important fold (the Rossman fold) of the SDR polypeptides leading to the tertiary structure of SDR enzymes. It participates to the formation of the enzyme active site as well as the coenzyme and the substrate binding sites. The catalytic consensus includes the amino acids directly involved in the intimate reaction catalytic mechanism of the SDR enzymes (Kallberg et al. 2002).
Two types of structure consensus are present in human SDR enzymes: TGxxxGxG and TGxxGxxG (Kallberg et al. 2002). We analyzed all the human SDR variants and found that structure consensus TGxxxGxG (consensus 1) is present in 54 human SDR variants (Online Resources 1, Table S1) and structure TGxxGxxG (consensus 2) in 21 human SDR variants (Online Resources 1, Table S2).
Variants of the SDR7C, SDR9C, SDR12C, SDE16C, SDR21C, SDR26C, SDR28C and SDR32C families have structure consensus 1, and variants of SDR10E, SDR11E and SDR42E2 families have structure consensus 2.
The conserved first glycine (G) of consensus 1 is substituted with alanine (A) in four variants (SDR15C1, SDR25C2 and SDR27X1) and with aspartic acid (D) in SDR32C2 variant (Gabrielli & Tofanelli, 2012 [35]; Online Resources 1, Table S1). The conserved last glycine of consensus 2 is substituted with alanine in SDR2E1 variant (Online Resources 1, Table S2).
The catalysis consensus (YxxxK), which is highly conserved in human, in SDR12C3 variant has the canonical tyrosine (T) substituted with phenylalanine (F). The tyrosine substitution makes the human SDR12C3 enzyme almost inactive as oxido-reductase enzyme, however the substitution is conserved among some vertebrate species (Meier et al. 2009 [36]; Online Resources 2, Fig. S17, Table S19), suggesting that, in different species, the SDR12C3 enzyme may have different molecular functions (Meier et al. 2009). Very long human variants SDR27X1 do not have the typical SDR catalytic consensus: we found only a single sequence, KAAAY, in which the sequence position of Y and K are inverted with respect to the conserved catalysis consensus. We also could not find the catalysis consensus in SDR17C1, SDR18C1, SDR39U1, SDR43U1 and SDR48A1 variants.

1. Kallberg, Y., Oppermann, U., Jörnvall, H., Persson, B., 2002. Short-chain dehydrogenases/reductases (SDRs). Coenzyme-based functional assignments in completed genomes. Eur. J. Biochem. 269, 4409-4417.

35. Gabrielli F., Tofanelli S., 2012. Molecular and functional evolution of human DHRS2 and DHRS4 duplicated genes. Gene 511, 461-9.

36. Meier, M., Tokarz, J., Haller, F., Mindnich, R., Adamski J., 2009. Human and zebrafish hydroxysteroid dehydrogenase like 1 (HSDL1) proteins are inactive enzymes but conserved among species. Chem. Biol. Interact. 178, 197–205.

Enzymes	Structure consensus 1	Catalysis consensus
SDR5C1	LVAVITGGASGLG	YSASK
SDR7C1	VVVVTGANTGIG	YCHSL
SDR7C2	VVVITGANTGIG	YCHSL
SDR7C3	VIVTGANTGIG	YCQSK
SDR7C4	VLITGANSGLG	YSRSK
SDR7C5	AVVTGANSGIG	YADTK
SDR8C1	LGGDFKGVG	YSAAK
SDR9C1	AVLVTGCDSGFG	YCITK
SDR9C2	AVLVTGGDCGLG	YGSSK
SDR9C3	AVLITGCDSGFG	YGTSK
SDR9C4	IFITGCDSGFG	YTPSK
SDR9C5	AFVFITGCDSGFG	YCVSK
SDR9C6	VFITGCDSGFG	YCVSK
SDR9C7	VFITGCDSGFG	YCVSK
SDR9C8	VFITGCDSGFG	YCISK
SDR12C1	AVVTGSTDGIG	YSATK
SDR12C2	AVITGAGDGIG	YSASK
SDR12C3	AVVSGATDGIG	FSASK
SDR13C1	VFTGASRGIG	YTIAK
SDR15C1	VIILTAAAQGIG	YSTTK
SDR16C1	VLITGGGRGIG	YCTSK
SDR16C2	IVLITGAGHGIG	YCSSK
SDR16C3	IVLITGAGHGIG	YCSSK
SDR16C4	LITGAGSGLG	YCASK
SDR16C5	IVLITGAGSGLG	YCASK
SDR17C1	VAFITGGGSGIG	N.F.
SDR18C1	VAFITGGGTGLG	N.F.
SDR19C1	VVTGASRGIG	YGVGK
SDR20C1	VLVTGAGKGIG	YCSTK
SDR21C1	VALVTGGNKGIG	YGVTK
SDR21C2	VALVTGANRGIG	YGVSK
SDR24C1	LALVTGASGGIG	YSATK
SDR25C1	VAVVTGSTSGIG	YNVSK
SDR25C2	VALVTASTDGIG	YNVSK
SDR25C3	VALVTASTDGIG	YNVSK
SDR26C1	VIVTGASKGIG	YSASK
SDR26C2	VLLTGANAGVG	YSAAK
SDR27X1	LLARALGLG	KAAAY
SDR28C1	VVLITGCSSGIG	YCASK
SDR28C2	VLISGCSSGIG	YAASK
SDR29C1	VAIVTGGATGIG	YNLTK
SDR30C1	ALALVTGAGSGIG	YAASK
SDR32C1	AVVITGATSGLG	YAASK
SDR32C2	VVVITDAISGLG	YAASK
SDR34C1	VTGASSGIG	YCASK
SDR35C1	VVVTGGSSGIG	YSASK
SDR36C1	VALVTGAAQGIG	YCASK
SDR37C1	VVLITGASSGIG	YSSSK
SDR38C1	AVCLLTGASRGFG	YCAGK
SDR40C1	VFLVTGGNSGIG	YAQNK
SDR41C1	VVVVTGANSGIG	YNRSK
SDR45C1	VCAVFGGSRGIG	YSASK
SDR46C1	VAIVTGGTDGIG	YAQSK
SDR47C1	VVVVTGGGRGIG	YVATK

Table S1. Structure and catalysis consensuses of the human SDR enzymes belonging to the Classical SDR families. Structure consensus 1 has the typical structure GxxxGxG. Atypical amino acid and hydrophobic amino acid are highlighted in turquoise and yellow respectively. Conserved amino acids symbols of the structure and of the catalysis consensuses are written in red. N.F., not found.

Enzymes	Structure consensus 2	Catalysis consensus
SDR1E1	VLVTGGAGYIG	YGKSK
SDR2E1	VLVTGGAGFIA	YASSK
SDR3E1	VALITGITGQDG	YGAAK
SDR4E1	ILVTGGSGLVG	YSYAK
SDR6E1	ILITGGAGFVG	YDEGK
SDR10E1	VLLTGATGFLG	YIYTK
SDR10E2	SILITGATGFLG	YTYTK
SDR11E1	LVTGAGGFLG	YPHSK
SDR11E2	LVTGAGGLLG	YPYSK
SDR11E3	LVTGGCGFLG	YPCSK
SDR14E1	VLITGALGQLG	YGVSK
SDR22E1	VFGATGFLG	YLRNK
SDR23E1	VLVTGATGLLG	YGKTK
SDR31E1	VIGGSGFLG	YTETK
SDR33C1	VLVYGGRGALG	YGMAK
SDR39U1	VLVGGGTGFIG	N.F.
SDR42E1	VLITGGSGYFG	YSRTK
SDR42E2	VLVTGGGGYLG	YSRTK
SDR43U1	IAIFGATGQTG	N.F.
SDR44U1	VFILGASGETG	YLQVK
SDR48A1	LVVVFGGTGAQG	N.F.

Table S2. Structure and catalysis consensuses of the human SDR enzymes belonging to the Extended, Atypical and Unknown SDR families. Structure consensus 2 has the typical structure GxxGxxG. Atypical and hydrophobic amino acid are highlighted in turquoise and yellow respectively. N.F., not found. For further details see Fig. S50.
