# Supplementary material for: Gene Structure Evolution of the Short-Chain Dehydrogenase/Reductase (SDR) Family
Source: Genes (Basel). 2022 Dec 30;14(1):110. doi: 10.3390/genes14010110 (PMC9859523; doi:10.3390/genes14010110)
Supplement: Supplementary file 1 [file genes-14-00110-s001.zip › Online Resources 2/Online Resources 2.pdf]

HUMAN SDR FAMILY VARIANTS

```
SDR7C1        ----------------MVELMFPLLLLLLPFLLYMAAPQIRKMLSSGVCTSTVQLPGKVV  1p.1
SDR7C2        ------------------MLVTLGLLTSFFSFLYMVAPSIRKFFAGGVCRTNVQLPGKVV  1p.1
SDR7C3        -------------------MSRYLLPLSALGTVAGAAVLLKDYVTGGACPSKATIPGKTV  1p.1
SDR7C4        --------------MAVATAAAVLAALGGALWLAARRFVGPRVQRLRRGGDPGLMHGKTV
SDR7C5        ---------------------MEALLLGAGLLLGAYVLVYYNLVKAPPCGGMGNLRGRTA
SDR40C1       -----------------MSLYRSVVWFAKGLREYTKSGYESACKDFVPHDLEVQIPGRVF  1p.1
                                                                      *

                 ×   × ×
SDR7C1        VVTGANTGIGKETAKELAQRGARVYLACRDVEKGELVAKEIQTT-------------TGN  2p.2
SDR7C2        VITGANTGIGKETARELASRGARVYIACRDVLKGESAASEIRVD-------------TKN  2p.2
SDR7C3        IVTGANTGIGKQTALELARRGGNIILACRDMEKCEAAAKDIRGE-------------TLN  2p.2
SDR7C4        LITGANSGLGRATAAELLRLGARVIMGCRDRARAEEAAGQLRRELRQAAECGPEPGVSGV
SDR7C5        VVTGANSGIGKMTALELARRGARVVLACRSQERGEAAAFDLRQE-------------SGN  1p.2 2p.0
SDR40C1       LVTGGNSGIGKATALEIAKRGGTVHLVCRDQAPAEDARGEIIRE-------------SGN  2p.2
                ** * * *  ** *    *      **     *

SDR7C1        QQVLVRKLDLSDTKSIRAFAKGFLAEEKHLHVLINNAGVMMCPYSKTADGFEMHIGVNHL  3p.2      4p.2
SDR7C2        SQVLVRKLDLSDTKSIRAFAEGFLAEEKQLHILINNAGVMMCPYSKTADGFETHLGVNHL  3p.2      4p.2
SDR7C3        HHVNARHLDLASLKSIREFAAKIIEEEERVDILINNAGVMRCPHWTTEDGFEMQFGVNHL  3p.2      4p.2
SDR7C4        GELIVRELDLASLRSVRAFCQEMLQEEPRLDVLINNAGIFQCPYMKTEDGFEMQFGVNHL  1p.0
SDR7C5        NEVIFMALDLASLASVRAFATAFLSSEPRLDILIHNAGISSCG--RTREAFNLLLRVNHI  3p.2
SDR40C1       QNIFLHIVDLSDPKQIWKFVENFKQ-EHKLHVLINNAGCMVNKRELTEDGLEKNFAANTL  3p.0 4p.0 5p.2
                      **        *       *     ** ***        *          *

                                                                  +   +
SDR7C1        GHFLLTHLLLEKLKESAPSRIVNVSSLAHHLGRIHFHNLQG-EKFYNA-GLAYCHSKLAN
SDR7C2        GHFLLTYLLLERLKVSAPARVVNVSSVAHHIGKIPFHDLQS-EKRYSR-GFAYCHSKLAN
SDR7C3        GHFLLTNLLLDKLKASAPSRIINLSSLAHVAGHIDFDDLNWQTRKYNT-KAAYCQSKLAI
SDR7C4        GHFLLTNLLLGLLKSSAPSRIVVVSSKLYKYGDINFDDLNS-EQSYNK-SFCYSRSKLAN
SDR7C5        GPFLLTHLLLPCLKACAPSRVVVVASAAHCRGRLDFKRLDRPVVGWRQELRAYADTKLAN
SDR40C1       GVYILTTGLIPVLEKEHDPRVITVSSGGMLVQKLNTNDLQSERTPFDG-TMVYAQNKRQQ  6p.0 7p.0
              *   **  *   *      *     *            *             *   *

SDR7C1        ILFTQELARRLKGSGVTTYSVHPGTVQSELVRHSSFMRWM------WWLFSFFIKTPQQG  5p.2
SDR7C2        VLFTRELAKRLQGTGVTTYAVHPGVVRSELVRHSSLLCLL------WRLFSPFVKTAREG  5p.2
SDR7C3        VLFTKELSRRLQGSGVTVNALHPGVARTELGRHTGIHGSTFSSTTLGPIFWLLVKSPELA  5p.2 6p.2
SDR7C4        ILFTRELARRLEGTNVTVNVLHPGIVRTNLGRHIHIPLLV--KPLFNLVSWAFFKTPVEG
SDR7C5        VLFARELANQLEATGVTCYAAHPGPVNSELFL-RHVPGWL--RPLLRPLAWLVLRAPRGG  4p.2
SDR40C1       VVLTERWAQGHP--AIHFSSMHPGWADTPGVRQ--------AMPGFHARFGDRLRSEAQG  8p.2
                                   ***

SDR7C1        AQTSLHCALTEG-LEILSGNHFSDCHVAWVS-AQARN---ETIARRLWDVSCDLLGLPID  6p.1
SDR7C2        AQTSLHCALAEG-LEPLSGKYFSDCKRTWVS-PRARN---NKTAERLWNVSCELLGIRWE  6p.1
SDR7C3        AQPSTYLAVAEE-LADVSGKYFDGLKQKAPA-PEAED---EEVARRLWAESARLVGLEAP
SDR7C4        AQTSIYLASSPE-VEGVSGRYFGDCKEEELL-PKAMD---ESVARKLWDISEVMVGLLK-
SDR7C5        AQTPLYCALQEG-IEPLSGRYFANCHVEEVP-PAARD---DRAAHRLWEASKRLAGLGPG
SDR40C1       ADTMLWLALSSAAAAQPSGRFFQDRKPVSTHLPLATASSSPAEEEKLIEILEQLAQTFK-  9p.2
              *      *         **  *            *           *
```

```
SDR7C1        -------------------------------------------------------------
SDR7C2        -------------------------------------------------------------
SDR7C3        SVREQPLPR----------------------------------------------------
SDR7C4        -------------------------------------------------------------
SDR7C5        EDAEPDEDPQSEDSEAPSSLSTPHPEEPTVSQPYPSPQSSPDLSKMTHRIQAKVEPEIQLS
SDR40C1       -------------------------------------------------------------
```

Figure S1. Alignment of the human SDR7C family and of SDR40C1 protein variants. × and + symbols mark the structure consensus and the catalysis consensus respectively; the couples of amino acid symbols in red lowercase mark the splicing-site positions. Splicing sites are progressively numbered and phase (p.) type is indicated after the splicing-site number. * symbol marks the position of identical amino acid residues of the aligned SDR protein sequences.

```
SDR9C1        --------------------MLA-------TRLSRPLSRLP---GKTLSACDRENGArrPL  1p.1
SDR9C2        --MSTFFSDTAWICLAVPTVLCGTVFCKYKKSSGQLWSWMVCLAGLCAVCLLLILSPFWGL
SDR9C3        MERWPWPSGGAWLLVAARALLQL--LRSDLRLGRPLLAALALLAALDWLCQRLLPPPAAL
SDR9C4        ----------------------------------------------------MLFWVL-
SDR9C5        ------------------------------------------------------MWLPL
SDR9C6        ------------------------------------------------------MWLYL
SDR9C7        -----------------------------------------------------------M
SDR9C8        ------------------------------------------------------MWLYL

                                             ×   × ×
SDR9C1        LLGS-TSFIPIGRRTYASAAEPVGSKAVLVTGCDSGFGFSLAKHLHSKGFLVFAGCLMKD  2p.0  3p.0
SDR9C2        ILFSVSCFLMYTYLSGQ-ELLPVDQKAVLVTGGDCGLGHALCKYLDELGFTVFAGVLNEN  1p.2
SDR9C3        AVLAAAGWIALSRLARP-QRLPVATRAVLITGCDSGFGKETAKKLDSMGFTVLATVLELN  1p.2
SDR9C4        --GLLILCGFLWTRKGKLKIEDITDKYIFITGCDSGFGNLAARTFDKKGFHVIAACLTE-
SDR9C5        LLGA-LLWAVLWLLRDR-QSLPASNAFVFITGCDSGFGRLLALQLDQRGFRVLASCLTP-
SDR9C6        AAFVGLYYLLHWYRERQ-VVSHLQDKYVFITGCDSGFGNLLARQLDARGLRVLAACLTE-
SDR9C7        AALTDLSFMYRWFKNCN-LVGNLSEKYVFITGCDSGFGNLLAKQLVDRGMQVLAACFTE-
SDR9C8        AVFVGLYYLLHWYRERQ-VLSHLRDKYVFITGCDSGFGKLLARQLDARGLRVLAACLTE-
                                            ** * * *          *  * *

SDR9C1        KGHDGVKELDSLNSDRLRTVQLNVCSSEEVEKVVEIVRSSLKDPEKGMWGLVNNAGIST-  4p.2
SDR9C2        --GPGAEELRRTCSPRLSVLQMDITKPVQIKDAYSKVAAML--QDRGLWAVINNAGVLGF  2p.2
SDR9C3        --SPGAIELRTCCSPRLRLLQMDLTKPGDISRVLEFTKAHT--TSTGLWGLVNNAGHNEV  2p.2
SDR9C4        ---SGSTALKAETSERLRTVLLDVTDPENVKRTAQWVKNQV--GEKGLWGLINNAGVPGV  1p.2
SDR9C5        ---SGAEDLQRVASSRLHTTLLDITDPQSVQQAAKWVEMHV--KEAGLFGLVNNAGVAGI  1p.2
SDR9C6        ---KGAEQLRGQTSDRLETVTLDVTKMESIAAATQWVKEHV--GDRGLWGLVNNAGILTP  1p.2
SDR9C7        ---EGSQKLQRDTSYRLQTTLLDVTKSESIKAAAQWVRDKV--GEQGLWALVNNAGVGLP  1p.2
SDR9C8        ---KGAEQLRGQTSDRLETVTLDVTKTESVAAAAQWVKECV--RDKGLWGLVNNAGISLP  1p.2
                  *   *    * **                              *     ****
                                                                    +
SDR9C1        FGEVEFTSLETYKQVAEVNLWGTVRMTKSFLPLIRRAKGRVVNISSMLGRMANPARSPYC  5p.2
SDR9C2        PTDGELLLMTDYKQCMAVNFFGTVEVTKTFLPLLRKSKGRLVNVSSMGGGAPMERLASYG  3p.2
SDR9C3        VADAELSPVATFRSCMEVNFFGALELTKGLLPLLRSSRGRIVTVGSPAGDMPYPCLGAYG  3p.2
SDR9C4        LAPTDWLTLEDYREPIEVNLFGLISVTLNMLPLVKKAQGRVINVSSVGGRLAIV-GGGYT
SDR9C5        IGPTPWLTRDDFQRVLNVNTMGPIGVTLALLPLLQQARGRVINITSVLGRLAAN-GGGYC
SDR9C6        ITLCEWLNTEDSMNMLKVNLIGVIQVTLSMLPLVRRARGRIVNVSSILGRVAFF-VGGYC
SDR9C7        SGPNEWLTKDDFVKVINVNLVGLIEVTLHMLPMVKRARGRVVNMSSSGGRVAVI-GGGYC
SDR9C8        TAPNELLTKQDFVTILDVNLLGVIDVTLSLLPLVRRARGRVVNVSSVMGRVSLF-GGGYC
                               **  *    *   **      **     *  *         *

                +
SDR9C1        ITKFGVEAFSDCLRYEMYPLGVKVSVVEPGNFIAATSLYSPESIQAIAKKMWEELPEVVR
SDR9C2        SSKAAVTMFSSVMRLELSKWGIKVASIQPGGFLTN-IAGTSDKWEKLEKDILDHLPAEVQ  4p.2
SDR9C3        TSKAAVALLMDTFSCELLPWGVKVSIIQPGCFKTE-SVRNVGQWEKRKQLLLANLPQELL  4p.2
SDR9C4        PSKYAVEGFNDSLRRDMKAFGVHVSCIEPGLFKT--NLADPVKVIEKKLAIWEQLSPDIK  2p.1
SDR9C5        VSKFGLEAFSDSLRRDVAHFGIRVSIVEPGFFRT--PVTNLESLEKTLQACWARLPPATQ  2p.1
SDR9C6        VSKYGVEAFSDILRREIQHFGVKISIVEPGYFRT--GMTNMTQSLERMKQSWKEAPKHIK  2p.1
SDR9C7        VSKFGVEAFSDSIRRELYYFGVKVCIIEPGNYRT--AILGKENLESRMRKLWERLPQETR  2p.1
SDR9C8        ISKYGVEAFSDSLRRELSYFGVKVAMIEPGYFKT--AVTSKERFLKSFLEIWDRSSPEVK  2p.1
                *                 *       **
```

```
SDR9C1        KDYGKKYFDEKIAKMET-YCSSGSTDTSPVIDAVTHALTATTPYTRYHPMDYYWWLRM--
SDR9C2        EDYGQDYILAQRNFLLL-INSLASKDFSPVLRDIQHAILAKSPFAYYTPGKGAYLWIC--
SDR9C3        QAYGKDYIEHLHGQFLH-SLRLAMSDLTPVVDAITDALLAARPRRRYYPGQGLGLMYF--
SDR9C4        QQYGEGYIEKSLDKLKG-NKSYVNMDLSPVVECMDHALTSLFPKTHYAAGKDAKIFWI--  3p.2
SDR9C5        AHYGGAFLTKYLKMQQRIMNLICDPDLTKVSRCLEHALTARHPRTRYSPGWDAKLLWL--  3p.2
SDR9C6        ETYGQQYFDALYNIMKE-GLLNCSTNLNLVTDCMEHALTSVHPRTRYSAGWDAKFFFI--  3p.2
SDR9C7        DSYGEDYFRIYTDKLKN-IMQVAEPRVRDVINSMEHAIVSRSPRIRYNPGLDAKLLYI--  3p.2
SDR9C8        EAYGEKFVADYKKSAEQ-MEQKCTQDLSLVTNCMEHALIACHPRTRYSAGWDAKLLYL--  3p.2
                **                         *      *     *   *

SDR9C1        QIMTHLPGAISDMIYIR---------------------------------------
SDR9C2        -LAHYLPIGIYDYFAKRHFGQDKPMPRALRMPNYKKKAT-------------------
SDR9C3        -IHYYLPEGLRRRFLQAFFIS-HCLPRALQPGQPGTTPPQDAAQDPNLSPGPSPAVAR
SDR9C4        -PLSHMPAALQDFLLLKQKAELANPKAV------------------------------
SDR9C5        -PASYLPASLVDAVLTWVLPKPAQAVY-------------------------------
SDR9C6        -PLSYLPTSLADYILTRSWPKPAQAV--------------------------------
SDR9C7        -PLAKLPTPVTDFILSRYLPRPADSV--------------------------------
SDR9C8        -PMSYMPTFLVDAIMYWVSPSPAKAL--------------------------------
                    *
```

Figure S2. Alignment of the human SDR9C family protein variants. For further details see Fig. S1.

* symbol marks the position of identical amino acid residues of the aligned SDR protein sequences.

```
                             ×  ×  ×
SDR10E1      MVSIPEYYEGKNVLLTGATGFLGKVLLEKLLRSCPKVNSVYVLVRQKAGQTPQERVEEVL
SDR10E2      MSTIAAFYGGKSILITGATGFLGKVLMEKLFRTSPDLKVIYILVRPKAGQTLQQRVFQIL
             *  *   * **  * *********** *** *  *     * *** ***** * **   *

SDR10E1      SGKLFDRLRDENPDFREKIIAINSELTQPKLALSEEDKEVIIDSTNIIFHCAATVRFNEN  1p.0
SDR10E2      DSKLFEKVKEVCPNVHEKIRAIYADLNQNDFAISKEDMQELLSCTNIIFHCAATVRFDDT  1p.0
               ***       *   *** **   * *   * * **       *************

SDR10E1      LRDAVQLNVIATRQLILLAQQMKNLEVFMHVSTAYAYCNRKHIDEVVYPPPVDPKKLIDS  2p.1
SDR10E2      LRHAVQLNVTATRQLLLMASQMPKLEAFIHISTAYSNCNLKHIDEVIYPCPVEPKKIIDS  2p.1
             ** ****** ***** * * **  ** * * ****  ** ****** ** ** *** ***
                                    +   +
SDR10E1      LEWMDDGLVNDITPKLIGDRPNTYIYTKALAEYVVQQEGAKLNVAIVRPSIVGASWKEPF  3p.1
SDR10E2      LEWLDDAIIDEITPKLIRDWPNIYTYTKALGEMVVQQESRNLNIAIIRPSIVGATWQEPF  3p.1
             *** **     ****** * ** * ***** * *****   ** ** ******* * ***

SDR10E1      PGWIDNFNGPSGLFIAAGKGILRTIRASNNALADLVPVDVVVNMSLAAAWYSGVNRPRNI  4p.0 5p.0 6p.1
SDR10E2      PGWVDNINGPNGIIIATGKGFLRAIKATPMAVADVIPVDTVVNLMLAVGWYTAVHRPKST  4p.0 5p.0 6p.1
              *** ** *** *  ** *** ** * *   * **  *** ***  **  **  * **

SDR10E1      MVYNCTTGSTNPFHWGEVEYHVISTFKRNPLEQAFRRPNVNLTSNHLLYHYWIAVSHKAP  7p.2
SDR10E2      LVYHITSGNMNPCNWHKMGVQVLATFEKIPFERPFRRPNANFTSNSFTSQYWNAVSHRAP  7p.2
              **  * *  **  *      *  **   * *  ***** * ***     ** **** **

SDR10E1      AFLYDIYLRMTGRSPRMMKTITRLHKAMVFLEYFTSNSWVWNTENVNMLMNQLNPEDKKT  8p.1 9p.0
SDR10E2      AIIYDCYLRLTGRKPRMTKLMNRLLRTVSMLEYFINRSWEWSTYNTEMLMSELSPEDQRV  8p.1 9p.0
             *  ** *** *** *** *   **      ****   ** * * *  ***  * ***

SDR10E1      FNIDVRQLHWAEYIENYCLGTKKYVLNEEMSGLPAARKHLNKLRNIRYGFNTILVILIWR  10p.1
SDR10E2      FNFDVRQLNWLEYIENYVLGVKKYLLKEDMAGIPKAKQRLKRLRNIHYLFNTALFLIAWR  10p.1
             ** ***** * ****** ** *** * * * * * *   *  **** * *** *    **

SDR10E1      IFIARSQMARNIWYFVVSLCYKFLSYFRASSTMRY
SDR10E2      LLIARSQMARNVWFFIVSFCYKFLSYFRASSTLKV
               ********* * * ** *************
```

Figure S3. Alignment of the human SDR10E protein variants. For further details see Fig. S1.

* symbol marks the position of identical amino acid residues of the aligned SDR protein sequences.

```
                             ×  ×  ×
SDR11E1       ------MTGWSCLVTGAGGFLGQRIIRLLV-KEKELKEIRVLDKAFGPELREEFSKLQNK   1p.1
SDR11E2       -------MGWSCLVTGAGGLLGQRIVRLLV-EEKELKEIRALDKAFRPELREEFSKLQNR   1p.1
SDR11E3       MADSAQAQKLVYLVTGGCGFLGEHVVRMLLQREPRLGELRVFDQHLGPWLEELKTGP---   1p.1
                          ****  * **    * *   *  * * *  *    * * *

SDR11E1       TKLTVLEGDILDEPFLKRACQDVSVIIHTACIIDVFGVTHRESIMNVNVKGTQLLLEACV   2p.1
SDR11E2       TKLTVLEGDILDEPFLKRACQDVSVVIHTACIIDVFGVTHRESIMNVNVKGTQLLLEACV   2p.1
SDR11E3       VRVTAIQGDVTQAHEVAAAVAGAHVVIHTAGLVDVFGRASPKTIHEVNVQGTRNVIEACV   2p.1
                 *   **         *     * ****   ****      *  *** **    ****
                                                       +   +
SDR11E1       QASVPVFIYTSSIEVAGPNSYKEIIQNGHEEEPLENTWPAPYPHSKKLAEKAVLAANGWN
SDR11E2       QASVPVFIYTSSIEVAGPNSYKEIIQNGHEEEPLENTWPTPYPYSKKLAEKAVLAANGWN
SDR11E3       QTGTRFLVYTSSMEVVGPNTKGHPFYRGNEDTPYEAVHRHPYPCSKALAEWLVLEANGRK  3p.2  4p.0
              *       **** ** ***        * *  * *     *** ** ***  ** ***

SDR11E1       LKNGGTLYTCALRPMYIYGEGSRFLSASINEALNNNGILS-SVGKFSTVNPVYVGNVAWA
SDR11E2       LKNGDTLYTCALRPTYIYGEGGPFLSASINEALNNNGILS-SVGKFSTVNPVYVGNVAWA
SDR11E3       VRGGLPLVTCALRPTGIYGEGHQIMRDFYRQGLRLGGWLFRAIPASVEHGRVYVGNVAWM  5p.1
                 *  * ******  *****           *   * *            ********

SDR11E1       HILALRALQDPKKAPSIRGQFYYISDDTPHQSYDNLNYTLSKEFGLRLDSRW-SFPLSLM
SDR11E2       HILALRALRDPKKAPSVRGQFYYISDDTPHQSYDNLNYILSKEFGLRLDSRW-SLPLTLM
SDR11E3       HVLAARELEQ--RATLMGGQVYFCYDGSPYRSYEDFNMEFLGPCGLRLVGARPLLPYWLL
              * ** * *     *    ** *   *  *  **   *       ****       *  *

SDR11E1       YWIGFLLEIVSFLLRPIYTYRPPFNRHIVTLSNSVFTFSYKKAQRDLAYKPLYSWEEAKQ
SDR11E2       YWIGFLLEVVSFLLSPIYSYQPPFNRHTVTLSNSVFTFSYKKAQRDLAYKPLYSWEEAKQ
SDR11E3       VFLAALNALLQWLLRPLVLYAPLLNPYTLAVANTTFTVSTDKAQRHFGYEPLFSWEDSRT
                   *      ** *   * *  *       *  ** *  ****   * ** ***

SDR11E1       KTVEWVGSLVDRHKETLKSKTQ
SDR11E2       KTVEWVGSLVDRHKETLKSKTQ
SDR11E3       RTILWVQAATGSAQ--------
               *  **
```

Figure S4. Alignment of the human SDR11E protein variants. For further details see Fig. S1.

* symbol marks the position of identical amino acid residues of the aligned SDR protein sequences.

```
SDR12C1  --------------------MESALPAAGFLYWVGAGTVAYLALRISYSLFTALRVWGVG
SDR12C2  MGDV----------------LEQFFILTGLLV-----CLACLAKCVRFSRCVLLNYWKVL
SDR12C3  MAAVDSFYLLYREIARSCNCYMEALALVGA--WYTARKS-ITVICDFYSLIRLHFIPRLG
                                     *                   *

                         ×   × ×
SDR12C1  NEAGVGPGLGEWavVTGSTDGIGKSYAEelAKHGMKVVLISRSKDKLDQVSSEikEKFKV  1p.2 2p.0 3p.2
SDR12C2  P-KSFLRSMGQWavITGAGDGIGKAYSFelAKRGLNVVLISRTLEKLEAIATEieRTTGR  1p.2 2p.0 3p.2
SDR12C3  SRADLIKQYGRWAVVsgATDGIGKAYAEELASRGLNIILISRNEEKLQVVAKDIADTYKV  1p.1
                  * ***  *  ***** *  ***  *    ****   **      *

SDR12C1  ETRTIAVDFAS-EDIYDKIKTGLAGLEIGIlvNNVGMSYE-YPEYFLDVPDLDnvIKKMI  4p.2 5p.0
SDR12C2  SVKIIQADFTK-DDIYEHIKEKLAGLEIGIlvNNVGMLPNLLPSHFLNAPDEIqsL---I  4p.2 5p.0
SDR12C3  ETDIIVADFSSGREIYLPIREALKDKDVGILVNNVGVFYP-YPQYFTQLSE--DKLWDII
             *  **     **  *   *     ********      *  *             *
                                                    +   +
SDR12C1  NINILSVCkmTQLVLPGMVErsKGAILNISSGSGMLPVPLLTIYSATktFVDFFSQCLHE  6p.0 7p.1 8p.0
SDR12C2  HCNITSVVkmTQLILKHMESrqKGLILNISSGIALFPWPLYSMYSASkaFVCAFSKALQE  6p.0 7p.1 8p.0
SDR12C3  NVNIAAASLMVHVVLPGMVERKKGAIVTISSGSCCKPTPQLAAFSASkaYLDHFSRALQY            2p.0
           **     *    *  *  * ** *  ****    * *     ** *     **  *

SDR12C1  EYRSKGVFVqsVLPYFVATKLAKI----RKPTLDKPSPETFVKSAIKTVGLQSRTNGYLI  9p.0
SDR12C2  EYKAKEVIIqvLTPYAVSTAMTKYL----NTNVITKTADEFVKESLNYVTIGGETCGCLA  9p.0
SDR12C3  EYASKGIFVQSLIPFYVATSMTAPSNFLHRCSWLVPSPKVYAHHAVSTLGISKRTTGYWS
         **  *    *   *  * *                                   * *

SDR12C1  HALmgSIISNLPSWIYLKIVMNMNKSTRAHYLKKTKKN----  10p.0
SDR12C2  HEIlaGFLSLIPAWAFYSGAFQ--RLLLTHYVAYLKLNTKVR  10p.0
SDR12C3  HSIqfLFAQYMPEWLWVWGANILNRSLRKEALSCTA------   3p.0
         *          * *
```

Figure S5. Alignment of the human the SDR12C1 protein variants. For further details see Fig. S1 and Table S5.

* symbol marks the position of identical amino acid residues of the aligned SDR protein sequences.

```
                                                          ×   × ×
SDR16C1     --MVWKRLGALVMFPLQMIYLVVKAAVGLVLPAKLRDLSRENVLITGGGRGIGRQLAREF
SDR16C2     ----MKFLLDILLLLPLLIVCSLESFVKLFIPKRRKSVTGEIVLITGAGHGIGRLTAYEF
SDR16C3     ----MNIILEILLLLITIIYSYLESLVKFFIPQRRKSVAGEIVLITGAGHGIGRQTTYEF
SDR16C4     ----MNIVVEFFVVTFKVLWAFVLAAARWLVRPKEKSVAGQVCLITGAGSGLGRLFALEF
SDR16C5     MSFNLQSSKKLFIFLGKSLFSLLEAMIFALLPKPRKNVAGEIVLITGAGSGLGRLLALQF
                                                       **** * * **     *

SDR16C1     AERGARKIVLWGRTEKCLKETTEEIRQM-------------------------GTECHYF  1p.0
SDR16C2     AKLKS-KLVLWDINKHGLEETAAKCKGL-------------------------GAKVHTF  1p.0
SDR16C3     AKRQS-ILVLWDINKRGVEETAAECRKL-------------------------GVTAHAY  1p.0
SDR16C4     ARRRA-LLVLWDINTQSNEETAGMVRHIYRDLEAADAAALQAGNGEEEILPHCNLQVFTY  1p.1
SDR16C5     ARLGS-VLVLWDINKEGNEETCKMAREA------------------------GATRVHAY
            *       ***        **

SDR16C1     ICDVGNREEVYQTAKAVREKVGDITILVNNAAVVHGKSLMDSDDDALLKSQHINTLGQFW  2p.0 3p.0
SDR16C2     VVDCSNREDIYSSAKKVKAEIGDVSILVNNAGVVYTSDLFATQDPQIEKTFEVNVLAHFW  2p.0 3p.2
SDR16C3     VVDCSNREEIYRSLNQVKKEVGDVTIVVNNAGTVYPADLLSTKDEEITKTFEVNILGHFW  2p.0 3p.0
SDR16C4     TCDVGKRENVYLTAERVRKEVGEVSVLVNNAGVVSGHHLLECPDELIERTMMVNCHAHFW  2p.0
SDR16C5     TCDCSQKEGVYRVADQVKKEVGDVSILINNAGIVTGKKFLDCPDELMEKSFDVNFKAHLW  1p.0 2p.0
              *    *  *     *    *      ***  *         *         *     *
                                               +   +
SDR16C1      TTKAFLPRMLELQNGHIVCLNSVLALSAIPGAIDYCTSKASAFAFMESLTLGL--LDCPG
SDR16C2      TTKAFLPAMTKNNHGHIVTVASAAGHVSVPFLLAYCSSKFAAVGFHKTLTDELAALQITG  4p.2
SDR16C3      ITKALLPSMMERNHGHIVTVASVCGHEGIPYLIPYCSSKFAAVGFHRGLTSELQALGKTG  4p.2
SDR16C4      TTKAFLPTMLEINHGHIVTVASSLGLFSTAGVEDYCASKFGVVGFHESLSHELKAAEKDG  3p.0
SDR16C5      TYKAFLPAMIANDHGHLVCISSSAGLSGVNGLADYCASKFAAFGFAESVFVETFVQKQKG  3p.1
               ** ** *     ** *   *            ** **     *              *

SDR16C1      VSATTVLPFHTSTEMFQGMRVRFPN--LFPPLKPETVARRTVEAVQLNQALLLLPWTMHA  4p.2
SDR16C2      VKTTCLCPNFVNTGFIKNPSTSL-----GPTLEPEEVVNRLMHGILTEQKMIFIPSSIAF  5p.2
SDR16C3      IKTSCLCPVFVNTGFTKNPSTRL-----WPVLETDEVVRSLIDGILTNKKMIFVPSYINI  5p.2
SDR16C4      IKTTLVCPYLVDTGMFRGCRIRKEIEPFLPPLKPDYCVKQAMKAILTDQPMICTPRLMYI  4p.2
SDR16C5      IKTTIVCPFFIKTGMFEGCTTGCPS--LLPILEPKYAVEKIVEAILQEKMYLYMPKLLYF  4p.2
                    *    *                * *                      *

SDR16C1      LVILKSILPQAALEEIHKFSGTYTCMNTFKGRT-------------  5p.2
SDR16C2      LTTLERILPERFLAVLKQKISVKF--DAVIGYKMKAQ---------  6p.2
SDR16C3      FLRLQKFLPERASAILNRMQNIQF--EAVVGHKIKMK---------  6p.2
SDR16C4      VTFMKSILPFEAVVCMYRFLGADKCMYPFIAQRKQATNNNEAKNGI  5p.2
SDR16C5      MMFLKSFLPLKTGLLIADYLGILHAMDGFVDQKKKL----------  5p.2
                    **
```

Figure S6. Alignment of the human SDR16C protein variants. For further details see Fig. S1.

* symbol marks the position of identical amino acid residues of the aligned SDR protein sequences.

```
                        ×   × ×
SDR21C1      MSSGIHVALVTGGNKGIGLAIVRDLCRLFSGDVVLTARDVTRGQAAVQQLQAEGLSPRFH
SDR21C2      MSSCSRVALVTGANRGIGLAIARELCRQFSGDVVLTARDVARGQAAVQQLQAEGLSPRFH
             ***   ****** * ****** * *** ************ *******************

SDR21C1      QLDIDDLQSIRALRDFLRKEYGGLDVLVNNAGIAFKVADPTPFHIQAEVTMKTNFFGTRD   1p.1
SDR21C2      QLDIDDLQSIRALRDFLRKEYGGLNVLVNNAAVAFKSDDPMPFDIKAEMTLKTNFFATRN   1p.1
             ************************ ******  ***  ** ** * ** * ***** **

SDR21C1      VCTELLPLIKPQGRVVNVSSIMSVRALKSCSPELQQKFRSETITEEELVGLMNKFVEDTK   2p.1
SDR21C2      MCNELLPIMKPHGRVVNISSLQCLRAFENCSEDLQERFHSETLTEGDLVDLMKKFVEDTK   2p.1
              * ****  ** ***** **    **   **  **  * *** **  ** ** *******
                          +   +
SDR21C1      KGVHQKEGWPSSAYGVTKIGVTVLSRIHARKLSEQRKGDKILLNACCPGWVRTDMAGPKA
SDR21C2      NEVHEREGWPNSPYGVSKLGVTVLSRILARRLDEKRKADRILVNACCPGPVKTDMDGKDS
               **  **** * *** * ******** ** * * ** * ** ****** * *** *

SDR21C1      TKSPEEGAETPVYLALLPPDAEGPHGQFVSEKRVEQW
SDR21C2      IRTVEEGAETPVYLALLPPDATEPQGQLVHDKVVQNW
                 *****************  * ** *  * *  *
```

Figure S7. Alignment of the human SDR21C protein variants. For further details see Fig.S1.

* symbol marks the position of identical amino acid residues of the aligned SDR protein sequences.

```
                                                          ×   × ×
SDR25C1Homo      MLSAVARGYQGWFHPCARLSVRMSSTGIDRKGVLANRVAVVTGSTSgiGFAIARRLARDG  1p.1
SDR25C2Homo      MHKAGLLGLCA----RAWNSVRMASSGMTRRDPLANKVALVTASTDgiGFAIARRLAQDG  1p.1
                 *  *   *        *  **** *    *   * * ** ** ** *********** **

SDR25C1Homo      AHVVISSRKQQNVDRAMAKLQGEGLSVAGIVCHVGKAEDREQLVAkaLEHCGGVDFLVCS  2p.0
SDR25C2Homo      AHVVVSSRKQQNVDQAVATLQGEGLSVTGTVCHVGKAEDRERLVAtaVKLHGGIDILVSN  2p.0
                 **** ********* * * ******** * *********** *** *    ** * **

SDR25C1Homo      AGVNPLVGSTLGTSEQIWDkiLSVNVKSPALLLSQLLPYMEN-rrGAVILVSSIAAYNPV  3p.0 4p.1
SDR25C2Homo      AAVNPFFGSIMDVTEEVWDktLDINVKAPALMTKAVVPEMEKRggGSVVIVSSIAAFSPS  3p.0 4p.1
                 * ***  **     *  *** *  *** ***      * **    * *  ******  *
                      +   +
SDR25C1Homo      vaLGVYNVSKTALLGLTRTLALELAPKDIRVNCVVPGIIKTDFSKvf-HGNESLWKNFKE  5p.0 6p.0
SDR25C2Homo      pgFSPYNVSKTALLGLTKTLAIELAPRNIRVNCLAPGLIKTSFSRml-WMDKEKEESMKE  5p.0 6p.0
                      ***********  *** ****  *****

SDR25C1Homo      HHQLQriGESEDCAGIVSFLCSPDASYVNGENIAVAGYSTRL-  7p.1
SDR25C2Homo      TLRIRrlGEPEDCAGIVSFLCSEDASYITGETVVVGGGTPSRL  7p.1
                      * ** ************ ****  **   * *
```

Figure S8. Alignment of the human SDR25C protein variants. For further details see Fig. S1 and Table S8.

* symbol marks the position of identical amino acid residues of the aligned SDR protein sequences.

```
                                                   ×   × ×
SDR26C1    MAFMKKYLLPILGLFMAYYYYSANEEFRpeMLQGKKVIVTGASKGIGREMAYHLAKMGAH  1p.2
SDR26C2    ---MKVLLLTGLGAL--FFAYYWDDNFDpaSLQGARVLLTGANAGVGEELAYHYARLGSH  1p.2
              **  **  **       *     * *  ***  *  ***  * * * *** *  * *

SDR26C1    VVVTARSKETLQkvVSHCLELGAASAHYIAGTMEDMTFAEQFVAQAGKLmgGLDMLILNH  2p.0 3p.2
SDR26C2    LVLTAHTEALLQkvVGNCRKLGAPKVFYIAADMASPEAPESVVQFALDKlgGLDYLVLNH  2p.0 3p.2
            * **     *****  *  ***    ***  *      *  *  *    **** * ***

SDR26C1    ITNTSLNLFHDDIHHVRKSMEVNFLSYVVLTVAALPMLKQSNGSIVVVSSLagKVAYPMV       4p.2
SDR26C2    IGGAPAGTRARSPQATRWLMqvNFVSYVQLTSRALPSLTDSKGSLVVVSSLlgRVPTSFS  4p.0 5p.2
           *               *  * *** *** **  *** *  * ** ****** * *
                               +   +
SDR26C1    AAYSASKFALDGFFSSIRKEYSVSRVNVSITLCVLGLIDteTAMKAVSGIVHMQAAPKEE  5p.2
SDR26C2    TPYSAAKFALDGFFGSLRRELDVQDVNVAITMCVLGLRDRASAAEAVrsSTSRPRQPEHR  6p.2
             *** ******** * * *  *  *** ** ***** *   *  **         *

SDR26C1    CA-LEI---------IKGGALR------------------------QEEVYYDSSLWTTL
SDR26C2    GVPLQSQTAMFLPPTVPGARTLTETPLRGWPQPKMKSSRQKSKTEKNDGHLEPVTAWEVQ
              *             *                                      *

SDR26C1    L--IRNPCRKILEFLYSTSYNMDRFINK
SDR26C2    VPRVRRLCRGLARP---HLFGHD-----
               *  **             *
```

Figure S9. Alignment of the human SDR26C1 protein variants. For further details see Fig. S1.

* symbol marks the position of identical amino acid residues of the aligned SDR protein sequences.

```
                                          ×   × ×
SDR28C1    ----------------------MARTVVLITGCSSGIGLHLAVRLASDPSQSFKVYATLR  1p.2
SDR28C2    MNGQSQVLPGGGHESREGINMAAAPRTVLISGCSSGIGLELAVQLAHDPKKRYQVVATMR  1p.2
                                  *   *** ******** *** ** **     * ** *

SDR28C1    DLKTQGRLWEAARALACPPGSLETLQLDVRDSKSVAAARERVTEGRVDVLVCNAGLGLLG  2p.2
SDR28C2    DLGKKETLEAAAGEAL--GQTLTVAQLDVCSDESVAQ-CLSCIQGEVDVLVNNAGMGLVG  2p.2
           **     *  **         *   ****    ***        * ***** *** ** *
                                                                    +
SDR28C1    PLEALGEDAVASVLDVNVVGTVRMLQAFLPDMKRRGSGRVLVTGSVGGLMGLPFNDVYCA  3p.2
SDR28C2    PLEGLSLAAMQNVFDTNFFGAVRLVKAVLPGMKRRRQGHIVVISSVMGLQGVIFNDVYAA  3p.2
           *** *   *   * * *  * **   * ** ****  *   *  ** ** *  ***** *
            +
SDR28C1    SKFALEGLCESLAVLLLPFGVHLSLIECGPVHTAFMEKVLGSPEE-VLDRTDIHTFHRFY  4p.1
SDR28C2    SKFALEGFFESLAIQLLQFNIFISLVEPGPVVTEFEGKLLAQVSMAEFPGTDPETLHYFR  4p.1
           *******  ****  ** *    ** * *** * *  * *          **  * * *

SDR28C1    -QYLAHSKQVFREAAQNPEEVAEVFLTALRAPKPTLRYFTTERFLPLLRMRLDDPSGSNY  5p.0
SDR28C2    DLYLPASRKLFCSVGQNPQDVVQAIVNVISSTRPPLRRQTNIRYSPLTTLKTVDSSGSLY  5p.0
             **   *   *    ***  *            * **  *  *  **      * *** *

SDR28C1    VTAMHREVFGDVPAKAEAGAEA-GGGAGPGAEDEAGRGAVGDPELGDPPAAPQ
SDR28C2    VRTTHRLLFRCPRLL-NLGLQCLSCGCLPTRVRPR------------------
           *   **  *         *      *  *
```

Figure S10. Alignment of the human SDR28C protein variants. For further details see Fig. S1.

* symbol marks the position of identical amino acid residues of the aligned SDR protein sequences.

```
                                                                     ×
SDR32C1    MVSPATRKSLPKVKAMDFITSTAILPLLFGCLGVFGLFRLLQWVRGKAYLRNAVVVITGA  1p.1
SDR32C2    ---------------MGVMAMLMLPLLLLGISGLLFIYQEVSRLWSKSAVQNKVVVITDA
                          *          ** *  *             *    * ***** *

             × ×
SDR32C1    TSGLGKECAKVFYAAGAKLVLCGRNGGALEELIRELTASHATKVQTHKPYLVTFDLTDSG  2p2 3p.0
SDR32C2    ISGLGKECARVFHTGGARLVLCGKNWERLENLYDALISVADPSKQTFTPKLVLLDLSDIS  1p.2 2p.0
            ******** **   ** ***** *   ** *   *        **  * **  ** *

SDR32C1    AIVAAAAEILQCFGYVDILVNNAGISYRGTIMDTTVDVDKRVMETNYFGPVALTKALLPS  4p.2
SDR32C2    CVPDVAKEVLDCYGCVDILINNASVKVKGPAHKISLELDKKIMDANYFGPITLTKALLPN  3p.2
                * * * * * **** ***     *         **  *  *****  *******
                                     +   +
SDR32C1    MIKRRQGHIVAISSIQGKMSIPFRSAYAASKHATQAFFDCLRAEMEQYEIEVTVISPGYI  5p.2
SDR32C2    MISRRTGQIVLVNNIQGKFGIPFRTTYAASKHAALGFFDCLRAEVEEYDVVISTVSPTFI  4p.2
           ** ** * **    ****  ****  *******   ******** * *       **  *

SDR32C1    HTNLSVNA-ITADGSRYGVMDTTTAQGRSPVEVAQDVLAAVGKKKKDVILADLLPSLAVY  6p.2
SDR32C2    RSYHVYPEQGNWEASIWKFFFRKLTYGVHPVEVAEEVMRTVRRKKQEVFMANPIPKAAVY  5p.2
                         *           *  *****  *   *  **  *  *   *  ***

SDR32C1    LRTLAPGLFFSLMASRARKERKSKNS-
SDR32C2    VRTFFPEFFFAVVACGVKEKLNVPEEG
            **  *  **   *
```

Figure S11. Alignment of the human SDR32C protein variants. For further details see Fig. S1 and Table S11.

* symbol marks the position of identical amino acid residues of the aligned SDR protein sequences.

```
SDR42E1    ------------------------------------------------------------
SDR42E2    MKSNPPRSSLEACKAAGQGEKSCPVCQACGEVSGPRSGSGSESRPAPKPGAIPGPGLGPK


SDR42E1    ------------------------------------------------------------
SDR42E2    AIPGPQAGSGTVPRPGAISGTGPGLGPGPGAGSVPGPGAGSVPGLGARSVPGPGAGSVPG


SDR42E1    ------------------------------------------------------------
SDR42E2    PGAGSVPGPGAGSVPGPGAGSGPGLGGGLGPGVGAGPGAGSVPGPGAGSVPGPGAGSVPG


SDR42E1    -------------------------------------------------MDPKRSQKESV
SDR42E2    PGAGSVPGAGAGSTPEPELGPGLRQGSGTGPRPSESTTTPTPAPQQKTQAKPTKAARQKV
                                                              *       *
              ×  ×  ×
SDR42E1    LITGGSGYFGFrlGCALNQNGVHVILFDISSPAQTIPEGIKFIQGDIRHLSDVEKAFQDA  1p.1
SDR42E2    LVTGGGGYLGFSLGSHLAKSGTSVILLDRRRPQWELSPETKFIqaDVRDEEALYRAFE--  1p.2
           * *** ** ** **  *   *  *** *   *        ***  * *       **

SDR42E1    DVTCVFHIASYGMSGREQLNRNLIKEVNVRGTDNILQVCQRRRVPRLVYTSTFNVIFGGQ
SDR42E2    GVDCVFHVASYGMSGAEklQKEQIESINVGGTKLVIdvCVRRRVPRLIYTSTVNVAFGGK  3p.0  4p.2
            * **** ******* * *    *   ** **     ** ******* **** ** ***
                                       +   +
SDR42E1    VIRNGD-ESLPYLPLHLHPDHYSRTKSIAEQKVLEANATPLDRGDGVLRTCALRPAGIYG
SDR42E2    PIEQGDEDSVPYFPLDehVDHYSRTKAIADQLTLMANGMPL-pgGGTLRTCVLRPPGIYG  5p.0  6p.2
            *  **  * ** **  * ******* ** *  * **  **  * * **** *** ****

SDR42E1    PGEQRHLPRIVSYIEKGLFKFVYGDPRSLVEFVHVDNLVQAHILASEALRADKGHIASGQ
SDR42E2    PEEQRHLPRVagHIKKRLFMFRFGDHKARMNWVHVHNLVQAHVLAAEALTTAKGYVasGQ  7p.0  8p.0
           * *******    * * ** *  **       *** ****** ** ***   **  ****

SDR42E1    PYFISDGRPVNNFEFFRPLVEGLGYTFPSTRLPLTLVYCFAFLTEMVHFILGRLYNFQPF
SDR42E2    AYYINDGESVNLFEWMAPlfEKLGYSQPWIQVPTSWVYLtaAVMERLHLALRPICSLPPL  9p.0 10p.2
            * * **  ** **   ** * ***  *    *   **  *   *  *  *       *

SDR42E1    LTRTEVYKTGVTHYFSLEKAKKELGYKAQPFDLQEAVEWFKAHGHGRSSGSRDSECFVW-
SDR42E2    LTRSevRSVAVTHTFQIAKARAQLGYAPDKFRFADAVELYVQSTTRRPRGSTARTLLRLL  11p.0
           *** **    *** *   **   ***    *    ***        *  **

SDR42E1    DGLLVFLLIIAVLM-WLPSSVILS----L
SDR42E2    LRLLLFLGLLALALHFLGLQPLHAAVERL
             ** **   *     *           *
```

Figure S12. Alignment of the human SDR42E protein variants. For further details see Fig. S1.

* symbol marks the position of identical amino acid residues of the aligned SDR protein sequences.

Family symbol and name	Enzyme symbol	Gene symbol	Gene ID	Chr	Exon number	Phase formula1	aa n.	Structure consensus	Catalysis consensus
SDR7C Retinol dehydrogenase	SDR7C1	RDH11	51109	14	7	122221	318	GANTGIG	YCHSK
	SDR7C2	RDH12	145226				316		
	SDR7C3	RDH13	112724	19	7	122222	331		
	SDR7C4	RDH14	57665	12	2	0	336	GANSGLG	YSRSK
	SDR7C5	DHRS13	147015	17	5	2022	377	GANSGIG	YADTK
SDR40C Dehydrogenase/reductase SDR family	SDR40C1	DHRS12	79758	13	10	120020022	317	GGNSGIG	YAQNK

Table S3a. Genetic and molecular data of the human SDR7C family and of the human SDR40C1 protein variants. Chr, chromosome; Phase formula contains phase type symbols aligned according to the sequence of their relative splicing sites; conserved amino acids of the consensus sequences are in red; aa n., number of the variant polypeptide amino acids.

	% Identity				
	SDR7C1	SDR7C2	SDR7C3	SDR7C4	SDR7C5
SDR7C2	71.66				
SDR7C3	49.68	48.87			
SDR7C4	46.15	46.47	48.88		
SDR7C5	45.78	46.41	42.32	44.48	
SDR40C1	32.54	33.22	30.64	31.21	28.23

Table S3b. The relative percent identity of the human SDR7C family and of the human SDR40C1 protein variants.

Family symbol and name	Enzyme symbol	Gene symbol	Gene ID	Chr	Exon s	Phase formula	aa n.	Structure consensus	Catalysis consensus
SDR11E 3beta-hydroxysteroid dehydrogenase	SDR11E1	HSD3B1	3283	1	3	22	375	GAGGFLG	YPHSK
	SDR11E2	HSD3B2	3284				372	GAGGLLG	YPYSK
	SDR11E3	HSD3B7	80270	16	6	22201	369	GGCGFLG	YPCSK

Table S4a. Genetic and molecular data of the human SDR11E family protein variants. For further details see Table S3a.

	% Identity	
	SDR11E1	SDR11E2
SDR11E2	93.55	
SDR11E3	38.61	39.00

Table S4b. Relative percent identity of the SDR11E family protein variants.

Family symbol and name	Enzyme symbol	Gene symbol	Gene ID	Chr	Exons	Phase formula	% identity	aa	Structure consensus	Catalysis consensus
SDR10E Fatty acyl-CoA dehydrogenase	SDR10E1	FAR1	84188	11	11	0110012101	59.03%	515	GATGFLG	YIYTK
	SDR10E2	FAR2	55711	12					GATGFLG	YTYTK

Table S5. Genetic and molecular data of the human SDR10E family protein variants. For further details see Table S3a.

Family	Enzyme symbol	Gene Symbol	Gene ID	Chr	Exons n.	Phase formula	aa n.	Structure consensus	Catalysis consensus
SDR9C Steroid and retinol dehydrogenase	SDR9C1	BDH1	622	3	6	10022	343	GCDSGFG	YCITK
	SDR9C2	HSD17B2	3294	16	5	2222	387	GGDCGLG	YGSSK
	SDR9C3	HSD11B2	3291				405	GCDSGFG	YGTSK
	SDR9C4	DHRS9	10170	2	4	212	319	GCDSGFG	YTPSK
	SDR9C5	RDH5	5959	12			317		YCVSK
	SDR9C6	HSD17B6	8630						
	SDR9C7	SDR9C7	121214				313		
	SDR9C8	RDH16	8608				317		YCISK

Table S6a. Genetic and molecular data of the human SDR9C family protein variants. For further details see Table S3a.

	% Identity						
	SDR9C1	SDR9C2	SDR9C3	SDR9C4	SDR9C5	SDR9C6	SDR9C7
SDR9C2	32.93						
SDR9C3	32.34	38.54					
SDR9C4	35.53	28.80	29.25				
SDR9C5	34.87	30.06	32.81	43.63			
SDR9C6	38.16	32.18	32.18	44.90	49.37		
SDR9C7	36.67	31.31	30.03	44.19	47.76	50.80	
SDR9C8	37.17	32.81	33.12	46.18	51.58	66.25	51.76

Table S6b. Relative percent identity of the SDR9C family protein variants.

Family symbol and name	Enzyme symbol	Gene symbol	Gene ID	Chr	Exons n.	Phase formula	aa n.	Structure consensus	Catalysis consensus
SDR12C 3beta-hydroxysteroid dehydrogenase	SDR12C1	HSD17B12	51144	11	11	2022001000	312	GSTDGIG	YSATK
	SDR12C2	HSD17B3	3293	9			310	GAGDGIG	YSASK
	SDR12C3	HSDL1	83693	16	4	100	330	GATDGIG	FSASK

Table S7a. Genetic and molecular data of the human SDR12C family protein variants. Phenylalanine (F), diagnostic of the SDR12C3 catalysis consensus, is highlighted in turquoise. For further details see table S3a and Consensuses in Online Resources 1.

	% Identity	
	SDR12C1	SDR12C2
SDR12C2	41.67	
SDR12C3	40.98	35.33

Table S7b. Relative percent identity of the SDR12C family protein variants.

Family Symbol and name	Enzyme symbol	Gene symbol	Gene ID	Chr	Exons	Phase formula	aa n.	Structure consensus	Catalysis consensus
SDR16C Steroid and retinol dehydrogenase	SDR16C1	DHRS3	9249	1	6	00011	302	GGGRGIG	YCTSK
	SDR16C2	HSD17B11	51170	4	7	000111	300	GAGHGIG	YCSSK
	SDR16C3	HSD17B13	345275						
	SDR16C4	RDH10	157506	8	6	20011	341	GAGSGLG	YCASK
	SDR16C5	SDR16C5	195814	8	6	00211	309		

Table S8a. Genetic and molecular data of the human SDR17C family protein variants. For further details see Table S3a.

	% Identity			
	SDR16C1	SDR16C2	SDR16C3	SDR16C4
SDR16C2	34.35			
SDR16C3	32.31	63.33		
SDR16C4	36.45	40.33	39.33	
SDR16C5	34.88	41.81	40.13	45.57

Table S8b. Relative percent identity of the SDR16C family protein variants.

Family symbol and name	Enzyme symbol	Gene symbol	Gene ID	Gene name	Chr	Exons	Phase formula	% Identity	aa n.	Structure consensus	Catalysis consensus
SDR21C Carbonyl reductase	SDR21C1	CBR1	873	Carbonyl reductase 1	21	3	22	71.84%	277	GGNKGIG	YGVTK
	SDR21C2	CBR3	874	Carbonyl reductase 3						GANRGIG	YGVSK

Table S9. Genetic and molecular data of the human SDR21C family protein variants. For further details see Table S3a.

Family symbol and name	Enzyme symbol	Gene Symbol	Gene ID	Chr	Exons	Phase formula	% identity	aa n.	Structure consensus	Catalysis consensus
SDR25C Dehydrogenase/reductase SDR family	SDR25C1	DHRS2	10202	14	8	1001001	59.27	280	GSTSGIG	YNVSK
	SDR25C2	DHRS4	10901					278	ASTDGIG	

Table S10. Genetic and molecular data of the human SDR25C family protein variants. Alanine (A), diagnostic of the SDR25C2 structure consensus, is highlighted in turquoise. For further details see Table S3a and Consensuses in Online Resources 1.

Family symbol and name	Enzyme symbol	Gene Symbol	Gene ID	Chr	Exons	Phase formula	% identity	aa n.	Structure consensus	Catalysis consensus
SDR26C Corticosteroid 11beta-dehydrogenase	SDR26C1	HSD11B1	3290	1	6	20222	41.22	292	GASKGIG	YSASK
	SDR26C2	HSD11B1L	374875	19	7	202022		315	GANAGVG	YSAAK

Table S11. Genetic and molecular data of the human SDR26C family protein variants. For further details see Table S3a.

Family symbol and name	Enzyme symbol	Gene Symbol	Gene ID	Chr	Exons	Phase formula[1]	% Id.	aa n.	Structure consensus	Catalysis consensus
SDR28C 17beta-hydroxysteroid dehydrogenase	SDR28C1	HSD17B1	3292	17	6	22210	44.12	328	GCSSGIG	YCASK
	SDR28C2	RDH8	50700	19		22210		331		YAASK

Table S12. Genetic and molecular data of the human SDR28C family protein variants. For further details see Table S3a.

Family symbol and name	Enzyme symbol	Gene	Gene ID	Chr	Exons	Phase formula	% identity	aa	Structure consensus	Catalysis consensus
SDR32C Dehydrogenase/ Reductase SDR Family	SDR32C1	DHRS7B	25979	17	6	110111	41.61	325	GATSGLG	YAASK
	SDR32C2	DHRS7C	201140		11	10111		312	DAISGLG	

Table S13. Genetic and molecular data of the human SDR32C family protein variants. Asparagine (D), diagnostic of the SDR32C2 structure consensus, is highlighted in turquoise. For further details see Table s3a and Consensuses in Online Resources 1.

Family symbol and name	Enzyme symbol	Gene symbol	Gene ID	Chr	Exons	Phase formula	aa n.	% identity	Structure consensus	Catalysis consensus
SDR42E 3-beta-HSD family	SDR42E1	SDR42E1	93517	16	2	1	393	47.18	GGSGYFG	YSRTK
	SDR42E2	SDR42E2	100288072		12	20020200020	626		GGGGYLG	

Table S14. Genetic and molecular data of the human SDR42E family protein variants. For further details see Table S3a.
