# Supplementary material for: Gene Structure Evolution of the Short-Chain Dehydrogenase/Reductase (SDR) Family
Source: Genes (Basel). 2022 Dec 30;14(1):110. doi: 10.3390/genes14010110 (PMC9859523; doi:10.3390/genes14010110)
Supplement: Supplementary file 1 [file genes-14-00110-s001.zip › Online Resources 3/Online Resources 3.pdf]

# VERTEBRATE SDR FAMILY VARIANTS.

```
SDR7C1  H. sapiens        ------MVELMF--PLLLLLLPFLLYMAAPQIrkML-SS--------GVCTSTVQLPGKV 1p.1
SDR7C1  G. gorilla        ------MVELMF--PLLLLLLPFLLYVAAPQIrkML-SS--------GVCTSTVQLPGKV 1p.1
SDR7C1  M. musculus       ---------MFG--FLLLLSLPFILYLVTPKIrkML-SS--------GVCTSNVQLPGKV 1p.1
SDR7C1  M. domestica      --------MFWL--LVLLASLVLLLVWATPYVreAV-SK--------GVCTSTVQLPGKV 1p.1
SDR7C1  P. formosa        --------MFVLLIIAGLGVVTLLVILFAPHIrrYA-AG--------AVCKSTARLDGKT 1p.1
SDR7C2  H. sapiens        --------MLVT--LGLLTSFFSFLYMVAPSIrkFF-AG--------GVCRTNVQLPGKV 1p.1
SDR7C2  P. troglodytes    --------MLVT--LGLLTSFFSFLYMVAPSIrkFF-AG--------GVCRTNVQLPGKV 1p.1
SDR7C2  M. musculus       --------MLFI--LVLLTSFLSILYLTAPSIrkFF-AG--------GVCTTNVQIPGKV 1p.1
SDR7C2  M. domestica      --------MIGI--MVGLTSFVSFLYMAGPYIrkFF-AG--------GVCTSTVQLPGKV 1p.1
SDR7C2  G. gallus         MEPAAGMLSCWGAVLGAAVSVPLLLLAAAPYVrrYV-AG--------GRCRSTARLDGKV 1p.1
SDR7C2  A. carolinensis   ------------------------MSSTFSPFrkWF-AG--------GVCTSTAMLHGKV 1p.1
SDR7C2  D. rerio          ------MMLALVAFAAGLGLVALILRLLSPQIrkYA-AG--------GSCRSTVRLDGKV 1p.1
SDR7C3  H. sapiens        ------MSR-----YLLPLSALGTVAGAAVLLkdYV-TG--------GACPSKATIPGKT 1p.1
SDR7C3  P. troglodytes    ------MSR-----YLLPLSALGTVAGAAVLLkdYV-TG--------GACPSKATIPGKT 1p.1
SDR7C3  M. musculus       ------MSR-----FLLPVSVVGTVIGGTVLLkdYV-AG--------GACPSKATIPGKT 1p.1
SDR7C3  X. tropicalis     ------MNK-----YVVRASMVGTALGGAILLkdYT-GG--------GNCPSKASIIGQT 1p.1
SDR7C3  O. latipes        ------MSK-----YILPVSVFGTVFGSAVLLkhHV-TG--------GRCPSKATITGKT 1p.1
SDR7C4  H. sapiens        ------MA-VATAAAVLAALGGALWLAARRFVGPRVQRL--------RRGGDPGLMHGKT
SDR7C4  P. troglodytes    ------MA-VATAAAVLAALGGALWLAARRFVGPRVQRL--------RRGGDPGLMHGKT
SDR7C4  M. musculus       ------MAVASVAAALLAALGGALWLAARRFSGPRNQRQ--------QGGGDPGLMHGKT
SDR7C4  M. domestica      MRSRRRAMVSLSSVLLAAALGGGLWLAVRRYLRSPGRTP----------AAGLMLMRGKT
SDR7C4  P. humilis        ------MAA----ALPALVLGAGLLVAAWRWLR----GA--------ARPGRGGSMRGKT
SDR7C4  A. carolinensis   --------MAATAVVLAAALGGGLLLIARRFWQVAA-GA--------GAGKVKRGMEGKT
SDR7C4  X. tropicalis     MAA----SSPALMVAAAVALGGGLILVVRRLLSNS------------VRAGGSGLMRGKT
SDR7C4  D. rerio          ---------MSAAVVLAAVLGGGVFFIARRIIFRRKALR--------LMSYPPALMRGKT
SDR7C5  H. sapiens        --------------MEALLLGAGLLLGAYVLVYYNLVKA--------PPCGGMGNLRGRT
SDR7C5  P. abelii         --------------MEALLLGAGLLLGAYVLVYYNLVKA--------PPCGGMGNLRGRT
SDR7C5  M. musculus       --------------MEMLLLGAGLLLGAYVLVYYNLVKA--------PSCGGIGSLRGRT
SDR7C5  M. domestica      -----------------------MLLGSYVLIYYNFIKA--------VPCMSPINLKGKT
SDR7C5  H. leucocephalus  --------------MGWTLLGAGLLLALYTLLRHGLRRS--------PPLRDRPELRGRT
SDR7C5  G. japonicus      ----------------MVLSGLALLTGLYVWLYYNFLRG--------PKCRNETSLRGKT
SDR7C5  X. tropicalis     --------------MVPVMLVVGLGIGAYILIYFNLIRG--------RQCRSDASLKGKT
SDR7C5  O. latipes        -------------MCGFILIVFALAAVAY-MFHDIVVKG--------KRCKSNANLNGKT
SDR40C1 H. sapiens        ------MSLYRS--------VVWFAK----GLREYTksGYESACKDFVPHDLEVQIPGRV 1p.1
SDR40C1 P. troglodytes    ------MSLYRS--------VVWFAK----GLREYTksGYESACKDFVPHDLEVQVPGRV 1p.1
SDR40C1 B. taurus         ------MLLYRS--------AAWFAK----GLREYTksGYESASKDFVPDDLEVQVPGRA 1p.1
SDR40C1 G. gallus         ------MSWYRN--------VVWFVK----GLREYTrsGYESASKHFDPADLEVDVAGRS 1p.1
SDR40C1 A. carolinensis   ------MSIYRN--------SVWFIK----GLNEYCrsGYESASKRFVPGDLEVDVTGRS 1p.1
SDR40C1 X. tropicalis     ------MSLYRN--------TIWFLK----GMREYTksGYETAAKQFLSEDLEFDVRGRS 1p.1
SDR40C1 O. latipes        ------MSIYRN--------AVWFMK----GLQEYTksGYEAAAKHFVPNDLDVNLTGRS 1p.1
                                                                                 *

                              ×   × ×
SDR7C1  H. sapiens        VVVTGANTGIGKETAKELAQrgARVYLACRDVEKGELVAKEIQTTTG------------- 2p.2
SDR7C1  G. gorilla        VVVTGANTGIGKETAKELAQrgARVYLACRDVEKGELVAKEIQTTTG------------- 2p.2
SDR7C1  M. musculus       AIVTGANTGIGKETAKDLAQrgARVYLACRDVDKGELAAREIQAVTG------------- 2p.2
SDR7C1  M. domestica      VIVTGANTGIGKETAKDLAQrgARVYIACRDLQKGELAASEIRAKTG------------- 2p.2
SDR7C1  P. formosa        VLITGANTGIGKETALDLAVrgARVIMACRDVEKGEEAAASIRAAYS------------- 2p.2
SDR7C2  H. sapiens        VVITGANTGIGKETARELASrgARVYIACRDVLKGESAASEIRVDTK------------- 2p.2
SDR7C2  P. troglodytes    VVITGANTGIGKETARELASrgARVYIACRDVLKGESAASEIRVDTK------------- 2p.2
SDR7C2  M. musculus       VVITGANTGIGKETARELARrgARVYIACRDVLKGESAASEIRADTK------------- 2p.2
SDR7C2  M. domestica      VVVTGANTGIGKETAKDLARrgARVYIACRNILKGESAASEIRAATK------------- 2p.2
SDR7C2  G. gallus         AVITGANTGIGKETARELARrgARVIVACRDIAKAEAAAREIRAETD------------- 2p.2
```

```
SDR7C2  A. carolinensis   AVITGANTGIGKETARELARrgARVIIACRNTEKGEAAAHEIQRETG------------- 2p.2
SDR7C2  D. rerio          ALVTGANSGIGKETALDLASrgARVILACRDLEKAEEAAAEIRTRVG------------- 2p.2
SDR7C3  H. sapiens        VIVTGANTGIGKQTALELARrgGNIILACRDMEKCEAAAKDIRGETL------------- 2p.2
SDR7C3  P. troglodytes    VIVTGANTGIGKQTALELARrgGNIILACRDMEKCEAAAKDIRGETL------------- 2p.2
SDR7C3  M. musculus       VIVTGANTGIGKQTALELAKrgGNVILACRDMEKCEVAAKDIRGETL------------- 2p.2
SDR7C3  X. tropicalis     VIVTGANTGIGKETALELAKrgGRIIMACRDMGKCENAARDIRGKTL------------- 2p.2
SDR7C3  O. latipes        VVITGANTGIGKETARELAKrgGRILMGCRDMEKCETAAKEIRGATL------------- 2p.2
SDR7C4  H. sapiens        VLITGANSGLGRATAAELLRLGARVIMGCRDRARAEEAAGQLRRELRQAAECGPEPGVSG
SDR7C4  P. troglodytes    VLITGANSGLGRATAAELLRLGARVIMGCRDRARAEEAAGQLRRELRQAAECGPEPGVSG
SDR7C4  M. musculus       VLITGANSGLGRATAAELLRLGARVIMGCRDRARAEEAAGQLRQELCQAGGAGPDG---T
SDR7C4  M. domestica      VIVTGANSGLGRATAAELLRQEARVILACRDRGRAEQTAAELRREQAQA---PPAAEGSP
SDR7C4  P. humilis        VIITGANSGLGRAAAAELLRMRARVIMGCRDRARAERAAREIRAEVGER--------ADG
SDR7C4  A. carolinensis   VIITGANSGIGRATAAELLRQHARVIMACRDPLRAEEAARELRAELGVCAR----GGGEC
SDR7C4  X. tropicalis     VIITGANCGIGKATAAELVKQEARVILACRDQGRAEEAAAELRREAGE------------
SDR7C4  D. rerio          VIVTGANCGIGKATAAELLKLQARVIMACRDRQRAEDAARDIQNQAG-----------TS
SDR7C5  H. sapiens        AVVtgANSGIGKMTALELARRGARVVLACRSQERGEAAAFDLRqeSG------------- 1p.2 2p.0
SDR7C5  P. abelii         AVVtgANSGIGKMTALELARRGARVVLACRSRERGEAAAFDLRqeSG------------- 1p.2 2p.0
SDR7C5  M. musculus       VVVtgANSGIGKMTALELARRGARVVLACRSRERGEAAAFDLRqeSG------------- 1p.2 2p.0
SDR7C5  M. domestica      AVVtgGNTGIGKMTALELAQRGARVVLACRSKEKGEAAVYDIRkeSG------------- 1p.2 2p.0
SDR7C5  H. leucocephalus  AIVtgGSSGIGAATALELARCGARVVLATRNAPRGEAAARRIRteTG------------- 1p.2 2p.0
SDR7C5  G. japonicus      VLItgGNTGIGKETALDLARRGARIIMACRNKARAEAAVYDIRreSG------------- 1p.2 2p.0
SDR7C5  X. tropicalis     VIVtgANVGIGKMTALDMAKRGARVILACRVKETGEAAAYDIRklSG------------- 1p.2 2p.0
SDR7C5  O. latipes        AIVtgSNTGIGKATAIELAKRGARVILACRSKQRGEAALEDVRrvTG------------- 1p.2 2p.0
SDR40C1 H. sapiens        FLVTGGNSGIGKATALEIAKrgGTVHLVCRDQAPAEDARGEIIRESG------------- 2p.2
SDR40C1 P. troglodytes    FLVTGGNSGIGKATALEIAKrgGTVHLVCRDQARAEDARDEIIRESS------------- 2p.2
SDR40C1 B. taurus         FMVTGGNSGIGKATAMEIAKrgGTVHLVCRDHSRAEGAKAEIIRESG------------- 2p.2
SDR40C1 G. gallus         FLITGSNSGIGKAAAKEIARrgGTVHLVCRNKERAEDAKGEIVTETG------------- 2p.2
SDR40C1 A. carolinensis   FMVTGANSGIGKATAKEIAKrgGIIHLVCRNKDRGEEAKKEITTETG------------- 2p.2
SDR40C1 X. tropicalis     YMITGANSGIGKAAALVIAKkgGTIHLVCRNKERAEEAQRELKANSG------------- 2p.2
SDR40C1 O. latipes        FMVTGANSGIGKAAAQEIAArgGTVHMVCRNKGRAEAAKEEIVERSK------------- 2p.2
                             **   * *   *              *     *

SDR7C1  H. sapiens        NQQVLVRKLDLSDTKSIRAFAKGFLaeEKHLHVLINNAGVMMCPYSKTADGFEMHIGVNH 3p.2
SDR7C1  G. gorilla        NQQVLVRKLDLSDTKSIRAFAKGFLaeEKHLHVLINNAGVMMCPYSKTADGFEMHIGVNH 3p.2
SDR7C1  M. musculus       NSQVFVRKLDLADTKSIRAFAKDFLaeEKHLHLLINNAGVMMCPYSKTADGFEMHIGVNH 3p.2
SDR7C1  M. domestica      NQQVLVRKLDLSDTKSIRAFAEAFLaeEKHLHILINNAGVMMCPYSKTADGFEMHMGINY 3p.2
SDR7C1  P. formosa        EAKVEVRELDLADTCSIRAFAQKFLreVNHLHILINNAGVMMCPYTKTIDGFEMHIGVNH 3p.2
SDR7C2  H. sapiens        NSQVLVRKLDLSDTKSIRAFAEGFLaeEKQLHILINNAGVMMCPYSKTADGFETHLGVNH 3p.2
SDR7C2  P. troglodytes    NSQVLVRKLDLSDTKSIRAFAEGFLaeEKQLHILINNAGVMMCPYSKTADGFETHLGVNH 3p.2
SDR7C2  M. musculus       NSQVLVRKLDLSDTKSIRAFAERFLaeEKKLHILINNAGVMMCPYSKTTDGFETHFGVNH 3p.2
SDR7C2  M. domestica      NQQVFVRKLDLSDTKSIRAFAEGFLaeEKQLHILINNAGVMMCPNSKTADGFETHLGVNH 3p.2
SDR7C2  G. gallus         NQEVIVKKLDLADTRSIREFANSFLaeEKELHILINNAGVMLCPYSKTADGFEMHLGVNH 3p.2
SDR7C2  A. carolinensis   NQQVIVKKLDLSDTKSIRTFAENLLkeEDKLHILINNAGVMFCPYSKTADGFEMQFGVNH 3p.2
SDR7C2  D. rerio          GAKVEVRELDLADCCSIRAFAQRFLreVDHLHILINNAGVMMCPYMKTADGFEMQIGVNH 3p.2
SDR7C3  H. sapiens        NHHVNARHLDLASLKSIREFAAKIIeeEERVDILINNAGVMRCPHWTTEDGFEMQFGVNH 3p.2
SDR7C3  P. troglodytes    NHHVNARHLDLASLKSIREFAAKIIeeEERVDILINNAGVMRCPHWTTEDGFEMQFGVNH 3p.2
SDR7C3  M. musculus       NPRVRAERLDLASLKSIREFARKVIkeEERVDILVNNAAVMRCPHWTTEDGFEMQFGVNY 3p.2
SDR7C3  X. tropicalis     NHNVFARHLDLASSKSIKEFAKTIIneEERVDVLINNAAVMRCPHWKTEDNFEMQFGVNH 3p.2
SDR7C3  O. latipes        NPHVYACHLDLASIKSIREFAERVNkeEKRVDVLINNAGVMRCPAWKTEDGFDMQFGVNH 3p.2
SDR7C4  H. sapiens        VGELIVRELDLASLRSVRAFCQEMLqeEPRLDVLINNAGIFQCPYMKTEDGFEMQFGVNH 1p.0
SDR7C4  P. troglodytes    VGELIVRELDLASLRSVRAFCQEMLqeEPRLDVLINNAGIFQCPYMKTEDGFEMQFGVNH 1p.0
SDR7C4  M. musculus       DGQLVVKELDLASLRSVRAFCQELLqeEPRLDVLINNAGVFHCPYTKTEDGFEMQFGVNH 1p.0
SDR7C4  M. domestica      AGELIVKELDLASLSSVRAFCREVLleEPRLDVLINNAGVFQCPYTKTEDGFEMQFGVNH 1p.0
SDR7C4  P. humilis        AGELVVRELDLASLRSVRAFCHRVLqeESRLDVLINNAGIFQCPYMKTEDGFEMQFGVNH 1p.0
SDR7C4  A. carolinensis   RGELLVRELDLASLRSVRTFCHQVLqeEPRLDVLINNAGIFQCPYTKTEDGFEMQFAVNH 1p.0
SDR7C4  X. tropicalis     RGEIVIKQLDLGSLQSVRRFCQEVLkeEPRLDVLINNAGVFQCPYTKTEDGFEMQFGVNH 1p.0
```

```
SDR7C4  D. rerio          QGEIVIKHLDLASLQSVRRFCEEVIreEPRIDVLINNAGLYQCPYSKTEEGFEMQLGVNH 1p.0
SDR7C5  H. sapiens        NNEVIFMALDLASLASVRAFATAFLSSEPRLDILIHNagISSCG--RTREAFNLLLRVNH 3p.2
SDR7C5  P. abelii         NNEVIFMALDLASLASVRAFATAFLSSEPRLDILIHNagISSCG--RTREAFNLLLRVNH 3p.2
SDR7C5  M. musculus       NNEVIFMALDLASLASVQAFATAFLSSEPRLDVLIHNagISSCG--RTRETFNLLLRVNH 3p.2
SDR7C5  M. domestica      NNEVIFMMLDLSSLTSVHSFATAFLSSEPRLDLLIHNagISSCG--KAKENFNLILRVNH 3p.2
SDR7C5  H. leucocephalus  NAEVLFMQLDLASLRSVRAFASAFLRQEPHLHLLINNagVSVGG--TTEDGFSLPFQVNH 3p.2
SDR7C5  G. japonicus      NNEVLFMSLDLADLSSVRAFVDAFLRSEPRLDILINNagVQSSG--KSADGFDLTFQVNH 3p.2
SDR7C5  X. tropicalis     NNQVVFMKLDLASLESVRSFCRAFLSSEPRLDILINNagLSGFG--KTAEGYNIVFGVNH 3p.2
SDR7C5  O. latipes        STQVLFMQLDLGSLKSVRNFAETFLKTESRLDILINNagLYMQG--RTEDGFGMMFGVNH 3p.2
SDR40C1 H. sapiens        NqnIFLHIVDLSDPKQIWKFVENFK-QEHKLHVliNNAGCMVNKRELTEDGLEKNFAANT 3p.0 4p.0
SDR40C1 P. troglodytes    NqnIFLHIVDLSDPKQIWKFVENFK-QEHKLHVliNNAGCMVNKRELTEDGLEKNFAANT 3p.0 4p.0
SDR40C1 B. taurus         NqnIFLHIVDLSLPKSVWKFVENFK-QEHTLNVliNNAGCMVNKRELTEDGLEKNFATNT 3p.0 4p.0
SDR40C1 G. gallus         NqnIFLHVVDISNPKEIWKFAEKFK-NEHKLNVliNNAGCMVNNRELTEDGLEKNFATNT 3p.0 4p.0
SDR40C1 A. carolinensis   NqkVFVHILDMSDPKGIWKFGEQFK-NEHRLNVliNNAGCMVNKRELTENGLEKNFATNT 3p.0 4p.0
SDR40C1 X. tropicalis     NedISVHLLDMSDPKQIWEFAEKFK-TEHKLNVliNNAGCMVNKRELTEDGLEKNFATNT 3p.0 4p.0
SDR40C1 O. latipes        NenVHVHIVDMSSAKQVWEFAQNFS-QNNKIHVliNNAGCMVNQRELTDEGLEKNFATNT 3p.0 4p.0
                                   *         *             *  **                    *

                                                                                    +
SDR7C1  H. sapiens        lgHFLLTHLLLEKLKESAPSRIVNVSSLAHHLGRIHFHNLQGE-------KFYNAGLAYC 4p.2
SDR7C1  G. gorilla        lgHFLLTHLLLEKLKESAPSRIVNVSSLAHHLGRIHFHNLQGE-------KFYNAGLAYC 4p.2
SDR7C1  M. musculus       lgHFLLTHLLLEKLKESAPSRIVNLSSLGHHLGRIHFHNLQGE-------KFYSAGLAYC 4p.2
SDR7C1  M. domestica      lgHFLLTHLLLERLKESAPSRVVNLSSLAFHLGRIHFYNLHGE-------KYYNRGLAYC 4p.2
SDR7C1  P. formosa        lgHFLLTYLLIGLLKRSAPARVVVVSSLAHNFGWIRFHDLHSQ-------GSYNSGLAYC 4p.2
SDR7C2  H. sapiens        lgHFLLTYLLLERLKVSAPARVVNVSSVAHHIGKIPFHDLQSE-------KRYSRGFAYC 4p.2
SDR7C2  P. troglodytes    lgHFLLTYLLLERLKVSAPARVVNVSSVAHHIGKIPFHDLQSE-------KRYSRGFAYC 4p.2
SDR7C2  M. musculus       lgHFLLTYLLLERLKESAPARVVNLSSIAHLIGKIRFHDLQGQ-------KRYCSAFAYG 4p.2
SDR7C2  M. domestica      lgHFLLTHLLLERLKESAPSRVVNVSSVGHHLGRIFFQDLQGE-------KYYNRSYAYC 4p.2
SDR7C2  G. gallus         lgHFLLTFLLLERLKQSAPSRIVNVSSLAHHGGRIRFHDLNGE-------KSYNRGLAYC 4p.2
SDR7C2  A. carolinensis   lgHFLLTFLLLDRLKESAPARIVNVSSLAHILGKIYFQDLQGE-------KCYSAQFAYF 4p.2
SDR7C2  D. rerio          lgHYLLTYLLIGLLKRSAPSRIVVVSSLAHNFGWIRFHDLHSQ-------GSYNSGLAYC 4p.2
SDR7C3  H. sapiens        lgHFLLTNLLLDKLKASAPSRIINLSSLAHVAGHIDFDDLNWQT------RKYNTKAAYC 4p.2
SDR7C3  P. troglodytes    lgHFLLTNLLLDKLKASAPSRIINLSSLAHVAGHIDFDDLNWQT------RKYNTKAAYC 4p.2
SDR7C3  M. musculus       lgHFLLTNLLLDKLKASAPSRIINLSSLAHVAGHIDFEDLNWQM------KKYDTKAAYC 4p.2
SDR7C3  X. tropicalis     lgHFLLTNLLLEKMKRSENSRIINVSSLAHIAGDIDFDDLNWEK------KKYNTKAAYC 4p.2
SDR7C3  O. latipes        lgHFLLTNLLLEKLKEPAPSRVINLSSLAHIIGNIDFEDLNWEK------KTFDTKQAYC 4p.2
SDR7C4  H. sapiens        LGHFLLTNLLLGLLKSSAPSRIVVVSSKLYKYGDINFDDLNSE-------QSYNKSFCYS
SDR7C4  P. troglodytes    LGHFLLTNLLLGLLKSSAPSRIVVVSSKLYKYGDINFDDLNSE-------QSYNKSFCYS
SDR7C4  M. musculus       LGHFLLTNLLLGLLKSSAPSRIVVVSSKLYKYGEINFEDLNSE-------QSYNKSFCYS
SDR7C4  M. domestica      LGHFLLTNLLLDRLKDSAPSRIVVVSSKLYKYGEINFEDLNSE-------LNYNKSFCYS
SDR7C4  P. humilis        LGHFLLTNLLLGLLKNSAPSRIVVVSSKLYKYGEINFEDLNSE-------ISYNKSFCYS
SDR7C4  A. carolinensis   LGHFLLTNLLLGLLKSSAPSRIVVVSSKLYKYGEINFDDLNSE-------LSYNKSFAYS
SDR7C4  X. tropicalis     LGHFLLTHHLLGLLKSSAPSRIVVVSSKLYKYGEINFDDLNSE-------KSYSRSFGYS
SDR7C4  D. rerio          LGHFLLTNLLLDLLKQSSPSRVVVVSSKLYKYGSINFEDLNSE-------QSYNKSFCYS
SDR7C5  H. sapiens        IGPFLLTHLLLPCLKACAPSRVVVVASAAHCRGRLDFKRLDRPV----V-GWRQELRAYA
SDR7C5  P. abelii         IGPFLLTHLLLPCLKACAPSRVVVVASAAHRRGRLDFKRLDRPV----V-GWRQELRAYA
SDR7C5  M. musculus       VGPFLLTHLLLPRLRSCAPSRVVIVSSAAHRRGRLDFTRLDCPV----V-GWQQELRAYA
SDR7C5  M. domestica      VGPFLLTHLLLPRLKANAPSRVVVMASAAHRRGRLDFSRLDCPV----W-GWQQELRAYA
SDR7C5  H. leucocephalus  LGHFLLTQLLLERLQSCAPSRVVIVASRAHCAGRLRPNTLGRPP----S-GLFSAFQDYC
SDR7C5  G. japonicus      LSHFLMTHLLLDRLKCCAPSRVVVVASRAHRIGKINFQNIHKPV----LGGLVKYFQAYC
SDR7C5  X. tropicalis     LGHFLLTSLLLDRLKQSTPSRIVVLASYAHEWGKIDFNKISVPS----E-HVKDTLQSYC
SDR7C5  O. latipes        LGHFLLTNLLLDRLKECGPSRIVNVSSSAHNVGNVNFDCLNTHKDLGVATSTRDALQMYC
SDR40C1 H. sapiens        lgVYILTTGLIPVLEKEHDPRviTVSSGGMLVQKLNTNDLQSER------TPFDGTMVYA 5p.2 6p.0
SDR40C1 P. troglodytes    lgVYILTTGLIPVLEKEHDPRviTVSSGGMLAQKLSTNDLQSER------TPFDGTMVYA 5p.2 6p.0
SDR40C1 B. taurus         lgVYVLTTALIPVLEKEHDPRviTVSSGGMLVQKLNTDDPQSER------TAFDGTMVYA 5p.2 6p.0
SDR40C1 G. gallus         lgTYVLTTALLPLLEKEADARvvTVSSGGMLVQKLNISDLQSGS------ETFDGTMVYA 5p.2 6p.0
```

```
SDR40C1 A. carolinensis   lgPYILTTVLLPLLEKEDDPRviTVSSGGMLVQKLNVSDLQTEN------TTFDGTMVYA 5p.2  6p.0
SDR40C1 X. tropicalis     lgTYILTTALLPSLEKEEDPRviTVSSGGMLVQKLNVSDLQFET------GTFDGTMAYA 5p.2  6p.0
SDR40C1 O. latipes        lgTYILTTALIPSLKQVEDPRviTVSSGGMLTQKLNVDDLQFEK------GAFDGTMAYA 5p.2  6p.0
                                *  *          *     *                               *


                            +
SDR7C1  H. sapiens        HSKLANILFTQELARRLkgSGVTTYSVHPGTVQSELV-RHSSFM------RWMWWLFSFF 5p.2
SDR7C1  G. gorilla        HSKLANILFTQELARRLkgSGVTTYSVHPGTVQSELV-RHSSFM------RWMWWLFSFF 5p.2
SDR7C1  M. musculus       HSKLANILFTKELAKRLkgSGVTTYSVHPGTVHSELT-RYSSIM------RWLWQLFFVF 5p.2
SDR7C1  M. domestica      HSKLANVLFTQELSRRLkgTGVTTYSVHPGTVDSDLF-RHSLFL------KLLVKLFSSF 5p.2
SDR7C1  P. formosa        QSKLANVLFARELARRLkgTNVMVNSVHPGTVNSDLT-RHSTLM------MILFTVFSVF 5p.2
SDR7C2  H. sapiens        HSKLANVLFTRELAKRLqgTGVTTYAVHPGVVRSELV-RHSSLL------CLLWRLFSPF 5p.2
SDR7C2  P. troglodytes    HSKLANVLFTRELAKRLqgTGVTTYAVHPGVVRSELV-RHSSLL------CLLWRLFSPF 5p.2
SDR7C2  M. musculus       HSKLANLLFTRELAKRLqgTGVTAYAVHPGVVLSEIT-RNSYLL------CLLWRLFSPF 5p.2
SDR7C2  M. domestica      NSKLANVLFTRELAYRLkgTGVTTYAVHPGLVQSELV-RHSFLM------CLLWRLLTPI 5p.2
SDR7C2  G. gallus         HSKLANVLFTRELARRLqgTKVTANSLHPGSVHSELV-RHSFVM------TWLWRIFSFF 5p.2
SDR7C2  A. carolinensis   QSKLANILFTRELAGRLqgTGVTVNALHPGAVLSELG-RHSYVA------KFLQRVFNFM 5p.2
SDR7C2  D. rerio          QSKLANVLFTRELARRLqgSNVTVNSVHPGTVRSELV-RHSTLM------SLLFAFFSMF 5p.2
SDR7C3  H. sapiens        QSKLAIVLFTKELSRRLqgSGVTVNALHPGVARTELG-RHTGIHGSTFSSTTlgPIFWLL 5p.2  6p.2
SDR7C3  P. troglodytes    QSKLAIVLFTKELSRRLqgSGVTVNALHPGVARTELG-RHTGIHGSTFSSTTlgPIFWLL 5p.2  6p.2
SDR7C3  M. musculus       QSKLAVVLFTKELSHRLqgSGVTVNALHPGVARTELG-RHTGMHNSAFSGFMlgPFFWLL 5p.2  6p.2
SDR7C3  X. tropicalis     QSKLANVLFTNELAKRLqgTKLTANSLHPGVADTELG-RHTGMHQSAFSSTIlaPLFWFL 5p.2  6p.2
SDR7C3  O. latipes        QSKLANVLFTRELAKRLqgTGVTVNAVHPGVVATELG-RHTGLHQSQFSSFMlgPFFSLL 5p.2  6p.2
SDR7C4  H. sapiens        RSKLANILFTRELARRLEGTNVTVNVLHPGIVRTNLG-RHIHIP--LLVKPLFNLVSWAF
SDR7C4  P. troglodytes    RSKLANILFTRELARRLEGTNVTVNVLHPGIVRTNLG-RHIHIP--LLVKPLFNLVSWAF
SDR7C4  M. musculus       RSKLANILFTRELARRLEGTNVTVNVLHPGIVRTNLG-RHIHIP--LLARPLFNLVSWAF
SDR7C4  M. domestica      QSKLANILFTRELARRLEGTNVTVNVLHPGIVRTNLG-RHINIP--LLVKPLFNLVSWAF
SDR7C4  P. humilis        RSKLANILFARELARRLEGTGVTVNSLHPGIVRTNLG-RHVNIP--LLAKPLFNLVSWAF
SDR7C4  A. carolinensis   RSKLANILFTRELSHRLEGTGVSVNVLHPGVVRTNLG-RYVHIP--LLARPLFNLVSWAF
SDR7C4  X. tropicalis     RSKLANILFTRELASRLEGTGVTVNALHPGIVRTNLG-RHINIP--ILIKPLFNVVSWAF
SDR7C4  D. rerio          QSKLANLLFTRELARRLDGTEVTVNALTPGIVRTRLG-RHVNIP--LLIKPLFWLVSWLF
SDR7C5  H. sapiens        DTKLANVLFARELANQLEATGVTCYAAHpgPVNSELFLRHVPG----WLRPLLRPLAWLV 4p.2
SDR7C5  P. abelii         DTKLANVLFARELANQLEGTGVTCYAAHpgPVNSELFLRHVPG----WLRPLLRPLAWLV 4p.2
SDR7C5  M. musculus       DSKLANVLFARELATQLEGTGVTCYAAHpgPVNSELFLRHLPG----WLRPILRPLAWLV 4p.2
SDR7C5  M. domestica      DSKLANVLFTRELATQLEGSGVTCYAAHpgPVNSELFLRHVPG----WLHLLLSPLAWLV 4p.2
SDR7C5  H. leucocephalus  DSKLANVLHARELATHLQGTQVTCYAVHpgFVNTELF-RHTPL----WLKPLFLPLAWLF 4p.2
SDR7C5  G. japonicus      NSKLANILYTRELANRLEGTSVTCYALHpgTVNTELF-RHASI----WLKLIVGPLCWLF 4p.2
SDR7C5  X. tropicalis     DSKLCNVLFARELANRLQGTSVTCYSVHpgTVHTNLA-RSLPS----WIKVLIEPVSWLF 4p.2
SDR7C5  O. latipes        DSKLCNVLFTHELAKRLEGTKVTCYSLHpgAISTELK-RNAGS----ILQFSLTFASVFF 4p.2
SDR40C1 H. sapiens        QNkrQQVVLTERWAQGHP--AIHFSSMHPGWADTpgV-RQA--------MPGFHARFGDR 7p.0  8p.2
SDR40C1 P. troglodytes    QNkrQQVVLTERWAQGHL--AIHFSSMHPGWADTpgV-RQA--------MPGFHARFRDR 7p.0  8p.2
SDR40C1 B. taurus        QNkrQQVVLTERWARAHP--AIHFSCMHPGWVDTpgV-RLS--------MPGFHARLGAR 7p.0  8p.2
SDR40C1 G. gallus         QNkrQQVVLTEQWAKTHR--SIHFSVMHPGWADTpaV-RSS--------MPDFYQKMKNS 7p.0  8p.2
SDR40C1 A. carolinensis   QNkrQQVVMTEQWAKAHP--SIHFSSMHPGWANTpaV-QSS--------MPDFYEKMKNK 7p.0  8p.2
SDR40C1 O. latipes        QNkrQQVVLTDKWAAQHK--DIHFSSMHPGWADTpaV-QSS--------MPSFHAKMQSK 7p.0  8p.2
SDR40C1 X. tropicalis     QNkrQQVILTEQWAKANP--NVHFSVMHPGWADTpaV-RSS--------MPDFYEKMKNR 7p.0  8p.2
                            *                         **

SDR7C1  H. sapiens        IKTPQQGAQTSLHCALTEG-LEILSGNHFsdCHVAWV-SAQARNETI---ARRLWDVSCD 6p.1
SDR7C1  G. gorilla        IKTPQQGAQTSLHCALTEG-LEILSGNHFsdCHVAWV-SAQARNETI---ARRLWDVSCD 6p.1
SDR7C1  M. musculus       IKTPQEGAQTSLYCALTEG-LESLSGSHFsdCQLAWV-SYQGRNEII---ARRLWDVSCD 6p.1
SDR7C1  P. formosa        LKTPLEGAQTSVYCAVAEE-LHSVSGKHFsdCAPAFV-APQGRSEET---ASKLWDVSCE 6p.1
SDR7C1  M. domestica      IKTPQEGAQTSLYCALTEG-LEPLSGNHFsdCRPAWI-SSRGRNMTT---AMRLWDASCN 6p.1
SDR7C2  H. sapiens        VKTAREGAQTSLHCALAEG-LEPLSGKYFsdCKRTWV-SPRARNNKT---AERLWNVSCE 6p.1
SDR7C2  P. troglodytes    VKTAREGAQTSLHCALAEG-LEPLSGKYFsdCKRTWV-SPRARNNKT---AERLWNVSCE 6p.1
SDR7C2  M. musculus       FKSTSQGAQTSLHCALAED-LEPLSGKYFsdCKRMWV-SSRARNKKT---AERLWNVSCE 6p.1
```

```
SDR7C2  M. domestica        MKTTSQGAQTSLHCALAEG-IESQSGRYFsdCRKAWV-SPKGRNNKT---ARRLWDVSCE 6p.1
SDR7C2  G. gallus           LKTPWEGAQTSVYCAVAEE-LESVTGQYFsdCQPAYV-SPWGRDDET---AKKLWNVSCE 6p.1
SDR7C2  A. carolinensis     WKTVEEGAQTTVHCAVAEE-LESVTGEYFsdCKPAWV-APQGLDENT---AKKLWKVSCE 6p.1
SDR7C2  D. rerio            LKSPKEGAQTSIYCAVAEE-LQSISGKHFsdCAPAFV-APQGRSEET---ARKLWDVSCE 6p.1
SDR7C3  H. sapiens          VKSPELAAQPSTYLAVAEE-LADVSGKYFDGLKQKAP-APEAEDEEV---ARRLWAESAR
SDR7C3  P. troglodytes      VKSPELAAQPSTYLAVAEE-LADVSGKYFDGLKQKAP-APEAEDEEV---AQRLWAESAR
SDR7C3  M. musculus         FKSPQLAAQPSTYLAVAEE-LENVSGKYFDGLREKAP-SPEAEDEEV---ARRLWTESAR
SDR7C3  X. tropicalis       VKSPKQAAQPSVYLAVAEN-LQGVSGKYFNALKEKEP-APQALDEES---ARKLWEESAK
SDR7C3  O. latipes          VKSPALGAQPSVFLAVSEE-MEGVTGRYYDVMTEKEP-AAQALDDEV---ACRLWEVSSR
SDR7C4  H. sapiens          FKTPVEGAQTSIYLASSPE-VEGVSGRYFGDCKEEEL-LPKAMDESV---ARKLWDISEV
SDR7C4  P. troglodytes      FKTPVEGAQTSIYLASSPE-VEGVSGRYFGDCKEEEL-LPKAMDESV---ARKLWDISEV
SDR7C4  M. musculus         FKTPLEGAQTSIYLACSPD-VEGVSGRYFGDCKEEEL-LPKAMDESV---ARKLWDISEV
SDR7C4  M. domestica        FKTPEEGAQTSIYLASSAE-VEGVTGKYFGDCKQEEL-LPKAMDDSV---ARKLWDISEV
SDR7C4  P. humilis          FKTPLEGAQTSIYLASSPD-VEGVSGKYFGDCKEEEL-LPKAMDDLV---ARKLWDISEV
SDR7C4  A. carolinensis     FKSPLEGAQTSVYLASSPE-VEGVSGKYFGDCKEEQL-LPKAMDDLV---ARKLWDISEV
SDR7C4  X. tropicalis       FKSPEEGAQTSIYLASSPE-VEGVSGSYFGNSKEEEL-LPKAMDDLV---ARKLWDISEV
SDR7C4  D. rerio            FKSPLEGAQTPLYLACSPE-VEGVSGKCFANCEEEQL-LSKATDDHA---AKRLWDLSES
SDR7C5  H. sapiens          LRAPRGGAQTPLYCALQEG-IEPLSGRYFANCHVEEV-PPAARDDRA---AHRLWEASKR
SDR7C5  P. abelii           LRAPRGGAQTPLYCALQEG-IEPLSGRYFANCHVEEV-PPAARDDRA---AHRLWEASKR
SDR7C5  M. musculus         LRAPQGGAQTPLYCALQEG-IEPLSGRYFANCHVEEV-SPAARDDQA---AQRLWKATKK
SDR7C5  M. domestica        LRTPRGGAQTPLHCALQEG-IEPLSGRYFANCHVEEV-PTTARDDRA---ARRLWEASEK
SDR7C5  H. leucocephalus    FLDAAEGAQTSLHCATQEG-LERFSGRYFADCRLQEP-WPPARDDRL---ARALWEASER
SDR7C5  G. japonicus        LRDPVNGAQTTIYCATQEG-IERFSGHYFANCKLQEP-YPQARDDAI---AKKLWEFSEK
SDR7C5  X. tropicalis       LRTPMNGAQTSIYCAVQEG-IEMYSGRYFDNCQVRQV-KPHARDDAV---AKKLWEVSER
SDR7C5  O. latipes          FKDAEQGSQTTLHCALQEG-IEHLSGRYFSNCTVRDV-FARAKDDAT---AKKLWELSER
SDR40C1 H. sapiens          LRSEAQGADTMLWLALSSAAAAQPSGRFFqdRKPVSTHLPLATASSSPAEEEKLIEILEQ 9p.2
SDR40C1 P. troglodytes      LRSEAQGADTVLWLALSSAAAAQPSGRFFqdRKPVSTHLPLARTSSSPAEEEKLIEILEQ 9p.2
SDR40C1 B. taurus           LRSEAQGADTVLWLALAPAATAQPSGCFFqdRKPAPTHLPLARTSSSPAEEEKLIEILEE 9p.2
SDR40C1 G. gallus           LRTEAQGADTVVWLAVSSEAAKLPSGLFFqdRQSVPKHLPLARTHSPPGDEEKLMEVLEE 9p.2
SDR40C1 A. carolinensis     LRTEAQGADTVVWLAVSSAARKQASGLFFqdREPVATHLPLAWTKSSPGDDEKLMQVLEE 9p.2
SDR40C1 X. tropicalis       LRTEEQGADTVVWLTLSPSAKKHPSGLFFqdRKPVSTHLPFALTHSSPGDEEKLMESLKE 9p.2
SDR40C1 O. latipes          LRTEAMGADTVVWLAVSAAAIKQPSGLFFqdRKPVPTHLPLAWSRPSAQDEDQLLAALQE 9p.2
                                                     *                           *

SDR7C1  H. sapiens          LLGLPID-------------------------------------------------------------
SDR7C1  G. gorilla          LLGLPID-------------------------------------------------------------
SDR7C1  M. musculus         LLGLPVDW------------------------------------------------------------
SDR7C1  M. domestica        MLGIQQD-------------------------------------------------------------
SDR7C1  P. formosa          LLGIDWD-------------------------------------------------------------
SDR7C2  H. sapiens          LLGIRWE-------------------------------------------------------------
SDR7C2  P. troglodytes      LLGIRWE-------------------------------------------------------------
SDR7C2  M. musculus         LLGIQWE-------------------------------------------------------------
SDR7C2  M. domestica        LLGI----------------------------------------------------------------
SDR7C2  G. gallus           LLGIQWD-------------------------------------------------------------
SDR7C2  A. carolinensis     LLGIQWQ-------------------------------------------------------------
SDR7C2  D. rerio            LLGIEWD-------------------------------------------------------------
SDR7C3  H. sapiens          LVGLEAPSVREQPLPR----------------------------------------------------
SDR7C3  P. troglodytes      LVGLEAPSVREQPLPR----------------------------------------------------
SDR7C3  M. musculus         LVGLAMAHGSPGRGHAIPR-------------------------------------------------
SDR7C3  X. tropicalis       LVHLEEALTECKAL------------------------------------------------------
SDR7C3  O. latipes          LVGLQENGQHGSLKKEGVSEP-----------------------------------------------
SDR7C4  H. sapiens          MVGLLK--------------------------------------------------------------
SDR7C4  P. troglodytes      MVGLLK--------------------------------------------------------------
SDR7C4  M. musculus         MVGILK--------------------------------------------------------------
SDR7C4  M. domestica        MVGILE--------------------------------------------------------------
SDR7C4  P. humilis          MVGLLK--------------------------------------------------------------
```

```
SDR7C4  A. carolinensis   MVGLLK--------------------------------------------------------------
SDR7C4  X. tropicalis     MVGLIK--------------------------------------------------------------
SDR7C4  D. rerio          MVGLRR--------------------------------------------------------------
SDR7C5  H. sapiens        LAGLGPGEDAEPDEDPQSEDSEAPSSLSTPHPEEPTVSQPYPSPQSSPDLSKMTHRIQAKVEPEIQLS
SDR7C5  P. abelii         LAGLGPGEDAEPDEDPQSEDSEAPFSLSTPHPEEPTVSQPYPSPQSSPDLSKMTHRIQAKVEPETQLS
SDR7C5  M. musculus       LAGLAPGDDDDDPDEEPEPEDPRAPSSQSAPSPEKTTVSGPSHSYQGSQDLSKLTQRRIQVKDEPTP-
SDR7C5  M. domestica      LAGLSKGGSQNTNPKPGSPRNPLAQIATSPALSRFRNLSFPLLSEGQRTVKDEPTP------------
SDR7C5  H. leucocephalus  LVGLGGPGENPSLAPAAVIQ------------------------------------------------
SDR7C5  G. japonicus      LLGLAE--------------------------------------------------------------
SDR7C5  X. tropicalis     MTGLAS--------------------------------------------------------------
SDR7C5  O. latipes        MSGLA---------------------------------------------------------------
SDR40C1 H. sapiens        LAQTFK--------------------------------------------------------------
SDR40C1 P. troglodytes    LAQTFK--------------------------------------------------------------
SDR40C1 B. taurus         LARRFK--------------------------------------------------------------
SDR40C1 G. gallus         FSQKFKSTSPGSPQCH----------------------------------------------------
SDR40C1 A. carolinensis   LSQQFKPQ------------------------------------------------------------
SDR40C1 X. tropicalis     LSQKFAPASCKL--------------------------------------------------------
SDR40C1 O. latipes        LALKFKP-------------------------------------------------------------
```

Figure S13. Alignment of the vertebrate SDR7C family and of SDR40C1 variants. × and + symbols mark the structure consensus and the catalysis consensus respectively; the couples of amino acid symbols in red lowercase mark the splicing-site positions. Splicing sites are progressively numbered and the phase (p.) type is indicated after the splicing-site number. * symbol marks the position of identical amino acid residues of the aligned SDR protein sequences.

```
SDR9C1 H. sapiens              ---------------------M-L-----ATRLSRPLSRLPG----KTLSACDRE-----
SDR9C1 P. paniscus             ---------------------M-L-----ATRLSRPLSRLPG----KTLSACDRE-----
SDR9C1 M. musculus             ---------------------M-L-----AARLSRPLSQLPG----KALSVRDRE-----
SDR9C1 M. domestica            ---------------------M-L-----TARFSRSLVHLSR----NFLSFQ--------
SDR9C1 C. livia                ---------------------M-L-----ATKLSRPLLNFAV----KALNFKDPG-----
SDR9C1 C. picta                --------------------MM-L-----ATRISRPLSLFSV----KTLNSRDIG-----
SDR9C1 X. tropicalis           ---------------------------------------MAS----CHLPVRACA-----
SDR9C1 D. rerio                MAS----------------LPM-V-RVALLVAFSVFLTLVLGFGLPSVLNWIARCCGFPE
SDR9C2 H. sapiens              -MSTFFSDTAWICLA----VPTVLCGTVFCK-YKKS-S------G-QLWSWMVCL-AGLC
SDR9C2 P. troglodytes          -MSTFFSDTAWICLA----VPTVLCGTVFCK-YKKS-S------G-QLWSWMVCL-AGLC
SDR9C2 M. musculus             -MSPFASESAWLCLA----AAAVLGGTLLCG-CRSG-R--------QLRSQAVCLAGLWG
SDR9C2 M. domestica            -----MNPFSWESRLLPLGVTVMLGAIALYK-FKKS-QSRTGS-SPVVLCHSVLL-CLWG
SDR9C2 G. gallus               MDALPSSPWGWLCVA----IPVLFGL---CL-AKRS-C------RAHMLLRSVLP-ALFG
SDR9C2 A. mississippiensis     ------------------------------------------------------------
SDR9C2 X. tropicalis           -MAHLDS------RLLYLAVCVFFSGSALHK-IMKN-KVKIEN-ASV---SGLLSLLILG
SDR9C2 D. rerio                ---------------------MYTSGIVLYVSFIRSNKRPIAF-TSKWRSRGAV------
SDR9C3 H. sapiens              -----MERWPWPSGG----AWLLVAARALLQ-LLRS---DLRL-GRPLLAALAL-LAALD
SDR9C3 P. troglodytes          -----MERWPWPSGG----AWLLVAARALLQ-LLRS---DLRL-GRPLLAALAL-LAALD
SDR9C3 M. musculus             -----MERWPWPSGG----AWLLVAARALLQ-LLRS---DLRL-GRPLLAALAL-LAALD
SDR9C3 C. picta                -----MERWV--YGS----LWLVFAGSLLLR-LSRS---QLLL-TRALL-HLGL-LGLLQ
SDR9C3 X. tropicalis           -MELAACPSLWAYGA----VWLSFLLLGFLR-FSNS---DMVL-SPALLLYVVV-LVLIE
SDR9C3 D. rerio                --MEDFAVSFWIYIG----VMSIFVGGAVKK-FLAF---NIGA-MPSVVVWLGA-TLLVE
SDR9C4 H. sapiens              ------------------------------------------------------------
SDR9C4 P. troglodytes          ------------------------------------------------------------
SDR9C4 M. musculus             ------------------------------------------------------------
SDR9C4 M. domestica            ------------------------------------------------------------
SDR9C4 G. gallus               ------------------------------------------------------------
SDR9C4 C. mydas                ------------------------------------------------------------
SDR9C4 X. tropicalis           ------------------------------------------------------------
SDR9C4 D. rerio                ------------------------------------------------------------
SDR9C5 H. sapiens              ------------------------------------------------------------
SDR9C5 P. troglodytes          ------------------------------------------------------------
SDR9C5 M. musculus             ------------------------------------------------------------
SDR9C5 S. harrisii             ------------------------------------------------------------
SDR9C5 G. gallus               ------------------------------------------------------------
SDR9C5 A. carolinensis         ------------------------------------------------------------
SDR9C5 X. tropicalis           ------------------------------------------------------------
SDR9C5 D. rerio                ---------------------------------------------------------MYE
SDR9C6 H. sapiens              ------------------------------------------------------------
SDR9C6 P. paniscus             ------------------------------------------------------------
SDR9C6 M. musculus             ------------------------------------------------------------
SDR9C6 S. harrisii             ------------------------------------------------------------
SDR9C6 X. tropicalis           ------------------------------------------------------------
SDR9C6 A. carolinensis         ------------------------------------------------------------
SDR9C7 H. sapiens              ------------------------------------------------------------
SDR9C7 P. troglodytes          ------------------------------------------------------------
SDR9C7 M. musculus             ------------------------------------------------------------
SDR9C7 S. harrisii             ------------------------------------------------------------
SDR9C7 X. tropicalis           ------------------------------------------------------------
SDR9C8 H. sapiens              ------------------------------------------------------------
SDR9C8 P. troglodytes          ------------------------------------------------------------
SDR9C8 M. musculus             ------------------------------------------------------------
SDR9C8 P. humilis              ------------------------------------------------------------
SDR9C8 A. carolinensis         ------------------------------------------------------------
SDR9C8 M. domestica            ------------------------------------------------------------
SDR9C8 X. tropicalis           ------------------------------------------------------------
```

```
SDR9C8 P. reticulata        ------------------------------------------------------------

                                                                        ×   × ×
SDR9C1 H. sapiens           NGArrPLLLGSTSFI----PI---GRRTYASAAEpvGSKAVLVTGCDSGFGFSLAKHLHS  1p.1 2p.0
SDR9C1 P. paniscus          NGArrPLLLGSTSFI----PI---GRRTYASAAEpvGSKAVLVTGCDSGFGFSLAKHLHS  1p.1 2p.0
SDR9C1 M. musculus          NGTrhTLLFYPASFS----PD---TRRTYASQADaaSGKAILITGCDSGFGFSLAKHLHS  1p.1 2p.0
SDR9C1 M. domestica         NIGrpGQELFDPQAT----PA---GQRSSSTQVTeiGNRAVLITGCDSGFGFTTAKDLHE  1p.1 2p.0
SDR9C1 C. livia             NGFrpAERFCFPLLS----PH---GSRSYASEVDqiGSKAVLITGCDSGFGFALAKHLHD  1p.1 2p.0
SDR9C1 C. picta             NGLrpVQGFCFPFLT----PS---GRRSYASEIDqiGSKAVLITGCDSGFGFSLAKHLHS  1p.1 2p.0
SDR9C1 X. tropicalis        NHFrfMPKWSFPLQS----AV---GKRPFASHTAed-SKAVLVTGCDSGFGFSLAKHLHN  1p.1 2p.0
SDR9C1 D. rerio             ASVTECIVFVYALFV---LYV---AVPRLPRGTVevEGKALLITGCDTGFGPALAKHFHK       1p.0
SDR9C2 H. sapiens           AVCLLILSPFWGLILFSVSCFLMY-TYLSGQELLPVDQKAVLVtgGDCGLGHALCKYLDE  1p.2
SDR9C2 P. troglodytes       AVCLLILSPFWGLVLFSVSCFLMY-TYLSGQELLPVDQKAVLVtgGDCGLGHALCKYLDE  1p.2
SDR9C2 M. musculus          GACLLSLSLLCTLFLLSVACFLLLYMSSSDQDLLPVDQKAVLVtgADSGFGHGLAKHLDK  1p.2
SDR9C2 M. domestica         IYCFSSFSIFWGWTLFSLACCISL-SYSTSQEMLPLDKRAVLItgGDSGIGHALSKYLDE  1p.2
SDR9C2 G. gallus            LLCVAMLGTCWGLIVFCSTWLSCS-AYL-DAGPLPVGDKAVLItgSDTGIGHALAKYLDN  1p.2
SDR9C2 A. mississippiensis  --------------------------------MLPVDNKAVLItgSDSGIGHALAKHLDN  1p.2
SDR9C2 X. tropicalis        IFCFFVLSDTAGLTLLTLACTIYYS-IPVRDMLSAEGKSVLItgCDSGFGHALAKHLDK  1p.2
SDR9C2 D. rerio             --AVELSIERRPLVILSSLTTLYF------RKIF----TLAGVfsCDSGFGHELAQVLDR  1p.2
SDR9C3 H. sapiens           WLCQRLLPPPAALAVLAAAGWIAL-SRLARPQRLPVATRAVLItgCDSGFGKETAKKLDS  1p.2
SDR9C3 P. troglodytes       WLCQRLLPPPAALAVLAAAGWIAL-SRLARPQRLPVATRAVLItgCDSGFGKETAKKLDA  1p.2
SDR9C3 M. musculus          WLCQRLLPPPAALVVLAGAGWIAL-SRLARPPRLPVATRAVLItgCDTGFGKETAKKLDA  1p.2
SDR9C3 C. picta             YLCQACLPLPLGA-ALTAAGCLAL-GRGAQGRRLPVVGKAVFItgCDSGFGKQAACHLDS  1p.2
SDR9C3 X. tropicalis        WLCHLYLPIALGILFLSSACWYVL-GIVSPKRTLPVEGKVVFItgCDSGFGNVAAHKLDS  1p.2
SDR9C3 D. rerio             RLCALCMPAVLARLVLCVCCWLYF-TWATPKPSLPVEDKAVFItgCDSGFGNATAKKLDA  1p.2
SDR9C4 H. sapiens           -------MLFWVLGLLILCGFL--WTRKGKLKIEDITDKYIFITGCDSGFGNLAARTFDK
SDR9C4 P. troglodytes       -------MLFWVLGLLILCGFL--WTRKGKLKIEDITDKYIFITGCDSGFGNLAARTFDK
SDR9C4 M. musculus          -------MLFWLLALLFLCAFL--WNYKGQLKIADIADKYVFITGCDTGFGNLAARTFDK
SDR9C4 M. domestica         -------MILWMLAFLIICCFL--WNYKGHLRIADINNKYIFITGCDTGFGNLAAKTFDK
SDR9C4 G. gallus            -------MFFYILLFLSISSLWWYWKTRDGGKVANLNGKYIFITGCDTGFGNMAAKTFDK
SDR9C4 C. mydas             -------MFFHILIFLAIFYLWWRWRAQDGQKIRDLTDKYIFITGCDSGFGNLAARTFDK
SDR9C4 X. tropicalis        -------MLLCFLIGVAILYIWW--RVRDGLKINNITEKYILITGCDTGFGNHAAKTFDK
SDR9C4 D. rerio             -------MYLYI-AGLVVLFYVY-RWFRELGRVSNKSEKFVYITGCDTGFGNLLARHLDT
SDR9C5 H. sapiens           -------MWLPL-LLGALLWAVL-WLLRDRQSLPA-SNAFVFITGCDSGFGRLLALQLDQ
SDR9C5 P. troglodytes       -------MWLPL-LLGALLWAVL-WLLRDRQSLPA-SDAFVFITGCDSGFGRLLALQLDQ
SDR9C5 M. musculus          -------MWLPL-LLGALLWAVL-WLLRDRQSLPA-SDAFIFITGCDSGFGRLLALQLDQ
SDR9C5 S. harrisii          -------MWLLL-LLGTLLWGVL-WFLRDRLVLPP-SDAFVFITGCDSGFGRHLALRLDR
SDR9C5 G. gallus            -------MWLYL-LLVVLAWALG-WLVRDRQTLPSVKDKHVFITGCDSGFGNLLARRLAQ
SDR9C5 A. carolinensis      -------MWCCV-LLFSVLWTVV-WFFRDRQMLSSFKDKYVFVTGCDTGFGNLLAKRLDK
SDR9C5 X. tropicalis        -------MWCYV-LLFAMVWAIG-WLLRDRQKISRFSNKHVFITGCDTGFGNLLAKRLSR
SDR9C5 D. rerio             FLsnSCPSTYAL-GTFAILWVLV-WFYRDNLKITRVSEKHVFVTGCDSGFGNLLCKRLDK  1p.1
SDR9C6 H. sapiens           -------MWLYL-AAFVGLYYLL-HWYRERQVVSHLQDKYVFITGCDSGFGNLLARQLDA
SDR9C6 P. paniscus          -------MWLYL-AAFVGLYYLL-HWYRERQVVSHLQDKYIFITGCDSGFGNLLARQLDA
SDR9C6 M. musculus          -------MWFYL-VTLVGLYHLL-RWYRERQVVSHLQDKYVFITGCDSGFGNLLARQLDR
SDR9C6 S. harrisii          -------MWLYV-ASLIGLYYLF-RWYREKQVVSNLEDKYVFITGCDSGFGNQLAKQLDL
SDR9C6 X. tropicalis        -------MWLPLLVI-ITLMLLY-RWSRQRKKIQNLSDKYVFITGCDSGFGNVLAKQLDK
SDR9C6 A. carolinensis      -------MWIYL-VPAVILYFVI-RFYRERQTVENLGEKYVFITGCDSGFGNQVAKQLDI
SDR9C7 H. sapiens           ------------MAALTDLSFMY-RWFKNCNLVGNLSEKYVFITGCDSGFGNLLAKQLVD
SDR9C7 P. troglodytes       ------------MAALTDLSFMY-RWFKNCNLVGNLSEKYVFITGCDSGFGNLLAKQLVD
SDR9C7 M. musculus          ------------MAALTDFAFMY-RWFKNCNLVKNLSEKYVFITGCDSGFGNLLAKQLVD
SDR9C7 S. harrisii          ------------MGGIMDISFMY-RWFKNLNLVPNLSDKYVFITGCDSGFGNLLARKLDQ
SDR9C7 X. tropicalis        -------MWLLLLVVAAAMIFLY-RWNIQRQILPNLTDKYVLITGCDSGFGKLLAKQLDG
SDR9C8 H. sapiens           -------MWLYL-AVFVGLYYLL-HWYRERQVLSHLRDKYVFITGCDSGFGKLLARQLDA
SDR9C8 P. troglodytes       -------MWLYL-AVFVGLYYLL-HWYRERQVLSHLRDKYVFITGCDSGFGKLLARQLDA
SDR9C8 M. musculus          -------MWLYL-VALVGLWTLL-RFFRVRQVVSHLQDKYVFITGCDSGFGTLLARQLDR
SDR9C8 M. domestica         -------MWLYL-AVLVGLYFLF-RWLRERQVVSNLGDKYVFITGCDSGFGNLLAKQLDL
```

```
SDR9C8 P. humilis          -------MWLYAVAVLLGLFLLR-RWHRERQTVPRLSEKHVLITGCDSGFGNLLARQLDA
SDR9C8 A. carolinensis     -------MWLYL-AAFLGLYLLC-RWYREKQILSHLTEKYVFITGCDSGFGNLVARQLDA
SDR9C8 X. tropicalis       -------MWLFLLVL-LALILLY-RWNIGRKMLPNLTDKYVFITGCDSGFGNLLAKQLDR
SDR9C8 P. reticulata       -------MFLYI-LGLVAVWYLY-RWYKESERVPNKGDKYVYITGCDSGFGNSLAKHLDK
                                                                         *   * *

SDR9C1 H. sapiens          KGFLVFAGCLMkdKGHDGVKELDSLNSDRLRTVQLNVCSSEEVEKVVEIVRSSLKDPEkg  3p.2 4p.2
SDR9C1 P. paniscus         KGFLVFAGCLMkdKGHDGVKELDSLNSDRLRTVQLNVCSSEEVEKAVEIVRSSLKDPEkg  3p.2 4p.2
SDR9C1 M. musculus         KGFLVFAGCLMkdKGDAGVKELDSLKSDRLRTIQLNVCNSEEVEKAVETIRSGLKDPEkg  3p.2 4p.2
SDR9C1 M. domestica        LGFRVFAGCLFkdKGGKGVEELDSMKSERMQTIQLDVCQSAEVEKAVKTIQESLEDPEkg  3p.2 4p.2
SDR9C1 C. livia           KGFIIYAGCLQkdKGEGGSKDLDNMNSDRMRTLQLNVCDSKEVDRAVEHVNRTLQDPEkg  3p.2 4p.2
SDR9C1 C. picta           KGFIVYAGCLFkdQGAAGSQELDNMKSERMRTIQLNVCDSQEIERAVEHMGASLKDPQkg  3p.2 4p.2
SDR9C1 X. tropicalis      KGFIVYAGCLFkdKGEAGVKELDSMKSDRMRTIQLNVVKQDEVDRTVEIIRENLTNPEkg  3p.2 4p.2
SDR9C1 D. rerio          LGFTVFAGCLFkdG--EGAKELENIHSEKLKVVQLDVCSEEQVSQAVQFVTENLPDSEkg  3p.0 4p.2
SDR9C2 H. sapiens         LGFTVFAGVLNENG--PGAEELRRTCSPRLSVLQMDITKPVQIKDAYSKVAAMLQ--Drg       2p.2
SDR9C2 P. troglodytes     LGFTVFAGVLNENG--PGAEELRRTCSPRLSVLQMDITKPVQIKDAYSKVAAMLQ--Drg       2p.2
SDR9C2 M. musculus        LGFTVFAGVLDKEG--PGAEELRKHCSERLSVLQMDVTKPEQIKDAHSKVTEKIQ--Dkg       2p.2
SDR9C2 M. domestica       LGFTVFVGVLNEKG--PGAEALKKTCSKRTSVFQMDITKPAQIREVQARVAEKVQ--Qtg       2p.2
SDR9C2 G. gallus          LGFIVFAGVLNKDG--PGAEELRRTCSQRLSLLQLDITNPTQVKEAYLQVSEKVQ--Ktg       2p.2
SDR9C2 A. mississippiensis LGFVVFAGVLNKEG--PGAEALKRSCSQRLSVLQMDITNPTQIREAYLAVSEKVQ--Nag       2p.2
SDR9C2 X. tropicalis      LGVHVFAGVLDKKG--PGAEELKRVCSTHLCLIQLNITNCEEIRKAYKEISSYIQ--Dag       2p.2
SDR9C2 D. rerio          AGMRVFAGVLDELS--PGALKLKESASVNLTVLQLDITNNTQITQTHQFIKSQTG--Ktg       2p.2
SDR9C3 H. sapiens         MGFTVLATVLELNS--PGAIELRTCCSPRLRLLQMDLTKPGDISRVLEFTKAHTT--Stg       2p.2
SDR9C3 P. troglodytes     MGFTVLATVLELNS--PGAIELRTCCSPRLRLLQMDLTKPGDISRVLEFTKAHTT--Stg       2p.2
SDR9C3 M. musculus        MGFTVLATVLDLNS--PGALELRDLCSPRLKLLQMDLTKAEDISRVLEITKAHTA--Stg       2p.2
SDR9C3 C. picta           MGFKVFASVLDLQS--AGAKELRQRCSPSLTLLQMDLTKPEDIRKAQQLVQPQTA--Rtg       2p.2
SDR9C3 X. tropicalis      MGFKVIATVLNLES--PGARKLRKICSDNLTIIQMDLTKQEDIQKAQQTTKLHTA--Grg       2p.2
SDR9C3 D. rerio          MGFEVFATVLNLEG--EGAKHLRKVCSSRLTLLQVDITQPQQVQQALLDTKAKLG--Ird       2p.2
SDR9C4 H. sapiens         KGFHVIAACLTE----SGSTALKAETSERLRTVLLDVTDPENVKRTAQWVKNQVG--Ekg       1p.2
SDR9C4 P. troglodytes     KGFHVIAACLTE----SGSTALKAETSERLCTVLLDVTNPENVKRTAQWVKNQVG--Ekg       1p.2
SDR9C4 M. musculus        KGFRVIAACLTE----SGSAALKAKTSERLHTVLLDVTDPENVKKTAQWVKSHVG--Ekg       1p.2
SDR9C4 M. domestica       KGFRVLAACLTE----SGCRDLKASTSGKLQTVLLDVTKPDNVKRTAQWVKDEVG--Eng       1p.2
SDR9C4 G. gallus          KGFRVLASCLTE----TGAKELQAATSDQLQTVLLDVRDSNSVKKAAAWIKAKVQ--Seg       1p.2
SDR9C4 C. mydas           KGFRVLASCLTE----AGAVELKAATSEQLQTVLLDVTDPDNVRKVAEWIKAEVG--Sag       1p.2
SDR9C4 X. tropicalis      QGFRILATCLTE----AGATGLREATSQRLKTTLLDVTIAENVVSMAEWVKGEVG--Srg       1p.2
SDR9C4 D. rerio          KGFRVIAGCYSE----KGEDELKKICSDRLITLHLDVTDNENVKKAAETIKSLVG--Qkg       1p.2
SDR9C5 H. sapiens         RGFRVLASCLTP----SGAEDLQRVASSRLHTTLLDITDPQSVQQAAKWVEMHVK--Eag       1p.2
SDR9C5 P. troglodytes     RGFRVLASCLTP----SGAEDLQRVASSRLHTTLLDITDPQSVQQAAKWVEMHVK--Eag       1p.2
SDR9C5 M. musculus        KGFQVLAGCLTP----SGAEDLQQMASSRLHTTLLDITDPQNVQQVAKWVKTRVG--Etg       1p.2
SDR9C5 S. harrisii        RGFRVLAACLTP----TGAENLKQAASPRLCTTLLDVTDPQNIQQVAEWVGTLVG--Erg       1p.2
SDR9C5 G. gallus          RGFRVLATCLTP----QGADGLQRGCAGHLRTTLLDVTRSDSIRRAAEWVREEVG--Ekg       1p.2
SDR9C5 A. carolinensis    KGFQVLAGCLTQ----KGADNLQRSSSPNLRTTLLDVTNSESIRKAVEWVKGEVG--Ekg       1p.2
SDR9C5 X. tropicalis      KGFQVLAGCLTQ----AGADDLQKACPTGLKCTLLDVTSQESINKAVEWVKSEVG--Drg       1p.2
SDR9C5 D. rerio          RGFRVLAGCLTE----KGADDLKRAAGPFLKTCILDVTSSASIQKAMEWTKNEVG--Dkg       1p.2
SDR9C6 H. sapiens         RGLRVLAACLTE----KGAEQLRGQTSDRLETVTLDVTKMESIAAATQWVKEHVG--Drg       1p.2
SDR9C6 P. paniscus        RGLRVLAACLTE----KGAEQLRGQTSDRLETVTLDVTKMESIAAATQWVKEHVG--Drg       1p.2
SDR9C6 M. musculus        RGMRVLAACLTE----KGAEELRNKTSDRLETVILDVTKTESIVAATQWVKERVG--Drg       1p.2
SDR9C6 S. harrisii        RGLRVLAACLTE----KGAEQLRHQTSDRVKTVILDVTKTESITAAAQWVKECVG--Nkg       1p.2
SDR9C6 A. carolinensis    QGLRVLAACLTQ----EGAEELERLTSDRLKTTILDVTSTESVKAASEWVKGIVG--Nkg       1p.2
SDR9C6 X. tropicalis      QGIQVLATCLTE----KGAETLKSETSSRLRTVNMNVTDSQSVKSAAEWVTGIVG--Dag       1p.2
SDR9C7 H. sapiens         RGMQVLAACFTE----EGSQKLQRDTSYRLQTTLLDVTKSESIKAAAQWVRDKVG--Eqg       1p.2
SDR9C7 P. troglodytes     RGMQVLAACFTE----EGSQKLQQDTSYRLQTTLLDVTKSESIKAAAQWVRDKVG--Eqg       1p.2
SDR9C7 M. musculus        RGMKVLAACLTE----EGAQKLLQDTSHQLQTFLLDVTKSENVKEAAQWVRDQVG--Eqg       1p.2
SDR9C7 S. harrisii        RGMRVLATCFTE----EGAQELRRACSHRLQITFLDVTKTESIKAAAQWVNAQVG--Drg       1p.2
SDR9C7 X. tropicalis      QGLKVLATCLTQ----KGAEELKKETSSRLKTVIMDVSDSESVSKAAQWVSQIVG--Nag       1p.2
SDR9C8 H. sapiens         RGLRVLAACLTE----KGAEQLRGQTSDRLETVTLDVTKTESVAAAAQWVKECVR--Dkg       1p.2
```

```
SDR9C8 P. troglodytes       RGLRVLAACLTE----KGAEQLRGQTSDRLETVTLDVTKTESVAAAAQWVKECVR--Dkg       1p.2
SDR9C8 M. musculus          RGMRVLAACLTE----KGAEELRNKTSDRLETVILDVTKTESIVTATQWVKEHVG--Nrg       1p.2
SDR9C8 M. domestica         RGLRVLAACLSE----EGAQRLRRATSERVETVILDVTKTESITAAAQWVKERVG--Nkg       1p.2
SDR9C8 P. humilis           RGLRVLAACLTD----SGAAQLRAATSDRLQTVLLDVTSSKSIADVTAWVRERVG--Dqg       1p.2
SDR9C8 A. carolinensis      RGLRVLAGCFTE----EGAKKLKEATSQRLQTISLDVTKTESVAQAAAWAKGIVG--Dkg       1p.2
SDR9C8 X. tropicalis        RGLWVLAACLTD----KGAEELKKETSSRLKTVILNVTDSQSVISASAWVSDIVG--Nkg       1p.2
SDR9C8 P. reticulata        LGFCVIAGCYTE----NGETELKKVSSERLTAISLDVSKSESVKKVAAFIKTLVG--Dkg       1p.2
                             *               *   *

SDR9C1 H. sapiens           MWGLVNNAGIST-FGEVEFTSLETYKQVAEVNLWGTVRMTKSFLPLIRRAkgRVVNISSM  5p.2
SDR9C1 P. paniscus          MWGLVNNAGIST-FGEVEFTSLETYKQVAEVNLWGTVRMTKSFLPLIRRAkgRVVNISSM  5p.2
SDR9C1 M. musculus          MWGLVNNAGIST-FGEVEFTSMETYKEVAEVNLWGTVRTTKSFLPLLRRAkgRVVNISSM  5p.2
SDR9C1 M. domestica         LWGLVNNAGIST-FGEVEFTSMETYKEVAEVNLWGTIRTTKACLPLIRRAkgRVVNISSM  5p.2
SDR9C1 C. livia            LWGLVNNAGIST-FGEVEFTSMDTYMEVAEVNLWGTVRTTKAFLPLIRRSkgRVVNISSM  5p.2
SDR9C1 C. picta            LWGLVNNAGIST-FGEVEFTSMETYKEVAEVNLWGTIRTTKAFLPLIRRAkgRVVNISSM  5p.2
SDR9C1 X. tropicalis        LWGVVNNAGIST-FGEVEFTSMETYKEVAEVNLWGTVRVTKACLPLIRRAkgRVVNISSM  5p.2
SDR9C1 D. rerio             LWGVVNNAGIST-FGEVEFTTMDTYKQVSEVNLWGTIRVTKAFLPLIRRAkgRVVNIASM  5p.2
SDR9C2 H. sapiens           LWAVINNAGVLGFPTDGELLLMTDYKQCMAVNFFGTVEVTKTFLPLLRKSKGRLVNVSSM
SDR9C2 P. troglodytes       LWAVINNAGVLGFPTDGELLLMTDYKQCMAVNFFGTVEVTKTFLPLLRKSKGRLVNVSSM
SDR9C2 M. musculus          LWAVVNNAGVFHLPIDGELIPMSIYRKCMAVNFFGTVEVTKAFLPLLRKSKGRLVNVSSM
SDR9C2 M. domestica         LWAIVNNAGILGSIGDGELLPMEVYRQCMDVNFFGAVEVTKAFMPLLRKSKGRVVNVSSL
SDR9C2 G. gallus            LWGVVNNAGILGFPADGELLPMSMYRQCMEVNFFGAVHVSKTFLPLLRKSRGRLVNMSSM
SDR9C2 A. mississippiensis  LWGIINNAGVLGFTADSELLPMSVYRQCMDVNFFGAVEVSKMFLPLLRKSQGRLVNMSSM
SDR9C2 X. tropicalis        LWGIVHNAGVLGYVADGELIPFSVYRQCMEVNFLGAVQVTQIFAPLLRKSKGRLVSISSM
SDR9C2 D. rerio             LWALVNNAGVLGYVCDGEILPMKMYKSCLDVNFLGSVMMTHTFLPLIRQSRGRVINITSM
SDR9C3 H. sapiens           LWGLVNNAGHNEVVADAELSPVATFRSCMEVNFFGALELTKGLLPLLRSSRGRIVTVGSP
SDR9C3 P. troglodytes       LWGLVNNAGHNEVVADAELSPVATFRSCMEVNFFGALELTKGLLPLLRSSRGRIVTVGSP
SDR9C3 M. musculus          LWGLVNNAGLNIVVADVELSPVATFRKCMEVNFFGALELTKGLLPLLRHSRGRIVTVGSP
SDR9C3 C. picta            LWGLVNNAGFDDTIADAELSPLCKFRTCMDVNFFGTLELTKALLPLLRSARGRIVTVSSP
SDR9C3 X. tropicalis        LWALINNAGYCAHFGDAELTLMSTYKACMDVNFFGTLELTQALLPFIRYAKGRIVTVGSP
SDR9C3 D. rerio             LWGLVNNAGWCVNIGDAELSLMSNYRGCMEVNFFGTVTVTRTFLPLLRQSKGRIVTISSP
SDR9C4 H. sapiens           LWGLINNAGVPGVLAPTDWLTLEDYREPIEVNLFGLISVTLNMLPLVKKAQGRVINVSSV
SDR9C4 P. troglodytes       LWGLINNAGVPGVLAPTDWLTLEDYREPIEVNLFGLISVTLNMLPLVKKAQGRVINVSSV
SDR9C4 M. musculus          LWGLINNAGVLGVLAPTDWLTVDDYREPIEVNLFGLINVTLNMLPLVKKARGRVINVSSI
SDR9C4 M. domestica         LWGLINNAGILGVLAPTDWLTVEHFREPFEVNLFGLINVTLNMLPLVKKAQGRIINISSI
SDR9C4 G. gallus            LWGLVNNAGIMGPTAPTDWLDIEHFREPIEVNLIGLINVTINMLPLIKLAKGRIVNVSSI
SDR9C4 C. mydas             LWGLVNNAGIMGPSAPTDWLNIEHFRAPIETNLIGLINVTLHLLPLVKKARGRLVNISST
SDR9C4 X. tropicalis        LWGLINNAGVMGTLAPFDWLTIEDIKKPMEINLIGLIHVTLVLLPFIKKAKGRIINVSSI
SDR9C4 D. rerio             LWAVVNNAGIAFPTAPNDWLEIEDFTPMINVNLIGVIAVTLSVLPLIKKAKGRVVNVASV
SDR9C5 H. sapiens           LFGLVNNAGVAGIIGPTPWLTRDDFQRVLNVNTMGPIGVTLALLPLLQQARGRVINITSV
SDR9C5 P. troglodytes       LFGLVNNAGVAGIIGPTPWLTRDDFQRVLNVNTMGPIGVTLALLPLLQQARGRVINITSV
SDR9C5 M. musculus          LFGLVNNAGVAGIIGPTPWLTQDDFQRVLSVNTLGPIGVTLALLPLLQQARGRVVNITSV
SDR9C5 S. harrisii          LFGLVNNAGITGIIGPTPWLNLEDYRKVLEVNTLGPIGVTLALLPLLRQSRGRIINISSV
SDR9C5 G. gallus            LFGLVNNAGVANPIGPTEWMRIEDYRQVMAVNTFGAIEVTLQLLPLLKRARGRVVNTSSV
SDR9C5 A. carolinensis      LFGLVNNAGVANPIGPTEWMIVEDYRKVMSINTFGMIEVSLAFLPLLKQARGRVVNTSSV
SDR9C5 X. tropicalis        LYGLVNNAGIANPIGPTEWMTIQDYKRVMDVNAFGTIAVSLSFLSLIKKAQGRIINMASI
SDR9C5 D. rerio             LWGLVNNAGRSLPMGPSEWMKIEDFESTLKVNMTGVIETTMTFLPLVKKARGRIVNVASV
SDR9C6 H. sapiens           LWGLVNNAGILTPITLCEWLNTEDSMNMLKVNLIGVIQVTLSMLPLVRRARGRIVNVSSI
SDR9C6 P. paniscus          LWGLVNNAGILTPITLCEWLNTEDSMNMLKMNLIGVIQVTLSMLPLVRRARGRIVNVSSI
SDR9C6 M. musculus          LWGLVNNAGVLQPFAYIEWYRPEDYMPIFQVNLIGLTQVTISMLFLVKKARGRIVNVSSA
SDR9C6 S. harrisii          LWGLVNNAGIVLPHICTEMMTKEILTKTLNVNLIGLIEVTLNMLPMIRQARGRIVNVCSI
SDR9C6 A. carolinensis      LWGLVNNAGILRTTCPNEWLTKEDFEKVLNVNLLGLIDVTLQMLPLLRKAKGRVVNVASI
SDR9C6 X. tropicalis        LWGLVNNAGYGFVFSPTGWQTKEHFVKVLEINLLGMVDVTLNLLPLLRKAQGRIVNVSSN
SDR9C7 H. sapiens           LWALVNNAGVGLPSGPNEWLTKDDFVKVINVNLVGLIEVTLHMLPMVKRARGRVVNMSSS
SDR9C7 P. troglodytes       LWALVNNAGVGLPSGPNEWLTKEDFVKVINVNLVGLIEVTLHMLPMVKRARGRVVNMSSS
SDR9C7 M. musculus          LWALVNNAGVGLPSGPNEWLTIKDFVKVININLVGLIDVTLNMLPMIKKARGRVVNMSSS
```

```
SDR9C7 S. harrisii          LWGLVNNAGVGLPSGPNEWLTKEDFAKVINVNLIGMIEVTLNMLPMIKRARGRVVNMSSS
SDR9C7 X. tropicalis        LWGLVNNAGIALPIGPNGWMKKEHFVKMIDVNLLGMVDVTLTLLPLIRRARGRIVNVSSS
SDR9C8 H. sapiens           LWGLVNNAGISLPTAPNELLTKQDFVTILDVNLLGVIDVTLSLLPLVRRARGRVVNVSSV
SDR9C8 P. troglodytes       LWGLVNNAGISLPTAPNELLTKQDFMTILDVNLLGVIDVTLSLLPLVRRAKGRVVNVSSV
SDR9C8 M. musculus          LWGLVNNAGISTPSGPNEWMKKQDFAHVLDVNLLGMIEVTLSMLPLVRKARGRVVNVSSV
SDR9C8 M. domestica         LWGLVNNAGISMPTAPNDWLTKEDFLRVINVNLIGLIEVTLSLLSLVRKAKGRIVNVSSI
SDR9C8 P. humilis           LWGLVNNAGIAIPTGPNEWLTKQDFVKVLDVNLIGVVEVTLSLLPLLRRARGRVVNVASV
SDR9C8 A. carolinensis      LWGLVNNAGISIPTAPNEWLTKEDFQKILDINLLGVFDVTKHMLPLLRKAKGRIVNVASV
SDR9C8 X. tropicalis        LWGLVNNAGISNPIAPNEWLSKEDYLKVLNVNLLGVIDITLKLLPLIRKARGRIVNVASV
SDR9C8 P. reticulata        LWAVVNNAGVATPSGPTEWLTIDDYKSMLAVNLNGVIDVTLSVLPLIKKARGRVVNVASV
                                  ***                      *  *                **     *


                                       +   +
SDR9C1 H. sapiens           LGRMANPARSPYCITKFGVEAFSDCLRYEMYPLGVKVSVVEPGNFIAATSLY-SPESIQA
SDR9C1 P. paniscus          LGRMANPARSPYCITKFGVEAFSDCLRYEMYPLGVKVSVVEPGNFIAATSLY-SPESIQA
SDR9C1 M. domestica         LGRMSSPSRSPYCITKYGVEAFSDCLRYEMYPLGVRVCVVEPGNFIAATSLY-SPERIRA
SDR9C1 M. musculus          LGRMANPARSPYCITKFGIEAFSDCLRYEMHPLGVKVSVVEPGNFIAATSLY-SPERIQA
SDR9C1 C. livia             MGRMGSPARSPYCITKFGVEAFSDCLRYEMQPQGVTVSIVEPGNFIAATNLY-SPERIKA
SDR9C1 C. picta             MGRMGSPARSSYCITKFGVEAFSDCLRYEMQPQEVKICVVEPGNFIGATNLY-TPERIKA
SDR9C1 X. tropicalis        LGRMANPARSPYCITKFGVEAFSDCLRYEMHPLGVKVSVVEPGNFIAGTSLY-SPEKIKA
SDR9C1 D. rerio             YGRMGNALRSPYCISKYGVEAFSDCLRYEMKTWGVKVSIIEPGNFIVATGIL-TRDIVTT
SDR9C2 H. sapiens           ggGAPMERLASYGSSKAAVTMFSSVMRLELSKWGIKVASIQPGGFL--tnIAGTSDKWEK  3p.2 4p.2
SDR9C2 P. troglodytes       ggGAPMERLASYGSSKAAVTMFSSVMRLELSKWGIKVASIQPGGFL--tnIAGTSDKWEK  3p.2 4p.2
SDR9C2 M. musculus          ggTVPLQMTSAYAATKAALTMFSTIIRQELDKWGVKVVTIKPGGFK--tnITGSQDIWDK  3p.2 4p.2
SDR9C2 M. domestica         agAMPMKFLAAYCASKAALTMFSAVMRMELEKWGIKVVIIHPAGFK--tfIFGTKETLGM  3p.2 4p.2
SDR9C2 G. gallus            tgGIPLPRYAAYGASKAALSMFSGVMRQELSKWGIKVAAIHPSGFR--tgIQGTPELWVK  3p.2 4p.2
SDR9C2 A. mississippiensis  agGVPMPRLSAYGASKAALSMFSGVMRQELSKWGIKVAVVHPSGFR--tcIQGTPELWDK  3p.2 4p.2
SDR9C2 X. tropicalis        ggHVPLNGFAAYASSKAALSMFSAVMRQDLSKWGVKVAVVCPSGFR--tnIFGSQ-----  3p.2 4p.2
SDR9C2 D. rerio             agEVPLVGFAGYGASKAALNIYSGAIRQELSRWGVRVIIVQPGAFR--tnILGSSEQWER  3p.2 4p.2
SDR9C3 H. sapiens           agDMPYPCLGAYGTSKAAVALLMDTFSCELLPWGVKVSIIQPGCFK--teSVRNVGQWEK  3p.2 4p.2
SDR9C3 P. troglodytes       agDMPYPCLGAYGTSKAAVALLMDTFSCELLPWGVKVSIIQPGCFK--teSVRNVGQWEK  3p.2 4p.2
SDR9C3 M. musculus          agDMPYPCLAAYGTSKAAIALLMDTFGCELLPWGIKVSIIKPGCFK--tdAVTNVNLWEK  3p.2 4p.2
SDR9C3 C. picta             agDMPYPCLAAYGASKAALTLLMDTFRSELEPWGVKVSVILPGYFK--tgTSCDPAYWHE  3p.2 4p.2
SDR9C3 X. tropicalis        agEHSFPYLAAYGSSKAALNRVMDIFRHELMPWGVKVILILPASYK--tgAHDNHIHWEN  3p.2 4p.2
SDR9C3 D. rerio             sgEHPFPCLASYGASKAALNLFINTLRHELDPWGVKVSTILPSAYK--tgQSSNAEYWEK  3p.2 4p.2
SDR9C4 H. sapiens           GGRLAI-VGGGYTPSKYAVEGFNDSLrrDMKAFGVHVSCIEPGLFK--TNLA-DPVKVIE  2p.1
SDR9C4 P. troglodytes       GGRLAI-VGGGYTPSKYAVEGFNDSLrrDMKAFGVHVSCIEPGLFK--TNLA-DPVKVIE  2p.1
SDR9C4 M. musculus          GGRLAF-GGGGYTPSKYAVEGFNDSLrrDMKAFGVHVSCIEPGLFK--TELA-DPIKTTE  2p.1
SDR9C4 M. domestica         GGRLAF-SGGGYTPSKYAVEGFNDSLrrDMKEFGVKVSCIEPGLFK--TGLS-DPVKAIE  2p.1
SDR9C4 G. gallus            GGRLAF-CGGGYCPSKFGVEGFNDSLrrDMKAFGVKVSCIQPGLFK--TPLT-DLAKILK  2p.1
SDR9C4 C. mydas             GGRLAV-WGGGYVPSKFGVEGFNDSLrrDMKAFGVKVSCIEPGLFK--TGLS-NRKKVIE  2p.1
SDR9C4 X. tropicalis        GGRVAA-SGGAYFSSKFGVEGFNDSLrrDMKAFGVQVSCIEPGLFK--TPLS-DPKKVLQ  2p.1
SDR9C4 D. rerio             FGRIST-LGGAYCITKYGVEAFNDALrrQMAPFGVKVLCIEPGFFK--TIVT-DFNIVES  2p.1
SDR9C5 H. sapiens           LGRLAA-NGGGYCVSKFGLEAFSDSLrrDVAHFGIRVSIVEPGFFR--TPVT-NLESLEK  2p.1
SDR9C5 P. troglodytes       LGRLAA-NGGGYCVSKFGLEAFSDSLrrDVAHFGIRVSIVEPGFFR--TPVT-NLESLEK  2p.1
SDR9C5 M. musculus          LGRIAA-NGGGYCVSKFGLEAFSDSLrrDMAPFGVQVSIVEPGFFR--TPVT-NLESLES  2p.1
SDR9C5 S. harrisii          LGRLAA-NGGGYCVSKYGVEAFSDSLrrDIAHFGVRVSIVEPGFFR--TAAT-DLESVEG  2p.1
SDR9C5 G. gallus            LGRLSA-NGGGYCVSKYCIEAFSDSLrrDMYHFGVKVSIVEPGFFK--TAVT-NLESIEA  2p.1
SDR9C5 A. carolinensis      LGRLSA-NGGGYCISKYCVEAFSDSLrrDMYHFGVKVSIVEPGFFK--TAVT-NLDSIES  2p.1
SDR9C5 X. tropicalis        LGRISA-NGGGYCVSKYAVEAFSDSLrrDMQHFGVRVCIIEPGFFK--TAVT-NLDSIER  2p.1
SDR9C5 D. rerio             LGRVAA-NGGGYCISKFAVESFSDCLrrDIQGFGVNVCIIEPGFFK--TQVT-SLEPIER  2p.1
SDR9C6 H. sapiens           LGRVAF-FVGGYCVSKYGVEAFSDILrrEIQHFGVKISIVEPGYFR--TGMT-NMTQSLE  2p.1
SDR9C6 P. paniscus          LGRVAF-FVGGYCVSKYGVEAFSDILrrEIQHFGVKISIVEPGYFR--TGMT-NMTQSLE  2p.1
SDR9C6 M. musculus          LGRVAL-FGGFYSCSKYGVEAFSDVLrhEVQDFGVKVSIIEPGSFK--TEMT-DAELTIE  2p.1
SDR9C6 S. harrisii          LGRMAA-FSTAYCSSKFGVEAFSDCLrlEMQYFGVKVCIIEPGYFK--TNLT-NEETFVE  2p.1
SDR9C6 A. carolinensis      LGRLSF-YGGGYCPSKYGVEAFSDSLrrELSPFGVKVSVIEPGYFK--TPMT-NVQDTLN  2p.1
```

```
SDR9C6 X. tropicalis        AGRLAF-IGGGYCLSKFGVEAFSDSLrrELLDFGIKVSIIEPGAFR--TGMG-VSGPHLQ  2p.1
SDR9C7 H. sapiens           GGRVAV-IGGGYCVSKFGVEAFSDSIrrELYYFGVKVCIIEPGNYR--TAIL-GKENLES  2p.1
SDR9C7 P. troglodytes        GGRVAV-IGGGYCVSKFGVEAFSDSIrrELYYFGVKVCIIEPGNYR--TAIL-GKENLES  2p.1
SDR9C7 M. musculus          GGRVAI-FGGGYCVSKFGVEAFSDSIrrELHFFGVKVSIIEPGNYK--TSIL-GQEALES  2p.1
SDR9C7 S. harrisii          GGRVAV-IGGGYCVSKFGVEAFSDSIrrELYYFGVKVCIIEPGNYR--TAIL-GTEDIEQ  2p.1
SDR9C7 X. tropicalis        MGRIAL-FGGAYSISKHGVEAFSDCLrrELSRFGIKVSIIEPGGFK--TSIF-SFSVCKK  2p.1
SDR9C8 H. sapiens           MGRVSL-FGGGYCISKYGVEAFSDSLrrELSYFGVKVAMIEPGYFK--TAVT-SKERFLK  2p.1
SDR9C8 P. troglodytes        MGRVSL-FGGGYCISKYGVEAFSDSLrrELSYFGVKVAMIEPGYFK--TAVT-SKERFLK  2p.1
SDR9C8 M. musculus          MGRVSL-FGGGYCISKYGVEAFSDSLrrELSYFGVKVAIIEPGFFL--TGVT-SSARLCS  2p.1
SDR9C8 M. domestica         MGRLSL-FGGGYCISKYGVEAFSDSLrrELYHSGVRIAMIEPGFFK--TNIT-NLELFKE  2p.1
SDR9C8 P. humilis           MGRVSF-FGGGYCISKYGVEAFSDSLrlEMNKFGVKVCVIEPGYFK--TMIT-NTDNLEK  2p.1
SDR9C8 A. carolinensis      MGRVSF-FGGGYCPSKYGVEAFSDSLriELARFGVKVCIIEPGYFQ--TAVT-NEKLLVD  2p.1
SDR9C8 X. tropicalis        AGRVSL-CGGGYSISKYGVESFSDSLrrEMAQFGIKVSIVEPGYFK--TPVA-DANTQKK  2p.1
SDR9C8 P. reticulata        FGRISP-FGGPYCVSKYGVESFNDSLrlNMAPFGVKVLCIEPGFFK--TSVT-DTVMLKK  2p.1
                             *         *   *                         *      *

SDR9C1 H. sapiens           IAKKMWEELPEVVRKDYGKKYFDEKIAKMET-YCSSGSTDTSPVIDAVTHALTATTPYTR
SDR9C1 P. paniscus          IAKKMWDELPEVVRKDYGKKYFDEKIAKMET-YCSSGSTDTSPVIDAVTHALTATTPYTR
SDR9C1 M. musculus          IAKKMWDDLPEVVRKDYGRKYFDEKIAKMET-YCNSGSTDTSSVINAVTHALTAATPYTR
SDR9C1 M. domestica         IANKMWNELPEVVRQDYGREYFEESVSKMER-YCTSGSTDTSAVIRAVRHALTSARPHTR
SDR9C1 C. livia            IADKMWDELPEIVRKDYGRKYFDEQVSKMET-YCNSGSTDTSPVIESVAHALMSTTPYTR
SDR9C1 C. picta            IAEKMWKELPETVRKDYGRKYLDEQITKMES-YCNSGSPDTSPVINSITHALTSSSPYAR
SDR9C1 X. tropicalis        IGDKMWADLPEVIKNDYGKKYFDEKIATMNT-YCNSGSTDTSEVIDAITHALTASTPYTR
SDR9C1 D. rerio             TAEKLWKEAPPIVQEDYGKAHFEQYMALMRS-YCNSGQREIEPVLDDITDAIMSQRPYTR
SDR9C2 H. sapiens           LEKDILDHLPAEVQEDYGQDYILAQRNFLLL-INSLASKDFSPVLRDIQHAILAKSPFAY
SDR9C2 P. troglodytes        LEKDILDHLPAEVQEDYGQDYILAQRNFLLL-INSLASKDFSPVLRDIQHAISAKSPFAY
SDR9C2 M. musculus          MEKEILDHFSKDIQENYGQDYVHTQKLIIPT-LKERSNPDITPVLRDIQHAISARNPSSF
SDR9C2 M. domestica         QEKNILDNISPDVLEAYGQDYIHSSIWPFLQ-TSEKCTTDFSPIFTDILNGILCKNPSFL
SDR9C2 G. gallus            QEKELVEHLSVDVQQDYGRDYLLGLKSYLLR-IPSCCDADLTPVLSSILHALLSKRPHGL
SDR9C2 A. mississippiensis  QEKHLMENLMPDVKTDYGEDYILALKNFLLH-MPSSCSSDLSPLLDDVLDALLAKSPHGL
SDR9C2 X. tropicalis        -NQVILDNVMPDVKEDYGEDYIENLKGLYHT-LHKFGSSDLSPVMEDACHALLAQTPNFL
SDR9C2 D. rerio             AQEQILSGLSEEVKDSYGEEYIHSMQKRLLD-MSSASSEDKGPFLQSLKHAILSSNPKHF
SDR9C3 H. sapiens           RKQLLLANLPQELLQAYGKDYIEHLHGQFLH-SLRLAMSDLTPVVDAITDALLAARPRRR
SDR9C3 P. troglodytes        RKQLLLANLPQELLQAYGKDYIEHLHGQFLH-SLRLAMSDLTPVVDAITDALLAARPRRR
SDR9C3 M. musculus          RKQLLLANIPRELLQAYGEDYIEHVHGQFLN-SLRMALPDLSPVVDAIIDALLAAQPRSR
SDR9C3 C. picta            KKEQLLASLPADLLQAYGEEYIHEINKQFVK-FMKTANEDLSSVVNSITDALIAASPAAR
SDR9C3 X. tropicalis        QHKKLLANLPIELLQEYGEEYITETQNRFLK-YGDTACSDFSPVIDSITDAIISENPKVK
SDR9C3 D. rerio             QYKSLLQGLSPNLLEEYGEEYLLETKELFQN-YAKTANEDLSPVIDTIVEALLSPQPQVR
SDR9C4 H. sapiens           KKLAIWEQLSPDIKQQYGEGYIEksLDK-LKGNKSYVNMDLSPVVECMDHALTSLFPKTH  3p.2
SDR9C4 P. troglodytes        KKLAIWEQLSPDIKQQYGEGYIEksLDK-LKGNKSYVNMDLSPVVECMDHALTSLFPKTH  3p.2
SDR9C4 M. musculus          KKLAIWKHLSPDIKQQYGEGYIEksLHR-LKSNTSSVNLDLSLVVGCMDHALTSLFPKTR  3p.2
SDR9C4 M. domestica         KKMAVWNQLSSDIKQQYGDGYIEksLAK-KAKNKPFQNMDLSLVVNCMDHALTSLFPQTH  3p.2
SDR9C4 G. gallus            EKEVIWNRLPPDTKKQYGEEYFQkaA-KKEKFSKACLNTDISLVVECMEHALTSLHPRAH  3p.2
SDR9C4 C. mydas             EREAIWNQLPPAIRKQYGEGYIQkdAARKEKLTQRLQNTNLSLVVQCMEHALTSINPRSR  3p.2
SDR9C4 X. tropicalis        QRTDIWKRLPTEIQKEYGDNYIQidASKKQKLNQRILNTDLSLVVQCMEHALTSRHPRTR  3p.2
SDR9C4 D. rerio             TLHRLWNKLPQEVKDEYGSDYVDktKLTAKELLEKLADGDLMKVVSCMEHAVAAVHPRTR  3p.2
SDR9C5 H. sapiens           TLQACWARLPPATQAHYGGAFLTkyLKMQQRIMNLICDPDLTKVSRCLEHALTARHPRTR  3p.2
SDR9C5 P. troglodytes        TLQACWARLPPATQAHYGGAFLIkyLKMQQRIMNLICDPDLTKVSRCLEHALTARHPRTR  3p.2
SDR9C5 M. musculus          TLKACWARLPPAIQAHYGEAFLDtyLRVQRRIMNLICDPELTKVTSCLEHALTARHPRTR  3p.2
SDR9C5 S. harrisii          ILQASWNRLSPATRATYGENFLSkyLQAQRLIINLICDGDLGKVSGCLEHALTARYPRTR  3p.2
SDR9C5 G. gallus            SLRQLWERLAPETRLSYGEEFFHkyLRVQRFIMNIICDADLGKVTRCMEHALGACHPRTR  3p.2
SDR9C5 A. carolinensis      SLRQIWDRMRPEARQSYGEDFFSnyLKVQKFIMNLICDPDLSKVTNCMEHALEAKHPRTR  3p.2
SDR9C5 X. tropicalis        SLQLLWDQMPPETKMTYGDKYFQqyLKVQRLIMNFICDADISKVPKCIEHALQARYPRTR  3p.2
SDR9C5 D. rerio             ELHRLWNQLTPEVKESYGDKYLDkyIWIQRLIMNAICDSDLSKVTNCMEHALLAVHPRTR  3p.2
SDR9C6 H. sapiens           RMKQSWKEAPKHIKETYGQQYFDalYNIMKEGL-LNCSTNLNLVTDCMEHALTSVHPRTR  3p.2
SDR9C6 P. paniscus          RMKQSWKEAPKHIKETYGQQYFDalYSIMKEGL-LNCSTNLNLVTDCMEHALTSVHPRTR  3p.2
```

```
SDR9C6 M. musculus          RTKKVWEAAPEHIKESYGQQFFDdfCSTTKREL-MKCSRNLSLVTDCMEHALTSTHPRTR  3p.2
SDR9C6 S. harrisii          NFKKSWNDCSVEIKESYGQEFFEktLNFIKVIM-KRCCRNLNLVTDCMEHALTCSYPRYR  3p.2
SDR9C6 A. carolinensis      YLENHWISVTQEIKDSYGQTYFTnlYKLLREDLTSRCNTNLFLVTDCMEHALTSKHPRTR  3p.2
SDR9C6 X. tropicalis        FLEQLWNNLDPETKKSYGEKYYQqyFQNVAC-LTSMTSPKLHKVTDCMEHALTAVHPWTR  3p.2
SDR9C7 H. sapiens          RMRKLWERLPQETRDSYGEDYFRiyTDKLKN-IMQVAEPRVRDVINSMEHAIVSRSPRIR  3p.2
SDR9C7 P. troglodytes      RMRKLWERLPQETRDSYGEDYFRiyTDKLKN-IMQVAEPRVRDVINSMEHAIVSRSPRIR  3p.2
SDR9C7 M. musculus         RMKKLWDRLPQETRDSYGEEYFQtyTKKLVN-LMRSAEPRISDVTNSMEHAIVSRSPRIR  3p.2
SDR9C7 S. harrisii         RMRYLWERLPQETRNSYGEQYYYlyTKNLKN-ITRLAEPKISEVTDCLEHALTSRNPRIR  3p.2
SDR9C7 X. tropicalis       SMEQLWADTSAETKECYGQQYLNetLQTIDE-LINTNSSKLCKVTNCMEHALTACHPWTR  3p.2
SDR9C8 H. sapiens          SFLEIWDRSSPEVKEAYGEKFVAdyKKSAEQ-MEQKCTQDLSLVTNCMEHALIACHPRTR  3p.2
SDR9C8 P. troglodytes      SFLEIWDRSSPEVKEAYGEKFVAdyKKSAEQ-MEQKCTQDLSLVTDCMEHALIACHPRTR  3p.2
SDR9C8 M. musculus         NTQMLWDQTSSEIREIYGEKYLAsyLKRLNK-LDKRCNKDLSGVTDCMEHALTACHPRTR  3p.2
SDR9C8 M. domestica        NFQSLWEQLDPEVRQHYGQKFFDayMRSTVD-MQALCKEDLSLVTNCMQHALTSRHPRSR  3p.2
SDR9C8 P. humilis          NFHSIWNKLPEEIKASYGENYLRrlVSTLKV-LEKTYNTDLSLVTNCMEHALTSLHPRYR  3p.2
SDR9C8 A. carolinensis     TFTRLWDRLPDEIKHAYGERYFEgmKKGTAE-MQAHCNPDLSLVTNCMEHALTAVYPRKR  3p.2
SDR9C8 X. tropicalys       FLNEIWAKLPQHIRETYGQEYFEkyCNNVER-NLEKVSSKLHLVTDCMEHALTAVYPRTR  3p.2
SDR9C8 P. reticulata       HFRSLWDRLPQDLKDDYGYAFLEtgLEQLDERFKQFRDNDLMKVVHCMEHAIASVHPRYR  3p.2
                                            **                                      *

SDR9C1 H. sapiens          YHPMD--YYWWLRMQIMTHLPGAISDMIYIR-----------------------------
SDR9C1 P. paniscus         YHPMD--YYWWLRMQIMTHLPGAISDMIYIR-----------------------------
SDR9C1 M. musculus         YHPMD--YYWWLRMQIMTHFPGAISDKIYIH-----------------------------
SDR9C1 M. domestica        YHPMD--YYWWLRMQIMTHTPAAVSDMLYIH-----------------------------
SDR9C1 C. livia            YHPMD--YYWWLRMQIMTHMPAAISDRLYVY-----------------------------
SDR9C1 C. picta            YHPMD--YYWWLRVQIMTHLPAAVADRIYIY-----------------------------
SDR9C1 X. tropicalis       YHPMD--YYWWLRMQIMTHMPGAISDKIYIH-----------------------------
SDR9C1 D. rerio            YSPME--PHWWIRIQIMTHLPGAISDRLYF------------------------------
SDR9C2 H. sapiens          YTPGKGAYLWICL---AHYLPIGIYDYFAKRHFGQD-KPMPRALRMPNYKKKAT------
SDR9C2 P. troglodytes      YTPGKGAYLWICL---AHYLPIGIYDYFAKRHFGQD-KPMPRALRMPNYKKKAT------
SDR9C2 M. musculus         YYPGRMAYLWVCL---AAYCPTSLLDYVIKKGFYP--QPTPRALRTVH------------
SDR9C2 M. domestica        YTAGMFSYLCHCL---IFYFPVSVFDYASKKMFPT--KTLPKALT---------------
SDR9C2 G. gallus           YTPGKGAYMMLCI---FCYFPLWFYDFLVSKSLGL--ESIPQALRTSEAENKDL------
SDR9C2 A. mississippiensis YTPGKNAYLALCI---FCYFPLWFYDFFIGKSLCV--KILPRALRGPDSKSKNT------
SDR9C2 X. tropicalis       YTPGRLAYLIPCL---YYYFPQWISDWMGMQAFQISRNMLPRALRNNNKSI---------
SDR9C2 D. rerio            YYPGAGAWVLSLL---YRYCPTALSDKIFSGMFMSGVQPAELARAIPH------------
SDR9C3 H. sapiens          YYPGQGLGLMYFI---HYYLPEGLRRRFLQAFFI-S-HCLPRALQPGQPGTTPPQDAAQD
SDR9C3 P. troglodytes      YYPGQGLGLMYFI---HYYLPEGLRRRFLQAFFI-S-HCLPRALQPGQPGTTPPQDAAQD
SDR9C3 M. musculus         YYPGRGLGLMYFI---HHYLPEGLRRCFLQNFFI-N-HLLPRALRPGQHGPAPA------
SDR9C3 C. picta            YYPGVGMWLIYFI---HHYLPSIIRDLFLKAFFI-N-QKLPRALQPKQQNGLKHE-----
SDR9C3 X. tropicalis       YYAGKNLWILYLI---GIYLPHSVSDNFCKCLFLKN-NVLPRSLRKQKTNE---------
SDR9C3 D. rerio            YYAGPGLILMYFI---CSYLPLSISDRFLQKLFVQK-KVMPRALIKQQGLSPNDNNNSIK
SDR9C4 H. sapiens          YAAGKDAKIFWIP---LSHMPAALQDFLLLKQKAELAN----PKAV--------------
SDR9C4 P. troglodytes      YAAGKDAKIFWIP---LSHMPAALQDFLLLKQKAELAN----PKAV--------------
SDR9C4 M. musculus         YIAGKDAKTFWIP---LSHMPAVLQDFLLLKQKVELAN----PKAV--------------
SDR9C4 M. domestica        YIAGVDAKTFWIP---LSLMPAILQDFLLQKHKVKLAD----PKAV--------------
SDR9C4 G. gallus           YVVGQDAKWFWTP---LSRMPADVQDFLLLGNREKPAV----SHRK--------------
SDR9C4 C. mydas            YSAGWDAKLLWIP---LSSMPAALQDFVLLRNKAELAD----PTAG--------------
SDR9C4 X. tropicalis       YSAGSDAAFLWIP---LSYMPTFIQDFVILRNKVKIPT----SGNTVH------------
SDR9C4 D. rerio            YSPGWDAKFFWLP---LSYMPTFISDALLLKKAVQPKA----SIL---------------
SDR9C5 H. sapiens          YSPGWDAKLLWLP---ASYLPASLVDAVLTWVLPKPAQ----AVY---------------
SDR9C5 P. troglodytes      YSPGWDAKLLWLP---ASYLPASLVDAVLTWVLPKPAQ----AVY---------------
SDR9C5 M. musculus         YSPGWDAKLLWLP---ASYLPARVVDAVLTWILPRPAQ----SVS---------------
SDR9C5 S. harrisii         YSPGWDAKLLWLP---ASYLPASLVDAILTFVLPKPAQ----TVC---------------
SDR9C5 G. gallus           YSAGWDAKLLWLP---ASYLPACIVDFVLATILPKPAH----HVR---------------
SDR9C5 A. carolinensis     YSAGWDAKFMWLP---ISYLPAFMVDIVLATILPKPAQ----RVR---------------
SDR9C5 X. tropicalis       YSPGWDAKLVWLP---ASYMPAFITDAVLAFVLPKPKH----SIH---------------
```

```
SDR9C5 D. rerio             YSAGWDAKFLWIP---LSYMPACFVDIALKLVMPKPAK----GV----------------
SDR9C6 H. sapiens           YSAGWDAKFFFIP---LSYLPTSLADYILTRSWPKPAQ----AV----------------
SDR9C6 P. paniscus          YSAGWDAKFFFIP---LSYLPTSLADYILTRSWPKPAQ----AV----------------
SDR9C6 M. musculus          YSAGWDAKFFFIP---LSYLPASLVDYLLAISRGKPAQ----AA----------------
SDR9C6 S. harrisii          YSAGWDAQFLYIP---MSYLPTRLLDYLLSKNLQKPAH----AI----------------
SDR9C6 A. carolinensis      YSAGWDAQFFFIP---LSYLPTALMDLILTWSSPKPAH----AI----------------
SDR9C6 X. tropicalis        YSVGWDCKLYHLP---LSYLPTAVSDYVLCRSAPKPAH----SAK---------------
SDR9C7 H. sapiens           YNPGLDAKLLYIP---LAKLPTPVTDFILSRYLPRPAD----SV----------------
SDR9C7 P. troglodytes       YNPGLDAKLLYIP---LAKLPTPVTDFILSRYLPRPAD----SV----------------
SDR9C7 M. musculus          YNPGLDVKFLYLT---LAKLPTPVTDFILSRYLPRPAD----SV----------------
SDR9C7 S. harrisii          YNPGLDAKLLYLP---LAKFPTALTDFILSRYLPKPAD----SVL---------------
SDR9C7 X. tropicalis        YSPGWDAKLFYIP---LSYLPTVLSDYVASRFAPRLSQ----GEK---------------
SDR9C8 H. sapiens           YSAGWDAKLLYLP---MSYMPTFLVDAIMYWVSPSPAK----AL----------------
SDR9C8 P. troglodytes       YSAGWDAKLLYLP---MSYMPTFLVDAIMYWVSPSPAK----AL----------------
SDR9C8 M. musculus          YSAGWDAKLFYLP---LSYLPTFLVDALLYWTSLKPEK----AL----------------
SDR9C8 M. domestica         YSAGWDAKLFYLP---LSYLPTALADFLIMWNYPKPSP----TR----------------
SDR9C8 P. humilis           YSAGWDAKLLYLP---ISYLPSALSDALFSLFYPKSVG----KA----------------
SDR9C8 A. carolinensis      YSAGWDAKLFYIP---MSYMPVLVHDLVFGWSYPKPAQ----TS----------------
SDR9C8 X. tropicalis        YSAGWDAKLFFIP---LSYLPTVCIDFLLNRSRAKAAH----SI----------------
SDR9C8 P. reticulata        YSPGWDAKFLWLP---ISYMPTWISDGFFLRYSPKPKA----SVI---------------
                            *                    *

SDR9C1 H. sapiens           ------------------
SDR9C1 P. paniscus          ------------------
SDR9C1 M. musculus          ------------------
SDR9C1 M. domestica         ------------------
SDR9C1 C. livia             ------------------
SDR9C1 C. picta             ------------------
SDR9C1 X. tropicalis        ------------------
SDR9C1 D. rerio             ------------------
SDR9C2 H. sapiens           ------------------
SDR9C2 P. troglodytes       ------------------
SDR9C2 M. musculus          ------------------
SDR9C2 M. domestica         ------------------
SDR9C2 G. gallus            ------------------
SDR9C2 A. mississippiensis  ------------------
SDR9C2 X. tropicalis        ------------------
SDR9C2 D. rerio             ------------------
SDR9C3 H. sapiens           PNLSPGPSPA--VAR---
SDR9C3 P. troglodytes       PNLSPGPSPA--VAR---
SDR9C3 M. musculus          ------------------
SDR9C3 C. picta             ------------------
SDR9C3 X. tropicalis        ------------------
SDR9C3 D. rerio             ENMNDSSSNNSNFTKCID
SDR9C4 H. sapiens           ------------------
SDR9C4 P. troglodytes       ------------------
SDR9C4 M. musculus          ------------------
SDR9C4 M. domestica         ------------------
SDR9C4 G. gallus            ------------------
SDR9C4 C. mydas             ------------------
SDR9C4 X. tropicalis        ------------------
SDR9C4 D. rerio             ------------------
SDR9C5 H. sapiens           ------------------
SDR9C5 P. troglodytes       ------------------
SDR9C5 M. musculus          ------------------
SDR9C5 S. harrisii          ------------------
```

```
SDR9C5 G. gallus            ------------------
SDR9C5 A. carolinensis      ------------------
SDR9C5 X. tropicalis        ------------------
SDR9C5 D. rerio             ------------------
SDR9C6 H. sapiens           ------------------
SDR9C6 P. paniscus          ------------------
SDR9C6 M. musculus          ------------------
SDR9C6 S. harrisii          ------------------
SDR9C6 A. carolinensis      ------------------
SDR9C6 X. tropicalis        ------------------
SDR9C7 H. sapiens           ------------------
SDR9C7 P. troglodytes       ------------------
SDR9C7 M. musculus          ------------------
SDR9C7 S. harrisii          ------------------
SDR9C7 X. tropicalis        ------------------
SDR9C8 H. sapiens           ------------------
SDR9C8 P. troglodytes       ------------------
SDR9C8 M. musculus          ------------------
SDR9C8 M. domestica         ------------------
SDR9C8 P. humilis           ------------------
SDR9C8 A. carolinensis      ------------------
SDR9C8 X. tropicalis        ------------------
SDR9C8 P. reticulata        ------------------
```

Figure S14. Alignment of the vertebrate SDR9C family variants. For further details see Fig. S13 and Table S14.

* symbol marks the position of identical amino acid residues of the aligned SDR protein sequences.

```
                                          ×  ×  ×
SDR10E1 H. sapiens       MVSIPEYYEGKNVLLTGATGFLGKVLLEKLLRSCPKVNSVYVLVRQKAGQTPQERVEEVL
SDR10E1 P. troglodytes   MVSIPEYYEGKNVLLTGATGFLGKVLLEKLLRSCPKVNSVYVLVRQKAGQTPQERVEEVL
SDR10E1 M. musculus      MVSIPEYYEGKNILLTGATGFLGKVLLEKLLRSCPRVNSVYVLVRQKAGQTPQERVEEIL
SDR10E1 S. harrisii      MVSIPEYYEGKNVLLTGATGFLGKVLLEKLLRSCPKVNSVYVLVRQKAGQTPQERVEEVI
SDR10E1 G. gallus        MVSIPEYYEGKNVLLTGATGFMGKVLLEKLLRSCPKVKAVYVLVRPKAGQTPEARIEEIT
SDR10E1 C. mydas         MVSIPEYYEGKNVLLTGATGFMGKVLLEKLLRSCPKVKAVYILVRHKAGQTPQERMEEMI
SDR10E1 X. tropicalis    MISIPEFYRGKNVLITGATGFMGKVLLEKLLRSCPNVKAVYVLVRPKASQKPRERVAEMM
SDR10E1 D. rerio         MVTIPEYYVGKNVLITGATGFMGKVLLEKLLRSCPGVKAAYVLVRPKAGQAPDARIADMI
SDR10E2 H. sapiens       MSTIAAFYGGKSILITGATGFLGKVLMEKLFRTSPDLKVIYILVRPKAGQTLQQRVFQIL
SDR10E2 P. troglodytes   MSTIAAFYGGKSILITGATGFLGKVLMEKLFRTSPDLKVIYILVRPKAGQTLQQRVFQIL
SDR10E2 M. musculus      MSMIAAFYSNKSILITGATGFLGKVLMEKLFRTSPHLKVIYILVRPKSGQTLQERVFQIL
SDR10E2 M. domestica     MSAIAAYYGGKSILITGATGFMGKVLVEKLFRTSPDLKVIYILVRPKAGQSLQQRVSQMI
SDR10E2 G. gallus        MSSVSAYYNGKTVLITGATGFMGKVLVEKLLRSSPDVKAVYILVRPKAGQSMQERVANML
SDR10E2 A. carolinensis  MSSVATYYNEKSVLVTGATGFMGKVLVEKLLRSSPDVKAIYILVRPKAGQLMQNRVEHMV
SDR10E2 E. lucius        MASIADYYAGKSVLITGATGFMGKVLVEKLLRSCPHVRALYLLVRPKAGQSMQERVSDMM
                         *      *  *  * ****** **** *** *  *     * *** *  *    *

SDR10E1 H. sapiens       SGklFDRLRDENPDFREKIIAINSELTQPKLALSEEDKEVIIDSTNIIFHCAATVRFNEN  1p.0
SDR10E1 P. troglodytes   SGklFDRLRDENPDFREKIIAINSELTQPKLALSEEDKEVIIDSTNIIFHCAATVRFNEN  1p.0
SDR10E1 M. musculus      SSklFDRLRDENPDFREKIIAINSELTQPKLALSEEDKEIIIDSTNVIFHCAATVRFNEN  1p.0
SDR10E1 S. harrisii      SGklFDRLRDENPQFRQKIIAISSELTQPKLALTEEDQEIILDSTNIIFHCAATVRFNEN  1p.0
SDR10E1 G. gallus        SCklFDRLREEQPDFKEKIIVITSELTQPELDLSNPVKEKLIECINIIFHCAATVRFNET  1p.0
SDR10E1 C. mydas         SCklFDRLRDEQPGFKEKIIAVTSELTQSELDLSEEDKEKLIDCINIVFHCAATVRFNET  1p.0
SDR10E1 X. tropicalis    SCklFDRLRDEQPECAQKVIAISSELTQPELDLSKEDQDMLIDCIDIVFHCAATVRFNES  1p.0
SDR10E1 D. rerio         NCklFDRLREDQPDFAGKIVAINSDLTQPNLDLSAEDQETLTGCINVVFHCAATIRFNEP  1p.0
SDR10E2 H. sapiens       DSklFEKVKEVCPNVHEKIRAIYADLNQNDFAISKEDMQELLSCTNIIFHCAATVRFDDT  1p.0
SDR10E2 P. troglodytes   DSklFEKVKEVCPNVHEKIRAIYADLNQNDFAISKEDMQELLSCTNIIFHCAATVRFDDT  1p.0
SDR10E2 M. musculus      NSklFEKVKEVCPNVHEKIRPISADLNQRDFAISKEDVQELLSCTNIIFHCAATVRFDAH  1p.0
SDR10E2 M. domestica     NCklFEKAKEICPNIFEKIRPIYADLTKPDLGISKEDLEELLDHTNIIFHCAATVRFDDS  1p.0
SDR10E2 G. gallus        KCkvFDRVREDCPNFHEKIKPINAELTQPKLAISAEDEEELLTRVNIVFHCAATVRFDEP  1p.0
SDR10E2 A. carolinensis  KCklFDRVREECPNFHEKIKPISAELTHPNLAINPEDTAELLSEVNIVFHCAATVRFDEP  1p.0
SDR10E2 E. lucius        KCklFDRVREYNPDFHQKIVPISSELIQPGLAIGPEDKETLTSCINIIFHCAATIRFDEP  1p.0
                           * *       *    *       *                      ****** **

SDR10E1 H. sapiens       LrdAVQLNVIATRQLILLAQQMKNLEVFMHVSTAYAYCNRKHIDEVVYPPPVDPKKLIDS  2p.1
SDR10E1 P. troglodytes   LrdAVQLNVIATRQLILLAQQMKNLEVFMHVSTAYAYCNRKHIDEVVYPPPVDPKKLIDS  2p.1
SDR10E1 M. musculus      LrdAVQLNVIATRQLILLAQQMKNLEVFMHVSTAYAYCNRKHIDEVVYPPPVDPKKLIDS  2p.1
SDR10E1 S. harrisii      LrdAVQLNVIATRQLILLAQQMKNLEVFMHVSTAYAYCNRKHIDEVVYPPPVDPKKLIDS  2p.1
SDR10E1 G. gallus        LrdAVQLNVLSTKQLLSLAQQMTNLEVFMHVSTAYAYCNRKHIEEVVYPPPVDPKKLMDS  2p.1
SDR10E1 C. mydas         LrdAVQLNVIATQQLIILAQRMKNLEVFMHVSTAYAYCNRKHIEEIIYPPPVDPKKLIGS  2p.1
SDR10E1 X. tropicalis    LrdAMQLNVIATRQLLYLAQKIKKLEVFIHVSTAYANCNRKQIEEMVYPPPVDPKKLIES  2p.1
SDR10E1 D. rerio         LkdAMQLNVLATQKMVSLAHRMKHLEVFIHVSTAYANCDRELIEEVVYPPPVDYRKLIDT  2p.1
SDR10E2 H. sapiens       LrhAVQLNVTATRQLLLMASQMPKLEAFIHISTAYSNCNLKHIDEVIYPCPVEPKKIIDS  2p.1
SDR10E2 P. troglodytes   LrhAVQLNVTATRQLLLMASQMPKLEAFIHISTAYSNCNLKHIDEVIYPCPVEPKKIIDS  2p.1
SDR10E2 M. musculus      LreAVQLNVTATQQLLLMASQMPKLEAFIHISTAFSNCNLSHIDEVIYPCPVEPRKIIDS  2p.1
SDR10E2 M. domestica     LrhALQLNVIATQQLLLMASQMPKLEVFIHFSTAYANCNLKHIDEVIYPCSVEPKKLIDS  2p.1
SDR10E2 G. gallus        LkhALQLNAMGTQRLLELAQQMQKLEAFIHISTAYANCVRKCIDEIIYPPPAEPKKLFDL  2p.1
SDR10E2 A. carolinensis  LkhALLLNVRGTQQLLALARQMKNLETFIHVSTAYANCNQRYIDEIIYPPPMEPKKLLDL  2p.1
SDR10E2 E. lucius        LkhALQLNVMATQQLISLAQQMPHLQAFIHISTAYANCNRRHIDDIIYPPPVEPKKLIDS
                         *  *  **   *      *     *  * * ***   *    *    **      *

                                               +   +
SDR10E1 H. sapiens       LewMDDGLVNDITPKLIGDRPNTYIYTKALAEYVVQQEGAKLNVAIVRPSIVGASWKEPF  3p.1
SDR10E1 P. troglodytes   LewMDDGLVNDITPKLIGDRPNTYIYTKALAEYVVQQEGAKLNVAIVRPSIVGASWKEPF  3p.1
SDR10E1 M. musculus      LewMDDGLVNDITPKLIGDRPNTYIYTKALAEYVVQQEGAKLNVAIVRPSIVGASWKEPF  3p.1
```

```
SDR10E1 S. harrisii      LewMDDGLVNDITPKLIGDRPNTYIYTKALAEYVVQQEGAKLNVAIVRPSIVGASWKEPF  3p.1
SDR10E1 G. gallus        LewMDDSLVNDITPKLIGDRPNTYTYTKALAEYVVQQEGARLNTAIIRPSIVGASWKEPF  3p.1
SDR10E1 C. mydas         LewMDDGLVKDITPKLIGDRPNTYTYTKALAEYIVQQEGAKLNIAIIRPSIVGASWKEPF  3p.1
SDR10E1 X. tropicalis    LewMDDGLVNDITPKLIGDRPNTYTYTKALAEYVVQQEGSKLNIAIVRPSIVGASWKEPF  3p.1
SDR10E1 D. rerio         LewMDDKLVSLMTPRLLGERPNTYTYTKALAEQLVQQECGNLNIAIIRPSIVGASWKEPF  3p.1
SDR10E2 H. sapiens       LewLDDAIIDEITPKLIRDWPNIYTYTKALGEMVVQQESRNLNIAIIRPSIVGATWQEPF  3p.1
SDR10E2 P. troglodytes   LewLDDAIIDEITPKLIRDWPNIYTYTKALGEMVVQQESRNLSIAIIRPSIVGATWQEPF  3p.1
SDR10E2 M. musculus      MewLDDSIIEEITPKLIGDRPNTYTYTKALGEIVVQQESGNLNVAIVRPSIVGATWQEPF  3p.1
SDR10E2 M. domestica     VewLDDSIIEEITPKLIENRPNTYTYTKALGEMVVYQESGNLNIAIIRPSIVGASWQEPF  3p.1
SDR10E2 G. gallus        VewLDESIIQDITPKLLGDWPNTYTYTKALSEYLIQQEKGNLNIAIIRPSIVGASWHEPF  3p.1
SDR10E2 A. carolinensis  VewMDEFLIEEITPKLIGDWPNTYTFTKALAEYLLQQEKGNINVAIVRPSIVGASWHEPF  3p.1
SDR10E2 E. lucius        LewMEDSIVRDITPRLIGDHPNTYTYTKALAECVVQKESSKLSIAIIRPSIVGASWQEPF  3p.1
                          **         ** *    ** *  **** *     *      ** ******* * ***

SDR10E1 H. sapiens       pgWIDNFNGPSGLFIaaGKGILRTIRASNNALADLVPVDVVVNMSLAAAWYSGVN---rp  4p.0 5p.0 6p.1
SDR10E1 P. troglodytes   pgWIDNFNGPSGLFIaaGKGILRTIRASNNALADLVPVDVVVNMSLAAAWYSGVN---rp  4p.0 5p.0 6p.1
SDR10E1 M. musculus      pgWIDNFNGPSGLFIaaGKGILRTMRASNNALADLVPVDVVVNTSLAAAWYSGVN---rp  4p.0 5p.0 6p.1
SDR10E1 S. harrisii      pgWIDNFNGPSGLFIaaGKGILRTMRASNNALADLVPVDVVVNTSLAAAWYSGVN---rp  4p.0 5p.0 6p.1
SDR10E1 G. gallus        pgWIDNFNGPSGLFIaaGKGILRTMRASNGAVADLVPVDVVVNMTLAAAWYSGVN---rp  4p.0 5p.0 6p.1
SDR10E1 C. mydas         pgWIDNFNGPSGLFIaaGKGILRTMRASNSAVADLVPVDVVVNTTLAAAWYSGVN---rp  4p.0 5p.0 6p.1
SDR10E1 X. tropicalis    pgWIDNFNGPSGLFIaaGKGILRTMRASNNAVADLIPVDVVVNTTLAAAWYSGVN---rp  4p.0 5p.0 6p.1
SDR10E1 D. rerio         pgWIDNFNGPSGIFIaaGKGILRTMRASNNAVADLVPVDVVINTTLAAAWYSGSQRHArp  4p.0 5p.0 6p.1
SDR10E2 H. sapiens       pgWVDNINGPNGIIIatGKGFLRAIKATPMAVADVIPVDTVVNLMLAVGWYTAVH---rp  4p.0 5p.0 6p.1
SDR10E2 P. troglodytes   pgWVDNINGPNGIIIatGKGFLRAIKATPMAVADVIPVDTVVNLMLAVGWYTAVH---rp  4p.0 5p.0 6p.1
SDR10E2 M. musculus      pgWVDNLNGPSGLIIatGKGFLRSIKATPMAVADVIPVDTVVNLTIAVGWYTAVH---rp  4p.0 5p.0 6p.1
SDR10E2 M. domestica     pgWVDNLNGPSGLIIatGKGFLRAIKATPRAVADVVPVDLVINLTLAVGWHTAVH---rp  4p.0 5p.0 6p.1
SDR10E2 G. gallus        pgWIDSFNGTSGIFVaaGKGILRTVIANNEAVADMIPVDVVINLTLAAGWYTAVH---rp  4p.0 5p.0 6p.1
SDR10E2 A. carolinensis  pgWIDNFNGTSGIFIaaGKGIIRTVKCNVEAVADIVPVDVAINLILATGWYTAVH---rp  4p.0 5p.0 6p.1
SDR10E2 E. lucius        pgWIDNFNGPSGVFIaaGKGILRTMRANNDAVADLIPVDVVINLTLAAGWYTAVH---rp  4p.0 5p.0 6p.1
                         *** *  **  *   * ***  *       * **  ***   *   *  *        **

SDR10E1 H. sapiens       RNIMVYNCTTGSTNPFHWGEveYHVISTFKRNPLEQAFRRPNVNLTSNHLLYHYWIAVSH  7p.2
SDR10E1 P. troglodytes   RNIMVYNCTTGSTNPFHWGEveYHVISTFKRNPLEQAFRRPNVNLTSNHLLYHYWIAVSH  7p.2
SDR10E1 M. musculus      RNIMVYNCTTGSTNPFHWGEveYHVISTFKRNPLEQAFRRPNVNLTSNHLLYHYWIAVSH  7p.2
SDR10E1 S. harrisii      RNIMVYNCTTGSTNPFHWGEveYHVISTFKRNPLEQAFRRPNVNLTSNHLLYHYWIAVSH  7p.2
SDR10E1 G. gallus        RNIMVYNCTTGGTNPFHWSEveYHVISTFKRNPLEQAFRRPNVNLTSNHLLYHYWIAVSH  7p.2
SDR10E1 C. mydas         RNIMVYNCTTGGTNPFHWGEveYHVISTFKRNPLEQAFRRPNVNLTSNHLLYHYWIAVSH  7p.2
SDR10E1 X. tropicalis    RNILVYNCTTGGTNPFHWGEveYHVISTFKRNPLEQAFRRPNVNLTSNHLLYHYWIAVSH  7p.2
SDR10E1 D. rerio         RSLLVYNCTTGGINPFHWGEveYHVISTFKRNPLEQAFRRPNVNLTTNHLINQYWIAVSH  7p.2
SDR10E2 H. sapiens       KSTLVYHITSGNMNPCNWHKmgVQVLATFEKIPFERPFRRPNANFTSNSFTSQYWNAVSH  7p.2
SDR10E2 P. troglodytes   KSTLVYHITSGNMNPCNWHKmgVQVLATFEKIPFERPFRRPNANFTSNSFTSQYWNAVSH  7p.2
SDR10E2 M. musculus      KSTLIYHSTSGNLNPCNWYKmgLQVLATIEKIPFESAFRRPNADFTTSNFTTHYWNTVSH  7p.2
SDR10E2 M. domestica     KSILIYHCTSGSLNPCSWIDlgFQVLATFEKAPLEKAFRRPKADFTTNSFSTYLWNTISH  7p.2
SDR10E2 G. gallus        KNMLVYNCTTGGINPFFWGEmeQYVMSTFKRNPLEQAFRTPNAHLTSNYLINQYWITVSH  7p.2
SDR10E2 A. carolinensis  KSMLIYNCTTGGMNPFCWGEmeHHVISTYKRNPLEKAFRIPKANMTSNYLMHQYWTAVSH  7p.2
SDR10E2 E. lucius        KAALVYNCTTGGINPFHWGEieHHVMSSFKRNPLEQAFRRPNANITSNYLMNQYWILVSH  7p.2
                              *  * *  **  *      *       * *  ** *    *        *   **

SDR10E1 H. sapiens       KAPAFLYDIYLRMTGRSPrmMKTITRLHKAMVFLEYFTSNSWVWNTENVNMLMNQLNPED  8p.1
SDR10E1 P. troglodytes   KAPAFLYDIYLRMTGRSPrmMKTITRLHKAMVFLEYFTSNSWVWNTDNVNMLMNQLNPED  8p.1
SDR10E1 M. musculus      KAPAFLYDIYLRMTGRSPrmMKTITRLHKAMVFLEYFTSNSWVWNTDNVNMLMNQLNPED  8p.1
SDR10E1 S. harrisii      KAPAFLYDIYLRITGRSPrmMKTITRLHKAMVFLEYFTSNSWVWNTDNVNMLMNQLNPED  8p.1
SDR10E1 G. gallus        KAPAFLYDIYLRITGRSPrmMKTITRLHKAMVFLEYFTSNSWIWNTENMTMLMNQLSPED  8p.1
SDR10E1 C. mydas         KAPAFLYDIYLRITGRSPrmMKTITRLHKAMMLIEYFTSNSWIWNTENVNMLMNQLSPED  8p.1
SDR10E1 X. tropicalis    KAPAFLYDVYLRITGRSPrmMKTITRLHKAMMLLEYFTSNSWVWNNENTNMLMSQLSPDD  8p.1
SDR10E1 D. rerio         KAPAFLYDLFLRMSGREPrmMKTITRLHKAMMVLEYFTSHSWVWNTDNVTMLMNQMGAED  8p.1
```

```
SDR10E2 H. sapiens       RAPAIIYDCYLRLTGRKPrmTKLMNRLLRTVSMLEYFINRSWEWSTYNTEMLMSELSPED  8p.1
SDR10E2 P. troglodytes   RAPAIIYDCYLRLTGRKPrmTKLMNRLLRTVSMLEYFINRSWEWSTYNTEMLMSELSPED  8p.1
SDR10E2 M. musculus      RVPAIIYDFYLRLTGRKPrmLKLMNRLLKTISMLEYFINHSWEWSTNNTEMLLSELSPED  8p.1
SDR10E2 M. domestica     MAPAVIYDFYLRLTGRKPrmAKLMNRMLKTISMLEYFINHSWEWSTYNTEMLMSQLSNED  8p.1
SDR10E2 G. gallus        KAPAILYDLYMRLTGRKPrmMKIINRLHKSMMLLQYFSTQSWDWSSDNMNMLMGQLNTED  8p.1
SDR10E2 A. carolinensis  KAPAFLYDLYLRLTGRKPrmMKLFSRLHKSMTFFEYFTSRTWEWSSDNMNMLMNQLSPKD  8p.1
SDR10E2 E. lucius        RFPALLYDLYLRLSGQKPqmMRIFNRLHKAIGLLEYFSSQDWEWNSENMNMLMSHLSPED  8p.1
                           **  **   *  *  * *     *         **    * *   *  **       *

SDR10E1 H. sapiens       KktFNIDVRQLHWAEYIENYCLGTKKYVLNEEMSGLPAARKHLNklRNIRYGFNTILVIL  9p.0 10p.1
SDR10E1 P. troglodytes   KktFNIDVRQLHWAEYIENYCLGTKKYVLNEEMSGLPAARKHLNklRNIRYGFNTILVIL  9p.0 10p.1
SDR10E1 M. musculus      KktFNIDVRQLHWAEYIENYCMGTKKYVLNEEMSGLPAARKHLNklRNIRYGFNTILVIL  9p.0 10p.1
SDR10E1 S. harrisii      KktFNIDVRQLHWAEYIENYCMGTKKYVLNEEMSGLPAARKHLNklRNIRYGFNTILVIL  9p.0 10p.1
SDR10E1 G. gallus        KktFNFDVRQLHWAEYMENYCMGTKKYVLNEEMSGLPAARKHLNklRNIRYGFNTILVIL  9p.0 10p.1
SDR10E1 C. mydas         KkvFNFDVRQLHWAEYMENYCMGTKKYVLNEEMSGLPAARKHLKklRNIRYGFNTVLVIL  9p.0 10p.1
SDR10E1 X. tropicalis    KkaFNFDVRQLHWAEYMENYCMGTKKYVLNEEMSGLPAARKHLNklRNIRYGFNTILVVL  9p.0 10p.1
SDR10E1 D. rerio         KkvFNFDVRQLHWAEYMENYCMGTKKYVLNEELSGLPAARKHLNklRNIRYTFNTVLVVL  9p.0 10p.1
SDR10E2 H. sapiens       QrvFNFDVRQLNWLEYIENYVLGVKKYLLKEDMAGIPKAKQRLKrlRNIHYLFNTALFLI  9p.0 10p.1
SDR10E2 P. troglodytes   QrvFNFDVRQLNWLEYIENYVLGVKKYLLKEDMAGIPEAKQRLKrlRNIHYLFNTALFLI  9p.0 10p.1
SDR10E2 M. musculus      QrvFNFDVRQLNWLEYIENYVLGVKKYLLKEDLAGIPKAKQHLRrlRNIHYLFNTALFLI  9p.0 10p.1
SDR10E2 M. domestica     QklFNFDVRHLNWLEYTENYCIGVKKYLLKEDMAGIPAAKKHLRmlRNIYYLFNTVVFLI  9p.0 10p.1
SDR10E2 G. gallus        KklYNFDVRQLHWSEYIESYCLGAKKYLLNEDMSGIPAAKQHLRklRNIRYAFNTTLLVI  9p.0 10p.1
SDR10E2 A. carolinensis  KklFCFDVRQLHWSEYIENYCLGTKKYLLNEDMAGIPAAKQHLRklRNIQYALNTIFLVI  9p.0 10p.1
SDR10E2 E. lucius        RktFNFDVRQLNWPEYIENYCIGTKKYVLNEDMSDIPAARQHLRklRNIRYTFNTLLLVF  9p.0 10p.1
                               *** * * ** * *  * *** * *     * *   *  **** *  **

SDR10E1 H. sapiens       IWRIFIARSQMARNIWYFVVSLCYKFLSYFRASSTMRY
SDR10E1 P. troglodytes   IWRIFIARSQMARNIWYFVVSLCYKFLSYFRASSTMRY
SDR10E1 M. musculus      IWRIFIARSQMARNIWYFVVSLCYKFLSYFRASSTMRY
SDR10E1 S. harrisii      IWRIFIARSQMARNIWYFVVSLCYKFLSYFRASSTMRY
SDR10E1 G. gallus        IWRIFIARSQMARNIWYFVVSLCYKFLSYFRASSTMRY
SDR10E1 C. mydas         IWRIFIARSQMARNIWYFVVSLCYKFLSYFRASSTMRY
SDR10E1 X. tropicalis    IWRVFIARSQMARNIWYFVVSMCFKFLSYFRASSTMRY
SDR10E1 D. rerio         IWRIFIARSQMARNIWYFVVSLCFKFLSYFRASSTMRY
SDR10E2 H. sapiens       AWRLLIARSQMARNVWFFIVSFCYKFLSYFRASSTLKV
SDR10E2 P. troglodytes   AWRLLIARSQMARNVWFFIVSFCYKFLSYFRASSTLKV
SDR10E2 M. musculus      IWRLLIARSQMARNVWFFIVSFCYKFISYFRASSTLKV
SDR10E2 M. domestica     IWRFFIARSQMARNIWYFVVSLCYKFLSYFRASSTFRH
SDR10E2 G. gallus        IWRIFIARSQMARNIWYFVVSLCYKFLSYFRASSTLRH
SDR10E2 A. carolinensis  IWRIFIARSQMAQNIWYFVVNLCYKFLSYFRASSTLRH
SDR10E2 E. lucius        IWRVFIARSQMARNIWYFVVSLCFKFLSYFRASSTLTN
                          **  ******* * * * *  * ** ********
```

Figure S15. Alignment of the vertebrate SDR10E family variants. For further details see Fig. S13.

* symbol marks the position of identical amino acid residues of the aligned SDR protein sequences.

```
                                        ×  ×  ×
SDR11E1 H. sapiens       ----MAMTGWSCLVTGAGGFLGQRIIRLLVK-EKELKEIRVLDKAFGPELREEFskLQNK  1p.2
SDR11E1 P. troglodytes   ----MAMTGWSCLVTGAGGFLGQRIIRLLVK-EKELKEIRVLDKAFRPELREEFskLQNK  1p.2
SDR11E1 M. musculus      ------MAGWSCLVTGAGGFVGQRIIKMLVQ-EKELQEVRALDKVFRPETKEEFskLQTK  1p.2
SDR11E1 M. domestica     ---MLPTDRWSCLVTGAGGFLGQRIVRLLLEEEKELEEIRVLDKFFSPQLLLEFseLKHK  1p.2
SDR11E1 N. nippon        ----MSLAGVSCLVTGAGGFLGQRIVGLLLEEKEALAEIRLLDKAFSSEALGRFgkFQGK  1p.2
SDR11E1 T. sirtalis      ----MSLGGIRCLVTGAGGFLGQRIVCQLLTEKESLAEVRLLDKTISAEALQDFkkVRSN  1p.2
SDR11E1 L. crocea        ----MSLKGDVCVVTGACGFLGKRLVRLLLE-EENVAEIRLMDKHIQPQLLHSLedCRGD  1p.2
SDR11E2 H. sapiens       -------MGWSCLVTGAGGLLGQRIVRLLVE-EKELKEIRALDKAFRPELREEFskLQNR  1p.2
SDR11E2 P. troglodytes   -------MGWSCLVTGAGGLLGQRIVRLLVE-EKELKEIRALDKAFRPELREEFskLQNK  1p.2
SDR11E2 M. musculus      ------MPGWSCLVTGAGGFLGQRIIQLLVQ-EEDLEEIRVLDKVFRPETRKEFfnLETS  1p.2
SDR11E2 S. harrisii      ---MLQMNKWSCLVTGAGGFLGQRIVHLLLEEEQELEEIRLLDKVFSPQLLLEFseLKHK  1p.2
SDR11E2 G. gallus        ----MSLAGVSCLVTGAGGFLGQRIVRLLLEEDEALAEIRLLDKAFSREALWSFgkFQGK  1p.2
SDR11E2 G. japonicus     ----MSLVGVKCLVTGAGGFLGQRIVCQLLEEGEKLAEVRALDKAFSSETLQRFadLKST  1p.2
SDR11E2 D. rerio         ----MALSGEVCVVTGACGFLGEKLVRLLLE-EENLSEIRLLDRNIRSELIQTLedGRGE  1p.2
SDR11E3 H. sapiens       MADSAQAQKLVYLVTGGCGFLGEHVVRMLLQREPRLGELRVFDQHLGPWLEELKtgP---  1p.2
SDR11E3 P. troglodytes   MADSAQAQKLVYLVTGGCGFLGEHVVRMLLQREPRLGELRVFDRHLGPWLEELKtgP---  1p.2
SDR11E3 M. musculus      MADSAQVPTLVYLVTGGCGFLGEHIVRMLLEREPRLRELRVFDLHLSSWLEELKagP---  1p.2
SDR11E3 S. harrisii      --MTDGGRNLVYLVTGGCGFLGEHMIRTLLEMEPRLKELRVFDLHLGPWLEALNpgS---  1p.2
SDR11E3 Z. albicollis    -----MDGAWVYLVTGGCGFVGERIVELL-SQQDYIKEVRVFDSVAREEVEKFStaP---  1p.2
SDR11E3 A. carolinensis  --MGSTSRNQIYLVTGGCGFLGKHLVQMLLEQEPDLAEVRVFDLHLDESMRHLNrv----  1p.2
SDR11E3 X. tropicalis    ---MTAGSGQVYVVTGGCGFLGSHLVRMLLEHEKNISEIRVFDLHLDESLRSLSnnR---  1p.2
SDR11E3 D. rerio         -MSNNNKSKLTYVITGGCGFLGQHLLRVLLEKKKNVKEIRLFDKNVFPSLQSESteD---  1p.2
                                       **  *  *      *        * *  *

SDR11E1 H. sapiens        TKLTVLEGDILDEPFLKRACQDVSVIIHTACIIDVFGVTHRESIMNVNVkgTQLLLEACV  2p.2
SDR11E1 P. troglodytes    TKLTVLEGDILDEPFLKRACQDVSVIIHTACIIDVFGVTHRESIMNVNVkgTQLLLEACV  2p.2
SDR11E1 M. musculus       TKVTVLEGDILDAQCLRRACQGISVVIHTAAVIDVTGVIPRQTILDVNLkgTQNLLEACV  2p.2
SDR11E1 M. domestica      TKVTVLEGDILDEQFVHKACQGISAVIHTACIIDTGICIKKEVIMkvNLkgTQLLLEACI  2p.2
SDR11E1 N. nippon         TEVKILEGDIRDATFLHRACQGVSLVIHTASIIDTLGLIDKQLLWEVNVtgTQLLLEACV  2p.2
SDR11E1 T. sirtalis       TLLTVLQGDIRDVVFLETAVQGVSLVIHAACIIDPRGFIDRKILWDVNVrgTQLLLEACL  2p.2
SDR11E1 L. crocea         TKLSVFEGDIRDGDFLKKVCRGASIVFHIASIIDVNDSVEYSEIYGVNVkgTQLLLEACI  2p.2
SDR11E2 H. sapiens        TKLTVLEGDILDEPFLKRACQDVSVVIHTACIIDVFGVTHRESIMNVNVkgTQLLLEACV  2p.2
SDR11E2 P. troglodytes    TKLTVLEGDILDEPFLKRACQDISVIIHTACIIDVFGVTHRESIMNVNVkgTQLLLEACV  2p.2
SDR11E2 M. musculus       IKVTVLEGDILDTQYLRRACQGISVVIHTAAIIDVTGVIPRQTILDVNLkgTQNLLEACI  2p.2
SDR11E2 S. harrisii       TKVTLLQGDILDKEFLHKACQGVTAVIHSACIIDTGLWRRKETIMNVNIkgTQFLLEACI  2p.2
SDR11E2 G. gallus         TEVKILEGDIRDVEFLHRACQGVSLVIHTASIIDTLGLVEKQLLWEVNVtgTQMLLEACA  2p.2
SDR11E2 G. japonicus      TPLTIMKGDIRDMSFLRKAVQGISLVIHSACVIDILGLVDKPVLWDINVtgTRLLLEMCL  2p.2
SDR11E2 D. rerio          TKVSVIEGDIRDRELLRRACKGATLVFHTASLIDYNGAVEYSELHAVNVkaTRLLLETCI  2p.2
SDR11E3 H. sapiens        VRVTAIQGDVTQAHEVAAAVAGAHVVIHTAGLVDVFGRASPKTIHEVNVqgTRNVIEACV  2p.2
SDR11E3 P. troglodytes    VRVTAIQGDVTQAHEVAAAVAGAHVVIHTAGLVDVFGRASPETIHEVNVqgTRNVIEACV  2p.2
SDR11E3 M. musculus       VQVTAIQGDVTQAHEVAAAMSGSHVVIHTAGLVDVFGKASPKTIHKVNVqgTQNVIDACV  2p.2
SDR11E3 S. harrisii       VQVTPIQGDVTRAEDVVAAVAGTDVVIHMAGLVDVWGQNDPEVIHRVNVqgTQNVIEACV  2p.2
SDR11E3 S. harrisii       TRVTVMRGDIRDPNALLAAMRGVHVVLHTAAVVDYRGTVPFWEMRAVNVggTENVVRACC  2p.2
SDR11E3 A. carolinensis   ---TLIQGDITNPEDVRTAVQGANVVIHTASLVDVWGRFSPEKINAVNVcgTQNVIEACV  2p.2
SDR11E3 X. tropicalis     VRVRLISGDISHLDDVREALHGSHLVIHTASLVDVWGRVPASKINEVNVtgTENVLQACK  2p.2
SDR11E3 D. rerio          VKVVIIQGDITKYEDVRNAFLGADLVFHAASLVDVWYKIPEKVIFAVNVqgTENAIKACV  2p.2
                                 **                  * *    *             *   *      *
                                                                   +   +
SDR11E1 H. sapiens        QASVPVFIYTSSIEVAGPNSYKEIIQNGHEEEPLENTWPAPYPHSKKLAEKAVLAANGWN
SDR11E1 P. troglodytes    QASVPVFIYTSSIEVAGPNSYKEIIQNGHEEEPLENTWPAPYPHSKKLAEKAVLAANGWN
SDR11E1 M. musculus       QASVPAFIFCSSVDVAGPNSYKKIVLNGHEEQNHESTWSDPYPYSKKMAEKAVLAANGSM
SDR11E1 M. domestica      SANVPVFLHTSTLEVAGPNSFIKHVRNGHEGEPLETKWSNAYPYSKKLAEKTILAANGQL
SDR11E1 N. nippon         RCNVQHFIYTSTIEVTGPNCKGDPVFNGDEDTAYESVSKSPYAQSKRLAEDSVLKADGQV
SDR11E1 T. sirtalis       RNDVQYFIYTSSLEVTGPNNRGDPIYDGDEDTIYQMTQGQPYAETKREAEKCVLQLDGLP
SDR11E1 L. crocea         QENVVSFIYTSTVEVMGPNLKGEPIVNGNEDTVYDSSLKFNYSKTKREAEQRTLQAHSEV
```

```
SDR11E2 H. sapiens        QASVPVFIYTSSIEVAGPNSYKEIIQNGHEEEPLENTWPTPYPYSKKLAEKAVLAANGWN
SDR11E2 P. troglodytes    QASVPVFIYTSSIEVAGPNSYKEIIQNGHEEEPLENTWRTPYPYSKKLAEKAVLAANGWN
SDR11E2 M. musculus       QASVPAFIFSSSVDVAGPNSYKEIVLNGHEEECHESTWSDPYPYSKKMAEKAVLAANGSM
SDR11E2 S. harrisii       AAEVPVFLYTSSVEVAGPNSYMKSVRNGHEDDLLESKWSNAYPYSKKLAEKAVLAADGQL
SDR11E2 G. gallus         HCNVQHFIYTSTIEVAGPNCRGDPIFNGDEDTPYESTSKFPYAQSKRLAEECVLKADGQM
SDR11E2 G. japonicus      LQDVKYFIYTSSLEVTGPNRKGDPMCNGDEDSVYRVTDGFPYAETKREAEKSVLELDGLP
SDR11E2 D. rerio          QQSVSSFIYTSSIEVACPNRSGEPIINGHEDTPYSSYPISNYSKTKQEAEQICLQANGEL
SDR11E3 H. sapiens        QTGTRFLVYTSSMEVVGPNTKGHPFYrgNEDTPYEAVHRHPYPCSKALAEWLVLEANGRk  3p.1 4p.0
SDR11E3 P. troglodytes    QTGTRFLVYTSSMEVVGPNTKGHPFYrgNEDTPYEAVHRHPYPCSKALAEWLVLEANGRk  3p.1 4p.0
SDR11E3 M. musculus       QTGTQYLVYTSSMEVVGPNIKGHPFYrgNEDTPYEAVHSHPYPCSKALAEQLVLEANGRk  3p.1 4p.0
SDR11E3 S. harrisii       QTGTRFLVYTSSMEALGPNKKRQPFYrgNEDTPYEVIHTEPYPRSKALAERLVLEANGRk  3p.1 4p.0
SDR11E3 S. harrisii       ALSIPYLLYTSSIAAVGPNTSCEPLLrgNEDTQYTGEVELPYGKTKAMAEKIVLEANGAk  3p.1 4p.0
SDR11E3 A. carolinensis  SEGTQYLVYTSSMEVVGPNTKGDHFYrgNENTPYKSIHELPYPVSKTKAEKLVLEANGRp  3p.1 4p.0
SDR11E3 X. tropicalis     EEGVQYLVYTSSMEVVGPNIHGDHFYrgNEETEYRIYHKEPYPLSKAKAEKLVLEANGTk  3p.1 4p.0
SDR11E3 D. rerio          EIGIQYLVYTSSMEVVGPNVKGDEFVrgNEDTPYNIFHEMPYPKSKAAAEKIVLEANGTk  3p.1 4p.0
                                    *      **        * *           *   *  **   *

SDR11E1 H. sapiens        LKNGGTLYTCALRPMYIYGEGSRFLSASINEALNNNGILSSVG-KFSTVNPVYVGNVAWA
SDR11E1 P. troglodytes    LKNGGTLYTCALRPMYIYGEGSRFLSASVNEALNNNGILSSVG-KFSTVNPVYVGNVAWA
SDR11E1 M. musculus       LKNGGTLNTCALRPMYIYGERSPFIFNAIIRALKNKGILCVTG-KFSIANPVYVENVAWA
SDR11E1 M. domestica      LRNGATMHTCALRPMYIYGEGSQLLQDQIIRALDNDKTFIRNK-EGAQANPVYVGNVAWA
SDR11E1 N. nippon         LKDGGMLMTCALRSMYIFGEGCPFLQGHLDKCLLNKNVYLRFSRKEALVNPVYVGNIAWA
SDR11E1 T. sirtalis       LKGGNSFVTCALRSMYIYGEGSSFLLGHLDESILNNNVFLRLSRKEAIVNPVYVGNIAWA
SDR11E1 L. crocea         LQNGGRLATCTLRPMYIYGEGCRFLLGHMGDAIRNKDVLFRMSLPEARVNPVYVGNVAMA
SDR11E2 H. sapiens        LKNGDTLYTCALRPTYIYGEGGPFLSASINEALNNNGILSSVG-KFSTVNPVYVGNVAWA
SDR11E2 P. troglodytes    LKNGDTLYTCALRPTYIYGEGGPFLSASINEALNNNGILSSVG-KFSTVNPVYVGNVAWA
SDR11E2 M. musculus       LKNGGTLQTCALRPMCIYGERSPLISNIIIMALKHKGILRSFG-KFNTANPVYVGNVAWA
SDR11E2 S. harrisii       LRNGTVLRTCSLRPVYIYGEGSIFLQNEINHALKNDKTFTRKS-KGSMANQVYVDNVAWA
SDR11E2 G. gallus         LKDGGVLVTCALRSMYIFGEGCPFLQGHLDKCLLNKNVYLRFSRKEALVNPVYVGNIAWA
SDR11E2 G. japonicus      LKDGSSFVTCALRSMYIFGEGSPFLLKHVDEAILNNRVFLRISRKEALVNPVYVGNVAWA
SDR11E2 D. rerio          LRDGGHLATCALRPMFIYGPGCRFTLNKLRDAIRSGNVQHRLSQQSAKVNPVYVGNAALA
SDR11E3 H. sapiens        vRGGLPLVTCALRPTGIYGEGHQIMRDFYRQGLRLGGWLFRAIPASVEHGRVYvgNVAWM  5p.2
SDR11E3 P. troglodytes    vRGGLPLVTCALRPTGIYGEGHQIMRDFYRQGLRLGGWLFRAIPASVEHGRVYvgNVAWM  5p.2
SDR11E3 M. musculus       vNGGLPLVTCALRPTGIYGEGHQVMRDFYYQGLRFGGRLFRAVPASVEHGRVYvgNVAWM  5p.2
SDR11E3 S. harrisii       vRGGLPLVTCALRPTGIFGEGHELMLSFYEKAQNTGGWLLRSIPPAVEHGRVYagNVAWM  5p.2
SDR11E3 S. harrisii       lSNGGTLRTCILRANTVYGEKAGFLQELYLLARARRGVLNYLEPEDTERNHTYvgNVAWM  5p.2
SDR11E3 A. carolinensis  mKGGKHLVTCALRPTGIYGENHPLIKEFYKQGLLTGRWMFRAIPASVEHGRVYvgNVAWM  5p.2
SDR11E3 X. tropicalis     mKGGKMLYTCSLRPTGIYGEGHELMKKFHRQGLRTGRCMFRAIPPAIEHGRVYvgNVAWM  5p.2
SDR11E3 D. rerio          vEGGNILYTCCLRPTGIYGEQHQLMKDFYLNSVRNGGWVMRGVPPHTEHGRVYagNVAWM  5p.2
                             *    ** **     *                                 *  * *

SDR11E1 H. sapiens        HILALRALQDPKKAPSIRGQFYYISDDTPHQSYDNLNYTLSKEFGLR-LDSRWSFPLSLM
SDR11E1 P. troglodytes    HILALRALQDPKKAPSIRGQFYYISDDTPHQSYDNLNYTLSKEFGLR-LDSRWSLPLSLM
SDR11E1 M. musculus       HILAARGLRDPKKSTSIQGQFYYISDDTPHQSYDDLNYTLSKEWGLR-PNASWSLPLPLL
SDR11E1 M. domestica      HVLALRTLQDSEKAQSIGGQFYYISDDTPHQSYSEFNYEMTKEWGFK-LGSKIGIPLTFL
SDR11E1 N. nippon         HVQAAKALQVPQKAKHIRGQFYYISDDTPHMSYADLNYELTRELGFG-IEPRLPMPLKML
SDR11E1 T. sirtalis       HIQVAKAMRNPTKVKQIQGRFYYISDDSPHTSYSDFNYELTKELGFG-IEPKPMMPVTLL
SDR11E1 L. crocea         HLQAARSLKDPQKRNVVGGKFYFISDDTPHVSYSDFNHVMMSPLGFI-IQEKLMLPLRLF
SDR11E2 H. sapiens        HILALRALRDPKKAPSVRGQFYYISDDTPHQSYDNLNYILSKEFGLR-LDSRWSLPLTLM
SDR11E2 P. troglodytes    HILALRALRDPKKAPSVRGQFYYISDDTPHQSYDNLNYILSKEFGLR-LDSRWSLPLTLM
SDR11E2 M. musculus       HILAARGLRDPKKSPNIQGEFYYISDDTPHQSFDDISYTLSKEWGFC-LDSSWSLPVPLL
SDR11E2 S. harrisii       HVLALRALRDPEKAQSIGGQFYFITDDTPPQSYSEFNYEVSKEWGFK-LGSKLGIPLTLL
SDR11E2 G. gallus         HVQAAKALQVPQKARHIRGQFYYISDDTPHMSYADLNYELTKELGFG-IEPWLPMPLTML
SDR11E2 G. japonicus      HVQVAKAMRNPAKAKQVRGRFYYISDDSPHLSYADFNHELAKELGFG-VEPRLRMPLTML
SDR11E2 D. rerio          HLQAGRALRDPEKRAVVGGNFYYVSDDTPHISYCDLTHALMSPLGFS-IQNKPILPIFLL
SDR11E3 H. sapiens        HVLAARELEQ--RATLMGGQVYFCYDGSPYRSYEDFNMEFLGPCGLRLVGARPLLPYWLL
SDR11E3 P. troglodytes    HVLAARELEQ--RAALMGGQVYFCYDGSPYKSYEDFNMEFLGPCGLRLVGARPLLPYWLL
SDR11E3 M. musculus       HILVARELEQ--RAALMGGQVYFCYDKSPYKSYEDFNMEFLSPCGLRLIGAHPLLPYWLL
```

```
SDR11E3 S. harrisii        HTLAARELGS--RPSTMGGQVYFCYDDSPYKSYEDFNMEILGRCGIRILGTRPLLPYCLL
SDR11E3 S. harrisii        HVLAARHLQL--KAELLAGQVYYCYDDTPGRKGFLVRHQLLSSAD-PSVKLGSHIPYWKM
SDR11E3 A. carolinensis    HLLAARKIQE--SPVSMGGQVYYCYDSSPYKSYEDFNMEILRPCGFRLLGSRPLIPYFLL
SDR11E3 X. tropicalis      HLLAARQLQI--HPSTLGGQVYFCYDSSPYKSYEDFNMEFLSACGFKMIGSRPLVPYFLL
SDR11E3 D. rerio           HLLAARALQE--HPNRLGGECYFCYDDSPYKPYDEFNMQFLSAFNFRSL----RLPVWML
                           *                 *  *   *  *                          *

SDR11E1 H. sapiens         YWIGFLLEIVSFLLRPIYTYRPPFNRHIVTLSNSVFTFSYKKAQRDLAYKPLYSWEEAKQ
SDR11E1 P. troglodytes     YWIGFLLEIVSFLLRPIYTYRPPFNRHIVTLSNSVFTFSYKKAQRDLAYKPLYSWEEAKQ
SDR11E1 M. musculus        YWLAFLLETVSFLLRPVYRYRPLFNRHLITLSNSTFTFSYKKAQRDLGYEPLVNWEEAKQ
SDR11E1 M. domestica       YWAAFLLEMISFMLGPIFMYEPPFNRHLLTLTNSIFTFSYKKAQKDFGYKPRFSWLEAKQ
SDR11E1 N. nippon          YYFSLLLEIVSFLLRPFVRYIPSTNRHLVTLLNTPFTFSYRKAQKDFGYMPRYTWEEAKQ
SDR11E1 T. sirtalis       YYYALFLEILSFLLRPFFRYVPSINRFLVILLNTPFSFSYKKAQRDFNYTPRYSWEEAKQ
SDR11E1 L. crocea          YVVCFLLEVLCMMLRPFVRITPPLNRQLLTMLNTPFSFSYQKAKRDLGYIPRYTWEEARK
SDR11E2 H. sapiens         YWIGFLLEVVSFLLSPIYSYQPPFNRHTVTLSNSVFTFSYKKAQRDLAYKPLYSWEEAKQ
SDR11E2 P. troglodytes     YWIGFLLEVVSFLLSPIYSYQPPFNRHTVTLSNSVFTFSYKKAQRDLAYKPLYSWEEAKQ
SDR11E2 M. musculus        YWLAFLLETVSFLLSPIYRYIPPFNRHLVTLSGSTFTFSYKKAQRDLGYEPLVSWEEAKQ
SDR11E2 S. harrisii        YWAAFVLEMISFMLSPIFIYEPPFNCHFLTLTNSVFTLSCKKAKKDLGYEPRVSWLEARK
SDR11E2 G. gallus          YYFSLLLEIVSFMLRPFVRYIPSTNRHLVTLLNTPFTFSYRKAQKDFGYVPRYTWEEAKR
SDR11E2 G. japonicus       YCYALLLEIMSFLLRPFVRYIPVINRHLLTVLNTPFTFSFQKAQRDFGYAPRYSWEEAKQ
SDR11E2 D. rerio           YLLAFFMEILQAVLRPVLRFTPPLNRQLVTMVNTPFSFSYQKACREFGYSPRYDWEEARR
SDR11E3 H. sapiens         VFLAALNALLQWLLRPLVLYAPLLNPYTLAVANTTFTVSTDKAQRHFGYEPLFSWEDSRT
SDR11E3 P. troglodytes     VFLAALNALLQWLLRPLVLYAPLLNPYTLAVANTTFTVSTDKAQRHFGYEPLFSWEDSRT
SDR11E3 M. musculus        VLLATLNALLQWLLRPLVLYTPLLNPYTLAMANTTFTVSTNKAQRHFGYKPLFSWEESRT
SDR11E3 S. harrisii        FFLATLNAFLQWLLRPLVVYTALLNPYTLATANTTFTVCTDKAQRHFGYQPLYGWEESRD
SDR11E3 S. harrisii        WLMIQLHRIIRAILSPFWRPQPFLNVPLLNTIVTTFSFETDKASRHFGYKPLFTWQESRL
SDR11E3 A. carolinensis    HLIAFLNVFLQWVLKPFFTYAPILNPYTLVIASTTFTVATDKAQRHFGYKPQYTWEESLS
SDR11E3 X. tropicalis      YLLALLNTLLQWVLHRFFIYAPILNPYTLAVASTTFTVQTDKAEKHFGYRPLYAWEEAKK
SDR11E3 D. rerio           WIIAWMNDMVRWVLKPIYNYTPLLNKYTLAVACTSFTVSTDKAFRHFQYQPLYSWQQCLS
                                        *          *          *     **     * *   *

SDR11E1 H. sapiens         KTVEWVGSLVDRHKETLKSKTQ------
SDR11E1 P. troglodytes     KTVEWVGSLVDRHKETLKSKTQ------
SDR11E1 M. musculus        KTSEWIGTIVEQHREILDTKCQ------
SDR11E1 M. domestica       KTSQWIGTLVAQNSEYQKTKTP------
SDR11E1 N. nippon          RTAQWIASMVPQRREYLKSKAA------
SDR11E1 T. sirtalis       RTSQWIADITPLRAAYLKSKTT------
SDR11E1 L. crocea          HTIEWLASQLPKERERIRAK--------
SDR11E2 H. sapiens         KTVEWVGSLVDRHKETLKSKTQ------
SDR11E2 P. troglodytes     KTVEWVGSLVDRHKETLKSKTQ------
SDR11E2 M. musculus        KTSEWIGTLVEQHRETLDTKSQ------
SDR11E2 S. harrisii        KTSQWVGTLLAKNRENLKIKTP------
SDR11E2 G. gallus          YTSQWIASIVPQRREYLKSKKA------
SDR11E2 G. japonicus       RTSRWIAEVTPLRTTYLRSKEV------
SDR11E2 D. rerio           STTDWLASVLPAERRLANKT--------
SDR11E3 H. sapiens         RTILWVQAATGSAQ--------------
SDR11E3 P. troglodytes     RTILWVQAATGSAQ--------------
SDR11E3 M. musculus        RTIQWVQAMEGSAR--------------
SDR11E3 S. harrisii        RTVSWVKTKKGCSGPRH-----------
SDR11E3 S. harrisii        RTAQWLKAAAGSLGPPQLQEKKN-----
SDR11E3 A. carolinensis    RTVKWLQEVDTQTQAGK-----------
SDR11E3 X. tropicalis      RTITWIKSLEVREKDLKDVGAKGNYCCN
SDR11E3 D. rerio           RTQSWVNTFPFETSTKDK----------
                            *  *
```

Figure S16. Alignment of the vertebrate SDR11E family variants. For further details see Fig. S13.

* symbol marks the position of identical amino acid residues of the aligned SDR protein sequences.

```
SDR12C1 H. sapiens       ---------------MESALPAAGFLYWVGAGTVAY--LALR-ISYSLFTALRVWGVGNE
SDR12C1 P. troglodytes   ---------------MESALPAAGFLYWVGAGTVAY--LALR-ISYSLFTALRVWGVGNE
SDR12C1 M. musculus      ---------------MECAPPAAGFLYWVGASTIAY--LALR-ASYSLFRAFQVWCVGNE
SDR12C1 M. domestica     --------------------MESYGFLYWVGVSTVAY--LSLR-LSYSLFSAIRVWGLAHE
SDR12C1 A. carolinensis  -----------MEASESFLAPWTGFFYWVGVAGVAY--WTVL-LLRYLRYIVRVWVTGNP
SDR12C1 Z. albicollis    -------------MVAPAALPAVGLFYWVGALGALY--AAAL-ASYRLLAGLRVWVLGSG
SDR12C1 X. tropicalis    ---------MATESLAEVPVPGCNCFWYLGVVAAVW--WGLR-AAWCLLDGARVWVLGSG
SDR12C1 P. latipinna     ---------MNRDSVDEMMSRAETPLFWVGAFTVAS--MALW-LLYRLLTGFRIWILGNG
SDR12C2 H. sapiens       -----------------MGDVLEQFFILTGLLVCLA--CLAKCVRFSRCVLLNYWKVLP-
SDR12C2 P. troglodytes   -----------------MGDVLEQFFILTGLLVCLA--CLAKCVRFSRRILLNYWKVLP-
SDR12C2 M. musculus      ---------------------MEKLFIAAGLFVGLV--CLVKCMRFSQHLFLRFCKALP-
SDR12C2 S. harrisii      -----------------MGEIQDQLFILIGALVCLK--YLLKCVKFSKYFLLRLWNPLP-
SDR12C2 G. gallus        -----------------MEDFQHQLLIVTGALICFS--ALLKCIRFMKYTFPHVWSALP-
SDR12C2 A. carolinensis  ------------------------------------------------------------
SDR12C2 X. tropicalis    ---------------------MEQFLFVVGLITCLY--LTVKFFGFLMYLFQHLRGVLP-
SDR12C2 D. rerio         ------------------MTLTEIIFVLTGTCAILV--FGGKIASLIMMLITKLFCPLP-
SDR12C3 H. sapiens       MAAVDSFYLLYREIARSCNCYME-ALALVGAWYTARKSITVICDFYSLIRL-HFIPRLGS
SDR12C3 P. troglodytes   MAAVDSFYLLYREIARSCNCYME-ALALVGAWYTARKSITVICDFYSLIRL-HFIPRLGS
SDR12C3 M. musculus      MAAVDSFYLLYREIARSCNCYME-ALALVGAWYTARKSITVICDFYSLVRL-HFIPRLGS
SDR12C3 S. harrisii      MAAVDSFYLLYREIARSCNCYME-ALALVGAWYTARKSITIICDFYSLIRL-HFIPRLVS
SDR12C3 G. gallus        MAAVDRFNLLYREISRSCSVYIE-ALAIVGAWYTVRKCLTLVFNTYSMIRL-HALPKLVG
SDR12C3 G. japonicus     MAAVDSFSLLLRDIGRTCNCYME-TLALIGALYTAKMCFTFVNDTYTLVRL-HFIPRLVR
SDR12C3 X. tropicalis    MAAVDSFHLLYRQVAWSCQSHME-FLAVVGALYTAGKGLKLICQSYNLIRL-HITPLLFS
SDR12C3 L. oculatus      MAAVDSFYLLYREIARSCNCYVE-TLALVGAFYTASTAVAIARDCYSLIRL-HFIPRLMS

                                        ×   × ×
SDR12C1 H. sapiens       -AGVGPGLGEWavVTGSTDGIGKSYAEelAKHGMKVVLISRSKDKLDQVSSEikEKFKVE  1p.2 2p.0 3p.2
SDR12C1 P. troglodytes   -AGVGPGLGEWavVTGSTDGIGKSYAEelAKHGMKVVLISRSKDKLDQVSSEikEKFKVE  1p.2 2p.0 3p.2
SDR12C1 M. musculus      -ALVGPRLGEWavVTGGTDGIGKAYAEelAKRGMKIVLISRSQDKLNQVSNNikEKFNVE  1p.2 2p.0 3p.2
SDR12C1 M. domestica     -DGVGPALGEWavVTGSTDGIGRSYAEelAKRGMKIVLISRSQEKLKEVANDikEKFKVE  1p.2 2p.0 3p.2
SDR12C1 Z. albicollis    AAAVGPALGAWavVTGATDGIGKAYAEelARRGMKVVLISRSKEKLDQVSTEirEKYKVE  1p.2 2p.0 3p.2
SDR12C1 A. carolinensis  -GAVGPHLGAWavVTGATDGIGKAYTEelAKRGLKVVLISRSQEKLDQVASDirEKFKVE  1p.2 2p.0 3p.2
SDR12C1 X. tropicalis    -AQVGPRIGKWavVTGATDGIGKAYAEelAKRGMNIVLISRSPEKLEEVAKQikEKFKVE  1p.2 2p.0 3p.2
SDR12C1 P. latipinna     -QLLTPKLGKWavVTGATDGIGKSYAEelARRGFAMMLISRSQEKLDDVARSleEQFKVE  1p.2 2p.0 3p.2
SDR12C2 H. sapiens       -KSFLRSMGQWavITGAGDGIGKAYSFelAKRGLNVVLISRTLEKLEAIATEieRTTGRS  1p.2 2p.0 3p.2
SDR12C2 P. troglodytes   -KSFLQSMGQWavITGAGDGIGKAYSFelAKRGLNVVLISRTLEKLEAIATEieRTTGRS  1p.2 2p.0 3p.2
SDR12C2 M. musculus      -SSFLRSMGQWavITGAGDGIGKAYSFelARHGLNVVLISRTLEKLQTIAEEieRTTGSC  1p.2 2p.0 3p.2
SDR12C2 S. harrisii      -KSFFRSMGEWavITGAGDGIGKAYSYelAKHGLNIVMISRTLEKLQAVAKGieQTTGSQ  1p.2 2p.0 3p.2
SDR12C2 G. gallus        -QAFFRSLGEWavVTGAGDGLGKAYSFelAKRGLNIVMISRTLEKLQRVANEieQATGQK  1p.2 2p.0 3p.2
SDR12C2 A. carolinensis  -------MGEWavITGAGDGIGRAYSIelAKRGLNIVLISRTFQKMQRVALDieQTTGQR  1p.2 2p.0 3p.2
SDR12C2 X. tropicalis    -QSFFQSLGEWavVTGAGDGIGKAYSTelANRGMNIVMISRTLEKMQAVAMDieQSTGKN  1p.2 2p.0 3p.2
SDR12C2 D. rerio         -EAFFTSLGKWavITGGSDGIGRAYAEelSKQGMSVIIISRNQEKLDRAAKKieLNTGGK  1p.2 2p.0 3p.2
SDR12C3 H. sapiens       RADLIKQYGRWAVVsgATDGIGKAYAEELASRGLNIIILISRNEEKLQVVAKDIADTYKVE  1p.2
SDR12C3 P. troglodytes   RADLIKQYGRWAVVsgATDGIGKAYAEELASRGLNIIILISRNEEKLQVVAKDIADTYKVE  1p.2
SDR12C3 M. musculus      RPDLIKQYGRWAVIsgATDGIGKAYAEELASHGLNVILISQEEEKLQAAAKHIADTYRVE  1p.2
SDR12C3 S. harrisii      RADLIKQYGRWAVVsgATDGIGKAYAEELASRGLNIVLISQNEEKLHKLSKTLAETYKVE  1p.2
SDR12C3 G. gallus        EIDIVKRYGRWAVVtgSTDGIGKAYAEELAKRGVNIIILISRSKEKLEAVSRSISETYKVE  1p.2
SDR12C3 G. japonicus     KADLVKLYGQWAVVtgGTSGIGKYYAKELASRKVNIIILVSRNQEKLEALAREIADTYEVE  1p.2
SDR12C3 X. tropicalis    RTNLGRQYGAWAVVtgATSGIAQAYAEELARCGMNVVLVDNNREKLQKMSDSITATHGVN  1p.2
SDR12C3 L. oculatus      RRDLVHQYGQWAVVcgASDGLGRAYTEELAREGVSIILISRSESGLQSFAKAIADAYGVE  1p.2
                                 * ***  *   *    *  **

SDR12C1 H. sapiens       TRTIAVDFAS-EDIYDKIKTGLAGLEIGIlvNNVGMSY-EYPEYFLDVPDLDnvIKKMIN  4p.2 5p.0
SDR12C1 P. troglodytes   TRTIAVDFAS-EDIYDKIKTALAGLEIGIlvNNVGMSY-EYPEYFLDVPDLDnvIKKMIN  4p.2 5p.0
SDR12C1 M. musculus      TRTIAVDFSL-DDIYDKIKTGLSGLEIGVlvNNVGMSY-EYPEYFLEIPDLDntIKKLIN  4p.2 5p.0
SDR12C1 M. domestica     TKTIAVDFGA-VDIYNKIEASLTGLEIGVlvNNVGMSY-EYPEYFLDIPDLDntINKLIN  4p.2 5p.0
```

```
SDR12C1 Z. albicollis    TKVIVADFGEREDIYNGIKTGLEGLEIGVlvNNVGISY-AYPEYFIDIPELEktIDKMVN  4p.2 5p.0
SDR12C1 A. carolinensis  TKTIVADFQDRETIYSKIKAGLEGLEIGIlvNNVGVSY-SYPENFLDVPELDklIDNMIN  4p.2 5p.0
SDR12C1 X. tropicalis    TKIIAADFGKPTEIYGRIESGLRDLEIGVlvNNVGVSY-EHPEYFLEIPDLEntLDKMIN  4p.2 5p.0
SDR12C1 P. latipinna     TRTIAVDFVK-LDIYSKIEEGLADLEIGVlvNNVGVSY-PYPEYYLHIPNLEnfITNTIN  4p.2 5p.0
SDR12C2 H. sapiens       VKIIQADFTK-DDIYEHIKEKLAGLEIGIlvNNVGMLPNLLPSHFLNAPDEIqsL---IH  4p.2 5p.0
SDR12C2 P. troglodytes   VKIIQADFTK-DDIYEHIKEKLAGLEIGIlvNNVGMLPNLLPSHFLNAPDEIqsL---IH  4p.2 5p.0
SDR12C2 M. musculus      VKIVQADFTR-EDIYDHIKEHLEGLEIGIlvNNVGMLPSFFPSHFLSTSGESqnL---IH  4p.2 5p.0
SDR12C2 S. harrisii      VKIIQADFTK-DDIYENIKESLQGLEIGIlvNNVGMVHNYLPSHFLSGPDKIqnL---IH  4p.2 5p.0
SDR12C2 G. gallus        VKVIQADFTR-NSVYKNIEKDLEGLEIGVlvNNVGMLHNPLPCRFLNAPDVDenL---VN  4p.2 5p.0
SDR12C2 A. carolinensis  VKIIQADFTK-MDIYSDIE-SLQGLEVGIlvNNVGMLQTSIPCHFLDAPDNDqaL---IN  4p.2 5p.0
SDR12C2 X. tropicalis    VKIIQADFTK-DNIYEHIEEGLKGLKIGIliNNVGMLHNPDPCRFLSGPDNDknV---IN  4p.2 5p.0
SDR12C2 D. rerio         VKVIAADFTK-DDIYGHITENIEGLDIGVlvNNVGILPSQIPCKLLETSDLEerIYDIVN  4p.2 5p.0
SDR12C3 H. sapiens       TDIIVADFSSGREIYLPIREALKDKDVGILVNNVGVFY-PYPQYFTQLSE--DKLWDIIN
SDR12C3 P. troglodytes   TDIIVADFSSGREIYLPIREALKDKDIGILVNNVGVFY-PYPQYFTQLSE--DKLWDIIN
SDR12C3 M. musculus      TLVLVADFSRGREIYAPIREALRDRDIGILVNDVGAFY-PYPQYFSQVPE--DTLWDIVN
SDR12C3 S. harrisii      TEIIVADFSNGRGIYLLIREALQDRDIGILVNNVGVFY-PYPQYFTQVSE--EKLWDIID
SDR12C3 G. gallus        TDFIVADFSKGREAYQAIKEGLKDREIGILVNNVGLFY-TYPDYFTNLSE--DMLWDMIN
SDR12C3 G. japonicus     TAIIVVDFNKGSEIYPALKNVLEDKEIGILVNNVGVFY-THPDYFANLTW--DKIWELIN
SDR12C3 X. tropicalis    TSFIEVDFCKGHEAYRPIKDALRHVEVGILVNCVGNFL-EYPQSVIECPE--EQLWKIIH
SDR12C3 L. oculatus      TATIGADFSQGQEAYKPIKDAVKDKEIGFLVNNICVPS-EYPQHFTNVPE--NKLWDIIN
                               **      *            * * *         *


                                                           +   +
SDR12C1 H. sapiens       INILSVCkmTQLVLPGMVErsKGAILNISSGSGMLPVPLLTIYSATktFVDFFSQCLHEE  6p.0 7p.1 8p.0
SDR12C1 P. troglodytes   INILSVCkmTQLVLPGMVErsKGAILNISSGSGMLPVPLLTIYSATktFVDFFSQCLHEE  6p.0 7p.1 8p.0
SDR12C1 M. musculus      INVLSVCkvTRLVLPGMVErsKGVILNISSASGMLPVPLLTIYSATkaFVDFFSQCLHEE  6p.0 7p.1 8p.0
SDR12C1 M. domestica     INIFSVCkmTQLVLPGMVKrsKGAILNISSASGMLPAPLLTIYSATkaFVDFFSQCLHVE  6p.0 7p.1 8p.0
SDR12C1 Z. albicollis    INIMSVCkmTRLVLPGMLErsKGIILNIASASGMCPTPLLTLYSATkaFVDYFSRGLNAE  6p.0 7p.1 8p.0
SDR12C1 A. carolinensis  INCISVCkmTQLVLPGMLKrsKGVIVNVSSIAAVSPTPFLAVYSATkaFVNYFSHCLNVE  6p.0 7p.1 8p.0
SDR12C1 X. tropicalis    INITSVCqmTRLVLPGMLGrgRGVILNISSASGMYPVPLLTVYSATkaFVDFFSRGLQAE  6p.0 7p.1 8p.0
SDR12C1 P. latipinna     VNMTSVCqmTRLVLPKMVArsKGVILNISSASGMYPVPLLTVYSATkaFVDFFSRGLHEE  6p.0 7p.1 8p.0
SDR12C2 H. sapiens       CNITSVVkmTQLILKHMESrqKGLILNISSGIALFPWPLYSMYSASkaFVCAFSKALQEE  6p.0 7p.1 8p.0
SDR12C2 P. troglodytes   CNITSVVkmTQLILKHMESrrKGLILNISSGIALFPWPLYSMYSASkaFVCAFSKALQEE  6p.0 7p.1 8p.0
SDR12C2 M. musculus      CNITSVVkmTQLVLKHMESrrKGLILNISSGAALRPWPLYSLYSASkaFVYTFSKALSVE  6p.0 7p.1 8p.0
SDR12C2 S. harrisii      CNISSVVkmTRLILRDMEIrrKGLILNISSGAGRFPCPLYSLYSSTkaFVCTFSKALQAE  6p.0 7p.1 8p.0
SDR12C2 G. gallus        CNIISVTkmTQIILKQMELrqKGLILNLSSGLGTFPCPLYTIYSASkaFICTFSKALQAE  6p.0 7p.1 8p.0
SDR12C2 A. carolinensis  CNIMSVTqmTRIVLKQMVPrqKGLILNISSavGTFPCPLYAIYSASkaFGCTFSKALQAE  6p.0 7p.1 8p.0
SDR12C2 X. tropicalis    CNITSTIkmTRIILKQMEKrkSGLILNISSAVGRFPCPLYAVYSASkaFVTTFSKALQAE  6p.0 7p.1 8p.0
SDR12C2 D. rerio         CNVKSMVkmCRIVLPGMQQrrRGVILNVSSGIAKIPCPIYTLYAASkvFVERFSQGLQAE  6p.0 7p.1 8p.0
SDR12C3 H. sapiens       VNIAAASLMVHVVLPGMVERKKGAIVTISSGSCCKPTPQLAAFSASkaYLDHFSRALQYE  2p.0
SDR12C3 P. troglodytes   VNIAAASLMVHVVLPGMVERKKGAIVTISSGSCCKPTPQLAAFSASkaYLDHFSRALQYE  2p.0
SDR12C3 M. musculus      VNIAAASLMVHIVLPGMVERKKGAIVTVSSGSCCKPTPQLAAFSASkaYLDHFSRALQYE  2p.0
SDR12C3 S. harrisii      VNIAAASLMVHIVLPGMVARRKGAIVNISSGSCCKPTPQMTAYSASkaYLDHFSRALQYE  2p.0
SDR12C3 G. gallus        VNIASANMMVHIVLPGMVEKRKGAIVNVSSASCCQPTPMLTTYGASkaYLDYFSRALYYE  2p.0
SDR12C3 G. japonicus     VNIGAATMMVHMVLPGMVERKKGAIVNISSMSCCQPTPLMTAYSASkaYLDHFSRALHYE  2p.0
SDR12C3 X. tropicalis    VSVSAATIMAKIVVPGMAQRRRGAIVNVSFRSCCKPNFPMTMYTPCqlYMDGFTKELQSE  2p.0
SDR12C3 L. oculatus      INIAAATMMVHIVLPGMVERKKGAIINISSGSCCKPTPKMTAYSASkaYLDHFSRALYYE  2p.0
                                         *     * *          *                *   *  *

SDR12C1 H. sapiens       YRSKGVFVqsVLPYFVATKLAKIRKP----TLDKPSPETFVKSAIKTVGLQSRTNGYLIH  9p.0
SDR12C1 P. troglodytes   YRSKGVFVqsVLPYFVATKLAKIRKP----TLDKPSPETFVKSAIKTVGLQSRTNGYLIH  9p.0
SDR12C1 M. musculus      YKSKGIFVqsVMPYLVATKLAKIQKP----TLDKPSAETFVKSAIKTVGLQTRTTGYVIH  9p.0
SDR12C1 M. domestica     YRSKGIIVqsVLPYFVATKLAKIRKP----TFDKPSAEAFVRSAIKTVGLQSRTNGYPVH  9p.0
SDR12C1 Z. albicollis    YKSKGIIVqsVLPYYVATKMSKIRKP----TLDKPSPERYVRAALGTVGLQSQTNGYLPH  9p.0
SDR12C1 A. carolinensis  YKRKGIIVqsLVPHLVVTNMSKLRKA----SRFRPMPGWFVKYAINTVGLESETAGYPYH  9p.0
```

```
SDR12C1 X. tropicalis    YRSKGVTVqsVLPFYVATKLAKIRKP----TWDKPSPETYVQSALNTVGLQTQTNGYLPH  9p.0
SDR12C1 P. latipinna     YRRQGIIIqsVLPFFVATKMTRIRKP----TLDKPTPERYVAAELNTVGLQNQTNGYFPH  9p.0
SDR12C2 H. sapiens       YKAKEVIIqvLTPYAVSTAMTKYLNT----NVITKTADEFVKESLNYVTIGGETCGCLAH  9p.0
SDR12C2 P. troglodytes   YKAKEVIIqvLTPYAVSTAMTKYLNT----NVITKTADEFVKESLNYVTIGGETCGCLAH  9p.0
SDR12C2 M. musculus      YRDKGIIIqvLTPYSISTPMTKYLNN----K-MTKTADEFVKESLKYVTIGAESCGCLAH  9p.0
SDR12C2 S. harrisii      YKEKGIIIqvVTPYSISTPMTKHINP----NKITKTADEFVKESLDFVAVGDETCGCLAH  9p.0
SDR12C2 G. gallus        YKEKGIIIqvVAPYGISTPMTMHQKP----GLITKTAEEFVSESLHYVTFGDEIFGCLAH  9p.0
SDR12C2 A. carolinensis  YNTKGIIIqaVTPYSVSTPMTKWSKP----NLINKTAEDFVRESLEYVTLGDETFGCLAH  9p.0
SDR12C2 X. tropicalis    YKSKGIIIqaVTPYGVSTPMTRNART----NAITKRPVDFVRQSLNCVTIGDETFGCFAH  9p.0
SDR12C2 D. rerio         YISKGIIIqtVAPFGVSTAMTGHQKP----DMVTFTAEEFVRSSLKYLKTGDQTYGSITH  9p.0
SDR12C3 H. sapiens       YASKGIFVQSLIPFYVATSMTAPSNFLHRCSWLVPSPKVYAHHAVSTLGISKRTTGYWSH
SDR12C3 P. troglodytes   YASKGIFVQSLIPFYVATSMTAPSSFLHRCSWLVPSPKVYAHHAVSTLGISKRTTGYWSH
SDR12C3 M. musculus      YASKGIFVQSLIPFYVTSSGAAPASFLHRCPWLAPSPRVYAQHAVSTLGISKRTTGYWSH
SDR12C3 S. harrisii      YASKGIFVQSLIPFSVATNVKACRSFLHGCSWLVPSPKVYAHHAIATLGISKRTTGYWFH
SDR12C3 G. gallus        YASKGIFVQSLTPFVIATRMVSCSRVTSKRSFFFPSAEEYASHAISTLGLSKRTPGYWKH
SDR12C3 G. japonicus     YAPQGIFVQSLIPFFISTNMTKFSKELTPKNFLVPSAEVYAHHAVTTLGISRRTTGYWLH
SDR12C3 X. tropicalis    LSSKGIFVQSLTPLCVAKERTLHYRPSFRFPFLVPSPEVYARHAVQMLGVSHRTTGYWAH
SDR12C3 L. oculatus      YASKGVFVQSLVPFYISEVGAAS------GGWLVPHPQVYARHAISTLGISHRTTGYWPH
                                 *   *                                          *   *

SDR12C1 H. sapiens       ALmgSIIS-NLPSWIYLKIVMNMNK-STRAHYLKKTKKN-----------  10p.0
SDR12C1 P. troglodytes   ALmgSIIS-NLPSWIYLKIVMNMNK-STRAHYLKKTKKN-----------  10p.0
SDR12C1 M. musculus      SLmgSINS-IMPRWMYFKIIMGFSK-SLRNRYLKKRKKN-----------  10p.0
SDR12C1 M. domestica     AImgWFLSCLLPSWLSMKLAYSVNK-GSRAHYLKKHKKA-----------  10p.0
SDR12C1 Z. albicollis    ALmgWVIS-LLPTSTAINLIMKTNK-QIRARYFKKMKEK-----------  10p.0
SDR12C1 A. carolinensis  ELwvWLIH-MLPRWVVDESATRLAV-KSKYNLLKKQKAN-----------  10p.0
SDR12C1 X. tropicalis    AIwiWISTSLVPVSTAISLGMKMNK-GLRARFLKRAKQK-----------  10p.0
SDR12C1 P. latipinna     AVmgWVTTRLVPSSIVIFLGARMNR-LQRTGYLHRRKLREQRNGASLKTE  10p.0
SDR12C2 H. sapiens       EIlaGFLS-LIPAWAFYSGAFQRLLLTHYVAYLKLNTKVR----------  10p.0
SDR12C2 P. troglodytes   EIlaGFLS-LIPAWAFYSGAFQRLLLTHYVAYLKLNTKVR----------  10p.0
SDR12C2 M. musculus      EIiaIILN-RIPSRIFYSSTAQRFLLTRYSDYLKRNISNR----------  10p.0
SDR12C2 S. harrisii      EIlaHLLD-FIPSWVIYSDLAQNIFLIHCTNYLKQNSNIQ----------  10p.0
SDR12C2 G. gallus        ELlaCVLQ-LVPLWVLHSDRLQEVALQAFTSYLKKRWRKS----------  10p.0
SDR12C2 A. carolinensis  EIlaRLVQ-CIPLWIFHSERVQNMLKDAISGQLKKNSNST----------  10p.0
SDR12C2 X. tropicalis    AVlgCIMD-CIPLWVIHSTAVQEKFLHRFNRKKKKI--------------  10p.0
SDR12C2 D. rerio         TLlgRIVQ-SIPTWVLQSETFQHHFQ----EYVKNRDRR-----------  10p.0
SDR12C3 H. sapiens       SIqfLFAQ-YMPEWLWVWGANILNRSLRKEALSCTA--------------   3p.0
SDR12C3 P. troglodytes   SIqfLFAQ-YMPEWLWVWGANILNRSLRKEALSCTA--------------   3p.0
SDR12C3 M. musculus      SIqfLFAQ-YMPEWLWVWGANLLNRSLRKEALSCQA--------------   3p.0
SDR12C3 S. harrisii      SIqfLFAQ-YMPEWLWAWGANIINNSLRHEALSHRL--------------   3p.0
SDR12C3 G. gallus        SIefTLGE-RLPEWIWAWFAQYFCRIIRKEALTHKAK-------------   3p.0
SDR12C3 G. japonicus     TIlfLLGQ-YIPEWLWAWGTYRMNNTLRLEATLQGPH-------------   3p.0
SDR12C3 X. tropicalis    SMqlAAAC-WLPDFICQLMGRFLHTASE----------------------   3p.0
SDR12C3 L. oculatus      SIqfWLSR-YMPEWMWVWGSNMLSRTI-----------------------   3p.0
                                    *
```

Figure S17. Alignment of the vertebrate SDR12C family variants. For further details see Fig. S13 and Table S17.

* symbol marks the position of identical amino acid residues of the aligned SDR protein sequences.

```
                                                                            ×   × ×
SDR16C1 H. sapiens           ---MVWKRLGALVMFPLQMIYLVVKAAVGLVLPAKLRDLSRENVLITGGGRGIGRQLARE
SDR16C1 P. troglodytes       ---MVWKRLGALVMFPLQMIYLVVKAAVGLVLPAKLRDLSRENVLITGGGRGIGRQLARE
SDR16C1 M. musculus          ---MVWKWLGALVVFPLQMIYLVTKAAVGMVLPPKLRDLSRESVLITGGGRGIGRHLARE
SDR16C1 G. gallus            ---MVWKWLGAVLLLPVQMLYLVLKAAVCALLPPKLRDLSGDAVLVTGGGRGIGRQLAKE
SDR16C1 G. japonicus         ---MVWKWLAAWLLLPVQLLGLVAKALVGTLLPSKLRDLSGDSVLITGGGRGIGRHLARE
SDR16C1 X. tropicalis        ---MDMRSVGRLLLFPVQMLFAILKAAANLLMPTRLRDLSGDTVLITGGGRGIGRHLARE
SDR16C1 D. rerio             ---MEMKVLGCALLFPFQIVFSILKATVRFFTRQKRRDLGTDVVLITGGGRGIGRHLAKE
SDR16C2 H. sapiens           -MKF----LLDILLLLPLLIVCSLESFVKLFIPKRRKSVTGEIVLITGAGHGIGRLTAYE
SDR16C2 P. troglodytes       -MKF----LLDILLLLPLLIVCSLESFVKLFIPKRRKSVTGEIVLITGAGHGIGRLTAYE
SDR16C2 M. musculus          -MKY----LLDLILLLPLLIVFSIESLVKLFIPKKKKSVAGEIVLITGAGHGIGRLTAYE
SDR16C2 S. harrisii          -MYV----VLELLLLLPILIYSTLESFVKFFIPKKRKSVSGEIVLITGAGHGIGRLTAYE
SDR16C2 G. gallus            -MNP----ALGLLVFLGTLLYAYAEALVKLLLPAKRKAVRGELVLVTGAARGLGRATARE
SDR16C2 A. mississippiensis  -MHT----ALELLRLLPLLLLAYLEALARLLLPPRRKSVQGETVLVTGAGHGLGRSTALE
SDR16C2 X. tropicalis        -MHV----LLEIVWLLLVIVYSYLESFVKLFIPLKRKSVKGEVVLITGAGHGIGKITAQI
SDR16C3 H. sapiens           -MNI----ILEILLLLITIIYSYLESLVKFFIPQRRKSVAGEIVLITGAGHGIGRQTTYE
SDR16C3 P. troglodytes       -MNI----ILEILLLLITIIYSYLESLVKFFIPQRRKSVAGEIVLITGAGHGIGRQTAYE
SDR16C3 M. musculus          -MNL----ILEFLLLVGVIIYSYLESLVKFFIPRRRKSVTGQTVLITGAGHGIGRLTAYE
SDR16C3 S. harrisii          -MNI----IAELFLLLAIVIYSYLEALVRLFIPVKRKSVSGEIVLITGAGHGIGRLTAYE
SDR16C3 F. albicollis        -MNV----FVELLLFLATLLYSYLEAFVKLFVPARRKSLSGELVLITGAGHGVGRATALE
SDR16C3 A. carolinensis      -------------------MYSCLEALVKLFIPKNRKSVYGEIVLITGAGHGLGRATAYE
SDR16C3 X. tropicalis        -MNI----ILEYMFLLFIAVYSYMESFVKLFLPVNRKSVAGNIVLITGSGHGIGRRTALE
SDR16C4 H. sapiens           -MNI----VVEFFVVTFKVLWAFVLAAARWLVRPKEKSVAGQVCLITGAGSGLGRLFALE
SDR16C4 P. troglodytes       -MNI----VVEFFVVTFKVLWAFVLAAARWLVRPKEKSVAGQVCLITGAGSGLGRLFALE
SDR16C4 M. musculus          -MNI----VVEFFVVTFKVLWAFVLAAARWLVRPKEKSVAGQVCLITGAGSGLGRLFALE
SDR16C4 M. domestica         -MNI----VVEFFVVTFKVLWAFVLAAAKWLVRPKEKSVAGQVCLITGAGSGLGRLFALE
SDR16C4 G. gallus            -MNI----LLELFVVTFRVLWAFVLAAAKWLVRPKEKSVAGQVCLITGAGSGLGRLFALE
SDR16C4 G. japonicus         -MNI----ILEFFVVTFKVLWAFVVAAAKWFMRPKEKSVAGQVCLITGAGSGLGRLFALE
SDR16C4 X. tropicalis        -MHI----VLEFFLVTFKVLWAFVLAAAKWLVRPKDKSVAGQVCLITGAGSGLGRLFALE
SDR16C4 P. latipinna         MMMI----IAEFFVVILKVLWAFVTAGARWVVRPKEKSVTGQVCVITGAGSGLGRLFAKE
SDR16C5 H. sapiens           -MSFNLQSSKKLFIFLGKSLFSLLEAMIFALLPKPRKNVAGEIVLITGAGSGLGRLLALQ
SDR16C5 P. troglodytes       -MSFNLQSSKKLFIFLGKSLFSLLEAMIFALLPKPRKNVAGEIVLITGAGSGLGRLLALQ
SDR16C5 M. musculus          -MSQNLESVKNLLVFLGKSLLSVLEALLFHVISKPRKNVAGEIVLITGAGSGLGRLLALQ
SDR16C5 M. domestica         -MLSKMNDLNHLLIFLGKFTYGFLEALFYMIAPKPKKNVSGEIVLITGAGSGIGRLLALR
SDR16C5 G. gallus            -MNF----FLELLKVIGLTTYYMLEALVLLFVPKRKKNVSGEIVLITGAGSGIGRLLSLK
SDR16C5 P. bivittatus        -MNF----FLETLRVILMCIYYLLEFFL-SFIFARKKNIAGEIVLITGAGSGIGRLMALK
SDR16C5 X. tropicalis        -MNI----FLETLKVLFLTIYLNLESFVLWFIPSRKKNVAGEIVLITGAGSGIGRLMALE
SDR16C5 D. rerio (a)         -MNI----LLETLRLIFLTVYYNLEAFLKFFIPLRKKDVSGEIVLITGSGSGIGRLMALE
SDR16C5 D. rerio (b)         -MNF----LLETLRVLFLSLVLGLEAFVRLFIPPRRKNVSGELVLLTGAGSGIGRLMALE
                                                                           **   * *

SDR16C1 H. sapiens           FAERGARkiVLWGRTEKCLKETTEEIRQMG--------------------------TECH  1p.0
SDR16C1 P. troglodytes       FAERGARkiVLWGRTEKCLKETTEEIRQMG--------------------------TECH  1p.0
SDR16C1 M. musculus          FAERGARkiVLWGRTEKCLKETTEEIRQMG--------------------------TECH  1p.0
SDR16C1 G. japonicus         FAKRGARkvILWGRTEKCLKETTEEIRVMG--------------------------TECY  1p.0
SDR16C1 G. gallus            FARRGARkiILWGRTEKCLKETTEEIRMMG--------------------------TECH  1p.0
SDR16C1 D. rerio             FAKQGARkvILWGRTEKCLKETCEEISMTG--------------------------TECH  1p.0
SDR16C1 X. tropicalis        FAKQNAKkiILWGRTERCLKETTEEIKQMG--------------------------TDCS  1p.0
SDR16C2 H. sapiens           FAKLKSK-LVLWDINkhGLEETAAKCKGLG--------------------------AKVH  1p.0
SDR16C2 P. troglodytes       FAKLKSK-LVLWDINkhGLEETAAKCKGLG--------------------------AKVH  1p.0
SDR16C2 M. musculus          FAKLNTK-LVLWDINknGIEETAAKCRKLG--------------------------AQAH  1p.0
SDR16C2 S. harrisii          FAKLKSK-LVLWDINkhGIEETAAECRKLG--------------------------ARAH  1p.0
SDR16C2 G. gallus            FARRQSR-LVLWDVEahGLKETATECEGLG--------------------------ATVH  1p.0
SDR16C2 A. mississippiensis  FAQHRSR-LVLWDVNqhGIEETAAECQRLG--------------------------ATVH  1p.0
```

```
SDR16C2 X. tropicalis        FGELESV-LVLWDINkqGVEETAEKCRKGG-------------------------AKVY  1p.0
SDR16C3 H. sapiens           FAKRQSI-LVLWDINkrGVEETAAECRKLG-------------------------VTAH  1p.0
SDR16C3 P. troglodytes       FSKQQSI-LVLWDINkrGVEETAAECRKLG-------------------------VTAH  1p.0
SDR16C3 M. musculus          FAKQKSR-LVLWDINkrGVEETADKCRKLG-------------------------AVVH  1p.0
SDR16C3 S. harrisii          FAKHKSK-LVLWDINkhGIEETAAECRKLG-------------------------APTH  1p.0
SDR16C3 F. albicollis        FARRQSR-LVLWDINkhGVEETAAECQKLG-------------------------ATVQ  1p.0
SDR16C3 A. carolinensis      FAKRQSV-LVLWDINkqGVEETAEECRKLG-------------------------AIAH  1p.0
SDR16C3 X. tropicalis        FAKHESI-LVLWDINqkGVEETADECRKLG-------------------------ATAY  1p.0
SDR16C4 H. sapiens           FARRRAL-LVLWDINTQSNEETAGMVRHIYRDLEAAD-AAALqaGNGEEEILPHCNLQVF  1p.2
SDR16C4 P. troglodytes       FARRRAL-LVLWDINTQSNEETAGMVRHIYRDLEAAD-AAALqaGNGEEEILPHCNLQVF  1p.2
SDR16C4 M. musculus          FARRRAL-LVLWDINTQSNEETAGMVRHIYRDLEAAD-AAALqaGKGEEEILPPCNLQVF  1p.2
SDR16C4 M. domestica         FARRRAL-LVLWDINTQSNEETAGMVRHIYQDLEAAD-AAALqaGNGEEEVLPPCNLQVF  1p.2
SDR16C4 G. gallus            FARRRAL-LVLWDINTQSNEETAGMVRHIYRELAE---AAPkvAGDGEKDALPHCSLQVY  1p.2
SDR16C4 G. japonicus         FARRRAR-LVLWDINTQSNEAQAGMVRHIYREVAEEAAAAAQkaGHGEEEVLPHYNLQVH  1p.2
SDR16C4 X. tropicalis        FARRRAQ-LVLWDINSQSNEETAEMVRSIYRELEAEDSAR-raGNATEEEVQPCCNFQVY  1p.2
SDR16C4 P. latipinna         FARRRAV-LVLWDINSQSNEETAEMVRQIYHETETPIA---KdgPVGGVEEVPAFQPQVY  1p.2
SDR16C5 H. sapiens           FARLGSV-LVLWDINKEGNEETCKMAREAG------------------------ATRVH
SDR16C5 P. troglodytes       FARLGSV-LVLWDINKEGNEETCKMAREAG------------------------ATRVH
SDR16C5 M. musculus          FARLGAV-LVLWDVNKEANDETHQLAREAG------------------------AARVH
SDR16C5 M. domestica         FAHLGAT-LVLWDINPEGNQETSKLAKEAG------------------------ASRVY
SDR16C5 G. gallus            FAKLGAT-LVLWDINQDGLKETLRLAEENG------------------------AVRIH
SDR16C5 P. bivittatus        FARLGAV-LVLWDVNLEGNKETARLACKIG------------------------AARVH
SDR16C5 X. tropicalis        FAHLGAT-LVLWDINEEGNKETCRLAKKNG------------------------AVRVH
SDR16C5 D. rerio (a)         FASLDVS-LVLWDINVDGLKETAEQVKEKG------------------------ASRVH
SDR16C5 D. rerio (b)         FARLDAR-LVLWDINEDGNKETARLIKEKY------------------------GARAH
                             *          **

SDR16C1 H. sapiens           YFICDVGNREEVYQTAKAVREkvGDITILVNNAAVVHGKSLMDSDDDALLKSQHINTLGQ  2p.0
SDR16C1 P. troglodytes       YFICDVGNREEVYQTAKAVREkvGDITILVNNAAVVHGKSLMDSDDDALLKSQHINTLGQ  2p.0
SDR16C1 M. musculus          YFICDVGNREEVYQMAKAVREkvGDITILVNNAAVVHGKSLMDSDDDALLKSQHVNTLGQ  2p.0
SDR16C1 G. gallus            YFICDVGNREEVYRQAKAVREkvGDITILVNNAAVVHGKSLMDSDDDALLKSQHINTLGQ  2p.0
SDR16C1 G. japonicus         YFICDVGNREEVYQQAKAVREkvGDITILVNNAAVVHGRSLMESDDDALLKSQHINTLGQ  2p.0
SDR16C1 X. tropicalis        YFVCDVGNREEVYQQAKAVREkvGDVTILVNNAAVVHGKSLMDSDDDALLKSQHINTLGQ  2p.0
SDR16C1 D. rerio             YFVCDVGNREEVYQQAKVLREkvGDVTILVNNAAVVHGKSLMESDDDALLKTQHINTLGQ  2p.0
SDR16C2 H. sapiens           TFVVDCSNREDIYSSAKkvKAEIGDVSILVNNAGVVYTSDLFATQDPQIEKTFEVNVLAH  2p.0
SDR16C2 P. troglodytes       TFVVDCSNREDIYSSAKkvKAEIGDVSILVNNAGVVYTSDLFATQDPQIEKTFEVNVLAH  2p.0
SDR16C2 M. musculus          PFVVDCSQREEIYSAAKkvKEEVGDVSILVNNAGVVYTADLFATQDPQIEKTFEVNVLAH  2p.0
SDR16C2 S. harrisii          AYVVDCSNKEDIYNYAKkvKAEVGDVSILVNNAGVVYTADLFSTQDPQIEKTFEVNVLAH  2p.0
SDR16C2 G. gallus            TLVVDCSKREEIYSAAEkvKKDIGDVSILVNNAGVITAADLLSTQDHQIEKMFEVNILAH  2p.0
SDR16C2 A. mississippiensis  TFVVDCSKREEIYRAAEkvKKEIGDISILVNNAGVITPADLLSTQDHQIEQTFAVNILAH  2p.0
SDR16C2 X. tropicalis        TYVVDCSKREEINTAANkvKQEVGDVTILINNAGIIFCADVLTLQDQQIEKIFEVNILAH  2p.0
SDR16C3 H. sapiens           AYVVDCSNREEIYRSLNqvKKEVGDVTIVVNNAGTVYPADLLSTKDEEITKTFEVNILGH  2p.0
SDR16C3 P. troglodytes       AYVVDCSNREEIYRSLNqvKKEVGDVTIVVNNAGTVYPADLLSTKDEEITKTFEVNILGH  2p.0
SDR16C3 M. musculus          VFVVDCSNRAEIYNSVDqvKREVGDVEIVVNNAGAIYPADLLSAKDEEITKTFEVNILGH  2p.0
SDR16C3 S. harrisii          AFVVDCSKKEEIYSMIDkiKKEVGDVTIVVNNAGTIYPADLLSTKDEEITKTFEVNILGH  2p.0
SDR16C3 F. albicollis        TFVVDCSKREEIYSTADkvKKDIGDVTILVNNVGIITAADFLSTQDHQIEKMFEVNILGH  2p.0
SDR16C3 A. carolinensis      ALVVNCKNREEIYTVADkvKKDIGDVSILVNNAGVITTAKLLSTKDEQIQEMFDVNVLAH  2p.0
SDR16C3 X. tropicalis        AFVVDCSTRNDIYRCAEkvKQDIGDVDILINNAGVVFGTEFLKLQDHQIEKTFSVNILAH  2p.0
SDR16C4 H. sapiens           TYTCDVGKRENVYLTAERVRKEVGEVSVLVNNAGVVSGHHLLECPDELIERTMMVNCHAH
SDR16C4 P. troglodytes       TYTCDVGKRENVYLTAERVRKEVGEVSVLVNNAGVVSGHHLLECPDELIERTMMVNCHAH
SDR16C4 M. musculus          TYTCDVGKRENVYLTAERVRKEVGEVSVLVNNAGVVSGHHLLECPDELIERTMMVNCHAH
SDR16C4 M. domestica         TYTCDVGKRENVYLTAERVRKEVGEVSVLVNNAGVVSGHHLLECPDELIERTMMVNCHAH
SDR16C4 G. gallus            TYTCDVGKRENVYTTAERVRKEVGEVSVLVNNAGVVSGHHLLECPDELIERTMMVNCHAH
SDR16C4 G. japonicus         TYTCDVSKRENVYTTAERVRKEVGEVSVLVNNAGVVSGHHLLECPDELIERTMMVNCHAH
SDR16C4 X. tropicalis        TYTCDVGKRESVYSTAERVRREVGDVYLLLNNAGVVSGHHLLECPDELIERTMMVNCHAH
SDR16C4 P. latipinna         TYVCDVGKRESVYSTAEKVRREVGEVDILINNAGVVSGHHLLECPDELIERTMVVNCHAH
```

```
SDR16C5 H. sapiens           AYTCDCSQKEGVYRVADqvKKEVGDVSILINNAGIVTGKKFLDCPDELMEKSFDVNFKAH  1p.0
SDR16C5 P. troglodytes       AYTCDCSQKEGVYRVADqvKKEVGDVSILINNAGIVTGKKFLDCPDELMEKSFDVNFKAH  1p.0
SDR16C5 M. musculus          AYTCDCSRREEVYRVADqvKKEVGDVSILINNAGIVTGRNFLDCPDDLMEKSFDVNFKAH  1p.0
SDR16C5 M. domestica         TYTCNCGQRQDVYRVADqvKKEVGDVTILINNAGVVTGRKFLEIPDECIEKAFDVNIKAH  1p.0
SDR16C5 G. gallus            SYICDCSKRQEVYRVADqvKKEVGDVSILVNNAGIVTGRSFIESPDSLVEKTMEVNTMAH  1p.0
SDR16C5 P. bivittatus        EYICDCSKRQEVYQVADqvKKEVGDVSILINNAGIITGQMFLDTPDMLLEKSIQVNTMAH  1p.0
SDR16C5 X. tropicalis        AYLCDCSKRQEVYKVADqvKKEVGDVSILINNAGIVTGKKFIDSPDALVEKTMQVNCMAH  1p.0
SDR16C5 D. rerio (a)         YYQCDCSDREAVYRVADqvKSEIGDVTILINNAGIVSGKKFMDTPDALIEKTLRVNAMSH  1p.0
SDR16C5 D. rerio (b)         TYTCDCSDREEVYRVANqvKREVGDVTILINNAGIVTGKKFMDSPDSLIEKSMEVNSLAH  1p.0
                                                    *      **             *         *

                                                                 +   +
SDR16C1 H. sapiens           FwtTKAFLPRMLELQNGHIVCLNSVLALSAIPGAIDYCTSKASAFAFMESLTLGL--LDC  3p.0
SDR16C1 P. troglodytes       FwtTKAFLPRMLELQNGHIVCLNSVLALSAIPGAIDYCTSKASAFAFMESLTLGL--LDC  3p.0
SDR16C1 M. musculus          FwtTKAFLPRMLELQNGHIVCLNSVLALSAIPGAIDYCTSKASAFAFMESLTLGL--LDC  3p.0
SDR16C1 G. gallus            FwtTKAFLPRMLELQNGHIVCLNSVLALSAIPGAIDYCTSKASSFAFMESLTLGL--LDC  3p.0
SDR16C1 G. japonicus         FwtTKAFLPRMLELQNGHIVCLNSVLALSAIPGAIDYCTSKASSFAFMESLTLGL--LDC  3p.0
SDR16C1 X. tropicalis        FwtTKAFLPRMLELQNGHIVCINSVLALSAIPGAIDYCTSKASSFAFMESLTLGL--LDC  3p.0
SDR16C1 D. rerio             FwtTKAFLPRMLELCNGHVVCINSILSLSSIPGAIDYCTSKASSYAFMESLTLGL--LDC  3p.0
SDR16C2 H. sapiens           FwtTKAFLPAMTKNNHGHIVTVASAAGHVSVPFLLAYcsSKFAAVGFHKTLTDELAALQI  3p.0 4p.1
SDR16C2 P. troglodytes       FwtTKAFLPAMTKNNHGHIVTVASAAGHVSVPFLLAYcsSKFAAVGFHKTLTDELAALQI  3p.0 4p.1
SDR16C2 M. musculus          FwtTKAFLPVMMKNNHGHIVTVASAAGHTVVPFLLAYcsSKFAAVGFHRALTDELAALGR  3p.0 4p.1
SDR16C2 S. harrisii          FwtTKAFLPAMMKNNHGHIVTVASAAGHTVVPFLLAYcsSKFAAVGFHRALTAEMDALEK  3p.0 4p.1
SDR16C2 X. tropicalis        FwtTRAFLPSMLRNNHGHIVTVASSAGFVGVQFMVDYcsTKFAALGYHKALTAELLALGK  3p.0 4p.1
SDR16C2 G. gallus            IwtTRAFLPTMMKNNHGHIVTVASAAGQFVTSFMVAYcsSKFAAVGFHKALTEELSSLGK  3p.0 4p.1
SDR16C2 A. mississippiensis  FwtTKAFLPTMMKNNHGHIVTVSSAAGHMPASFMLSYcsSKFAAVGFHKALTQELSALGK  3p.0 4p.1
SDR16C3 H. sapiens           FwiTKALLPSMMERNHGHIVTVASVCGHEGIPYLIPYcsSKFAAVGFHRGLTSELQALGK  3p.0 4p.1
SDR16C3 P. troglodytes       FwiTKALLPSMMERNHGHIVTVASVCGHEGIPYLIPYcsSKFAAVGFHRGLTSELQALEK  3p.0 4p.1
SDR16C3 M. musculus          FwiIKALLPSMLRRNSGHIVTVASVCGHGVIPYLIPYcsSKFAAVGFHRALTAELDTLGK  3p.0 4p.1
SDR16C3 S. harrisii          FwiIKALLPPMMKRNHGHIITVASICGHGVIPYLIPYcsSKFAAVGFHRALTSELAAMGK  3p.0 4p.1
SDR16C3 F. albicollis        MwtTRAFLPVMMNNNYGHIVTVASAAGHFVVPYMVTYcsSKFAAVGFHKALTEELSALGK  3p.0 4p.1
SDR16C3 A. carolinensis      YwtTKAFLPAMIKNNHGHIVTVASISGHIGIPFTVTYtsSKFAAVGFHNGLKEELRFLRK  3p.0 4p.1
SDR16C3 X. tropicalis        FwtTKSFLSAMMKKDRGHIVTVASIAGQLGVPYLVDYcaSKFGLVGFHESLTSELKLLGK  3p.0 4p.1
SDR16C4 H. sapiens           FwtTKAFLPTMLEINHGHIVTVASSLGLFSTAGVedYCASKFGVVGFHESLSHELKAAEK  2p.0 3p.0
SDR16C4 P. troglodytes       FwtTKAFLPTMLEINHGHIVTVASSLGLFSTAGVedYCASKFGVVGFHESLSHELKAAEK  2p.0 3p.0
SDR16C4 M. musculus          FwtTKAFLPTMLEINHGHIVTVASSLGLFSTAGVedYCASKFGVVGFHESLSHELKAAEK  2p.0 3p.0
SDR16C4 M. domestica         FwtTKAFLPTMLEINHGHIVTVASSLGLFSTAGVedYCASKFGVVGFHESLSHELKAAEK  2p.0 3p.0
SDR16C4 G. japonicus         FwtAKAFLPKMLELNHGHIVTVASSLGLFSTAGVedYCASKFGAVGFHESLSHELKAAEK  2p.0 3p.0
SDR16C4 G. gallus            FwtTKAFLPKMLEMNHGHIVTVASSLGLFSTAGVedYCASKFGAVGFHESLSHELKAAEK  2p.0 3p.0
SDR16C4 X. tropicalis        FwtTKAFLPKMMEMNHGHIVSVASSLGLFSTAGVedYCASKFGVVGFHESLSHELKAADK  2p.0 3p.0
SDR16C4 P. latipinna         FwtTKAFLPKMLELNHGHIVTVASSLGLFSTAGVedYCASKFGAIGFHESLSHEIKASEK  2p.0 3p.0
SDR16C5 H. sapiens           LwtYKAFLPAMIANDHGHLVCISSSAGLSGVNGLadYCASKFAAFGFAESVFVETFVQKQ  2p.0 3p.2
SDR16C5 P. troglodytes       LwtYKAFLPAMIANDHGHLVCISSSAGLSGVNGLadYCASKFAAFGFAESVFVETFVQKQ  2p.0 3p.2
SDR16C5 M. musculus          LwmYKAFLPAMIANNHGHLVCISSSAGLIGVNGLsdYCASKFAALGFAESMFIETLAKKQ  2p.0 3p.2
SDR16C5 M. domestica         FwiYKAFLPAMMANNHGHLVCISSSAGLLGVNKLsdYCASKFAAFGFAESIFLELHAERK  2p.0 3p.2
SDR16C5 G. gallus            FwtYKAFLPAMIASNHGHLVSIASSAGLIGVNRLadYCASKFAAVGFAESMSSEMRAQGK  2p.0 3p.2
SDR16C5 P. bivittatus        FwtVKAFLPAMVASNHGHVVTISSAAGFNGVNKMadYCTSKFAVLGFAESLASEMLAMKK  2p.0 3p.2
SDR16C5 X. tropicalis        FwtYKAFLPAMMASNHGHLVSIASSAGLIGVNGLadYCASKFAAVGFAESVGLEMLALGK  2p.0 3p.2
SDR16C5 D. rerio (a)         FwtYKAFLPAMMDKNHGHLVSVASSAGLIGVNGLadYCASKFAAVGFAESVALELLSAGK  2p.0 3p.2
SDR16C5 D. rerio (b)         FwtYKAFLPAMIAGNHGHLVSIASSAGLIGVNGLadYCASKFAAVGFAESMGLELLATGC  2p.0 3p.2
                              *     *  *     **     *            *   *

SDR16C1 H. sapiens           PGVSATTVLPFHTSTEMFQGMRVrfPN--LFPPLKPETVARRTVEAVQLNQALLLLPWTM  4p.1
SDR16C1 P. troglodytes       PGVSATTVLPFHTSTEMFQGMRVrfPN--LFPPLKPETVARRTVEAVQLNQALLLLPWTM  4p.1
SDR16C1 M. musculus          PGVSATTVLPFHTSTEMFQGMRVrfPN--LFPPLKPETVARRTVDAVQQNQALLLLPWTM  4p.1
SDR16C1 G. gallus            PGVNATTVLPFHTSTEMFQGMRIrfPN--LFPPLKPETVARRTVEAVQMNQAFLLLPWTM  4p.1
```

```
SDR16C1 G. japonicus         PGVNATTVLPFHTSTEMFQGMRIrfPN--LFPPLKPEVVARRTVEAVQQNQAFLLLPWTM  4p.1
SDR16C1 X. tropicalis        PGVNATTVLPFHTNTEMFQGMRVrfPN--LFPPLKPETVATKTVEAVQKNKAFLLLPWTM  4p.1
SDR16C1 D. rerio             PGVGCTTVLPFHTDTEMFQGMRVrfPK--LFPPLNPEMVAERTVDAVRTNTAFVVLPWTM  4p.1
SDR16C2 H. sapiens           TGVKTTCLCPNFVNTGFIKNPSTslGP-----TLEPEEVVNRLMHGILTEQKMIFIPSSI  5p.1
SDR16C2 P. Troglodytes       TGVKTTCLCPNFVNTGFIKNPSTslGP-----TLEPEEVVNRLMHGILTEQKMIFIPSSI  5p.1
SDR16C2 M. musculus          TGVRTSCLCPNFINTGFIKNPSTnlGP-----TLEPEEVVEHLMHGILTEKQMIFVPSSI  5p.1
SDR16C2 S. harrisii          NGIKTSCLCPNFINTGFIKNPNSrfLP-----TLEPEEVVNKLMKGILTEQKMIFVPSSL  5p.1
SDR16C2 G. gallus            DGIKTTCLCPVFMNTGFVKNPSTrlGK-----ILEVDEVVKALMEGILTNQKMVFVPPQL  5p.1
SDR16C2 A. mississippiensis  DGIKTTCLCPIFINTGFVKNPKMrlTP-----ILEPDMVAKKLLEGILINQKMIFVPSSL  5p.1
SDR16C2 X. tropicalis        SGIKTSCLCPVFVDTGFVKNPSLrlAP-----VLQPEEVAQTLVDGILINKKMICVPSSV  5p.1
SDR16C3 H. sapiens           TGIKTSCLCPVFVNTGFTKNPSTrlWP-----VLETDEVVRSLIDGILTNKKMIFVPSYI  5p.1
SDR16C3 P. troglodytes       TGIKTSCLCPVFVNTGFTKNPSTrlWP-----VLETDEVVRSLIDGILTNKKMIFVPSYT  5p.1
SDR16C3 M. musculus          TGIQTSCLCPVFVNTGFTKNPSTrlWP-----VLEPEEVARSLINGILTNKKMIFVPSYI  5p.1
SDR16C3 S. harrisii          NGIKTSCLCPVFVNTGFTKNPSTrlWP-----ILETDDVVRKLMDGILTDKKMIFVPSYL  5p.1
SDR16C3 F. albicollis        DGIKTTCLCPVFINTGFVKNPSTrlGK-----ILEIEEVVETLMEGIVTNKKMVFVPPNQ  5p.1
SDR16C3 A. carolinensis      DGIQMTCLCPSIINTGFLKISKTliLP-----VLKAEDTAKDLMEGILTNQKIVFSPPWA  5p.1
SDR16C3 X. tropicalis        DGVKTTCLCPVFVNTGFVQNPSTrvWP-----VLKTEDVVKCLMEGILTNKKMIIVPSSV  5p.1
SDR16C4 H. sapiens           DGIKTTLVCPYLVDTGMFRGCRIrkEIEPFLPPLKPDYCVKQAMKAILTDQPMICTPRLM  4p.1
SDR16C4 P. troglodytes       DGIKTTLVCPYLVDTGMFRGCRIrkEIEPFLPPLKPDYCVKQAMKAILTDQPMICTPRLM  4p.1
SDR16C4 M. musculus          DGIKTTLVCPYLVDTGMFRGCRIrkEIEPFLPPLKPDYCVKQAMRAILTDQPMVCTPRLM  4p.1
SDR16C4 G. gallus            DGIKTTLVCPYLVDTGMFRGCRIrkEIEPFLPPLKPEYCVKQAMRAILTDQPMICTPRLM  4p.1
SDR16C4 M. domestica         DGIKTTLVCPYLVDTGMFRGCRIrkEIEPFLPPLKPDYCVKQAMKAILTDQPMICTPRLM  4p.1
SDR16C4 G. japonicus         DGIKTTLVCPYLVDTGMFRGCRIrkEIEPFLPPLKPDYCVKQAMRAILTDQPMICTPRLM  4p.1
SDR16C4 X. tropicalis        DGIKTTLVCPYLVDTGMFRGCRIrkEIEPFLPPLKPDYCVKQAMRAILTDQPMICTPRLM  4p.1
SDR16C4 P. latipinna         DGINMTLVCPYLVNTGMFKGCRIrkEIEPFLPPLSPDFCVKQAMRAILTDQPMVCTPRII  4p.1
SDR16C5 H. sapiens           KGIKTTIVCPFFIKTGMFEGCTTgcPS--LLPILEPKYAVEKIVEAILQEKMYLYMPKLL  4p.1
SDR16C5 P. troglodytes       KGIKTTIVCPFFIKTGMFEGCTTgcPS--LLPILEPKYAVEKIVEAILQEKMYLYMPKLL  4p.1
SDR16C5 M. musculus          WGIKTTIVCPFFIKTGMFEGCTTkcPT--LLPILDPEYAVRKIIDAILQEQLYLYMPKFL  4p.1
SDR16C5 M. domestica         TGIKTTIVCPFFIKTGMFEGCTTanSH--LLPILEPKYVVDKIMDAILTEQVYLYLPKFL  4p.1
SDR16C5 G. gallus            TGVKTTTVCPYFINTGMFNGCSTkvSL--LLPILEPEYVAEKTMTAIRRDEPILLLPRSL  4p.1
SDR16C5 P. bivittatus        NGIKSTIVCPYLVNTGMFDGCETkwPC--LLPIINPEYAAERIVSGILRNERYILMPRVL  4p.1
SDR16C5 X. tropicalis        TGIKTTIVCPYFINTGMFDGCSTkvPR--LLPILEAEYASKKIVDAILKDQVYLVMPRSL  4p.1
SDR16C5 D. rerio (a)         DGIKTTIVCPFLINTGLFDGCGTkwPL--LMPMLEPDYVAKRIVSAILTDQVFVLLPRSL  4p.1
SDR16C5 D. rerio (b)         DGVKTTIVCPFFINTGMFDGANTkwPR--LMPILDPDYACRKIVDAIRREQVYLYMPRSI  4p.1
                              *       *    *                                         *

SDR16C1 H. sapiens           HALVILKsiLPQAALEEIHKFSGTYTCMNTFKGRT-------------  5p.1
SDR16C1 P. troglodytes       HALVILKsiLPQAALEEIHKFSGTYTCMNTFKGRT-------------  5p.1
SDR16C1 M. musculus          NILIIILKsiLPQAALEEIHRFSGTYTCMNTFKGRT-------------  5p.1
SDR16C1 G. japonicus         HILIIILKslLPQAALEEIHKFSGSYTCMNTFKGRT-------------  5p.1
SDR16C1 G. gallus            NVLVILKsiLPQAALEEIHKFSGSYTCMNTFKGRT-------------  5p.1
SDR16C1 X. tropicalis        HALVILKslLPQSALEEIHRFSGAYTCMNTFKGRT-------------  5p.1
SDR16C1 D. rerio             HFLVILKslLPQSALEEIHKFSGSYTCMNTFKGRT-------------  5p.1
SDR16C2 H. sapiens           AFLTTLEriLPERFLAVLKRKISVK-F-DAVIGYKMKAQ---------  6p.1
SDR16C2 P. troglodytes       AFLTTLEriLPERFLAVLKRKISVK-F-DAVIGYKMKAQ---------  6p.1
SDR16C2 M. musculus          ALLTVLEriVPERFLQVLKHRINVK-F-DAVVGYKDK-----------  6p.1
SDR16C2 S. harrisii          SVITLLEriFPERFQKALRRIFNIK-F-DAVIAFKNKTQ---------  6p.1
SDR16C2 G. gallus            SFALLSEmlIPERASNVLKKLTDVK-F-DAVVGHGSNQ----------  6p.1
SDR16C2 X. tropicalis        SLVPVLAffLPERALNALNEFQNLK-F-EAKVHSRDKDK---------  6p.1
SDR16C2 A. mississippiensis  NIFLFMEkiLPERALAAWNKLQAIQ-F-DKVVGYRNKE----------  6p.1
SDR16C3 H. sapiens           NIFLRLQkfLPERASAILNRMQNIQ-F-EAVVGHKIKMK---------  6p.1
SDR16C3 P. troglodytes       NIFLRLQkfLPERASAILNRMQNIQ-F-EAVVGHKIKMK---------  6p.1
SDR16C3 M. musculus          NISLILEkfLPERALKAISRIQNIQ-F-EAIVGHKTKMK---------  6p.1
SDR16C3 S. harrisii          NFCLVLEkfLPERALAAINRVQNIQ-F-EAVVSYKHKKN---------  6p.1
SDR16C3 F. albicollis        SVALLLErvFPERALNLLKKMSEVK-F-DAVIGQRSTQ----------  6p.1
SDR16C3 A. carolinensis     KIIVVSSkfLPERACNAILDTEKSN-Y-HKLLQ---------------  6p.1
```

```
SDR16C3 X. tropicalis        KYSLIMNqfLPERVIATMTKMQDIQ-F-STPYRCDKKED---------  6p.1
SDR16C4 H. sapiens           YIVTFMKsiLPFEAVVCMYRFLGADKCMYPFIAQRKQATNNNEAKNGI  5p.1
SDR16C4 P. troglodytes       YIVTFMKsiLPFEAVVCMYRFLGADKCMYPFIAQRKQATNNNEAKNGI  5p.1
SDR16C4 M. musculus          YIVTFMKsiLPFEAVVCMYRFLGADKCMYPFIAQRKQATNNNEAKNGI  5p.1
SDR16C4 M. domestica         YIVTFMKsiLPFEAVVCMYRFLGADKCMYPFIAQRKQATNNNEAKNGI  5p.1
SDR16C4 G. gallus            YMVTFMKsiLPFEAVVCMYRFLGADKCMYPFIAQRKQATNNNEAKNGI  5p.1
SDR16C4 G. japonicus         YMVTFMKsiLPFEAVVCMYRFLGADKCMYPFIAQRKQATNNNEAKNGI  5p.1
SDR16C4 X. tropicalis        YIVTCMKsiLPFEAVVCMYRFLGADKCMYPFIAQRKQATNNNEAKNGI  5p.1
SDR16C4 P. latipinna         YMVNFMKsiLPFEAIVCMYRFLGADKCMYPFLAQRKEAMNNNEAKNGI  5p.1
SDR16C5 H. sapiens           YFMMFLKsfLPLKTGLLIADYLGILHAMDGFVDQKKKL----------  5p.1
SDR16C5 P. troglodytes       YFMMFLKsfLPLKTVLLIADYLGILHAMDGFVDQKKKL----------  5p.1
SDR16C5 M. musculus          YFIVFLKsiLPIKTGILIADYLGVFHMTEGFTGQKKKT----------  5p.1
SDR16C5 M. domestica         YYALFLKsfLPAKSGVAVCEYMKIFDVMNSFKGQLKKF----------  5p.1
SDR16C5 G. gallus            YFFLALKniLPVKVGVLLADFVGALHFMDSFKGRAKKD----------  5p.1
SDR16C5 P. bivittatus        YFCNVMKsiLPAKMVDVLYDYFGVLQVMNRFKGRTKMTED--------  5p.1
SDR16C5 X. tropicalis        YIMFALKniMSTKLGVVLGNYFGAFHFMDHFKGRQKKE----------  5p.1
SDR16C5 D. rerio (a)         YFLMALKgvIPYKQSVILGMYFGAFNFMDAFKGREKKRD---------  5p.1
SDR16C5 D. rerio (b)         YIIIGLRnlLPTKVGVLLGEYLGAFNFMAKFKGHGQKSD---------  5p.1
```

Figure S18. Alignment of the vertebrate SDR16C family variants. For further details see Fig. S13.

* symbol marks the position of identical amino acid residues of the aligned SDR protein sequences.

```
                                   ×   × ×
SDR21C1 H. sapiens      MSSGIHVALVTGGNKGIGLAIVRDLCRLFSGDVVLTARDVTRGQAAVQQLQ-AEGLSPRF
SDR21C1 P. troglodytes  MSSGIHVALVTGGNKGIGLAIVRDLCRLFSGDVVLTARDVTRGQAAVQQLQ-AEGLSPRF
SDR21C1 M. musculus     MSSSRPVALVTGANKGIGFAITRDLCRKFSGDVVLAARDEERGQTAVQKLQ-AEGLSPRF
SDR21C1 M. domestica    MSSSSRVAVVTGSNKGIGFAIVRDLCQKFSGDVILTSRDTTRGQAATKKLQ-EEGLNPIF
SDR21C1 G. gallus       -MSNVPVAVVTGSNKGIGLAIVRDLCKQFKGDVYLTARDPARGQGAVAKLQ-EEGLHPLF
SDR21C1 C. picta        -MSSTPVAVVTGGNKGIGFAIVRALCKQFTGDVYLTARDPGRGQVAVTKLQ-QEGLNPLF
SDR21C1 X. tropicalis   -MASAKVAVVTGGNKGIGLAIVRALCKQFKGDVYLTARDPKLGEEAVRALKEQEGLSPHF
SDR21C1 D. rerio        -MSQCKVALVTGANKGIGFAIVRALCKEYTGDVYLSSRDVGRGTAAVDSLK-KEGLHPLF
SDR21C2 H. sapiens      MSSCSRVALVTGANRGIGLAIARELCRQFSGDVVLTARDVARGQAAVQQLQ-AEGLSPRF
SDR21C2 P. troglodytes  MSSCSRVALVTGANRGIGLAIARELCRQFSGDVVLTARDVARGQAAVQQLQ-AEGLSPRF
SDR21C2 M. musculus     MSSCSRVALVTGANKGIGFAITRDLCRKFSGDVVLTARDEARGRAAVQQLQ-AEGLSPRF
SDR21C2 M. domestica    MSSSSRVAVVTGSNKGIGFAIVRDLCQKFSGDVILTSRDPTRGQEAVKELQ-EEGLNPIF
SDR21C2 X. tropicalis   -MASAKVAVVTGGNKGIGLAIVRALCKQFKGDVYLTARDPKLGEEAVRALKEQEGLSPHF
                              ** *** * *** ** * **    *** *  **   *  *   *   *** * *

SDR21C1 H. sapiens      HQLDIDDLQSIRALRDFLRKEYGGLDVLVNNAGIAFkvADPTPFHIQAEVTMKTNFFGTR  1p.2
SDR21C1 P. troglodytes  HQLDIDDLQSIRALRDFLRKEYGGLDVLVNNAGIAFkvADPTPFHIQAEVTMKTNFFGTR  1p.2
SDR21C1 M. musculus     HQLDIDNPQSIRALRDFLLKEYGGLDVLVNNAGIAFkvNDDTPFHIQAEVTMKTNFFGTR  1p.2
SDR21C1 G. gallus       HQLDIDDLQSIKVLRDFLKEKYGGLNVLVNNAGIAFkvSDRTPFAVQAEVTLKTNFFGTR  1p.2
SDR21C1 C. picta        HQLDIEDLQSIRTLQDFLKEKYGGLNVLVNNAGIAFkvADTTPFAIQAEVTMRTNFFATR  1p.2
SDR21C1 M. domestica    HQLDIDDPQSIRTLRDFLKERYGGVDVLVNNAGIAFkvADPTPFPIQAEVTMKTNFFGTK  1p.2
SDR21C1 X. tropicalis   HQLDINDLQSIRALGSFLKEKYGGIDVLINNAGIAFkgTDPTPFGTQANVTLQTNFFATR  1p.2
SDR21C1 D. rerio        HQLDINDPNSVRTARDFFQEKYGGLDVLINNAGIAFkmADTTPFGTQADVTLKTNFFATR  1p.2
SDR21C2 H. sapiens      HQLDIDDLQSIRALRDFLRKEYGGLNVLVNNAAVAFksDDPMPFDIKAEMTLKTNFFATR  1p.2
SDR21C2 P. troglodytes  HQLDIDDLQSIRALRDFLRKEYGGLNVLVNNAAVAFksDDPMPFDIKAEMTLKTNFFATR  1p.2
SDR21C2 M. musculus     HQLDIDDPQSIRALRDFLRKEYGGLNVLVNNAGIAFrmDDPTPFDIQAEVTLKTNFFATR  1p.2
SDR21C2 M. domestica    HQLDIDDPQSIRTLRDFLKERYGGVDVLVNNAGIAFkvTDTTPFPIQAEVTMKTNFFGIK  1p.2
SDR21C2 X. tropicalis   HQLDINDLQSIRALGGFLKEKYGGIDVLINNAGIAFkvADTTPFGTQAEVTLKTNFFATR  1p.2
                        *****    *      *    ***  ** ***  **   *  **   *  *  ****

SDR21C1 H. sapiens      DVCTELLPLIKPqgRVVNVSSIMSVRALKSCSPELQQKFRSETITEEELVGLMNKFVEDT  2p.2
SDR21C1 P. troglodytes  DVCTELLPLIKPqgRVVNVSSIMSVRALKSCSPELQQKFRSETITEEELVGLMNKFVEDT  2p.2
SDR21C1 M. musculus     DVCKELLPLIKPqgRVVNVSSMVSLRALKNCRLELQQKFRSETITEEELVGLMNKFVEDT  2p.2
SDR21C1 G. gallus       NICTELLPLIKPygRVVNVSSMVSISALGGCSQELQKKFRSDTITEDELVELMTKFVEDT  2p.2
SDR21C1 C. picta        DVCTELLPLIKPhgRVVNVSSMGSVSALARCSRDLQQKFRSDTITEEELVKLMTKFVEDT  2p.2
SDR21C1 X. tropicalis   DVCNELLPQVRPqgRVVNVSSMLSSSALQGCSPELQKVFRSDTITEEELVTLMEKFVEDA  2p.2
SDR21C1 D. rerio        DMCNVFLPIIKPggRLVNVSSGMGSMALGRCSPELQARFRSDITEEELNGLMERFVREA  2p.2
SDR21C2 H. sapiens      NMCNELLPIMKPhgRVVNISSLQCLRAFENCSEDLQERFHSETLTEGDLVDLMKKFVEDT  2p.2
SDR21C2 P. troglodytes  NMCNELLPIMKPhgRVVNISSLQCLRAFENCSEDLQERFHSETLTEGDLVDLMKKFVEDT  2p.2
SDR21C2 M. musculus     NVCTELLPIMKPhgRVVNISSLQGLKALENCREDLQEKFRCDTLTEVDLVDLMKKFVEDT  2p.2
SDR21C1 M. domestica    AVSAELLPLVKPrgRVVNVSSMVSLRSLKSCSPELQQKFRSDTITEEELVRLMEKFVEDT  2p.2
SDR21C2 M. domestica    AVSAELLPLVKPggRVVNISSMMSLRALEGCSPELQQKFRSDTITEEELVRLMEKFVEDT  2p.2
SDR21C2 X. tropicalis   DACHELLPLIKPrgRVVNVSSMASYMALGRCSQELQKVFRSDTITEEELVTLMEKFVEDA  2p.2
                              **   * ** ** **         *   **  *     **  *  **  **
                                      +   +
SDR21C1 H. sapiens      KKGVHQKEGWPSSAYGVTKIGVTVLSRIHARKLSEQRKGDKILLNACCPGWVRTDMAGPK
SDR21C1 P. troglodytes  KKGVHQKEGWPSSAYGVTKIGVTVLSRIHARKLSEQRKGDKILLNACCPGWVRTDMAGPK
SDR21C1 M. musculus     KKGVHAEEGWPNSAYGVTKIGVTVLSRILARKLNEQRRGDKILLNACCPGWVRTDMAGPK
SDR21C1 M. domestica    KKGVHQKEGWPNSAYGVTKIGVTVLSRIHARQLNEQRKGDKILLNACCPGWVRTDMAGPK
SDR21C1 G. gallus       KKSVHEKEGWPNTAYGVSKIGVTVLSRIQARMLNEKRKGDHILLNACCPGWVRTDMAGPK
SDR21C1 C. picta        KNGVHEKEGWPNTAYGVTKIGVTVLSRIQARMLNKERKADRILLNACCPGWVRTDMAGPN
SDR21C1 X. tropicalis   KKGAHQKEGWPNTAYGVSKVGVTVLSRIQARELNEKRKDDGILLNACCPGWVRTDMAGPK
SDR21C1 D. rerio        QEGVHSERGWPSTAYGISKTGLTTLTRIQARNLTKERPGDGILCNACCPGWVRTDMAGPN
SDR21C2 H. sapiens      KNEVHEREGWPNSPYGVSKLGVTVLSRILARRLDEKRKADRILVNACCPGPVKTDMDGKD
```

```
SDR21C2 P. troglodytes  KNEVHEREGWPNSPYGVSKLGVTVLSRILARHLDEKRKADRILVNACCPGPVKTDMDGKD
SDR21C2 M. musculus     KNEVHEREGWPDSAYGVSKLGVTVLTRILARQLDEKRKADRILLNACCPGWVKTDMARDQ
SDR21C2 M. domestica    KKGVHQKEGWPNSAYGVTKIGVTVLSRIHARQLNEQRKGDKILLNACCPGWVRTDMAGPK
SDR21C2 X. tropicalis   KKGAHQKEGWPNTAYGVSKIGVTVLSRIQARELNEKRKDDGILLNACCPGWVRTDMAGPN
                            *   ***   **  * * * * ** ** *   *  * ** ****** * ***

SDR21C1 H. sapiens      ATKSPEEGAETPVYLALLPPDAEGPHGQFVSEKRVEQW
SDR21C1 P. troglodytes  ATKSPEEGAETPVYLALLPPDAEGPHGQFVSEKRVEQW
SDR21C1 M. musculus     ATKSPEEGAETPVYLALLPPDAEGPHGQFVQDKKVEPW
SDR21C1 M. domestica    ATKSPEEGAETPVYLALLPPDATEPHGQFVTEKRVEKW
SDR21C1 G. gallus       APKSPEEGAETPVYLALLPSDADGPHGQFVSEKTVRTW
SDR21C1 C. picta        ATKSADEGAETPVYLALLAPDADGPHGQFVSEKKVQKW
SDR21C1 X. tropicalis   APKSPDEGAETPVYLALLPNNAHSPHGELVSEKKVVPW
SDR21C1 D. rerio        ATKSPDEGAITPVYLALLPAGAKEPHGQFVSEMKVQPW
SDR21C2 H. sapiens      SIRTVEEGAETPVYLALLPPDATEPQGQLVHDKVVQNW
SDR21C2 P. troglodytes  SIRTVEEGAETPVYLALLPPDATEPQGQLVHDKVVQNW
SDR21C2 M. musculus     GSRTVEEGAETPVYLALLPPDATEPHGQLVRDKVVQTW
SDR21C2 M. domestica    ATKSPEEGAETPVYLALLPPDATEPHGQFVMEKRVEKW
SDR21C2 X. tropicalis   ATKSPDEGAETPVYLALLPNNAHSPHGELVSEKKVVPW
                              *** ********   *  * *  *    *  *
```

Figure S19. Alignment of the vertebrate SDR21C family variants. For further details see Fig. S13.

* symbol marks the position of identical amino acid residues of the aligned SDR protein sequences.

```
SDR25C1 H. sapiens      --------------------------------------------MLSAVARGYQ-GWFHP
SDR25C1 P. paniscus     --------------------------------------------MLSAVARGYW-GWFHP
SDR25C1 M. musculus     -----------------------------------------MAAIFRPIPWAFRGSL---
SDR25C1 M. domestica    --------------------------------------------MLRTLAFV-HGVLFSP
SDR25C2 H. sapiens      --------------------------------------------MHKAG---LLG-LCAR
SDR25C2 P. troglodytes  --------------------------------------------MHKTG---LLG-LCAR
SDR25C2 M. musculus     -------------------------------------------MQKAGR---LLG-GWTQ
SDR25C2 M. domestica    MATGLHPLQLPVRISGNMSKGAKKSGKQGTPGFSKHISLLIKnrMLRTL---LKT-TCAQ  1p.2
SDR25C2 G. gallus       --------------------------------------------------------MWGA
SDR25C2 A. carolinensis -------------------------------MFPRAFSSFA-------A---IPQ-ARQA
SDR25C2 X. tropicalys   ------------------------------------------------------------
SDR25C2 D. rerio        --------------------------------------------MLKAI---TRC-LWSN

                                                       ×   × ×
SDR25C1 H. sapiens      CARLSVRMSSTG----IDRKGVLANRVAVVTGSTSgiGFAIARRLARDGAHVVISSRKQQ  1p.1
SDR25C1 P. paniscus     CARLSVRMSSTG----IDGKGVLANRVAVVTGSTSgiGFAIARRLARDGAHMVISSRKQQ  1p.1
SDR25C1 M. musculus     CLPLSARRFSKT----ADENRSLAGKVAVITGSTRgiGFAIARRLAQDGAHVVISSRKQE  1p.1
SDR25C1 M. domestica    RTPPSVRMSSTG----VDKKGVLADKVALITGSTQgiGFAIAQRLARDGAHVVVSSRKQE  1p.1
SDR25C2 H. sapiens      AW-NSVRMASSG----MTRRDPLANKVALVTASTDgiGFAIARRLAQDGAHVVVSSRKQQ  1p.1
SDR25C2 P. troglodytes  AW-NSVRMASSG----MTRRDPLANKVALVTASTDgiGFAIARRLAQDGAHVVVSSRKQQ  1p.1
SDR25C2 M. musculus     AW-MSVRMASSG----LTRRNPLSNKVALVTASTDgiGFAIARRLAEDGAHVVVSSRKQQ  1p.1
SDR25C2 M. domestica    TL-KSIRTNASTTTMSRSNRLLLQDKVALVTASTEgiGFAIAQRLARDGAHVIVSSRKQQ  2p.1
SDR25C2 G. gallus       VGRAGLRAFSGG--GSPNVGRTLEGKVAVVTAATDgiGLAVAQRLGEAGARVLLSSRRQP  1p.1
SDR25C2 A. carolinensis MS-RFVRMN------ATDSKSRLANKVAVVTASTEgiGFAIARRLAQDGAHVVLSSRKKA  1p.1
SDR25C2 X. tropicalys   -------MQSAG---GVQTPKKLQGKVALVTASTEgiGLAIARRLGQDGARVLLSSRKQQ  1p.1
SDR25C2 D. rerio        PV-AGRRM------MSHHISQNLSGKVAIVTASTDgiGLAAAEALGQRGAHVVVSSRRQT  1p.1
                                              *   **  *  * *** * *  *   **    ***

SDR25C1 H. sapiens      NVDRAMAKLQGEGLSVAGIVCHVGKAEDREQLVAkaLEHCGGVDFLVCSAGVNPLVGSTL  2p.0
SDR25C1 P. paniscus     NVDWAMAKLQGEGLSVAGIVCHVGKAEDREQLVAkaLEHCGGVDFLVCSAGVNPLVGSTL  2p.0
SDR25C1 M. musculus     NVDEAVTILKEEGLSVTGTMCHVGKAEDRQHLVTtaLKHSGGIDFLVCVAGVNPLVGSTL  2p.0
SDR25C1 M. domestica    NVDQAVALLKEEGLIAKGMVCHAGKAEDRDKLVTmvADQYGGVDFLICAAGVNPLVGSTL  2p.0
SDR25C2 H. sapiens      NVDQAVATLQGEGLSVTGTVCHVGKAEDRERLVAtaVKLHGGIDILVSNAAVNPFFGSIM  2p.0
SDR25C2 P. troglodytes  NVDQAVATLQGEGLSVTGTVCHVGKAEDRERLVAtaVKLHGGIDILVSNAAVNPFFGSIM  2p.0
SDR25C2 M. musculus     NVDRAVATLQGEGLSVTGIVCHVGKAEDREKLITtaLKRHQGIDILVSNAAVNPFFGNLM  2p.0
SDR25C2 M. domestica    NVDRAVAELQKEGLSVRGTVCHVAKAEDRKRLVNtaLEYYGGIDILVSNAAVNPFFGKLL  3p.0
SDR25C2 G. gallus       NVDAAVQKLRAQGLEVSGVVCHVGQPQDRQHLVQtaLDTYGAIDILVSNAAVNPVMGSTL  2p.0
SDR25C2 A. carolinensis NVDRAVAELQTENLSVSGLVCHVGKAEDRKRLIEtaVERHGGIDILVSNAAVNPYFGSIL  2p.0
SDR25C2 X. tropicalys   NVDRAVQDLRKEGIEVEGTVCHVGNKEDRERLIEtaVQRFGGVDILVSNAAVNPFAGSIL  2p.0
SDR25C2 D. rerio        NVDKAVSLLRSKNIKVIGTTCNVGKAEDREKLINmtVEQCGGVDILVSNAAVNPFFGNIL  2p.0
                        *** *   *        *  *      **  *           * *   * ***  *

                                                                      +   +
SDR25C1 H  sapiens      GTSEQIWDkiLSVNVKSPALLLSQLLPYMEN-rrGAVILVSSIAAYNPVvaLGVYNVSKT  3p.0 4p.1 5p.0
SDR25C1 P. paniscus     GTSEQIWDkiLSVNVKSPALLLSQLLPYMEN-rrDAVILVSSIAAYNPVvaLGVYNVSKT  3p.0 4p.1 5p.0
SDR25C1 M. musculus     GASEQIWDkiLDVNVKSPALLLSKVLPYMENRrgGSVVLVSSGVAYVPVpkLGVYNTSKT  3p.0 4p.1 5p.0
SDR25C1 M. domestica    GASEQVWDkiLDVNVKSPALLLAKLLPYMEKRgsSSVVLVSSVTGYVPVpkLGPYNISKT  3p.0 4p.1 5p.0
SDR25C2 H. sapiens      DVTEEVWDktLDINVKAPALMTKAVVPEMEKRggGSVVIVSSIAAFSPSpgFSPYNVSKT  3p.0 4p.1 5p.0
SDR25C2 P. troglodytes  DVTEEVWDktLDINVKAPALMIKAVVPEMEKRggGSVVIVSSIAAFSPSpgFSPYNVSKT  3p.0 4p.1 5p.0
SDR25C2 M. musculus     DVTEEVWDkvLSINVTATAMMIKAVVPEMEKRggGSVVIVGSVAGFTRFpsLGPYNVSKT  3p.0 4p.1 5p.0
SDR25C2 M. domestica    DATEEVWDkiLDINVKSAALLVNVVVPEMVKRggGSVVFVSSIAAYSPFqyLGPYNVSKT  4p.0 4p.1 5p.0
SDR25C2 G. gallus       EVEESAWEkiFQVNVTAAAMLVKLVVPHMEKRggGAVVLVTSVAGFMPFpaLGPYSVSKT  3p.0 4p.1 5p.0
SDR25C2 A. carolinensis DTPGEVWDkiLDINVKAAAMLVQSVVPHMEKRggGAIVLVSSIAAYSPFpgLGPYNVSKT  3p.0 4p.1 5p.0
SDR25C2 X. tropicalys   ESNEEVWDkiLDVNVKATFLLVKLVVPKMQERggGSIVIVSSVAGFTPFpsLGPYSVSKT  3p.0 4p.1 5p.0
SDR25C2 D. rerio        DSTEEVWDkiLGVNVKASFLLTKMVVPHIEKRggGSVVIVSSVAGYQPMpaLGPYSVSKT  3p.0 4p.1 5p.0
```

```
                              * *    **           *            * *            *  ***

SDR25C1 H. sapiens      ALLGLTRTLALELAPKDIRVNCVVPGIIKTDFSKvfHGNESLWKNFKEHHQLQriGESED  6p.0 7p.1
SDR25C1 P. paniscus     ALLGLTRTLALELAPKDIRVNCVVPGIIKTDFSKvfYGNEPFWKNFKEHHQLQriGESED  6p.0 7p.1
SDR25C1 M. musculus     ALLGLCKSLAVELAPKGIRVNCLVPGIIKTDFSLreKTMPNMLPDMNKIFGVKrlGEPEE  6p.0 7p.1
SDR25C1 M. domestica    ALLGLTKTLAVELAPKGIRVNCLVPGLIKTDFSHilHEDEAFKKDFKNLYGMQrtGQPED  6p.0 7p.1
SDR25C2 H. sapiens      ALLGLTKTLAIELAPRNIRVNCLAPGLIKTSFSRmlWMDKEKEESMKETLRIRrlGEPED  6p.0 7p.1
SDR25C2 P. troglodytes  ALLGLNKTLAIELAPRNVRVNCLAPGLIKTSFSRmlWMDKEKEESMKETLRISrlGEPED  6p.0 7p.1
SDR25C2 M. musculus     ALLGLTKNFAAELAPKNIRVNCLAPGLIKTRFSSvlWEEKAREDFIKEAMQIRrlGKPED  6p.0 7p.1
SDR25C2 M. domestica    ALLGLTKNYASELEPKGIRVNCLAPGLIKTNFSSllWKDESSENTSKNVMKISrlGEPHE  7p.0 8p.1
SDR25C2 G. gallus       ALLGLVKVLAPELRARGVRINAVAPGLIQTRFSAalWQNEATKEQLMSSMGIDrlGTPSD  6p.0 7p.1
SDR25C2 A. carolinensis ALLGLVRNFVPELSSRKIRINCLAPGLIETKFSLalREDEATLEKTMESLRIQriGVPSD  6p.0 7p.1
SDR25C2 X. tropicalys   ALLGLTKALAPELSPLNIRVNCLAPGLIRTKFSSalWKNEAVCEHLMSTLGISriGQPDD  6p.0 7p.1
SDR25C2 D. rerio       ALLGLTRALAPELAQSNIRVNCVAPGIIKTRFSSalWENEGVLEEFLKQTSIKrlGQPEE  6p.0 7p.1
                        *****      **     * *   ** * * **                     * *

SDR25C1 H. sapiens      CAGIVSFLCSPDASYVNGENIAVAGYSTRL-
SDR25C1 P. paniscus     CAGIVSFLCSPDASYINGENIAVAGYSTRL-
SDR25C1 M. musculus     CAGLVSFLCSSDASYITGENIMVAGFSSKL-
SDR25C1 M. domestica    CAGIVSFLCSPDASYITGENIIVAGFSPKL-
SDR25C2 H. sapiens      CAGIVSFLCSEDASYITGETVVVGGGTPSRL
SDR25C2 P. troglodytes  CAGIVSFLCSEDASYITGETVVVGGGTPSRL
SDR25C2 M. musculus     CAGIVSFLCSEDASYINGETVVVGGGTPSRL
SDR25C2 M. domestica    CAGIVSFLCSPDAGYITGETIVVAGGSPSRL
SDR25C2 G. gallus       VAEVVAFLCSPAASYVVGETMVVAGGTPSRL
SDR25C2 A. carolinensis CSGIVSFLCSPDADYITGETIVVAGGAPSRL
SDR25C2 X. tropicalys   CAGAVSFLCSPDASYITGETIVVSGGSHSRL
SDR25C2 D. rerio        IGGVIAFLCSDEASYITGETITVTGGMNSRL
                              ****  * *  **   * *
```

Figure S20. Alignment of the vertebrate SDR25C family variants. For further details see Fig. S13, Table S20.

* symbol marks the position of identical amino acid residues of the aligned SDR protein sequences.

```
                                                                     ×     × ×
SDR26C1 H. sapiens           MAFMKKYLLPILGLFMAYYYYSANEEFRpeMLQGKKVIVTGA--SKGIGREMAYHLAKMG  1p.2
SDR26C1 P. troglodytes       MAFMKKYLLPILGLFMAYYYYSANEEFRpeMLQGKKVIVTGA--SKGIGREMAYHLAKMG  1p.2
SDR26C1 M. musculus          MAVMKNYLLPILVLFLAYYYYSTNEEFRpeMLQGKKVIVTGA--SKGIGREMAYHLSKMG  1p.2
SDR26C1 S. harrisii          MALLKMTFLPALGLLLAYYFYPTTDNFRseMLQGKRVIVTGA--STGIGEQIAYHLARMG  1p.2
SDR26C1 G. gallus            MGLLQKILIPVLGLVLAFCFYSSRENFRpeMLKGKRVIVTGA--STGIGEQMAYHLARMG  1p.2
SDR26C1 A. carolinensis      MGFFQKLLIPSVALVLAVCFYSRKEDFKpeMLKGKYVIVTGA--STGIGEQMAYHLARMG  1p.2
SDR26C1 X. tropicalis        MAGVKLVLLSLFV-GYTVNYFYRSESMNpeLVRGKRVLITGS--STGIGEQIAYEFAQMG  1p.2
SDR26C2 H. sapiens           ----MKVLL-LTGLGALFFAYYWDDNFDpaSLQGARVLLTGA--NAGVGEELAYHYARLG  1p.2
SDR26C2 P. abelii            ----MKVLL-LTGLGALFFAYYWDDNFDpaSLQGARVLLTGA--NAGVGEELAYHYARLG  1p.2
SDR26C2 I. tridecemlineatus  ----MKVLL-LAGLGALFSAYYWDDNFEpaRLQGARVLLTGAAGDAGVGEELAYHYARLG  1p.2
SDR26C2 N. nippon            MKPVGKVLC-AAAIVAGLLAFFWKDPFNpeSLSGARVLLTGA--SAGIGEQMAYHYARFG  1p.2
SDR26C2 A. carolinensis      MRAAGLLLL-VLGIAALWVAFSWRDTFEpgSLSGARVLLTGA--SDGIGEQMAYHYARFG  1p.2
SDR26C2 X. tropicalis        MGIHIKRWC-F-IILVASAAYILRDSFDpeTLANTRVLVTGA--STGIGEEIAYHYARAG  1p.2
SDR26C2 S. grahami           ---MKGYLG-FFLLIAALTAYMWRDTFDpeSVRGARVLITGA--SSGIGEQMAYHYAKFG  1p.2
                                                                 *  **     * *   **     *

SDR26C1 H. sapiens           AHVVVTARSKETLQkvVSHCLELGAASAHYIAGTMEDMTFAEQFVAQAGKLmgGLDMLIL  2p.0 3p.2
SDR26C1 P. troglodytes       AHVVVTARSKETLQkvVSHCLELGAASAHYIAGTMEDMTFAEQFVAQAGKLmgGLDMLIL  2p.0 3p.2
SDR26C1 M. musculus          AHVVLTARSEEGLQkvVSRCLELGAASAHYIAGTMEDMTFAEQFIVKAGKLmgGLDMLIL  2p.0 3p.2
SDR26C1 S. harrisii          AHLVVTARTEAKLKkvIAECLKLGAASAHFISGTMEDLVFAEQVVIKAEKLmgGLDMLIL  2p.0 3p.2
SDR26C1 G. gallus            AHVLVTARTEAKLQqvVERCRALGAGSARLVSGSMEDMATTRQLVEVAEAElgGLDMLIL  2p.0 3p.2
SDR26C1 A. carolinensis      SHILITARTEAKLQkvVSRCLELGAASARYVNGSMEDIIFAQLVVKKAEELwgNLDMLIL  2p.0 3p.2
SDR26C1 X. tropicalis        AHIMLTARRHQRLQevANQCLKLGAASADYVASDMGNLTSAQYVAQETVKKlgGLDYLVL  2p.0 3p.2
SDR26C2 H. sapiens           SHLVLTAHTEALLQkvVGNCRKLGAPKVFYIAADMASPEAPESVVQFALDKlgGLDYLVL  2p.0 3p.2
SDR26C2 P. abelii            SHLVLTAHTEALLQkvVGNCRKLGAPKVFYIAADMASPEAPESVVQFALDKlgGLDYLVL  2p.0 3p.2
SDR26C2 I. tridecemlineatus  SHLVLSAQAEALLQkvVGNCRKLGAPKVFYIAADMASPEAPERVVQFALDKlgGLDYLVL  2p.0 3p.2
SDR26C2 N. nippon            AEIVLTARREAVLQkvVEKCLTLGAKKIFYIPADMSSPSEPEKVVQFAVQKlgGLDYLVL  2p.0 3p.2
SDR26C2 A. carolinensis      AQIVLTARREAVLQkvMAKCLELGAKKAVYFVADMASPTEPEKLVRFASEQlgGLDFVVL  2p.0 3p.2
SDR26C2 X. tropicalis        AKLVLTARREHALQevKSRCLELGAKNVFLVVADMASHNAREQVVAEALSAlgGLDYLVL  2p.0 3p.2
SDR26C2 S. grahami           AQIVITARRVDALKkvVQKCVKLGAQKAMYVTGDMSDPADPERVFKYAVEKlgGLDFLVL  2p.0 3p.2
                                   *     *  *   *  ***         *                 * **   *

SDR26C1 H, sapiens           NHITN-TSLNLFHDDIHHVRKSMEVNFLSYVVLTVAALPMLKQSNGSIVVVSSLagKVAY  4p.2
SDR26C1 P. troglodytes       NHITN-TSLNLFHDDIHHVRKSMEVNFLSYVVLTVAALPMLKQSNGSIVVVSSLagKVAY  4p.2
SDR26C1 M. musculus          NHITQ-TSLSLFHDDIHSVRRVMEVNFLSYVVMSTAALPMLKQSNGSIAVISSLagKMTQ  4p.2
SDR26C1 S. harrisii          NHIEH-SSLDYFNGDIARLRKSMDINFFSYITMTSAALPMLKHTNGSIVVVSSVagKITI  4p.2
SDR26C1 G. gallus            NHVGK-SYFNYFDGDVGHVQKLLNINFLSYVAMTVSALPMLKRSGGSIVVVSSMagKVGF  4p.2
SDR26C1 A. carolinensis      NHVGS-SYFKFFDSDVGHVQKLLNINFLSYVSMTVAALPMLKKSGGSIVVVSSMagKVGF  4p.2
SDR26C1 X. tropicalis        NHIGGSASFGFFKGDMDPVVGSITINFLSYVQLTSTALRALQESQGSIVVMSSMsgRIGA  4p.2
SDR26C2 H. sapiens           NHIGG-APAGTRARSPQATRWLMqvNFVSYVQLTSRALPSLTDSKGSLVVVSSLlgRVPT  4p.0 5p.2
SDR26C2 P. abelii            NHIGG-APAGTRARSPQATRWLMqvNFLSYVQLTSRALPSLTDSKGSLVVVSSLlgRVPT  4p.0 5p.2
SDR26C2 I. tridecemlineatus  NHLGG-IPAGMRARSAQATHWLVqvNFLSYVQLTKLALPSLTDSKGSLVVVSTLlgRVPV  4p.0 5p.2
SDR26C2 N. nippon            NHIGM-TRFQMWAGDVEYTRWLMqvNFFSYVALATAALPTLEKNKGSLVVVSSLtgKIPT  4p.0 5p.2
SDR26C2 A. carolinensis      NHIGA-SPFEMWAGDVDHLRWLMqvNFFSYVALASAALPALAESKGSLVVVSSLagRVST  4p.0 5p.2
SDR26C2 X. tropicalis        NHIGW-TPFKMWDGDVNHTRWLMevNFLSYIHLATAALPYLTQSKGSIIVLSSLtaKTPI  4p.0 5p.2
SDR26C2 S. grahami           NHVGN-TNVELWKGDADHVRSLMqvNFVSYVQMAAAALPVLETSGGSIIVVSSVagKLAS  4p.0 5p.2
                             **                       ** **      **  *    *   * *

                                  +   +
SDR26C1 H, sapiens           PMVAAYSASKFALDGFFSSIRKEYSVSRVNVSITLCVLGLIDteTAMKAVSGIV-HMQAA  5p.2
SDR26C1 P. troglodytes       PMVAAYSASKFALDGFFSSIRKEYSVSKVNVSITLCVLGLIDteTAMKAVSGIV-HMQAA  5p.2
SDR26C1 M. musculus          PMIAPYSASKFALDGFFSTIRTELYITKVNVSITLCVLGLIDteTAMKEISGII-NAQAS  5p.2
SDR26C1 S. harrisii          PLLAGYSATKFALDGFYSSLRVELQMMKVNISITLCILGLIDtdNAVKAISEVMENLIPA  5p.2
SDR26C1 G. gallus            PFTVPYSATKFALDGFFSSLRQEFSIQSVNVSITLCILGFIDteNAMRAAADVL-LMSPA  5p.2
SDR26C1 A. carolinensis      PFTVPYSATKFALDGFFSSLRQEFVMQNTNVSITLCILGLIDteSAMKAISGVF-TSPAA  5p.2
```

```
SDR26C1 X. tropicalis        PFTTSYCASKFALEGFYSSLRREFDLQKNNMSVTVAILGYIDteNAVKKVGNKV-TMTAS  5p.2
SDR26C2 H. sapiens           SFSTPYSAAKFALDGFFGSLRRELDVQDVNVAITMCVLGLRDRASAAEAVrsST-SRPRQ  6p.1
SDR26C2 P. abelii            SFSTPYSAAKFALDGFFGSLRRELDVQDVNVAITMCVLGLRDRASAAEAVrgVT-RVKAA  6p.1
SDR26C2 I. tridecemlineatus  SFSTPYSAAKFALDSFFGSLRRELDVQDVNVAITMCVLGLRDPASTVEGVrgVR-RAKAA  6p.1
SDR26C2 N. nippon            PFTTSYSATKFALDGFFSSLRHELIMQKRNISITLCILGLIDTDSALENTrgKV-HLTAS  6p.1
SDR26C2 A. carolinensis      PFVAPYSATKFALEGFFGSLRHELVMQQKEISITLCFLGLIDTESAISKTqgKV-AMTAA  6p.1
SDR26C2 X. tropicalis        PYTTSYAASKFALEGFFSSLRHELTMQNNPVSITLCILGLIDTQSAMEKIkdKI-TMSAY  6p.1
SDR26C2 S. grahami           PFVAPYTSTKFAMNGFFGALQNELAIRKSNVSVSILILGLIDTESAMNKIrgFT-TMTAY  6p.1
                                  *   ***   *      *              **

SDR26C1 H, sapiens           PKEECALEIIKGGALRQEEVYYDSS----LW-TTLLIRNPCRKILEFLYSTSYNMDRFIN
SDR26C1 P. troglodytes       PKEECALEIIKGGALRQEEVYYDSS----LW-TTLLIRNPCRKILEFLYSTSYNMDRFIN
SDR26C1 M. musculus         PKEECALEIIKGTALRKSEVYYDKS----PL-TPILLGNPGRKIMEFFSLRYYNKDMFVS
SDR26C1 S. harrisii         PKEECALKIIEGGVLRQEEIAYPF------F-PKFLLWNPIRMIMEYFYFLNIKIDKFNR
SDR26C1 G. gallus           PKEECALEIIKGGTLRQREVYYRYA----STKLPLLLRDWAAELLDLLVRQRYRPERLRA
SDR26C1 A. carolinensis     PKEECALEIIKGGALRQREVYYEYT----STKIPLLIREWAPDLLDYLIRRSYNMEKLKE
SDR26C1 X. tropicalis       SKEDCAREVVKAAVLRRPELFYPYW----GIKPIVLLRDWFPGLVAKFLDKFYILENIQ-
SDR26C2 H. sapiens          PEHRGVPLQ------SQTAMFLPPTVPGARTLTETPLRGWPQ-PKMKSSRQKSKTEKNDG
SDR26C2 P. abelii           PGPKAALAVIRGGATRAAGVFYPWR-----FHLLCLLRRWLPRPRAWFIRQELNVTAAAA
SDR26C2 I. tridecemlineatus PGPKAALAVIRGGATRASCVFYPWR-----LHLLCLLRGWLPHPRAWFIRQDLNISAVAA
SDR26C2 N. nippon           PAPEAALAIIWGGATRVQEVFYPWW-----LQYTCCLWVLFPHDRDQVLQSYYNYSSP--
SDR26C2 A. carolinensis     PASEAALSIVKGGATRAQEVFYPWT-----VRILFLVRDFFPAQRDAMIRGAYVYPPPSA
SDR26C2 X. tropicalis       PASDAALAVVSAGAGRQREMYYPWF-----VRPLCFFRDWFPQHRDWFIQRMYHYNS---
SDR26C2 S. grahami         PASEAALSIIKAGATRQKEAYYPWF-----HYFTCLINNIFPFMKDILLSSLSAENMD--


SDR26C1 H. sapiens          K------------------------------
SDR26C1 P. troglodytes      K------------------------------
SDR26C1 M. musculus         N------------------------------
SDR26C1 S. harrisii         KLFMKEN-PQQYKRV--DSRPLLVP------
SDR26C1 G. gallus           A------------------------------
SDR26C1 A. carolinensis     D------------------------------
SDR26C1 X. tropicalis       -------------------------------
SDR26C2 H. sapiens          HLEPVTAWEVQVPRVRRLCRGLARPHLFGHD
SDR26C2 P. abelii           A------------------------------
SDR26C2 I. tridecemlineatus -------------------------------
SDR26C2 N. nippon           -------------------------------
SDR26C2 A. carolinensis     -------------------------------
SDR26C2 X. tropicalis       -------------------------------
SDR26C2 S. grahami          -------------------------------
```

Figure S21. Alignment of the vertebrate SDR26C family variants. For further details see Fig. S13 and Table S21.

* symbol marks the position of identical amino acid residues of the aligned SDR protein sequences.

```
                                                                         ×   × ×
SDR28C1 H. sapiens      ----------------------------------------MARTVVLITGCSSGIGLHLA
SDR28C1 P. abelii       ----------------------------------------MARTVVLITGCSSGIGLHLA
SDR28C1 M. musculus     ----------------------------------------MDPTVVLITGCSSGIGMHLA
SDR28C1 M. domestica    ----------------------------------------MERKVVLITGCSSGIGLHLA
SDR28C1 G. gallus       ---------------------------------MEGTAVPVEKTTVLITGCSSGIGLGLA
SDR28C1 X. tropicalis   ----------------------------------------MEKRVVLITGCSSGIGLGLA
SDR28C1 D. rerio        ----------------------------------------MEQKVVLITGCSSGIGLSLA
SDR28C2 H. sapiens      ----------------MNG--QSQVLPGGGHESREGINMAAAPRTVLISGCSSGIGLELA
SDR28C2 P. paniscus     --------------------------------------MAAAPRTVLISGCSSGIGLELA
SDR28C2 M. musculus     --------------------------------------MASQQRTVLISGCSSGIGLELA
SDR28C2 M. domestica    --------------------------------MARPEAPGGHPRTVLITGCSSGIGLQLA
SDR28C2 G. gallus       ----------------------------------------MAPRTVLITGCSSGIGLALA
SDR28C2 A. carolinensis -------------------------------------MAKATPRTVLITGCSSGIGLRMA
SDR28C2 X. tropicalis   MIKRSNQDSALSLNISMTERFSTPSTPLRSNRPSLPRPPPMEPRLVLITGCSSGIGLALA
SDR28C2 I. punctatus    -------------------------------------MASASQRVVLITGCSSGIGLRIA
                                                                     *** *******   *

SDR28C1 H. sapiens      VRLASDPSQSFkvYATLRDLKTQGRLWEAARALACPPGSLETLQLDVRDSKSVAAARERV  1p.2
SDR28C1 P. abelii       VRLASDPSQSFkvYATLRDLKTQGRLWEAARALACPPGSLETLQLDVRDSNSVAAARERV  1p.2
SDR28C1 M. musculus     VRLASDRSQSFkvYATLRDLKAQGPLLEAARTQGCPPGSLEILELDVRDSKSVAAAQACV  1p.2
SDR28C1 M. domestica    WRLAADPSGSFkvYATLRDLRAQGPLWEGARVRGCPPGSLETLQLDVTDPSSVASARDQV  1p.2
SDR28C1 G. gallus       VRLAADAARRFkvFATMRDLAKGEQLLER--LGGRCPDTLELLQLDVTDPRSLAAAVRCM  1p.2
SDR28C1 X. tropicalis.  VRLASDSYQRFkvYATMRDLSKKENLMEC--VRDCHADTFEILQMDVTDQQSVVSTTEKI  1p.2
SDR28C1 D. rerio        VHLASNPAKAYkvYATMRNLDKKQRLLES--VRGLHKDTLDILQMDVTDQQSILDAQRNV  1p.2
SDR28C2 H. sapiens      VQLAHDPKKRYqvVATMRDLGKKETLEAA--AGEALGQTLTVAQLDVCSDESVAQCLSCI  1p.2
SDR28C2 P. paniscus     VQLAHDPKKRYqvVATMRDLGKKETLEAA--AGEALGQTLTVAQLDVCSDESVAQCLSCI  1p.2
SDR28C2 M. musculus     LQLAHDPRQRYqvVATMRDLGKKEPLEAA--AGEALGKTLSVVQLDVCNDESVTDCLSHI  1p.2
SDR28C2 M. domestica    VKLAQDPQQRYhvIATMRDLGKKEKLETA--AGEALGRTLTVAQMDVCSEDSVSACLNSI  1p.2
SDR28C2 G. gallus       VRLARDKQRRFrvIATMRNVSRSGALVEA--AGSALGRTLEVKQLDVCDEASIRACLDSI  1p.2
SDR28C2 A. carolinensis VQLAQDPGKRFhvIATMRDLRKKDKLEAA--AGDTLNKTLTIQRLDVCNDESVTECINSL  1p.2
SDR28C2 X. tropicalis   VKLANDEQKRFkvVATMRNLAKRGALEEA--AGDALNDTLEIKKLDVCSDSSIRECMGSI  1p.2
SDR28C2 I. punctatus    VLLAKDEQKRYyvIATMRDLKKKDKLVQA--AGDAFGNTLTLQALDVCSDDSVRQCISRV  1p.2
                          **        * ** *       *                   **    *

SDR28C1 H. sapiens      TEGRVDVlvCNAGLGLLGPLEALGEDAVASVLDVNVVGTVRMLQAFLPDMKRRGSGRVLV  2p.2
SDR28C1 P. abelii       TEGRVDVlvCNAGLGLLGPLEALGEDAMASVLDVNVVGTVRVLQAFLPDMKRRGSGRVLV  2p.2
SDR28C1 M. musculus     TEGRVDVlvCNAGRGLFGPLEAHELNAVGAVLDVNVLGTIRMLQAFLPDMKRRHSGRVLV  2p.2
SDR28C1 M. domestica    SEGRVDVlvCNAGRGLLGPLEAHEMEAVGSVLDVNVVGTVRMLQAFLPDMKRRGTGRVLV  2p.2
SDR28C1 G. gallus       RGRHPDVlvCNAGVGLMAPLEMCSEQAMRAVFEVNVFGTARTIQAFLPAMKRHRAGRILV  2p.2
SDR28C1 X. tropicalis.  KEQRVDIlvCNAGVGLMGPLECHSYESMKKIFDVNLFGTISTIQAFLPGMKQRKSGRIII  2p.2
SDR28C1 D. rerio        SEGRIDIlvCNAGVGLMGPLETHSLDTIRAIMDVNLLGTIRTIQTFLPDMKKKRHGRILV  2p.2
SDR28C2 H. sapiens      Q-GEVDVlvNNAGMGLVGPLEGLSLAAMQNVFDTNFFGAVRLVKAVLPGMKRRRQGHIVV  2p.2
SDR28C2 P. paniscus     Q-GEVDVlvNNAGMGLVGPLEGLSLAAMQNVFDTNFFGAVRLVKAVLPGMKRRRQGHIVV  2p.2
SDR28C2 M. musculus     EGGQVDVlvNNAGVGLVGPLEGLSLATMQSVFNTNFFGAVRLVKAVLPGMKRRRQGHIVV  2p.2
SDR28C2 M. domestica    EGRNVDVlvSNAGLGLVGPVESLSMADIRKVFDTNFFGAVQLVKAVLPSMKQRRRGHILV  2p.2
SDR28C2 G. gallus       PGRRIDVlvSNAGVGMIGPLECQSLAAMQGLMDTNFFGLVRLVKEVLPDMKRRRSGHIVV  2p.2
SDR28C2 A. carolinensis PGKQLDVlvNNAGVGLVGPVESISIDDMKRVFETNFFGAIRMIKAVLPEMKKRQKGHIVV  2p.2
SDR28C2 X. tropicalis   PGRHVDVliNNAGVGLIGPIECQSIDDMKGVFETNFFGVVRMIKEVLPDMKRRRRGHIVV  2p.2
SDR28C2 I. punctatus    KDRHIDIliNNAGVGLLGPVESISLDQMKTVFETNFFGTVRMIKEVMPDMKRRRMGHIIV  2p.2
                             * *  *** *   * *             *  *         * **    *

                                       +   +
SDR28C1 H. sapiens      TGSVGGLmgLPFNDVYCASKFALEGLCESLAVLLLPFGVhlSLIECGPVHTAFMEKVLGS  3p.2 4p.1
SDR28C1 P. abelii       TGSVGGLmgLPFNEVYCASKFALEGLCESLAVLLLPFGVhlSLIECGPVHTAFMEKVLGS  3p.2 4p.1
SDR28C1 M. musculus     TASVGGLmgLPFHEVYCASKFALEGLCESLAILLPLFGVhvSLIECGAVHTAFYEKLVGG  3p.2 4p.1
```

```
SDR28C1 M. domestica    TGSVGGLmgLPFNAVYCASKFALEGLCESLAVLLLPFGVhiSLIECGPVRTAFQEKLEGG  3p.2 4p.1
SDR28C1 G. gallus       SSSVGGLqgLPFNAVYCASKFALEGLCESLAIVLRPFNIhmTLVECGPVHTAFMDNAWRA  3p.2 4p.1
SDR28C1 X. tropicalis   SSSVGGLqgIPFNDIYCASKFAVEGLCESLAIVLQHFNIhvSLIECGPVSTNFMNNLHGV  3p.2 4p.1
SDR28C1 D. rerio        TGSMGGLqgLPFNEVYCASKFAIEGACESLAILLQHFNIhiSLIECGPVNTDFLMNLKRT  3p.2 4p.1
SDR28C2 H. sapiens      ISSVMGLqgVIFNDVYAASKFALEGFFESLAIQLLQFNIfiSLVEPGPVVTEFEGKLLAQ  3p.2 4p.1
SDR28C2 P. paniscus     ISSVMGLqgVIFNDVYAASKFALEGFFESLAIQLLQFNIfiSLVEPGPVVTEFEGKLLAQ  3p.2 4p.1
SDR28C2 M. musculus     VSSVMGLqgVMFNDVYAASKFALEGFFESLAIQLRQFNIfiSMVEPGPVTTDFEGKLLAQ  3p.2 4p.1
SDR28C2 M. domestica    ISSVMGLqgVIFNDIYSASKFALEGFCESLAVQLLQFNIfvSLVEPGPVNTDFESKLMDQ  3p.2 4p.1
SDR28C2 G. gallus       ISSVMGLqgIVFNDVYAAAKFAVEGFCESLVVQALRFNVaiSLVEPGPVTTEFEAKVYEE  3p.2 4p.1
SDR28C2 A. carolinensis ISSVMGLqgVPFNDVYAASKFAMEGFCESLAVQLLKFNIfvSMVEPGPVNTEFEMKLMEE  3p.2 4p.1
SDR28C2 X. tropicalis.  ISSVMGLqgIMFNDIYAASKFAVEGFCESLLYQTMNFNIflTLVEPGPVVTEFELKVNEE  3p.2 4p.1
SDR28C2 I. punctatus    MSSVMGMqgVVFNDVYTASKFAIEGFCESLAVQLLKFNVklSLIEPGPVHTEFEAKMMEE  3p.2 4p.1
                          *  *  *  *   * * *** **  ***      *       * * * * *

SDR28C1 H. sapiens      PEE---VLDRTDIHTFHRF-YQYLAHSKQVFREAAQNPEEVAevFLTALRAPKPTLRYFT  5p.0
SDR28C1 P. abelii       PDE---VLDRTDIHTFHRF-YQYLAHSKQVFREAAQNPEEVAevFLTALRAPKPTLRYFT  5p.0
SDR28C1 M. musculus     PGG---ALERADAQTRHLF-AHYLRGYEQALSE-AQDPEEVTelFLTAMRAPQPALRYFS  5p.0
SDR28C1 M. domestica    PGG---VLGSADLDTRALF-SRYQRHCERVFREVAQDPEDVVevFVQALCAPRPVLRYFS  5p.0
SDR28C1 G. gallus       DPDGRELRA-LDAETQQLY-RRYLQHCTQLFLDAAQEVDEVLqvFLEAIAAPRPPLRCIT  5p.0
SDR28C1 X. tropicalis.  DANDSRLQA-GDSDTRSLY-AQYLQHCHSLFQDVAQDTEEILqvFLEAIEAPTPSLRYFT  5p.0
SDR28C1 D. rerio        ETGDKELEVEVDAHTRSLY-DQYLQHCQSVFQNAAQDTEDIIqvYLEAMEAQTPFLRYYT  5p.0
SDR28C2 H. sapiens      VSM--AEFPGTDPETLHYFRDLYLPASRKLFCSVGQNPQDVVqaIVNVISSTRPPLRRQT  5p.0
SDR28C2 P. paniscus     VST--AEFPGTDPETLHYFRDLYLPASRELFRSVGQNPQDVVqaIVNVISSTRPPLRRQT  5p.0
SDR28C2 M. musculus     VSK--AEFPDTDPDTLGYFRDLYLPASRELFRSVGQSPRDVAqvIAKVIGTTRPPLRRQT  5p.0
SDR28C2 M. domestica    VSK--ADFPGTDHDTLNYFRNVYLPASKEIFQTLGQSPEDVAqaIVRVIDSTQPPLRYQT  5p.0
SDR28C2 G. gallus       AER--ADYSQTDPETAAIFTDLYLRNSRDVFASLGQSPEDIAehTLRVIEAARPPFRHQT  5p.0
SDR28C2 A. carolinensis VAR--SEFPGADAATVRYFKEVYLPASHEIFTTMGQTPESVAkaVVNVIAKERPPFRTQT  5p.0
SDR28C2 X. tropicalis.  AIR--GDYSKTDLETAEMFINFYLKNTKAIFSSLGQTPEDVAehILRVITMEEPPFRHQT  5p.0
SDR28C2 I. punctatus    VAK--MEFPGADADTVRYFKDVYMPSSIDIFEAMGQTPDDIAkcTKKVIESSNPRFRNLT  5p.0
                                   *  *       *            *                 *  *

SDR28C1 H. sapiens      TERFLPLLRMRLDDPSGSNYVTAMHREVFGDVPAKAEAGAEAGGGAGPGAEDEAGRGAVG
SDR28C1 P. abelii       TERFLPLLRMRLDDPSGSNYVTAMHREVFGDDPAKAE----AGGGAGPGAGAEAGPSGVG
SDR28C1 M. musculus     TNRFLPLARMRTEDPSGSSYVAAMHQEAFSNLQTQENAK---AGAQVPGVSDTASSALIC
SDR28C1 M. domestica    TEHFLPLAGLRFSDPGGSQYVEAMHHAVFTELEEGEAQ----SGSFHPEASPQ-------
SDR28C1 G. gallus       ARLPAPLARLRPGGPDGADYVRAMHDFVFGPGEAAEEQP---------------------
SDR28C1 X. tropicalis.  TQFFMPLIKLKLSCTGGSEYVRAMHKFVFSGTKQKEHEK---------------------
SDR28C1 D. rerio        NRALLPMSSLKLTSMDGSQYIRAMSKLIFSSPGTDAQK----------------------
SDR28C2 H. sapiens      NIRYSPLTTLKTVDSSGSLYVRTTHRLLFRCPRLLNLGL-----------QCL---SCGC
SDR28C2 P. paniscus     NIRYSPLTTLKTVDSSGSLYVRTTHRLLFRCPRLLNLGL-----------QCL---SCGC
SDR28C2 M. musculus     NTRYLPLTALKAMDPSGSLYVKTAHRLLFRWPHLLNLGL-----------RCL---ACGC
SDR28C2 M. domestica    NARYMPLIALKYADPSGDLSVRTSYRLLFRGQHFFRLSL-----------CCLRFLSGNC
SDR28C2 G. gallus       NAAYTPMAALKHADPSGALMTEAFYKLVFKYGAVLRFGL-----------RAIRLLRWKA
SDR28C2 A. carolinensis NTLYTPLVALKYADTSGDLSVGTYYNLLFRFTGLFHLSM-----------SCLKCITCSC
SDR28C2 X. tropicalis   NQVYTPVTALKYADPSGELVNDIFYKLVFHHDTLMQASL-----------RAIKLIRWKA
SDR28C2 I. punctatus    NSLYTPIVAMKYADETGGLSVHTFYNLLFNFGSLMHITM-----------SILKCLTCSC
                             *          *           *

SDR28C1 H. sapiens      DPELGDPPAAPQ--------------------
SDR28C1 P. abelii       DPELGDPPAARQ--------------------
SDR28C1 M. musculus     LPECAIPRVASELGWSASDKPGQDNSCYQQKI
SDR28C1 M. domestica    --------------------------------
SDR28C1 G. gallus       --------------------------------
SDR28C1 X. tropicalis   --------------------------------
SDR28C1 D. rerio        --------------------------------
SDR28C2 H. sapiens      LPTRVRPR------------------------
```

```
SDR28C2 P. paniscus     LPMRVRPR------------------------
SDR28C2 M. musculus     LPTRVWPR---QTEQN----------------
SDR28C2 M. domestica    LRPKVSPM------------------------
SDR28C2 G. gallus       QKVKAGAR---LLGFK----------------
SDR28C2 A. carolinensis FRRRVTPA------------------------
SDR28C2 I. punctatus    LRRRTISP---D--------------------
SDR28C2 X. tropicalis   HKVQQGAR---MLGLI----------------
```

Figure S22. Alignment of the vertebrate SDR28C family variants. For further details see Fig. S13.

* symbol marks the position of identical amino acid residues of the aligned SDR protein sequences.

```
SDR32C1 H. sapiens      ----------------------------------------MVSPATrkSLPKVKAMDFIT  1p.1
SDR32C1 P. troglodytes  ----------------------------------------MVSPATrkSLPKVKAMDFIT  1p.1
SDR32C1 M. musculus     ----------------------------------------MISPSFrkGMLKERVMDLAS  1p.1
SDR32C1 M. domestica    MALRHFLLGREKLIYHGLSGNqdEDLLTLKIFKGVCQKILKPNASVerIVLKGKLMDYIT  1p.0 2p.1
SDR32C1 G. gallus       -------------------------------------------------------MDL-T
SDR32C1 A. carolinensis -------------------------------------------------------MGL-P
SDR32C1 X. tropicalis   -----------------------------------------MFIPFRKRVYGVKPMDL-T
SDR32C1 D. rerio        ------------------------------------------------MGQSLRHMERAL
SDR32C2 H. sapiens      -------------------------------------------------------MGV-M
SDR32C2 P. troglodytes  -------------------------------------------------------MGV-M
SDR32C2 M. musculus     ------------------------------------------------------------
SDR32C2 M. domestica    ----------------------------MSLFKTL-----IVLSSYPT-LTLKSKMGI-M
SDR32C2 G. gallus       -------------------------------------------------------MGI-L
SDR32C2 A. carolinensis -------------------------------------------------------MGV-L
SDR32C2 X. tropicalis   -------------------------------------------------------MGF-L
SDR32C2 D. rerio        -------------------------------------------------------MAV-P

                                                               ×   × ×
SDR32C1 H. sapiens      STAILP-LLFGCLGVFGLFRLLQWVRGKAYLRNAVVVITGATSGLGkeCAKVFYAAGAKL  2p.2
SDR32C1 P. troglodytes  STAILP-LLFGCLGVFGLFRLLQWVRGKAYLRNAVVVITGATSGLGkeCAKVFYAAGAKL  2p.2
SDR32C1 M. musculus     QTTILP-LLFGCLGIFSLFRLLQRIRSKAYLRNAVVVVTGATSGLGreCAKVFHAAGAKL  2p.2
SDR32C1 M. domestica    SIAIIP-LLVGCMGIFGLFRFLQWIRMKAYIQDSVVVITGATSGLGkeCAKVFYTAGAKL  3p.2
SDR32C1 G. gallus       STVIIP-LLFGSLGLFSLFRLLQWMRMRAYLRGAVVVVTGATSGLGkeCAKAFHAAGSRL  1p.2
SDR32C1 A. carolinensis SAAVLP-LLFGSLGLYGVFWLLRRMRAKAYLKDAVVVITGATSGLGkeCAKTFHAAGSKL  1p.2
SDR32C1 X. tropicalis   TWAIFP-LLLGSIGVYSLYKLLQRLRSGAYLQDAVVVITGATSGLGreCAKVFYAAGTRL  1p.2
SDR32C1 D. rerio        GVGIGP-LAAGTVGLLILLKVIQRLRRRPNIQDKVVVITGASSGLGkeCARVFHAAGARL  1p.2
SDR32C2 H. sapiens      AMLMLPLLLLGISGLLFIYQEVSRLWSKSAVQNKVVVITDAISGLGkeCARVFHTGGARL  1p.2
SDR32C2 P. troglodytes  AMLMLPLLLLGISGLLFIYQEVSRLWSKSAVQNKVVVITDAISGLGkeCARVFHTGGARL  1p.2
SDR32C2 M. musculus     ---MLPLLLLGISGLLFIYQEASRLWSKSAVQNKVVVITDAISGLGkeCARVFHAGGARL  1p.2
SDR32C2 M. domestica    AVLMLPLLLLAISGILFIYQEVSRLWSKSAVQNKVVVITDAISGLGkeCSRLFHAGGARL  1p.2
SDR32C2 A. carolinensis GVLILPLLVVVISGVVYIYRTVIQMMSKSAVRSKVVVITDALSGVGkeCSHVFHAGGARL  1p.2
SDR32C2 G. gallus       AVFALPLLLLGISGIIYIYQTVIWLVSKSAVQNKVVVITDAISGLGkeCSRVFHSGGARL  1p.2
SDR32C2 X. tropicalis   TFLIVPLLILGISGIVYIYREVVRLMSRSALKNKVVVITDAISGLGkeCSRVFHSAGARL  1p.2
SDR32C2 D. rerio        SVMVLPLLIVVFAGVYYVYNEVMRFMSKSVVRNKVVVITDAVSGMGseCARLFHAGGARL  1p.2
                             * *     *                    *** * * ** * **   *   *  *

SDR32C1 H. sapiens      VLCGRNGGALEELIRELTASHATkvQTHKPYLVTFDLTDSGAIVAAAAEILQCFGYVDIL  3p.0
SDR32C1 P. troglodytes  VLCGRNGGALEELIRELTASHATkvQTHKPYLVTFDLTDSGAIVAAAAEILQCFGYVDIL  3p.0
SDR32C1 M. musculus     VLCGRNVKALEELSRELAGSS--qgQTHQPFVVTFDLADPGTIAAAAAEILQCFGYVDVL  3p.0
SDR32C1 M. domestica    ILCGRNNERLEELIRELIATNIKkt--HRPHAVVFDLAESKTIVPAANEILKYSGHVDIL  4p.0
SDR32C1 G. gallus       VLCGRDSEKLKDLAQELSAMTDHRKniHKPHTVVFDLSDTKTILNAAEEILKHLGHVDIL  2p.0
SDR32C1 A. carolinensis VLCGRNGERLRDLLRELSATADPTKnpHQHHTVVFDLSDIKAVVSAAEEILKCVDHVDIL  2p.0
SDR32C1 X. tropicalis   VLCGRSEEGLKNLVQELSQMRIKSAqlHKPHMVIFDLSDVEAVNSAANEILHLTGRVDIL  2p.0
SDR32C1 D. rerio        ILCGRDQRRLQEVVEELRNKTYGKTqtYTPCTVTFDLSNTSVVCSAAAEILKCHGHIDVL  2p.0
SDR32C2 H. sapiens      VLCGKNWERLENLYDALISVADPSkqTFTPKLVLLDLSDISCVPDVAKEVLDCYGCVDIL  2p.0
SDR32C2 P. troglodytes  VLCGKNWERLENLYDALISVADPSkqTFTPKLVLLDLSDISCVPDVAKEVLDCYGCVDIL  2p.0
SDR32C2 M. musculus     VLCGKNWEGLESLYATLTSVADPSkt-FTPKLVLLDLSDISCVQDVAKEVLDCYGCVDIL  2p.0
SDR32C2 M. domestica    VLCGKNWEKLEILYDALISVADPSkt-FTPKLVLLDLSDISCVQDVAKEILDCYGCVDIL  2p.0
SDR32C2 G. gallus       VLCGRTWEKLEALYDALISVADPStt-YTPKLVLLDISDTDYIQDVAKEILNCYGSVDIL  2p.0
SDR32C2 A. carolinensis VLCGKHLDNLEALYDSLTSAADPTat-FTPKLVLLDLSDVNCIQDVAKEILDCYGCVDIL  2p.0
SDR32C2 X. tropicalis   VLCGKTWEKLEALHDALISVADPSvt-FTPKLVLLDISDINNMEAMGKEIQDCYGCVDVL  2p.0
SDR32C2 D. rerio        VLCGPSWDKLESLYDSLCSGSDPSqt-FTPKLVLLDFSDMENISDVVSEICECYGCVDVL  2p.0
                         ***     *      *               *  *            *        * *

SDR32C1 H. sapiens      VNNAGISYRGTIMDTTVDVDKRVMETNYFGPVALTkaLLPSMIKRRQGHIVAISSIQGKM  4p.2
SDR32C1 P. troglodytes  VNNAGISYRGTIMDTTVDVDKRVMETNYFGPVALTkaLLPSMIKRRQGHIVAISSIQGKI  4p.2
```

```
SDR32C1 M. musculus     INNAGISYRGTISDTIVDVDRKVMEINYFGPVALTkaLLPSMVERKQGHIVAISSIQGKI  4p.2
SDR32C1 M. domestica    INNAGVSYRGTIMDTALEVDKKVMETNYFGPVALTkaILPSMIEKKQGHIVVISSIQGKI  5p.2
SDR32C1 G. gallus       INNAGISYRGTIVDTGLDVDKKVMETNYFGPIALTkaLLPSMIKRRQGHIVAISSVQGKI  3p.2
SDR32C1 A. carolinensis INNAGISYRGSIAETVIEVDRKVMETNYFGPIALTkaLLPSMIRRRKGHVVAISSVQGKF  3p.2
SDR32C1 X. tropicalis   INNAGISYRGTILDTKVSVDRMVMDTNYFGPVALTkaLIPSMIKNRRGHIVVISSVQGKI  3p.2
SDR32C1 D. rerio        INNAGVSYRGNILDTHVSVQREVMETNYFGPVALTqaILPSMVDRGSGHIVVISSVQGKI  3p.2
SDR32C2 H. sapiens      INNASVKVKGPAHKISLELDKKIMDANYFGPITLTkaLLPNMISRRTGQIVLVNNIQGKF  3p.2
SDR32C2 P. troglodytes  INNASVKVKGPAHKISLELDKKIMDANYFGPITLTkaLLPNMISRRTGQIVLVNNIQGKF  3p.2
SDR32C2 M. musculus     INNASVKVKGPAHKISLELDKKIMDANYFGPITLTkvLLPNMISRRTGQIVLVNNIQAKF  3p.2
SDR32C2 M. domestica    INNASMKVKGPAQNISLELDKKIMDVNYFGPITLTkaLLPNMISRRTGQIVLVNNIQGRF  3p.2
SDR32C2 G. gallus       INNASMKVKGAVQSISLELDKKIMDANYFGPITLTkaILPNMISRRTGQIVLINSIQGKI  3p.2
SDR32C2 A. carolinensis INNASTKLKGTVQNISLELDKKIMDANYFGPITLTkaLIPNMISRRTGQVILVNNIQGKV  3p.2
SDR32C2 X. tropicalis   INNASMKMKGPLQSVSLELDKKIMDANYFGPITLVkaILPHMISRRTGQIVLVNTIQGKI  3p.2
SDR32C2 D. rerio        ICNSSMKVKAPVQNLSLEMDKTIMDVNYFGPITLAkgVLPLMITRRTGQFVLVNSIQGKL  3p.2
                          *                    *  *****  *     * *     *        *

                               +   +
SDR32C1 H. sapiens      SIPFRSayAASKHATQAFFDCLRAEMEQYEIEVTVISPGYIHTNLSVNAIT---------  5p.2
SDR32C1 P. troglodytes  SIPFRSayAASKHATQAFFDCLRAEMEQYEIEVTVISPGYIHTNLSVNAIT---------  5p.2
SDR32C1 M. musculus     SIPFRSaySASKHATQAFFDCLRAEMEEANIKVTVISPGYIHTNLSVNAVT---------  5p.2
SDR32C1 M. domestica    SVPFRSayAASKHATQAFFDCLRAEVEQHDIEVTVISPGYIQTNLSLNALT---------  6p.2
SDR32C1 G. gallus       SIPFRSayAASKHATQAFFDCLRAEVEQYDIDVTVISPGYIQTNLSLNAVT---------  4p.2
SDR32C1 A. carolinensis GLPFRSayAASKHAFQAFFDCLRAEVEQYGVVVTVVSPGYIQTNLSLNAIT---------  4p.2
SDR32C1 X. tropicalis   SIPFRSaySASKHATQAFFDCLRAEMSPYEIDVTVVNPGYIKTNLSLNAVT---------  4p.2
SDR32C1 D. rerio        SIPYRSayAASKHAMQAYYDCLRAEVDSLGLHVSVLSPGYVRTNMSINAVT---------  4p.2
SDR32C2 H. sapiens      GIPFRTtyAASKHAALGFFDCLRAEVEEYDVVISTVSPTFIRSYHVY------------P  4p.2
SDR32C2 P. troglodytes  GIPFRTtyAASKHAALGFFDCLRAEVEEYDVVISTVSPTFIRSYHVY------------P  4p.2
SDR32C2 M. musculus     GIPFRTayAASKHAVMGFFDCLRAEVEEYDVVVSTVSPTFIRSYRAS------------P  4p.2
SDR32C2 M. domestica    GIPLRTayAASKHAAQGFFDCLRAEVEEFEVVISTVSPTFIRSYHMY------------T  4p.2
SDR32C2 G. gallus       GVPFRAayAASKHAAVGFFDCLRAEMEEFDISVSTVNPTFICSYHHQ------------P  4p.2
SDR32C2 A. carolinensis GVPFRAayAASKHAALGFFDCLRAELQEFGVCVSTVTPSFIRSYSVQ------------P  4p.2
SDR32C2 X. tropicalis   GVPFRAayAASKHAIQGFFDCLRAEVEEFDVSVSTVSPTFIRSYHVQ------------P  4p.2
SDR32C2 D. rerio        ALPFRTcyAASKHAVQAFFDCLRAEVEEFGISVSTISHTFINAGAENATPTEATPITATP  4p.2
                          * *  * *****     ******

SDR32C1 H. sapiens      ----ADGSRYgv-MDTTTAQGRSPVEVAQDVLAAVGKKKKDVILADLLPSLAVYLRTLAP  6p.2
SDR32C1 P. troglodytes  ----ADGSRYgv-MDTTTAQGRSPVEVAQDVLAAVGKKKKDVILADLLPFLAVYLRTLAP  6p.2
SDR32C1 M. musculus     ----ADGSRYga-LDKNTAQGRSAAEVAQDVFDAVGKKKKDVLLTDFVPSMAVYIRTLAP  6p.2
SDR32C1 M. domestica    ----ADGSTYgv-MDKNTANGRSPTEVAYAVLTAVGKKKKEVMVADLLPCLAVSLRTLFP  7p.2
SDR32C1 G. gallus       ----ADGSRYgv-MDKNTAEGKTAAEVAQVVLCAVGQKKKEVLIAGLKPSLAVYLRNLFP  5p.2
SDR32C1 A. carolinensis ----ADGSQYgv-MDKATARGKAAAEVAQVVLDAVGEKRKEVVVAGFLPSLVVYLRTLCP  5p.2
SDR32C1 X. tropicalis   ----GDGSNYgv-MDNNTAEGRTPEEVAQTVLRAVGERRKELLVAGLVPTLAVYLRTLAP  5p.2
SDR32C1 D. rerio        ----GDGSKYgv-MDRTTATGADPVDVAKDILKAVCQKKKDVVMAGLGPTTAIYLRTLWP  5p.2
SDR32C2 H. sapiens      EQGNWEASIWkfFFRKLT-YGVHPVEVAEEVMRTVRRKKQEVFMANPIPKAAVYVRTFFP  5p.2
SDR32C2 P. troglodytes  EQGNWEASIWkfFFRKLT-YGVHPVEVAEEVMRTVRRKKQEVFMANPIPKAAVYVRTFFP  5p.2
SDR32C2 M. musculus     EQRNWETSICkfFCRKLA-YGVHPVEVAEEVMRTVRRKKQEVFMANPVPKAAVFIRTFFP  5p.2
SDR32C2 M. domestica    EPGNWEASIWkfFFRKFA-YGVHPVEVAEEVMKTIRRKKQEVFIANPIPKAAIYIRTFFP  5p.2
SDR32C2 G. gallus       APGNWEASIWkfFFRKVT-YGVHPAEVAEEVLRTVSSKKQEVLMANPIPRAAVYIRTFFP  5p.2
SDR32C2 A. carolinensis QPSNWESSVWkfLCRKLA-YGVHPIEVADEILRTVNRKKQEVFMANPIAKAAVYIRTFFP  5p.2
SDR32C2 X. tropicalis   QPGNWEASIWkfFFRKLS-YGAHPVEVAEEVLSTVSRKKQEVFMANPIPRAAVYIRTFLP  5p.2
SDR32C2 D. rerio        TKATPTNPIWayVCSKLNTHGVGPQILAREIVRSVNRQSREVFLAHPVPTVALYIRALMP  5p.2
                                            *      *                           *   *

SDR32C1 H. sapiens      GLFFSLMASRARKERKSKNS-
SDR32C1 P. troglodytes  GLFFSLMASRARKERKSKNS-
SDR32C1 M. musculus     GLFFRIMASRARKERKSKSS-
```

```
SDR32C1 M. domestica    RLFFYIMAMRARKERKGKTS-
SDR32C1 G. gallus       RLFFNLMAARAKKERKTKDS-
SDR32C1 A. carolinensis RLFFAIMASRAQKERKAKAS-
SDR32C1 X. tropicalis   TIFFSFMAARAKKERKLKDS-
SDR32C1 D. rerio        ALYFRVMASRARKQTGKEE--
SDR32C2 H. sapiens      EFFFAVVACGVKEKLNVPEEG
SDR32C2 P. troglodytes  EFFFAVVACGVKEKLNVPEEG
SDR32C2 M. musculus     EFFFAVVACGVKEKLNVPEEG
SDR32C2 M. domestica    EFFFAVVACGVKEKLNVPEDR
SDR32C2 G. gallus       EMFFAIVASGIREKLKTEQEN
SDR32C2 A. carolinensis ELFFAVVAAGVKEKQKIEDEK
SDR32C2 X. tropicalis   ELFFAVVATGVKEKHFVEEEK
SDR32C2 D. rerio        GCFFSVVSAGVRDGAMAEQLK
                           *
```

Figure S23. Alignment of the vertebrate SDR32C family variants. For further details see Fig. S13, Table S23.

* symbol marks the position of identical amino acid residues of the aligned SDR protein sequences.

```
SDR42E1 H. sapiens     ------------------------------------------------------------
SDR42E1 P. troglodytes ------------------------------------------------------------
SDR42E1 M. musculus    ------------------------------------------------------------
SDR42E1 S. harrisii    ------------------------------------------------------------
SDR42E1 G. gallus      ------------------------------------------------------------
SDR42E1 P. bivittatus  ------------------------------------------------------------
SDR42E1 X. tropicalis  ------------------------------------------------------------
SDR42E1 L. oculatus    ------------------------------------------------------------
SDR42E2 H. sapiens     MKSNPPRSSLEACKAAGQGEKSCPVCQACGEVSGPRSGSGSESRPAPKPGAIPGPGLGPK
SDR42E2 P. troglodytes MKSNPPRSSLEACKAAGQGEKSCPVCQACGEVSGPRSGSGSESRPAPKPGAIPGPGLGPK
SDR42E2 M. musculus    MKTNPAGSPLETCQTAGQGEKGCPVCQACRGTSGLGSASESGSGLRPGPGVVLDSGLGPG
SDR42E2 S. harrisii    ------------------------------------------------------------
SDR42E2 G. gallus      ------------------------------------------------------------
SDR42E2 P. bivittatus  ------------------------------------------------------------
SDR42E2 X. tropicalis  ------------------------------------------------------------
SDR42E2 L. oculatus    ------------------------------------------------------------


SDR42E1 H. sapiens     ------------------------------------------------------------
SDR42E1 P. troglodytes ------------------------------------------------------------
SDR42E1 M. musculus    ------------------------------------------------------------
SDR42E1 S. harrisii    ------------------------------------------------------------
SDR42E1 G. gallus      ------------------------------------------------------------
SDR42E1 P. bivittatus  ------------------------------------------------------------
SDR42E1 X. tropicalis  ------------------------------------------------------------
SDR42E1 L. oculatus    ------------------------------------------------------------
SDR42E2 H. sapiens     AIPGPQAGSGTVPRPGAISGTGPGLGPGPGAGSVPGPGAGSVPGLGARSVPGPGAGSVPG
SDR42E2 P. troglodytes AIPGPQAGSDTVPRPGAISGTGPSLGPGPGAGSVPGPGTGSVP--------GPGAGSVPG
SDR42E2 M. musculus    SGTSAVPGSGPQPSAVSVTGLRPGTTSGPNATLGPGLGAGSLPGPGARSGLRPGSESVPQ
SDR42E2 S. harrisii    -MAQPKSDSLTVPQAQAPQGKVPQSQPP-QT--------QALQGQAPQSKPSQSQVLQAQ
SDR42E2 G. gallus      ------------------------------------------------------------
SDR42E2 P. bivittatus  ------------------------------------------------------------
SDR42E2 X. tropicalis  ------------------------------------------------------------
SDR42E2 L. oculatus    ------------------------------------------------------------


SDR42E1 H. sapiens     ------------------------------------------------------------
SDR42E1 P. troglodytes ------------------------------------------------------------
SDR42E1 M. musculus    ------------------------------------------------------------
SDR42E1 S. harrisii    ------------------------------------------------------------
SDR42E1 G. gallus      ------------------------------------------------------------
SDR42E1 P. bivittatus  ------------------------------------------------------------
SDR42E1 X. tropicalis  ------------------------------------------------------------
SDR42E1 L. oculatus    ------------------------------------------------------------
SDR42E2 H. sapiens     PGAGSVPGPGAGSVPGPGAGSGPG--LGGGLGPGVGAGPGAGSVPGPGAGSVPGPGAGS-
SDR42E2 P. troglodytes LGAGSVPGPGAGSVPGPGAGSGPG--LGAGLGPGLGAGPGAGSVPGPGAGSVPG------
SDR42E2 M. musculus    PGAGSVPQPGAGPVPQPG----------------------VGPVPHPGAGSVSQPGAGS-
SDR42E2 S. harrisii    ALQGKVPQSQAPQAQTP-QGQAPQAQIPQGQAP-----QGQAPQAKISQGQAPQGQAPQ-
SDR42E2 G. gallus      -------------------------MKLAG-------------------------IVCIG
SDR42E2 P. bivittatus  ------------------------------------------------------------
SDR42E2 X. tropicalis  -------------------------MR---------EKEGAYE-----------------
SDR42E2 L. oculatus    -------------------------MKPLGEA-----ARAAGAGRDLSHGSSGAPRCCRD


SDR42E1 H. sapiens     ------------------------------------------------------------
SDR42E1 P. troglodytes ------------------------------------------------------------
```

```
SDR42E1 M. musculus    ------------------------------------------------------------
SDR42E1 S. harrisii    ------------------------------------------------------------
SDR42E1 G. gallus      ------------------------------------------------------------
SDR42E1 P. bivittatus  ------------------------------------------------------------
SDR42E1 X. tropicalis  ------------------------------------------------------------
SDR42E1 L. oculatus    ------------------------------------------------------------
SDR42E2 H. sapiens     -VPGPGAGSVPGAGAGSTPEPELGPGLRQGSG--TGPRPS---ESTTTPTPAPQQK----
SDR42E2 P. troglodytes ------------PGAGSTPEPELGPGLRQGSG--TGPKPS---ESTTTPTPAPQQK----
SDR42E2 M. musculus    -VSQPVSESVTSPGSAPLPGLGLGPGTRRGSG--TGSDPI---ESVRTPVPVQQQK----
SDR42E2 S. harrisii    -SQAPQDQ-------------VLQSQVPQSQAPQAQALQD---KPPQSQVPNSKPK----
SDR42E2 G. gallus      EHYGAI-FNMEKKLLC-------ASGH-RGHYFSDRKLPVSEAANPWHQPPSSGVR----
SDR42E2 P. bivittatus  ------------------------------------------MEKPNDPKPSRFY--KCT
SDR42E2 X. tropicalis  --LEGLRSRVEMGDLCSTCL--------RNSRPFRGS----IKHRANGVVPHQSQASACT
SDR42E2 L. oculatus    rcWCAARQRM---ALCRTKGPPARPGQPAGTK------RR---S--NGVVP-HRSR---- 1p.1

                                                   ×  ×  ×
SDR42E1 H. sapiens     --------------MDPKRSQKESVLITGGSGYFGFrlGCALNQNGVHVILFDISSPAQT  1p.1
SDR42E1 P. troglodytes --------------MDPKRSQKETVLITGGSGYFGFrlGCALNQKGVHVILFDISSPAQT  1p.1
SDR42E1 M. musculus    --------------MDSPRFPEETVLITGGGGYFGFrlGCALNQKGARVILFDITQPAQN  1p.1
SDR42E1 S. harrisii    --------------MDLQKSPQETVLITGGGGYFGFrlGCTLCKKGITVILFDINISIQD  1p.1
SDR42E1 G. gallus      --------------MEAGSSAKETVLITGGGGYFGFrlGCTIYKKGVDVILFDVTKPLQP  1p.1
SDR42E1 P. bivittatus  --------------MERENPNKEAVLITGGSGYFGFrlGSALAKNKVDVILFDVSRPTQE  1p.1
SDR42E1 X. tropicalis  --------------MSSSQHARQTVVITGGGGYFGHrlGCTLHQKGVNVILFDIRKPDLE  1p.1
SDR42E1 L. oculatus    ------------MEQLNQGKTKEMVLITGGGGYFGFrlACALHEAGALVTLFDVQPPSEA  1p.1
SDR42E2 H. sapiens     ------------TQAKPTKAARQKVLVTGGGGYLGFSLGSHLAKSGTSVILLDRRRPQWE
SDR42E2 P. troglodytes ------------TQAKPTKAARQKVLVTGGGGYLGFSLGSHLAKSGTSVILLDCRRPQWE
SDR42E2 M. musculus    ------------TQAKHTQAPRQKVLVTGGGGYLGFSLGSSLAKRGTSVILLDLRRPQWP
SDR42E2 S. harrisii    ------------EQAKPPYPSSQKALVTGGGGYFGFSLGSSLVRKGVSVVLLDIQHPKWE
SDR42E2 G. gallus      ------------GNAGALKDKAMRAVVTGGGGYFGYKLGCALASSGASVVLYDIHKPIWE
SDR42E2 P. bivittatus  KEDAKLVEKLPESCQSPLKKVSDKTVITGGAGYFGFTLGCALAKSGTKVILYDVNLPIWD
SDR42E2 X. tropicalis  P---------SAHLGTLLHAGITKVLVTGGGGYVGHNLACALAQSGISVLLFDINRSQWE
SDR42E2 L. oculatus    ------------GPGWQAAPAGGRALVTGGAGYFGRSLGRALASSGVSVVLLDLHEPRWE
                                                  *** ** *  *          * * *

SDR42E1 H. sapiens     IPEGIKFIQGDIRHLSDVEKAFQDAD-VTCVFHIASYGMSGREQLNRNLIKEVNVRGTDN
SDR42E1 P. troglodytes IPEGIKFIQGDIRHLSDVEKAFQDAD-VTCVFHIASYGMSGREQLNRNLIEEVNVRGTDN
SDR42E1 M. musculus    LPEGIKFVCGDIRCLADVETAFQDAEKVACVFHVASYGMSGREQLNKTQIEEVNVGGTEN
SDR42E1 S. harrisii    IPRGMKLICGDIRCIADLENALQN---VTCVFHIASFGMSGKEQLNHKRIEDVNVKGTEN
SDR42E1 G. gallus      VPEGIKFMQGNVCCLAEVEEALKD---VICVFHIASYGMSGREQLNRKLIEDVNVKGTEN
SDR42E1 P. bivittatus  IPKGVKFVKGDVCDIQEVEAALQG---MSCVFHIASFGMSGREQLNQKRIEEVNIKGTEN
SDR42E1 X. tropicalis  VPEGIQFVQGDVRSLSQLEDVMTG---ASCVFHTASYGMSGREQLQWQKIEAVNVKGTEN
SDR42E1 L. oculatus    IPDGMAFLRGDVREYLEVEGATRG---VGCVFHTASYGMSGREQLDRALIEEVNMRGTEH
SDR42E2 H. sapiens     LSPETKFIqaDVRDEEALYRAFEG---VDCVFHVASYGMSGAEklQKEQIESINVGGTKL  1p.0 2p.0
SDR42E2 P. troglodytes LSPETKFIqaDVRDEEALYRAFEG---VDCVFHVASYGISGAEklQKEQIESINVGGTKL  1p.0 2p.0
SDR42E2 M. musculus    LPSGTEFVqaDVRDEEALYQAFQG---VDCVFHVASYGMSGAEklQKQEIESINVGGTKL  1p.0 2p.0
SDR42E2 S. harrisii    LPKGVAFIqaDIRDGEALYQACEG---VDCVFHVASYGMSGAEklHKEQIESVNIGGTKI  1p.0 2p.0
SDR42E2 G. gallus      IPNGVVCIqaDVRDYDAVFAACEG---ADCVFHVASYGMSGREqlHREEIETVNINGTRF  1p.0 2p.0
SDR42E2 P. bivittatus  IPKGIILIksDVRNFSDLYSACEG---VDCLFHAAAYGMTGIEqlHKKHIRSVNIGGTGI  1p.0 2p.0
SDR42E2 X. tropicalis  IPSGAVFIqgDIRDYNCLYTACEG---VDCIFHVASYGMSGYQqlEKEKIDSINIGGTKL  1p.0 2p.0
SDR42E2 L. oculatus    IPEGAVFQqsDIRDYDALYKACEG---VDCVFHTASYGMSGPEqlKKEQIESVNVGGTNN  2p.0 3p.0
                                                    * ** *  *  *   *    *   *  **

                                                                      +   +
SDR42E1 H. sapiens     ILQVCQRRRVPRLVYTSTFNVIFGGQVIRNGDE-SLPYLPLHLPDHYSRTKSIAEQKVL
SDR42E1 P. troglodytes ILQVCQRRSVPRLVYTSTFNVIFGGQVIRNGDE-SLPYLPLHLPDHYSRTKSIAEQKVL
SDR42E1 M. musculus    ILRACLERGVPRLVYTSTFNVIFGGQVIRNGDE-SLPYLPLHLPDHYSRTKSIAEKKVL
```

```
SDR42E1 S. harrisii    VLEACRRKGVSRLVYTSTYNVVFGGQVIMNGDE-SLPYLPLHLHPDHYSRTKSVADKKVL
SDR42E1 G. gallus      VIQACKSTGVSSLVYTSTYNVIFGGQIIENGDE-SLPYLPLHLHPDHYSRTKSLAEMKVL
SDR42E1 P. bivittatus  VIETCRKVGISKLVYTSTYNVVFGGQVIENGDE-SWPYLPLHLHPDHYSRTKALAEMKVL
SDR42E1 X. tropicalis  IIQACINKNVKRLVYTSTFNVVFGGQTIRNGDE-SLPYLPQDAFVDNYSRTKTIAEAFVL
SDR42E1 L. oculatus    VIRACVGNAVPRLVYTSTFNVVFGGQVIENGDE-SLPYLPLHLHPDHYSRTKSLAEMAVL
SDR42E2 H. sapiens     VIdvCVRRRVPRLIYTSTVNVAFGGKPIEQGDEDSVPYFPLDehVDHYSRTKAIADQLTL  3p.2 4p.0
SDR42E2 P. troglodytes VIdvCVRRRVPRLIYTSTVNVAFGGKPIEQGDEDSVPYFPLDehVDHYSRTKAIADQLTL  3p.2 4p.0
SDR42E2 M. musculus    VInvCVRRRVPRLVYTSTVNVTFGGKPIEQGNEESIPYFPLDkhMDHYSRTKAIADQLTL  3p.2 4p.0
SDR42E2 S. harrisii    VIdvCIKRQIPRLVYTSTVNVVFGGKPIVQGDEDSVPYFPLEkhIDHYSRTKAVADQMIL  3p.2 4p.0
SDR42E2 G. gallus      IIdaCKQRNITRLIYTSTVNVVFGGLPIEDGDEETVPYFPIEkhVDHYSRTKSIAEQMVL  3p.2 4p.0
SDR42E2 P. bivittatus  VLevCKRQSIPRLIYTSSVNVVFAGQTIIDGDEATVPYFPLEqqVNEYSRTKSIAEQMVL  3p.2 4p.0
SDR42E2 X. tropicalis  VIdvCVQRSIPRLIYTSTVNVVFGGNPIEDGDEETVTYFPLEkqIDPYSRSKTIADQMIL  3p.2 4p.0
SDR42E2 L. oculatus    VInvCLQRSIPRLVYTSSVNVVFAGSPIEQGDEETVPCVPLDrhVDHYSKTKAIADQAVL  4p.2 5p.0
                           *       * ***  ** * *  *  * *      *       **  *  *    *

SDR42E1 H  sapiens     EANATPLDRGDGVLRTCALRPAGIYGPGEQRHLPRIVSYIEKGLFKFVYGDPRSLVEFVH
SDR42E1 P. troglodytes EANATPLDRGDGVLRTCALRPAGIYGPGEQRHLPRIVSYIEKGLFKFVYGDPRSLVEFVH
SDR42E1 M. musculus    EANGLAFKQGDGILRTCAIRPAGIYGAGEQRHLPRIVSYIERGLFRFVYGDPQSLVEFVH
SDR42E1 S. harrisii    EANGTALDRGTGVLRTCVLRSAGIYGPGEQRHLPRIVKYIEKGLFKFVYGDPKSLVDFVH
SDR42E1 G. gallus      KANGTELGNGKGVLRTCALRPAGIYGPGEQRHLPRIVSYIERGLFKFVYGDPLSLVEFVH
SDR42E1 P. bivittatus  EANGTVLRDGQGILRTCALRPAGIYGPGEQRHLPRIVNYIERGWFRFVYGDPSSLVDFVH
SDR42E1 X. tropicalis  KMNNQELKNKSGFLKTCSLRAAGIYGPGEQRHLPRIRSVLEKGMFLFIYGDN-PLVQFVH
SDR42E1 L. oculatus    RANGTALRGGAGLLRTCALRPAGIYGPGERRHLPRIVRYIESGIFRFVYGDAGSLVEFVH
SDR42E2 H. sapiens     MANGMPLpg-GGTLRTCVLRPPGIYGPEEQRHLPRVagHIKKRLFMFRFGDHKARMNWVH  5p.2 6p.0
SDR42E2 P. troglodytes MANGTPLpg-GGTLRTCVLRPPGIYGPEEQRHLPRVagHIKKRLFMFRFGDRKARMNWVH  5p.2 6p.0
SDR42E2 M. musculus    MANGTPLlg-GGTLRTCVLRPPGIYGPEEQRHLPRVasHIKKRLFMFRFGDRKTRMNWVH  5p.2 6p.0
SDR42E2 S. harrisii    TCNGTPLpg-GGTLRTCVLRPPGIYGPEEQRHLPRVagSIRKRLFTFKFGDPRTRMNWVH  5p.2 6p.0
SDR42E2 G. gallus      AANGTPLag-GGVLYTSVLRPPGIYGPEEQRHLPRLakNIERGILSFKFGDPSAKMNWVH  5p.2 6p.0
SDR42E2 P. bivittatus  AANGSRLag-GGKLHTCAIRPPGIYGPEEQRHLPRLalNIERGIFNTRVGDPETLMNWVH  5p.2 6p.0
SDR42E2 X. tropicalis  AADGRPLkg-GGKLHTCVLRPPGIYGPDEQRHLPRLvsNIEKGLIFFTVGSQKTFLNWIH  5p.2 6p.0
SDR42E2 L. oculatus    AANGRTLrg-GGLLRTCVLRPPGIYGPEERRHLQRVavNIERRLFSFSFGDREAKMNWVH  6p.2 7p.0
                                  * * *   *  ****  * *** *              *         *

SDR42E1 H. sapiens     VDNLVQAHILASEALRADKGHIASGQPYFISDGRPVNNFEFFRPLVEGLGYTFPSTRLPL
SDR42E1 P. troglodytes VDNLVQAHILASEALRADKGHIASGQPYFISDGRPVNNFEFFRPLVEGLGYTFPSTRLPL
SDR42E1 M. musculus    VDNLAKAHILASEALKADKGHVASGQPYFISDGRPVNNFEFFRPLVEGLGYTFPSTRLPL
SDR42E1 S. harrisii    VDNLVQAHILASEALKADKKHIASGQAYFISDGRPVNNFEFFRPLVEGLGYPFPTIRLPL
SDR42E1 G. gallus      VDNLVQAHILAFEALKANKKHIAAGQAYFISDGRPVNNFEFFRPLVEGLGYKFPTCRLPL
SDR42E1 P. bivittatus  VDNLVQAHILAAEALGATKKHIAAGQPYFISDGRPVNNFEFFRPLVENLGYAFPTLHLPL
SDR42E1 X. tropicalis  VDNLISAHILAAEALTSEKKYIAAGQPYFISDGPPVNNFDFFRPFVEGLGYKFPTLQLPL
SDR42E1 L. oculatus    VDNLAAAHVLAAEALTAAKRHRAAGQAYFISDGRPVNNFEFFRPLVEGLGYPFPRLRLPV
SDR42E2 H. sapiens     VHNLVQAHVLAAEALTTAKGYVasGQAYYINDGESVNLFEWMAPlfEKLGYSQPWIQVPT  7p.0 8p.0
SDR42E2 P. troglodytes VHNLVQAHVLAAEALTTAKGYVasGQAYYINDGESVNLFEWMAPlfEKLGYSQPWIQVPT  7p.0 8p.0
SDR42E2 M. musculus    VQNLVQAHMLAAEGLTMAKGYVasGQAYYINDGESVNLFEWMAPlfEKLGYSQPWIQVPT  7p.0 8p.0
SDR42E2 S. harrisii    VQNLVQAHILAAEALTANKKYIasGQAYYINDGESVNLFEWMSPlfDKMGYSRPWIQIPT  7p.0 8p.0
SDR42E2 G. gallus      VENLVQAQILAAEALTPEKNYIasGQVYFIHDGEKFNLFEWLAPlfERLGCSKPWIPIPT  7p.0 8p.0
SDR42E2 P. bivittatus  VENLVQAHILAAKALTPEMNYIagGQVYFINDDEKVNLFEWLSPlfEGLGSHNPWIRVPV  7p.0 8p.0
SDR42E2 X. tropicalis  LYNLVEAHILAAEALTASKGYIasGQSYFIHDGENVNIYEFLHPlfEKLAFSDPWISLPY  7p.0 8p.0
SDR42E2 L. oculatus    VDNLVMAHVLAAEGLTAGKGFVasGQAYYINDGESVNLFEWLTPlfEKLGYSRPLIHLPV  8p.0 9p.0
                         **  *  **   *       * ** * * *    *      *         *    *

SDR42E1 H. sapiens     TLVYCFAFLTEMVHFILGRLYNFQPFLTRTEVYKTGVTHYFSLEKAKKELGYKAQPFDLQ
SDR42E1 P. troglodytes TLVYCFAFLTEMVHFILGRLYNFQPFLTRTEVYKTGVTHYFSLEKAKKELGYKAQPFDLQ
SDR42E1 M. musculus    TLIYCLAFLVEMTHFIVGRLYNFQPFLTRTEVYKTGVTHYFSLEKAKKELGFEPQPFDLQ
SDR42E1 S. harrisii    NLIYFIAFMTEMVYFLLGRFYNFQPFLTRAEVYKTGVTHYFSMEKARKELHYEPQPFDLK
SDR42E1 G. gallus      SLVYFFAFLTEVVHLLVGHVYNFQPLLTRTEVYKTGVTHYFSMEKARKELGYEPQKYSLN
```

```
SDR42E1 P. bivittatus  SLVYFFAFFTELVHFVVGRLYNFQPLLTRTEVYKTGVTHFFSLAKARRELGYEPQQYSLG
SDR42E1 X. tropicalis  WFIYFLAFLIEWIHFFVSPVCDFQPFLTRSEVCKTGVTHYFSIEKAKRELGFEPQPFTMQ
SDR42E1 L. oculatus    SLVYFFAFLTEMVHFLVGRLYNFQPLLTRTEVYKTGVTHYFSMAKAEEELGYEPRRYSLD
SDR42E2 H. sapiens     SWVYLtaAVMERLHLALRPICSLPPLLTRSevRSVAVTHTFQIAKARAQLGYAPDKFRFA  9p.2 10p.0
SDR42E2 P. troglodytes SWVYLtaAVMERLHLALRPICSLPPLLTRSevRSVAVTHTFQIAKARAQLGYAPDKFRFA  9p.2 10p.0
SDR42E2 M. musculus    SCVYLtaAVMEYLHLALRPICTIPPLLTRSevLSVAVTHTFQIAKARTQLGYAPDKFSFA  9p.2 10p.0
SDR42E2 S. harrisii    SLAYLsaSGMEYLHLALKPICDFPPLLTRSevWSIAVTHTFQIRKARDHLGYTPEKFAFA  9p.2 10p.0
SDR42E2 G. gallus      SLVYAsaTVMEHLHLILKPLVELSPLLTRNevQNISTTHTFRIDKARRQLGYSPEKFTFA  9p.2 10p.0
SDR42E2 P. bivittatus  FLVHLsaVIMENVHSMLLPIVEITPPITRNevHNMACTHTFKIDKARAHLGYAPQKYSFA  9p.2 10p.0
SDR42E2 X. tropicalis  SLVYDsaIFFEYLHLALRPFVNLNPVLTRYevLKISKTHTFRINKACKELGYCPKKFSFA  9p.2 10p.0
SDR42E2 L. oculatus    SLVYTaaILMECLHVALRPVVGIPLLFTRNevRNIAVTHTFRIDKARRQLGFTPQRRDLA 10p.2 11p.0
                             *   *                ** **      ** *   **   *

SDR42E1 H. sapiens     EAVEWFKAHGHGRSSGSRDS--ECFVWDGLLVFLLIIA-VLMWLP---SSVILSL-----
SDR42E1 P. troglodytes EAVEWFKAHGHGRSSGSRDS--ECFVWDGLLVFLLIIA-VLMWLP---SSVILSL-----
SDR42E1 M. musculus    EVVEWFKAHGHGRGAAGQDS--EFMLWDGILILLLALS-VLTWIL---PSTTLSI-----
SDR42E1 S. harrisii    EVVDWFKAEGHGRKFQNYTL--KHLIWNGMLIFFLAIA-ILTWFS---NDHGDSS--LKD
SDR42E1 G. gallus      EVVEWFRSQGCGPKPRKYTI--THLLRDGGLLLLFIAV-LVSWFP---PAVIFSP-----
SDR42E1 P. bivittatus  EVVEWFQAHGHGQKFSMSSG--KHFMRNAALAFLVVGM-VFLWFP---RFSWSFF-----
SDR42E1 X. tropicalis  EVAEWFKNHGYGKQDKKFKR--NYFIWDIIFILLVAVV-LLSWHS---KLIGTSE-----
SDR42E1 L. oculatus    DVVQWFKDKGHGRKPRSPSF--KQLLRDVFLMAMLVAV-IFSYIP---VVGT--------
SDR42E2 H. sapiens     DAVELYVQSTTRRPRGSTARTLL--------RLLLRLLLFLGLLALALH-----FLGLQP
SDR42E2 P. troglodytes DAVELYVQSTTRRPRGSTARTLL--------RLLLGLLLFLGLLALALH-----FLGLQP
SDR42E2 M. musculus    DAVERYVQATTPQPRCCIVLTLL--------RLLLMLLLLFALLGLALY-----FLGLQP
SDR42E2 S. harrisii    DSVDHYIQTWHKQQRHSP--RFL--------RLLMVLICLLGTLLLSLSLLDPSFLGLSH
SDR42E2 G. gallus      DSVEHYIKTRAEARSEHGFP-----------RVMLLFLAILSLIFLSLRFDD-----LSV
SDR42E2 P. bivittatus  DCVDHFLKKGPRSRNFFL-C-----------KCLFLLVLLTGLIVLAVKFSqtSLccKGR  11p.2 12p.1
SDR42E2 X. tropicalis  DSVDFYIKTRPQPLQPHYILMR---------KLFLLLLGFFSLFAFLYYSGS--ALSLAR
SDR42E2 L. oculatus    EAVDGYLKTRARSPRRLPACLRAALLLGGALALLLLLSCAWGDLAAPRSLNS-----TRP


SDR42E1 X. tropicalis  ------------------------------------------------------------
SDR42E1 L. oculatus    ------------------------------------------------------------
SDR42E1 S. harrisii    -LF---------------------------------------------------------
SDR42E1 M. musculus    ------------------------------------------------------------
SDR42E1 H. sapiens     ------------------------------------------------------------
SDR42E1 P. troglodytes ------------------------------------------------------------
SDR42E1 G. gallus      ------------------------------------------------------------
SDR42E1 P. bivittatus  ------------------------------------------------------------
SDR42E2 H. sapiens     --LHAAVERL--------------------------------------------------
SDR42E2 P. troglodytes --LHAAVERL--------------------------------------------------
SDR42E2 M. musculus    --LQAVVERL--------------------------------------------------
SDR42E2 S. harrisii    --GNYM------------------------------------------------------
SDR42E2 G. gallus      --LHFFKEAQH-------------------------------------------------
SDR42E2 P. bivittatus  ALPKRPRERAGSLQGPRREEKPGRSGLGETRSKGLRSAPDRQRGSPGGCYCTRRAPGGAA
SDR42E2 X. tropicalis  ------------------------------------------------------------
SDR42E2 L. oculatus    ------------------------------------------------------------


SDR42E1 H. sapiens     ------------------------------------------------------------
SDR42E1 P. troglodytes ------------------------------------------------------------
SDR42E1 M. musculus    ------------------------------------------------------------
SDR42E1 S. harrisii    ------------------------------------------------------------
SDR42E1 G. gallus      ------------------------------------------------------------
SDR42E1 P. bivittatus  ------------------------------------------------------------
SDR42E1 X. tropicalis  ------------------------------------------------------------
```

```
SDR42E1 L. oculatus    -------------------------------------------------------------
SDR42E2 H. sapiens     -------------------------------------------------------------
SDR42E2 P. troglodytes -------------------------------------------------------------
SDR42E2 M. musculus    -------------------------------------------------------------
SDR42E2 S. harrisii    -------------------------------------------------------------
SDR42E2 G. gallus      -------------------------------------------------------------
SDR42E2 P. bivittatus  LQRFRLQLHRSFRKKSraFGGGALSWLQERLAALEMELHYGDPIFSEGQPEQGAKAKGSSG 13p.1
SDR42E2 X. tropicalis  -------------------------------------------------------------
SDR42E2 L. oculatus    -------------------------------------------------------------
```

Figure S24. Alignment of the vertebrate SDR42E family variants. For further details see Fig. S13.

* symbol marks the position of identical amino acid residues of the aligned SDR protein sequences.

| Species | Gene symbol | Gene ID | Chr[4] | Enzyme symbol | Exons n. | Phases formula | % identity | aa n. | Structure consensus | Catalytic consensus |
|---|---|---|---|---|---|---|---|---|---|---|
| Homo sapiens | | 51109 | 14 | | | | 100.00 | 318 | | |
| Gorilla gorilla | | 101134301 | 14 | | | | 99.69 | | | YCHSK |
| Mus musculus | RDH11 | 17252 | 12 | SDR7C1 | 7 | 122221 | 85.08 | 316 | GANTGIG | |
| Monodelphis domestica | | 100028370 | 1 | | | | 77.71 | 316 | | |
| Poecilia formosa | | 103144764 | - | | | | 60.38 | 318 | | YCQSK |
| | | | | | | | | | | |
| Homo sapiens | | 145226 | 14 | | | | 100.00 | | | |
| Pan troglodytes | | 100615509 | 14 | | | | 100.00 | 316 | | |
| Mus musculus | RDH12 | 77974 | 12 | | | | 86.39 | | | YCHSK |
| Monodelphis domestica | | 100028379 | 1 | SDR7C2 | 7 | 122221 | 80.19 | 313 | GANTGIG | |
| Gallus gallus | | 423274 | 5 | | | | 68.67 | 326 | | |
| Anolis carolinensis | LOC100556987 | 100556987 | - | | | | 67.55 | | | YCQSK |
| Danio rerio | RDH12 | 436597 | 13 | | | | 62.62 | 302 | GANSGIG | |
| | | | | | | | | | | |
| Homo sapiens | RDH13 | 112724 | 19 | | | | 100.00 | | | |
| Pan troglodytes | | 456293 | 19 | | | | 99.70 | 331 | | |
| Mus musculus | Rdh13 | 108841 | 7 | SDR7C3 | 7 | 122222 | 84.29 | 334 | GANTGIG | YCQSK |
| Xenopus tropicalis | rdh13 | 496409, | - | | | | 72.64 | 329 | | |
| Oryzias latipes | LOC101164512 | 101164512 | 19 | | | | 68.88 | 336 | | |
| | | | | | | | | | | |
| Homo sapiens | RDH14 | 57665 | 2 | | | | 100 | | | |
| Pan troglodytes | | 470316 | 2A | | | | 100.00 | 336 | | |

| | | | | | | | | | | |
|---|---|---|---|---|---|---|---|---|---|---|
| *Mus musculus* | Rdh14 | 105014 | 12 | | | | 90.09 | 334 | GANSGLG | YSRSK |
| *Monodelphis domestica* | RDH14 | 103093572 | - | SDRC4 | 2 | 0 | 78.85 | 338 | | YSQSK |
| *Pseudopodices humilis* | | 102101217 | - | | | | 81.93 | 321 | | |
| *Anolis carolinensis* | LOC100557468 | 100557468 | 1 | | | | 79.09 | 330 | | YSRSK |
| *Xenopus tropicalis* | rdh14 | 549985 | - | | | | 77.19 | 323 | GANCGIG | |
| *Danio rerio* | | 334665 | 20 | | | | 67.18 | | | YSQSK |
| | | | | | | | | | | |
| *Homo sapiens* | DHRS13 | 147015 | 17 | | | | 100.00 | 377 | GANSGIG | YADTK |
| *Pongo abelii* | | 100452796 | 17 | | | | 98.67 | | | |
| *Mus musculus* | Dhrs13 | 70451 | 11 | | | | 84.76 | 375 | | YADSK |
| *Monodelphis domestica* | | 100025100 | 2 | | | | 73.64 | 349 | GGNTGIG | |
| *Haliaeetus leucocephalus* | DHRS13 | 104835594 | - | SDRC5 | 5 | 2022 | 59.15 | 328 | GGSSGIG | YCDSK |
| *Gekko japonicus* | | 107119410 | - | | | | 58.33 | 313 | GGNTGIG | YCNSK |
| *Xenopus tropicalis* | dhrs13 | 394935 | - | | | | 57.32 | 314 | GANVGIG | YCDSK |
| *Oryzias latipes* | LOC101160574 | 101160574 | 13 | | | | 49.84 | 318 | GSNTGIG | |
| | | | | | | | | | | |
| *Homo sapiens* | | 79758 | 13 | | | | 100% | | | |
| *Pan troglodytes* | DHRS12 | 743256 | 13 | | | | 96.53 | 317 | GGNSGIG | |
| *Bos taurus* | | 507276 | 12 | | | | 85.49 | | | |
| *Gallus gallus* | | 770438 | 1 | SDR40C1 | 10 | 120020022 | 71.61 | 327 | GSNSGIG | YAQNK |
| *Anolis carolinensis* | dhrs12 | 10056528 | 1 | | | | 70.66 | 319 | | |
| *Xenopus tropicalis* | | 780002 | - | | | | 67.82 | 323 | GANSGIG | |
| *Oryzias latipes* | LOC101155475 | 101155475 | 21 | | | | 66.25 | 318 | | |

Table S15. Genetic and molecular data of the vertebrate SDR7C family and of the SDR40C variants. Chr, chromosome; Phases formula contains the phase types symbols aligned according to the sequence of their relative splicing sites; conserved amino acids of the consensus sequences are in red; aa n., number of the variant amino acids.

| Species | Gene symbol | Gene ID | Chr | Enzyme symbol | Exons n. | Phases formula | % identity | aa n. | Structure consensus | Catalysis consensus |
|---|---|---|---|---|---|---|---|---|---|---|
| *Homo sapiens* | BDH1 | 622 | 3 | SDR9C1 | 6 | 10022 | 100.00 | 343 | GCDSGFG | YCITK |
| *Pan paniscus* | | 100967469 | 3 | | | | 99.42 | | | |
| *Mus musculus* | Bdh1 | 71911 | 16B2 | | | | 85.71 | | | |
| *Monodelphis domestica* | BDH1 | 100032119 | - | | | | 69.71 | 340 | | |
| *Columba livia* | | 102092067 | - | | | | 72.59 | 343 | | |
| *Chrysemys picta* | | 101941677 | - | | | | 71.72 | 344 | | |
| *Xenopus tropicalis* | bdh1 | 100495647 | - | | | | 74.85 | 330 | | |
| *Danio rerio* | | 100037356 | 24 | | | | 54.71 | 359 | GCDTGFG | YCISK |
| | | | | | | | | | | |
| *Homo sapiens* | HSD17B2 | 3294 | 16 | SDR9C2 | 5 | 2222 | 100.00 | 387 | GGDCGLG | YGSSK |
| *Pan troglodytes* | | 454269 | 16 | | | | 99.48 | | | |
| *Mus musculus* | Hsd17b2 | 15486 | 8 | | | | 59.37 | 381 | GADSGFG | YAATK |
| *Monodelphis domestica* | LOC100031815 | 100031815 | 1 | | | | 52.28 | 383 | GGDSGIG | YCASK |
| *Gallus gallus* | HSD17B2 | 415807 | 11 | | | | 53.40 | 384 | GSDTGIG | YGASK |
| *Alligator mississippiensis* | HSD17B2 | 102574309 | - | | | | 61.61 | 310 | GSDSGIG | |
| *Xenopus tropicalis* | hsd11b2 | 100497002 | - | | | | 45.92 | 381 | GCDSGFG | YASSK |
| *Danio rerio* | | 449537 | 25 | | | | 38.97 | 358 | SCDSGFG | YGASK |
| | | | | | | | | | | |
| *Homo sapiens* | HSD11B2 | 3291 | 16 | | | | 100.00 | 405 | GCDSGFG | YGTSK |
| *Pan troglodytes* | | 468001 | 16 | | | | 99.75 | | | |
| *Mus musculus* | Hsd11b2 | 15484 | 8 | | | | 86.53 | 385 | GCDTGFG | |

| | | | | | | | | | | |
|---|---|---|---|---|---|---|---|---|---|---|
| *Gallus gallus* | HSD11B2 | 100858361 | 11 | SDR9C3 | 5 | 2222 | 58.49 | 388 | GCDSGFG | YGASK |
| *Chrysemys picta* | HSD11B2 | 101949646 | - | | | | 59.79 | 383 | GCDSGFG | |
| *Xenopus tropicalis* | hsd11b2 | 100489854 | - | | | | 45.95 | 368 | | YGSSK |
| *Danio rerio* | | 334098 | 7 | | | | 42.47 | 414 | | YGASK |
| | | | | | | | | | | |
| *Homo sapiens* | DHRS9 | 10170 | 2 | SDR9C4 | 4 | 212 | 100.00 | 319 | GCDSGFG | YTPSK |
| *Pan troglodytes* | | 470578 | 2B | | | | 99.37 | | | |
| *Mus musculus* | Dhrs9 | 241452 | 2 | | | | 85.89 | | GCDTGFG | |
| *Monodelphis domestica* | DHRS9 | 100017287 | 4 | | | | 77.43 | | | |
| *Gallus gallus* | | 424169 | 7 | | | | 59.75 | 321 | | YCPSK |
| *Chelonia mydas* | | 102934689 | - | | | | 65.20 | 322 | GCDSGFG | YVPSK |
| *Xenopus tropicalis* | dhrs9 | 407852 | - | | | | 57.55 | | GCDTGFG | YFSSK |
| *Danio rerio* | | 322529 | 9 | | | | 45.74 | 319 | | YCITK |
| | | | | | | | | | | |
| *Homo sapiens* | RDH5 | 5959 | 12 | SDR9C5 | 4 | 212 | 100.00 | 318 | GCDSGFG | YCVSK |
| *Pan troglodytes* | | 467029 | 12 | | | | 99.37 | | | |
| *Mus musculus* | Rdh5 | 19682 | 10 | | | | 87.11 | | | |
| *Sarcophilus harrisii* | RDH5 | 100926680 | - | | | | 76.73 | | | |
| *Gallus gallus* | | 395452 | 33 | | | | 66.04 | 319 | | |
| *Anolis carolinensis* | rdh5 | 100561257 | - | | | | 61.32 | | GCDTGFG | YCISK |
| *Xenopus tropicalis* | RDH5 | 496830 | - | | | | 58.49 | | | YCVSK |
| *Danio rerio* | | 556528 | 22 | | | 1212 | 53.31 | 328 | GCDSGFG | YCISK |
| | | | | | | | | | | |
| *Homo sapiens* | HSD17B6 | 8630 | 12 | | | | 100.00 | 317 | | YCVSK |
| *Pan paniscus* | | 100996145 | 12 | | | | 99.05 | | | |
| *Mus* | | | | | | | | | | |

| | | | | | | | | | | |
|---|---|---|---|---|---|---|---|---|---|---|
| *musculus* | Hsd17b6 | 27400 | 10 | SDR9C6 | 4 | 212 | 73.19 | | GCDSGFG | YSCSK |
| *Sarcophilus harrisii* | LOC100931687 | 100931687 | 5 | | | | 65.30 | | | YCSSK |
| *Anolis carolinensis* | hsd17b6 | 100564174 | 2 | | | | 64.04 | 318 | | YCPSK |
| *Xenopus tropicalis* | | 496749, | - | | | | 54.29 | | | YCLSK |
| | | | | | | | | | | |
| *Homo sapiens* | SDR9C7 | 121214 | 12 | SDR9C7 | 4 | 212 | 100.00 | 313 | GCDSGFG | YCVSK |
| *Pan troglodytes* | | 467039 | 12 | | | | 99.36 | | | |
| *Mus musculus* | Sdr9c7 | 70061 | 10 | | | | 84.66 | | | |
| *Sarcophilus harrisii* | SDR9C7 | 100929345 | - | | | | 78.91 | 314 | | |
| *Xenopus tropicalis* | LOC100170425 | 100170425 | - | | | | 48.88 | 319 | | YSISK |
| | | | | | | | | | | |
| *Homo sapiens* | RDH16 | 8608 | 12 | SDR9C8 | 4 | 212 | 100.00 | 317 | GCDSGFG | YCISK |
| *Pan troglodytes* | | 467206 | 12 | | | | 99.05 | | | |
| *Mus musculus* | Rdh16 | 19683 | 10 | | | | 73.19 | | | |
| *Monodelphis domestica* | RDH16 | 100011050 | - | | | | 67.82 | | | |
| *Pseudopodices humilis* | LOC102113964 | 102100504 | - | | | | 64.67 | 318 | | |
| *Anolis carolinensis* | LOC100567570 | 100567570 | 2 | | | | 67.51 | 317 | | YCPSK |
| *Xenopus tropicalis* | RDH16 | 100135184 | - | | | | 57.91 | | | YSISK |
| *Poecilia reticulata* | LOC103461718 | 103461718 | - | | | | 51.10 | 319 | | YCVSK |

Table S16. Genetic and molecular data of the vertebrate SDR9C family variants. The atypical amino acid of *Danio rerio* SDR9C2 structure consensus is highlighted in turquoise. For further details see Table S15.

| Species | Gene symbol | Gene ID | Chr | Enzyme symbol | Exons n. | Phases formula | % 1dentity | aa n. | Structure consensus | Catalysis consensus |
|---|---|---|---|---|---|---|---|---|---|---|
| *Homo sapiens* | FAR1 | 84188 | 11 | SDR10E1 | 11 | 0110012101 | 100 | 515 | GATGFLG | YIYTK |
| *Pan troglodytes* | | 451036 | 11 | | | | 99.81 | | | |
| *Mus musculus* | Far1 | 67420 | 7 | | | | 98.06 | | | |
| *Sarcophilus harrisii* | FAR1 | 100920845 | - | | | | 97.48 | | | |
| *Gallus gallus* | | 423028 | 5 | | | | 89.71 | | GATGFMG | YTYTK |
| *Chelonia mydas* | | 102930779 | - | | | | 89.71 | | | |
| *Xenopus tropicalis* | far1 | 100038270 | - | | | | 85.63 | | | |
| *Danio rerio* | | 406829 | 25 | | | | 78.06 | 518 | | |
| *Homo sapiens* | FAR2 | 55711 | 12 | SDR10E2 | 11 | 0110012101 | 100 | 515 | GATGFLG | YTYTK |
| *Pan troglodytes* | | 473394 | 12 | | | | 99.61 | | | |
| *Mus musculus* | Far2 | 330450 | 6 | | | | 87.96 | | | |
| *Monodelphis domestica* | FAR2 | 100013219 | 8 | | | | 78.83 | | GATGFMG | |
| *Gallus gallus* | | 419043 | 1 | | | | 63.50 | | | |
| *Anolis carolinensis* | far2 | 100565436 | 5 | | | | 62.14 | | | YTFTK |
| *Esox lucius* | | 105023414 | - | | | | 62.14 | | | YTYTK |

Table S17. Genetic and molecular data of the vertebrate SDR10E family variants. For further details see Table S15.

| Species | Gene symbol | Gene ID | Chr | Enzyme symbol | Exons n. | Phases formula | % identity | aa n. | Structure consensus | Catalysis consensus |
|---|---|---|---|---|---|---|---|---|---|---|
| *Homo sapiens* | HSD3B1 | 3283 | 1 | SDR11E1 | 3 | 22 | 100.00 | 375 | GAGGFLG | YPHSK |
| *Pan troglodytes* | | 469446 | 1 | | | | 99.20 | | | |
| *Mus Musculus* | Hsd3B1 | 15492 | 3 | | | | 71.58 | 373 | GAGGFVG | YPYSK |
| *Monodelphis domestica* | LOC100012668 | 100012668 | 2 | | | | 62.93 | 377 | GAGGFLG | |
| *Nipponia nippon* | LOC104008615 | 104008615 | - | | | | 55.73 | | | YAQSK |
| *Thamnophis sirtalis* | LOC106542700 | 106542700 | - | | | | 50.67 | | | YAETK |
| *Larimichthys crocea* | hsd3B1 | 104921488 | - | | | | 46.65 | 374 | GACGFLG | YSKTK |
| *Homo sapiens* | HSD3B2 | 3284 | 1 | SDR11E2 | 3 | 22 | 100 | 372 | GAGGLLG | YPYSK |
| *Pan troglodytes* | LOC457165 | 457165 | 1 | | | | 98.92 | | | |
| *Mus musculus* | Hsd3b2 | 15493 | 3 | | | | 70.70 | 373 | GAGGFLG | |
| *Sarcophilus harrisii* | HSD3B2 | 100913530 | - | | | | 61.83 | 377 | | |
| *Gallus Gallus* | | 396015 | 1 | | | | 55.91 | | | YAQSK |
| *Gekko japonicus* | LOC107114856 | 107114856 | - | | | | 49.73 | | | YAETK |
| *Danio rerio* | hsd3b2 | 373131 | 20 | | | | 44.50 | 374 | GACGFLG | YSKTK |
| *Homo sapiens* | HSD3B7 | 80270 | 16 | SDR11E3 | 6 | 22102 | 100 | 369 | GGCGFLG | YPCSK |
| *Pan troglodytes* | | 454059 | 16 | | | | 98.92 | | | |
| *Mus musculus* | Hsd3b7 | 101502 | 7 | | | | 86.99 | | | |
| *Sarcophilus harrisii* | HSD3B7 | 100929800 | - | | | | 73.02 | 370 | | YPRSK |
| *Zonotrichia albicollis* | | 102073947 | - | | | | 43.37 | 371 | GGCGFVG | YGKTK |
| *Anolis carolinensis* | hsd3b7 | 100555869 | - | | | | 65.01 | 366 | GGCGFLG | YPVSK |
| *Xenopus tropicalis* | | 100145626 | - | | | | 60.38 | 380 | | YPLSK |
| *Danio rerio* | | 327462 | 3 | | | | 54.12 | 368 | | YPKSK |

Table S18. Genetic and molecular data of the vertebrate SDR11E family variants. For further details see Table S15.

| Species | Gene symbol | Gene ID | Chr | Enzyme symbol | Exons n. | Phases formula | % identity | aa n. | Structure consensus | Catalysis consensus |
|---|---|---|---|---|---|---|---|---|---|---|
| *Homo sapiens* | HSD17B12 | 51144 | 11 | SDR12C1 | 11 | 2022001000 | 100 | 312 | GSTDGIG | YSATK |
| *Pan troglodytes* | | 741010 | 11 | | | | 99.68 | | | |
| *Mus musculus* | Hsd17b12 | 56348 | 2 | | | | 81.09 | | GGTDGIG | |
| *Monodelphis domestica* | HSD17B12 | 100013277 | 5 | | | | 77.27 | 309 | GSTDGIG | |
| *Zonotrichia albicollis* | | 102062927 | - | | | | 67.31 | 316 | GATDGIG | |
| *Anolis carolinensis* | hsd17b12 | 100557660 | 1 | | | | 57.05 | 317 | | |
| *Xenopus tropicalis* | | 549988 | - | | | | 60.90 | 320 | | |
| *Poecilia latipinna* | | 106964816 | - | | | | 60.58 | | | |
| *Homo sapiens* | HSD17B3 | 3293 | 9 | SDR12C2 | 11 | 2022001000 | 100 | 310 | GAGDGIG | YSASK |
| *Pan troglodytes* | | 742116 | 9 | | | | 98.71 | | | |
| *Mus musculus* | Hsd17b3 | 15487 | 13 | | | | 73.11 | 305 | | |
| *Sarcophilus harrisii* | HSD17B3 | 100935109 | 2 | | | | 69.68 | 310 | | YSSTK |
| *Gallus gallus* | | 427474 | Z | | | | 59.35 | | GAGDGLG | YSASK |
| *Anolis carolinensis* | hsd17b3 | 100561157 | 2 | | | | 55.20 | 264 | GAGDGIG | |
| *Xenopus tropicalis* | | 101730254 | - | | | | 57.28 | 302 | | |
| *Danio rerio* | | 393335 | 8 | | | | 45.07 | 307 | GGSDGIG | YAASK |
| *Homo sapiens* | HSDL1 | 83693 | 16 | | | | 100 | 330 | GATDGIG | FSASK |
| *Pan troglodytes* | | 741546 | 16 | | | | 99.39 | | | |
| *Mus musculus* | Hsdl1 | 72552 | 8 | | | | 87.88 | | | |
| *Sarcophilus harrisii* | | 100918948 | - | | | | 86.06 | | | YSASK |

| | | | | | | | | | | |
|---|---|---|---|---|---|---|---|---|---|---|
| *Gallus gallus* | HSDL1 | 415703 | 11 | SDR12C3 | 4 | 100 | 65.15 | 331 | GSTDGIG | YGASK |
| *Gekko japonicus* | | 107106023 | - | | | | 65.76 | | GGTSGIG | YSASK |
| *Xenopus tropicalis* | hsdl1 | 100489686 | - | | | | 50.00 | 322 | GATSGIA | YTPCQ |
| *Lepisosteus oculatus* | | 102682551 | LG1 | | | | 69.84 | 319 | GASDGLG | YSASK |

Table S19. Genetic and molecular data of the vertebrate SDR12C family variants. The atypical amino acids of the structure and catalytic consensuses are highlighted in turquoise. For further details see Table S15 and Consensuses in Online Resources 1.

| Species | Gene symbol | Gene ID | Chr | Enzyme symbol | Exons n. | Phases formula | % identity | aa n. | Structure consensus | Catalysis consensus |
|---|---|---|---|---|---|---|---|---|---|---|
| *Homo sapiens* | DRSH3 | 9249 | 1 | SDR16C1 | 6 | 00011 | 100 | 302 | GGGRGIG | YCTSK |
| *Pan troglodytes* | | 737006 | 1 | | | | | | | |
| *Mus musculus* | dhrs3 | 20148 | 4 | | | | 95.03 | | | |
| *Gallus gallus* | DHRS3 | 419480 | 21 | | | | 90.07 | | | |
| *Gekko japonicus* | | 107124081 | - | | | | 87.75 | | | |
| *Xenopus tropicalis* | dhrs3 | 493258 | - | | | | 83.77 | | | |
| *Danio rerio* | dhrs3a | 445083 | 23 | | | | 77.15 | | | |
| *Homo sapiens* | HSD17B11 | 51170 | 4 | SDR16C2 | 7 | 000111 | 100 | 300 | GAGHGIG | YCSSK |
| *Pan troglodytes* | | 461364 | 4 | | | | 100 | | | |
| *Mus musculus* | Hsd17b11 | 14664 | 5 | | | | 83.22 | 298 | | |
| *Sarcophilus harrisii* | HSD17B11 | 100917981 | 6 | | | | 78.00 | 300 | | |
| *Gallus gallus* | LOC428754 | 42875 | 4 | | | | 61.87 | 299 | GAARGLG | |
| *Alligator mississippiensis* | HSD17B11 | 102560079 | - | | | | 63.88 | | GAGHGLG | |
| *Xenopus tropicalis* | hsd17b11 | 496757 | - | | | | 59.67 | 300 | GAGHGIG | YCSTK |
| *Homo sapiens* | HSD17B13 | 345275 | 4 | | | | 100 | | | |
| *Pan troglodytes* | | 471245 | 4 | | | | 98.33 | | | |

| | | | | | | | | | | |
|---|---|---|---|---|---|---|---|---|---|---|
| *Mus musculus* | Hsd17b13 | 243168 | 5 | SDR16C3 | 7 | 000111 | 81.33 | 300 | GAGHGIG | YCSSK |
| *Sarcophilus harrisii* | HSD17B13 | 100918501 | 6 | | | | 79.33 | | | |
| *Ficedula albicollis* | LOC101813950 | 101813950 | 4 | | | | 62.88 | 299 | GAGHGVG | |
| *Anolis carolinensis* | LOC100561108 | 100561108 | 5 | | | | 59.29 | 280 | GAGHGLG | YTSSK |
| *Xenopus tropicalis* | hsd17b13 | 496682 | - | | | | 61.33 | 300 | GSGHGIG | YCASK |
| *Homo sapiens* | RDH10 | 157506 | 8 | SDR16C4 | 6 | 20011 | 100 | 341 | GAGSGLG | YCASK |
| *Pan troglodytes* | | 472795 | 8 | | | | 100 | | | |
| *Mus musculus* | Rdh10 | 98711 | 1 | | | | 98.83 | 340 | | |
| *Monodelphis domestica* | RDH10 | 100020635 | 3 | | | | 98.83 | 341 | | |
| *Gallus gallus* | | 420183 | 2 | | | | 92.92 | 339 | | |
| *Gekko japonicus* | | 107114646 | - | | | | 91.50 | 342 | | |
| *Xenopus tropicalis* | rdh10 | 496504 | - | | | | 88.86 | 341 | | |
| *Poecilia latipinna* | | 106955667 | - | | | | 80.53 | 340 | | |
| *Homo sapiens* | SDR16C5 | 195814 | 8 | SDR16C5 | 6 | 00211 | 100 | 309 | GAGSGLG | YCASK |
| *Pan troglodytes* | | 472773 | 8 | | | | 99.68 | | | |
| *Mus musculus* | Sdr16c5 | 242285 | 4 | | | | 79.94% | | | |
| *Monodelphis domestica* | LOC100029976 | 100029976 | 3 | | | | 67.64 | | GAGSGIG | |
| *Gallus gallus* | SDR16C5 | 421130 | 2 | | | | 60.33 | 305 | | |
| *Python bivittatus* | | 103063029 | - | | | | 57.24 | 306 | | YCTSK |
| *Xenopus tropicalis* | sdr16c5 | 448386 | - | | | | 63.28 | 305 | | YCASK |
| *Danio rerio* | sdr16c5a | 405814 | 2 | | | | 56.39 | 306 | GSGSGIG | |
| | sdr16c5b | 406799 | 7 | | | | 60.66 | | GAGSGIG | |

Table S20. Genetic and molecular data of the vertebrate SDR16C family variants. For further details see Table S15.

| Species | Gene symbol | Gene ID | Chr | Enzyme symbol | Exons n. | Phases formula | % identity | aa n. | Structure consensus | Catalysis consensus |
|---|---|---|---|---|---|---|---|---|---|---|
| *Homo sapiens* | CBR1 | 873 | 21 | SDR21C1 | 3 | 22 | 100.00 | 277 | GGNKGIG | YGVTK |
| *Pan troglodytes* | | 473983 | 21 | | | | 100.00 | | | |
| *Mus musculus* | Cbr1 | 12408 | 16 | | | | 87.73 | | GANKGIG | |
| *Monodelphis domestica* | LOC100015233 | 100015233 | 4 | | | | 84.12 | | GSNKGIG | |
| *Gallus gallus* | CBR1 | 418512 | 1 | | | | 78.26 | 276 | | YGVSK |
| *Chrysemys picta* | | 101937374 | - | | | | 78.26 | | GGNKGIG | YGVTK |
| *Xenopus tropicalis* | cbr1 | 496612 | 2 | | | | 72.83 | 277 | | YGVSK |
| *Danio rerio* | | 373866 | 1 | | | | 65.94 | 276 | GANKGIG | YGISK |
| *Homo sapiens* | CBR3 | 874 | 21 | SDR21C2 | 3 | 22 | 100.00 | 277 | GANRGIG | YGVSK |
| *Pan troglodytes* | | 458533 | 21 | | | | 99.64 | | | |
| *Mus musculus* | Cbr3 | 109857 | 16 | | | | 85.20 | | GANKGIG | |
| *Monodelphis domestica* | LOC100015187 | 100015187 | 4 | | | | 66.43 | | GSNKGIG | YGVTK |
| *Xenopus tropicalis* | cbr3 | 100145008 | 2 | | | | 61.96 | | GGNKGIG | YGVSK |

Table S21. Genetic and molecular data of the vertebrate SDR21C family variants. For further details see Table S15.

| Species | Gene symbol | Gene ID | Chr | Enzyme symbol | Exons n. | Phases formula | % identity | aa n. | Structure consensus | Catalysis consensus |
|---|---|---|---|---|---|---|---|---|---|---|
| *Homo sapiens* | DHRS2 | 10202 | 14 | SDR25C1 | 8 | 1001001 | 100.00 | 280 | GSTSGIG | YNVSK |
| *Pan paniscus* | | 100976049 | 14 | | | | 96.79 | | | |
| *Mus musculus* | Dhrs2 | 71412 | 14 | | | | 65.47 | 282 | GSTRGIG | YNTSK |
| *Monodelphis domestica* | DHRS2 | 100030637 | 1 | | | | 67.50 | 281 | GSTQGIG | YNISK |

| | | | | | | | | | | |
|---|---|---|---|---|---|---|---|---|---|---|
| *Homo sapiens* | DHRS4 | 10901 | 14 | SDR25C2 | 8 | 1001001 | 100.00 | 278 | ASTDGIG | YNVSK |
| *Pan troglodytes* | | 452804 | 14 | | | | 98.20 | | | |
| *Mus musculus* | Dhrs4 | 28200 | 14 | | | | 79.14 | 279 | | |
| *Monodelphis domestica* | DHRS4 | 100030652 | 1 | | 9 | 21001001 | 69.42 | 326 | ASTEGIG | |
| *Gallus gallus* | | 426247 | - | | 8 | 1001001 | 54.89 | 273 | AATDGIG | YSVSK |
| *Anolis carolinensis* | LOC100553479 | 100553479 | - | | | | 67.41 | 282 | ASTEGIG | YNVSK |
| *Xenopus tropicalis* | dhrs4 | 548501 | - | | | | 66.28 | 261 | | YSVSK |
| *Danio rerio* | dhrs4 | 393539 | 7 | | | | 58.39 | 276 | ASTDGIG | |

Table S22. Genetic and molecular data of the vertebrate SDR25C family variants. Alanine (A), diagnostic of the vertebrate SDR25C2 structure consensus, is highlighted in turquoise. For further details see Table S15 and Consensuses in Online resources 1.

| Species | Gene symbol | Gene ID | Chr | Enzyme symbol | Exons n. | Phases formula | % identity | aa n. | Structure consensus | Catalysis consensus |
|---|---|---|---|---|---|---|---|---|---|---|
| *Homo sapiens* | HSD11B1 | 3290 | 1 | SDR26C1 | 6 | 20222 | 100.00 | 292 | GASKGIG | YSASK |
| *Pan troglodytes* | | 457698 | 1 | | | | 99.66 | | | |
| *Mus musculus* | Hsd11b1 | 15483 | 1 | | | | 78.42 | | | |
| *Sarcophilus harrisii* | HSD11B1 | 100923255 | 4 | | | | 60.69 | 312 | GASTGIG | YSATK |
| *Gallus gallus* | HSD11B1 | 771930 | 26 | | | | 57.53 | 293 | | |
| *Anolis carolinensis* | hsd11b1 | 100555338 | 4 | | | | 59.93 | | GASTGIG | |
| *Xenopus tropicalis* | hsd11b1l.2 | 100038278 | - | | | | 42.51 | 291 | GSSTGIG | YCASK |
| *Homo sapiens* | HSD11B1L | 374875 | 19 | SDR26C2 | 7 | 202021 | 100.00 | 315 | GANAGVG | YSAAK |
| *Pongo abelii* | | 100460111 | 19 | | | | 82.86 | 287 | | |
| *Ictidomys tridecemlineatus* | Hsd11b1l | 101978515 | - | | | | 75.27 | 288 | GAAGDAGVG | |
| *Nipponia nippon* | HSD11B1L | 104016278 | - | | | | 52.11 | 288 | GASAGIG | YSATK |
| *Anolis carolinensis* | hsd11b1l | 100565341 | - | | | | 51.30 | 290 | GASDGIG | |
| *Xenopus tropicalis* | | 100037875 | - | | | | 45.45 | 286 | GASTGIG | YAASK |

| | | | | | | | | | | |
|---|---|---|---|---|---|---|---|---|---|---|
| *Sinocyclocheilus grahami* | LOC107596225 | 107596225 | - | | | | 43.43 | 285 | GASSGIG | YTSTK |

Table S23. Genetic and molecular data of the vertebrate SDR21C family variants. The atypical structure consensus of *Ictidomys tridecemlineatus* is highlighted in turquoise. For further details see Table S15.

| Species | Gene symbol | Gene ID | Chr | Enzyme symbol | Exons n. | Phases formula | % identity | aa n. | Structure consensus | Catalysis consensus |
|---|---|---|---|---|---|---|---|---|---|---|
| *Homo sapiens* | HSD17B1 | 3292 | 17 | SDR28C1 | 6 | 22210 | 100 | 328 | GCSSGIG | YCASK |
| *Pongo abelii* | | 100456666 | 17 | | | | 96.30 | 324 | | |
| *Mus musculus* | Hsd17b1 | 15485 | 11 | | | | 68.83 | 344 | | |
| *Monodelphis domestica* | HSD17B1 | 100011776 | 2 | | | | 71.15 | 305 | | |
| *Gallus gallus* | | 395641 | 27 | | | | 53.92 | 302 | | |
| *Xenopus tropicalis* | hsd17b1 | 100489679 | - | | | | 53.24 | 295 | | |
| *Danio rerio* | | 402842 | 3 | | | | 51.03 | | | |
| *Homo sapiens* | RDH8 | 50700 | 19 | SDR28C2 | 6 | 22210 | 100 | 331 | GCSSGIG | YAASK |
| *Pan paniscus* | | 100984041 | 19 | | | | 98.71 | 311 | | |
| *Mus musculus* | Rdh8 | 235033 | 9 | | | | 82.96 | 317 | | |
| *Monodelphis domestica* | RDH8 | 100013649 | 3 | | | | 68.14 | 321 | | YSASK |
| *Gallus gallus* | LOC100858293 | 100858293 | 27 | | | | 56.63 | 318 | | YAAAK |
| *Anolis carolinensis* | rdh8 | 100560987 | - | | | | 61.86 | 316 | | YAASK |
| *Xenopus tropicalis* | LOC100491569 | 100491569 | - | | | | 51.06 | 358 | | |
| *Ictalurus punctatus* | rdh8 | 108257408 | 24 | | | | 54.81 | 317 | | YTASK |

Table S24. Genetic and molecular data of the vertebrate SDR28C family variants. For further details see Table S15.

| Species | Gene symbol | Gene ID | Chr | Enzyme symbol | Exons n. | Phases formula | % identity | aa n. | Structure consensus | Catalysis consensus |
|---|---|---|---|---|---|---|---|---|---|---|
| *Homo sapiens* | DHRS7B | 25979 | 17 | SDR32C1 | 7 | 120222 | 100 | 325 | GATSGLG | YAASK |
| *Pan troglodytes* | | 454512 | 17 | | | | 99.38 | | | |
| *Mus musculus* | Dhrs7b | 216820 | 11 | | | | 78.02 | 323 | | YSASK |
| *Monodelphis domestica* | DHRS7B | 100017149 | 6 | | 8 | 0120222 | 72.14 | 363 | | YAASK |
| *Gallus gallus* | | 416499 | 14 | | 6 | 20222 | 72.49 | 309 | | |
| *Anolis carolinensis* | dhrs7b | 100561191 | - | | | | 67.96 | | | |
| *Xenopus tropicalis* | | 779695 | - | | | | 66.87 | 323 | | YSASK |
| *Danio rerio* | | 550454 | 3 | | | | 56.01 | 316 | GASSGLG | YAASK |
| *Homo sapiens* | DHRS7C | 201140 | 17 | SDR32C2 | 6 | 20222 | 100 | 312 | DAISGLG | YAASK |
| *Pan troglodytes* | | 455079 | 17 | | | | 100 | | | |
| *Mus musculus* | Dhrs7c | 68460 | 11 | | | | 91.78 | 304 | | |
| *Monodelphis domestica* | DHRS7C | 100031331 | 2 | | | | 89.39 | 332 | | |
| *Gallus gallus* | | 417304 | 18 | | | | 77.17 | 311 | | |
| *Anolis mississippiensis* | dhrs7c | 100556174 | 2 | | | | 72.99 | | DALSGVG | |
| *Xenopus tropicalis* | | 100135266 | - | | | | 77.17 | | DAISGLG | |
| *Danio rerio* | dhrs7cb | 553684 | 12 | | | | 56.27 | 324 | DAVSGMG | |

Table S25. Genetic and molecular data of the vertebrate SDR32C family variants. The asparagine (D) diagnostic amino acids of the vertebrate structure consensus are highlighted in turquoise. For further details see Table S15 and Consensuses in Online Resources 1.

| Species | Gene symbol | Gene ID | Chr | Enzyme symbol | Exons n. | Phases formula | % identity | aa n. | Structure consensus | Catalysis consensus |
|---|---|---|---|---|---|---|---|---|---|---|
| *Homo sapiens* | SDR42E1 | 93517 | 16 | | | | 100.00 | 393 | GGSGYFG | |
| *Pan troglodytes* | | 468047 | 16 | | | | 98.98 | | | |

| | | | | | | | | | | |
|---|---|---|---|---|---|---|---|---|---|---|
| *Mus Musculus* | Sdr42e1 | 74032 | 8 | SDR42E1 | 2 | 1 | 79.64 | 394 | GGGGYFG | YSRTK |
| *Sarcophilus harrisii* | SDR42E1 | 100921033 | 1 | | | | 72.12 | 396 | | |
| *Gallus gallus* | | 415806 | 11 | | | | 70.59 | 391 | | |
| *Python bivittatus* | | 103058160 | - | | | | 67.26 | | GGSGYFG | |
| *Xenopus tropicalis* | Sdr42e1 | 100496843 | 4 | | | | 59.49 | | GGGGYFG | |
| *Lepisosteus oculatus* | | 102689558 | LG23 | | | | 65.46 | 390 | | |
| | | | | | | | | | | |
| *Homo sapiens* | SDR42E2 | 100288072 | 16 | SDR42E2 | 11 | 0020200020 | 100.00 | 626 | GGGGYLG | YSRTK |
| *Pan troglodytes* | | 100615511 | 16 | | | | 97.84 | 602 | | |
| *Mus musculus* | Gm5737 | 436008 | 7 | | | | 75.41 | 608 | | |
| *Sarcophilus harrisii* | SD43E2 | 100916789 | 2 | | | | 58.76 | 540 | GGGGYFG | |
| *Gallus gallus* | | 416618 | 14 | | | | 53.59 | 454 | | |
| *Python bivittatus* | | 103065881 | - | | 14 | 0020200020211 | 51.96 | 541 | GGAGYFG | |
| *Xenopus tropicalis* | sdr42e2 | 101731274 | 9 | | 11 | 0020200020 | 52.15 | 451 | GGGGYVG | YSRSK |
| *Lepisosteus oculatus* | | 102689689 | LG13 | | 11 | 10020200020 | 55.70 | 470 | GGAGYFG | YSKTK |

Table S26a. Genetic and molecular data of the vertebrate SDR42E family variants. For further details see Table S15.

| Variants | Species | % Identity | Variants | Species | % Identity |
|---|---|---|---|---|---|
| SDR42E1 | *H. sapiens* | 100.00 | SDR42E2 | *H. sapiens* | 100.00 |
| | *P. troglodytes* | 98.98 | | *P. troglodytes* | 97.84 |
| | *M. musculus* | 79.64 | | *M. musculus* | 75.41 |
| | *S. harrisii* | 72.12 | | *S. harrisii* | 58.76 |
| | *G. gallus* | 70.59 | | *G. gallus* | 53.39 |
| | *P. bivittatus* | 67.26 | | *P. bivittatus* | 51.84 |
| | *X. tropicalis* | 59.49 | | *X. tropicalis* | 53.21 |
| | *L. oculatus* | 65.46 | | *L. oculatus* | 55.70 |
| SDR42E2 | *H. sapiens* | 48.81 | SDR42E1 | *H. sapiens* | 48.81 |
| | *P. troglodytes* | 48.81 | | *P. troglodytes* | 48.81 |
| | *M. musculus* | 48.01 | | *M. musculus* | 46.42 |
| | *S. harrisii* | 48.42 | | *S. harrisii* | 45.93 |
| | *G. gallus* | 47.35 | | *G. gallus* | 46.15 |
| | *P. bivittatus* | 46.05 | | *P. bivittatus* | 45.89 |

| | | | | | |
|---|---|---|---|---|---|
| | *X. tropicalis* | 45.91 | | *X. tropicalis* | 45.21 |
| | *L. oculatus* | 46.77 | | *L. oculatus* | 46.03 |

Table S26b. Percent identity of the vertebrate SDR42E1 and SDR42E2 variants.
