# Supplementary material for: Gene Structure Evolution of the Short-Chain Dehydrogenase/Reductase (SDR) Family
Source: Genes (Basel). 2022 Dec 30;14(1):110. doi: 10.3390/genes14010110 (PMC9859523; doi:10.3390/genes14010110)
Supplement: Supplementary file 1 [file genes-14-00110-s001.zip › Online Resources 4/Online Resources 4.pdf]

INVERTEBRATE ORTHOLOGS OF THE HUMAN SDR FAMILY VARIANTS

```
SDR7C1   H. sapiens       ---------------------------------------------MV-EL--MFPLL--L
SDR7C2   H. sapiens       ----------------------------------------------------MLVTLGLL
SDR7C3   H. sapiens       -----------------------------------------------------MSRYLLP
SDR7C4   H. sapiens       --------------------------------MAVATA-----AAVL-A----ALGGALW
SDR7C5   H. sapiens       -------------------------------------------ME------ALLLGAGLL
SDR40C1  H. sapiens       ---------------------------------------------------MSLYRSVVW
SDR7-1C1 C. intestinalis  MSTFVANPPSSHIFESLVPYYAEALLGAQNN----NTAGE----EISHPNG-HSVTNGVW
SDR7-1C2 C. intestinalis  ------------------------------------------------------------
SDR7-3C2 C. intestinalis  ---------------------------------------------------MSIYQKTAF
SDR7-3C3 C. intestinalis  ---------------------------------------------------MSRFRVIVW
SDR7-3C4 C. intestinalis  ----------------------------------------------------MQARYRLL
SDR7-1C1 S. purpuratus    ------------------------------------------------------------
SDR7-1C3 S. purpuratus    -------------------------------------------MDIVNRI--FHPRYTLP
SDR7-2C2 S. purpuratus    ------------------------MISLSTLS-----------LERVPDFGQRGPGDLEW
SDR7-1C3 M. domestica     -------------------------------------------MEGLFSF--LRSRPVFW
SDR7-1C1 B. malayi        ------------------------MIEFSESSMTSDE--------GY--FG------SLS
SDR7-1C2 A. californica   ------MRPEQHAKM--AA-EMKQRLKVEELEENIDELEDV--VEDVFPI--LIL--TVF
SDR7-1C3 A. californica   -------------------------MTLQEYSLPVSVVGTLVALAVFFnf--LDF----V  1p.1

                                                                 ×   × ×
SDR7C1   H. sapiens       LLLPFL-LY-MAAPQIrkMLSSGVCTSTVQLPGKVVVVTGANTGIGKETAKELAQrgARV  1p.1        2p.2
SDR7C2   H. sapiens       TSFFSF-LY-MVAPSIrkFFAGGVCRTNVQLPGKVVVITGANTGIGKETARELASrgARV  1p.1        2p.2
SDR7C3   H. sapiens       LSALGT-VA-GAAVLLkdYVTGGACPSKATIPGKTVIVTGANTGIGKQTALELARrgGNI  1p.1        2p.2
SDR7C4   H. sapiens       LAARRF-----VGPRVQRLRRG---GDPGLMHGKTVLITGANSGLGRATAAELLRLGARV
SDR7C5   H. sapiens       LG--------AYVLVYYNLVKAPPCGGMGNLRGRTAVVtgANSGIGKMTALELARRGARV        1p.2
SDR40C1  H. sapiens       FAKGLR--EYTksGYESACKDFVPHDLEVQIPGRVFLVTGGNSGIGKATALEIAKrgGTV  1p.1        2p.2
SDR7-1C1 C. intestinalis  VLLVGTLLW--SYILLVILAKKPKCDCTARLDGKTVVItgATDGVGKATAFELASRGAEI        1p.2
SDR7-1C2 C. intestinalis  ----------------MKSVRNGWVRSDISMVGKNIIITGANAGIGFITTRDLVKRGGRV
SDR7-3C2 C. intestinalis  FIKGRL--QFTngGYAKAAKSFHPEDTKKDVSDQVFMVTGANSGLGKAAAIAIAKNGGEV  1p.1
SDR7-3C3 C. intestinalis  SSLICA----VAYLWIVFIKRGPICVSNKRLDGKTVLItgGNTGVGEATVFEVAKLGARI        1p.2
SDR7-3C4 C. intestinalis  SGFVGS-LS-----KMVKSARGSWVRSDVKMTGKTVVITGANTGIGLETAIDLVKREARV
SDR7-1C1 S. purpuratus    ------------------------------MDGKTVIITGCNTGIGKETAKDLAKrgARV              1p.2
SDR7-1C3 S. purpuratus    FTTFGA-LV-GGTLLLkdYYGGDKCPSTARVDGKTVIITGANSGIGKETALELAKrgGKI  1p.1        2p.2
SDR7-2C2 S. purpuratus    TTIALMMVVLVVGLYYYVVKVADRCSCPKRLDGKTVIVtgANTGIGKETARDLARRGARV        1p.2
SDR7-1C3 M. domestica     TSVAGT-TVGVAC-FVkdMMQGGKFTKPTRADGKVIIVTGSNTGIGKETVRELAKRGATV  1p.1
SDR7-1C1 B. malayi        VPLFGV-LTCAGIYFlrRYLKGAQFNEYVEAGGKIALVTGASAGIGKQTARELNLRGATV  1p.2
SDR7-1C2 A. californica   VAVLLL-IIFLIRLYLR---WFAPNPSKNRMDGKTVLItgGTSGIGKCTAIELAKRNARV        1p.2
SDR7-1C3 A. californica   IGLLLI-LVPVSCILLKEYMSGGRYYGKERIDGKTVVITGANCGIGKETARDLAGrgGRI              2p.2
                                                                **   * *

SDR7C1   H. sapiens       YLACRDVEKGELVAKEIQTT-------------TGNQQVLVRKLDLSDTKSIRAFAKGFL
SDR7C2   H. sapiens       YIACRDVLKGESAASEIRVD-------------TKNSQVLVRKLDLSDTKSIRAFAEGFL
SDR7C3   H. sapiens       ILACRDMEKCEAAAKDIRGE-------------TLNHHVNARHLDLASLKSIREFAAKII
SDR7C4   H. sapiens       IMGCRDRARAEEAAGQLRRELRQAAECGPEPGVSGVGELIVRELDLASLRSVRAFCQEML
SDR7C5   H. sapiens       VLACRSQERGEAAAFDLRqe-------------SGNNEVIFMALDLASLASVRAFATAFL  2p.0
SDR40C1  H. sapiens       HLVCRDQAPAEDARGEIIRE-------------SGNqnIFLHIVDLSDPKQIWKFVENFK        3p.0
SDR7-1C1 C. intestinalis  VIPCRNAEkgRRVAREIADI-------------TGNHKIQVVYVDFTNLESVKVCTQILK  2p.2
SDR7-1C2 C. intestinalis  ILACRNMELALAAKDtiLKETG-----------KEEKYVVVKKLDLSSLQSIRDFAHDIN  1p.0
SDR7-3C2 C. intestinalis  HMVCRNLERANQAKEDIVKE-------------SGNsnITVHVLDISNTKEVYEFAKNFS        2p.0
SDR7-3C3 C. intestinalis  VIASRNKTKSEDVKIRVVKA-------------TGNQniRVMELDLGDFDSVRKFVKNFN  2p.0
SDR7-3C4 C. intestinalis  ILGCRNMEKAEEAKQriFKEAG-----------GKDDTVVIKQLDLSSLASVRAFAKDIN  1p.0
SDR7-1C1 S. purpuratus    IMACRNVEKAKEAQLDVIKE-------------SGSSNVVVKKLDLASMKSIREFVEELK
SDR7-1C3 S. purpuratus    ILACRDIKKAEKAREEIVAA-------------SGNTDVKVLQLDLASLSSIRQFADKIS
SDR7-2C2 S. purpuratus    ILACRDAVRGREAEKDIRMS-------------TGNDDVIFMKLNLASFDSIRHFAQEFN
SDR7-1C3 M. domestica     YMACRDMKKCEeaREEIVLE-------------TKNKYVYCRECDLASLESIRQFAEafK  2p.0  3p.1
```

```
SDR7-1C1 B. malayi        YMLCRDRAKSENARIELTkl-G-----------CNPTRLILKDVDLASFATIRKFADEIR  2p.0
SDR7-1C2 A. californica   LIAGREKAKVEAVARNIrkK-------------TGNQHVNALVLDLGNLRSIQEFAEDFR  2p.1
SDR7-1C3 A. californica   IMACRDLEKCEQAKKEIVEE-------------TGNKNIECQKLDLASFASIRAFAKLIN
                              *

SDR7C1   H. sapiens       aeEKHLHVLINNAGVMMCPYS-KTAD-GFEMHIGVNHlgHFLLTHLLLEKLKE---SAPS  3p.2      4p.2
SDR7C2   H. sapiens       aeEKQLHILINNAGVMMCPYS-KTAD-GFETHLGVNHlgHFLLTYLLLERLKV---SAPA  3p.2      4p.2
SDR7C3   H. sapiens       eeEERVDILINNAGVMRCPHW-TTED-GFEMQFGVNHlgHFLLTNLLLDKLKA---SAPS  3p.2      4p.2
SDR7C4   H. sapiens       qeEPRLDVLINNAGIFQCPYM-KTED-GFEMQFGVNHLGHFLLTNLLLGLLKS---SAPS  1p.0
SDR7C5   H. sapiens       SSEPRLDILIHNagISSCGR---TRE-AFNLLLRVNHIGPFLLTHLLLPCLKA---CAPS       3p.2
SDR40C1  H. sapiens       QEH-KLHVliNNAGCMVNKRE-LTED-GLEKNFAANTlgVYILTTGLIPVLEK---EHDP  4p.0      5p.2
SDR7-1C1 C. intestinalis  KSLKKVDILINNagIGNGVG--TTLD-GFPLVFGVNHLAPFLFTTELLPLLrk---APTA       3p.2 4p.1
SDR7-1C2 C. intestinalis  QTERRIDVLINNagVMLCPET-KTKD-GFESHFGVNHLGHFLLTNLLLDLLkh---SAPS       2p.2 3p.0
SDR7-3C2 C. intestinalis  SNHEKLNVLVNNAGCMVNDRK-TTEVGNLELNFATNTVGTYVLTQELVPLLLK---STKP
SDR7-3C3 C. intestinalis  ETEEHLDFLINNagLLGIYG--QTKQ-GINKLYAVNHFGPFLLTNLLLPKMKEQSKSRPV       3p.2
SDR7-3C4 C. intestinalis  DNESKIDVLLNNagIMLVPKG-KTED-GFELHYGVNHLGHFLLTNLLLDLIkr---SAPS       2p.2 3p.0
SDR7-1C1 S. purpuratus    KEEKSLDVLVNNagVMMCPRW-ETED-GFEMQFGTNHLGHFLLTLLLLDLIKA---SAPS       2p.2
SDR7-1C3 S. purpuratus    sdEEGVDVLVNNAGLMRCPKW-KTED-GFEMQFGVNHlgHFYLTNLLLDKLKA---SAPS  3p.2      4p.2
SDR7-2C2 S. purpuratus    NTEERLDILVNNagVINDGSL-RTEE-GHELVFGVNHLGHFLLTNILLDKLQK---CAPS       2p.2
SDR7-1C3 M. domestica     AEQKRLDILINNAGVMRCPRS-LTKE-GFELQLGVNHMGHFLLTNLLLDELkk---SAPS            4p.0
SDR7-1C1 B. malayi        yeVDKIDILVNNAGIMFYPKFELTED-GHEMTWQTNYlgHFLLTELLLPLIKK---SSNG  3p.2      4p.2
SDR7-1C2 A. californica   KDEKYLHVLINNAAym-GPLS-KTDD-GLERSFGVNYLGHFYLTYLLSDklKK---NAPS  3p.2      4p.2
SDR7-1C3 A. californica   stESHVDILINNAGVMMCPKM-LTED-GLEMQIGVNHfgHFLLTQLLMDKLKA---SAPS  3p.2      4p.2
                                  *  **          *           *       *  *

                                                              +   +
SDR7C1   H. sapiens       RIVNVSSLAHHLGR--IHFHNLQGE--KFY-NAGLAYCHSKLANILFTQELARRLkgSGV       5p.2
SDR7C2   H. sapiens       RVVNVSSVAHHIGK--IPFHDLQSE--KRY-SRGFAYCHSKLANVLFTRELAKRLqgTGV       5p.2
SDR7C3   H. sapiens       RIINLSSLAHVAGH--IDFDDLNWQT-RKY-NTKAAYCQSKLAIVLFTKELSRRLqgSGV       5p.2
SDR7C4   H. sapiens       RIVVVSSKLYKYGD--INFDDLNSE--QSY-NKSFCYSRSKLANILFTRELARRLEGTNV
SDR7C5   H. sapiens       RVVVVASAAHCRGR--LDFKRLDRPV-VGWRQELRAYADTKLANVLFARELANQLEATGV
SDR40C1  H. sapiens       RviTVSSGGMLVQK--LNTNDLQSE--RTPFDGTMVYAQNkrQQVVLTERWAQGHP--AI  6p.0 7p.0
SDR7-1C1 C. intestinalis  RIVCVSSVTHLWGN--INFDELQDLDGGSLPSQFRAYSRSKLANVLFAKQLHRNESQHGI
SDR7-1C2 C. intestinalis  RVVIVASEAHRIGKTYIQWSDMNSG--EGM-D---TYCRSKLMNILFARELSDRLkgSGV       4p.2
SDR7-3C2 C. intestinalis  RviTVSSGGMYTQK--LNVKDLQSE--KGTFSGDMAYAQQKRQqvILTEEWAKMHP--DI  3p.0
SDR7-3C3 C. intestinalis  RIINLSSSAHraSP--LDFSKIYEPA-EGVLENFRVYSNTKLATIYFTTELLKRIEGFDI  4p.0
SDR7-3C4 C. intestinalis  RIVTVSSEAHRLGTPKIDFKDMNFD--NNY-DESVAYGRSKLMNILFTKELSKRLegTNV       4p.2
SDR7-1C1 S. purpuratus    RIVNVSSLAHQFGK--INFDDIMSK--EKY-SDMDAYGQSKLANVLFTRELATRLkgSGV       3p.2
SDR7-1C3 S. purpuratus    RIINVSSVAHQVGK--INFEDINSD--QRY-NSAEAYANSKLAKVLFTRELSKRLegTGV       5p.2
SDR7-2C2 S. purpuratus    RVINVSSDAYMFGK--LDLERLSVN----D-GRVKSYARSKLANVLFTRQLADKMAGTGV
SDR7-1C3 M. domestica     RIVVLSSMAHTRGE--INVGDLNSE--KSY-DEGKAYNQSKLANILFTRELARRLegTGV       5p.2
SDR7-1C1 B. malayi        RIINVSSSLHKTADS-VDVSIVNN---KKYFSKSMPYSRSKLAqvMHVRELTRRLRTKDP  5p.0
SDR7-1C2 A. californica   RIINVVSDSYKQGE--LDLDDLAMN--KGY-SVYSAYTRSKLAQVMFNLECHKRYIGNCV
SDR7-1C3 A. californica   RIIFVSSLGHTLGK--MNFEDLNSE--KSY-SKLNAYGQSKLANILCSREMARRLegTGV       5p.2
                          *     *                             *   *

SDR7C1   H. sapiens       ----TTYSVHPGTVQSELV-RHSSF----------MRWMWWLFSFFIKTPQQGAQTSLHC
SDR7C2   H. sapiens       ----TTYAVHPGVVRSELV-RHSSL----------LCLLWRLFSPFVKTAREGAQTSLHC
SDR7C3   H. sapiens       ----TVNALHPGVARTELG-RHTGIHGSTF----SSTTlgPIFWLLVKSPELAAQPSTYL       6p.2
SDR7C4   H. sapiens       ----TVNVLHPGIVRTNLG-RHIHI--PLL----VKPLFNLVSWAFFKTPVEGAQTSIYL
SDR7C5   H. sapiens       ----TCYAAHpgPVNSELFLRHVPGWLRPL--------LRPLAWLVLRAPRGGAQTPLYC  4p.2
SDR40C1  H. sapiens       ----HFSSMHPGWADTpgVRQAMPGFHAR-------------FGDRLRSEAQGADTMLWL       8p.2
SDR7-1C1 C. intestinalis  ----STYIAHpgWTFTAIF-RDSPVLAKL--------LLFPLLYLFLYTPAQGAQTQIHL  5p.2
SDR7-1C2 C. intestinalis  ----TVNSLHPGVIRSGLW-QHLHDEELSIWRWVLHKTMNPFMKMFWKSPEYGAQTTIYC
SDR7-3C2 C. intestinalis  ----HFSAMHPGWADTPAVRTSMPGFYEY-------------MKDNLRTAEqgADTIVWL       5p.2
SDR7-3C3 C. intestinalis  ----STYAVHpgYISSELF-ENFPVPFNYI--------GRIPLILFSksTYYGCQTTMYC  5p.2 6p.1
SDR7-3C4 C. intestinalis  ----TANCLHPGVIKSELW-RHMDGSRKPVRDFF----VGTFVRWFGKTIIHGAQTNIYC
```

```
SDR7-1C1 S. purpuratus    ----TSYAVHPGGVDTDLA-RHRDS--YPF----YLRVLLPLMVPFIKTSEEGAQTNIYC
SDR7-1C3 S. purpuratus    ----TANVLHPGVVKTNIG-RHTGMHQSGF----SMAILGPIFWLFVRSPQQGAQTSVYC
SDR7-2C2 S. purpuratus    ----VSFSLHpgSVNTEIK-RNWAGWLRAL--------APLISFFFLKSVKAGAQTSIHC  3p.2
SDR7-1C3 M. domestica     ----TVNALHPGVVDTELF-RHMGFFNSFF----ASLIFKPLSWPFLKSPKNGAQTTLYA
SDR7-1C1 B. malayi        GTTVTINAVHPGVCFTELM-RYTVFSRKY-----ILKIISPLLWFFMKTDkdGAQTTLYV       6p.0
SDR7-1C2 A. californica   ----WSFAAHpgPTATDLLRNYPGMWGNA---------LRLVSRIMFKPPEDGCQTLVYL  5p.2
SDR7-1C3 A. californica   ----TVNSLHPGTVSTELT-RHLSFWNSSI----FKVFAGPFQYILLKTPMQGAQTSITL
                                   ***

SDR7C1   H. sapiens       ALTEG-LEILSGNHFsdCHVAWVSAQ-------A-RNETIARRLWDVSCDLLGLPID---  6p.1
SDR7C2   H. sapiens       ALAEG-LEPLSGKYFsdCKRTWVSPR-------A-RNNKTAERLWNVSCELLGIRWE---  6p.1
SDR7C3   H. sapiens       AVAEE-LADVSGKYFDGLKQKAPAPE-------A-EDEEVARRLWAESARLVGLEAPSVR
SDR7C4   H. sapiens       ASSPE-VEGVSGRYFGDCKEEELLPK-------A-MDESVARKLWDISEVMVGLLK----
SDR7C5   H. sapiens       ALQEG-IEPLSGRYFANCHVEEVPPA-------A-RDDRAAHRLWEASKRLAGLGPGEDA
SDR40C1  H. sapiens       ALSSAAAAQPSGRFFqdRKPVSTHLPLATASSSPAEEEKLIEILEQLAQTFK--------  9p.2
SDR7-1C1 C. intestinalis ALAPG-IESSSGNYFVSCKPARTGKS-------A-KDIELASNLWSYSEKAVASALNQSV
SDR7-1C2 C. intestinalis SVAPE-LLNVTGKYFsdCAIAYESGE-------A-KSKRNAVKLWNISCELTGINEFITF  5p.1
SDR7-3C2 C. intestinalis SVSDAALAQPSGLFFldRKAVPTHLALAWTRESAEDRNTFLTKIAEIANQFKP-------  6p.2
SDR7-3C3 C. intestinalis MLDDM-VTEFSGHYFASCRKTEPQSF-------A-HNQTAAQKLWQLSLEITEFSEQ---
SDR7-3C4 C. intestinalis CMAPE-IEDVTGKYFsdCAVASENSQ-------A-KKDKNAEQLWQVSLQATGLSEA---  5p.1
SDR7-1C1 S. purpuratus    SVDEK-AGQETGLYYsdCAVKQAAKQ-------G-RDDEAARKLWDLSLKLVGLEE----  4p.1
SDR7-1C3 S. purpuratus    AVDPE-LEKVSGQYFrdCKKSECDAS-------G-KDDEVAAKLWDVSCQLTGLSSEETK  6p.1
SDR7-2C2 S. purpuratus    AVSDD-ILDQSGEFFKGCQVQKLSRT-------A-QDQDLAQRLWDVSLEMTGLQSS---
SDR7-1C3 M. domestica     ALDED-LATVSGKYFSDCNIKDVAPQ-------A-KDDALAKWLWAVSEKWTRLTK----
SDR7-1C1 B. malayi        ALSKN-VEAISGRYFgeCKEHTPSPN-------S-LDDTKCNILYNQSLEAVKLSE----  7p.1
SDR7-1C2 A. californica   AVADG-LREFsgKLFTDCKAVKTNEK-------VKANKELSSRLWNVSAHLCGFEGDALM  6p.2
SDR7-1C3 A. californica   AVDPR-LEKVTGKYFsdCWEKRTARQ-------A-EDDGAAKKLWEISEEWTRLKQD---  6p.1
                                     *

SDR7C1   H. sapiens       ----------------------------------------------------------
SDR7C2   H. sapiens       ----------------------------------------------------------
SDR7C3   H. sapiens       EQPLPR----------------------------------------------------
SDR7C4   H. sapiens       ----------------------------------------------------------
SDR7C5   H. sapiens       EPDEDPQSEDSEAPSSLSTPHPEEPTVSQPYPSPQSSPDLSKMTHRIQAKVEPEIQLS
SDR40C1  H. sapiens       ----------------------------------------------------------
SDR7-1C1 C. intestinalis GYCTSI----------------------------------------------------
SDR7-1C2 C. intestinalis ----PTHRL-------------------------------------------------
SDR7-3C2 C. intestinalis ----------------------------------------------------------
SDR7-3C3 C. intestinalis ----------------------------------------------------------
SDR7-3C4 C. intestinalis ----------------------------------------------------------
SDR7-1C1 S. purpuratus    ----------------------------------------------------------
SDR7-1C3 S. purpuratus    EKSAKTHDVVKEAER-------------------------------------------
SDR7-2C2 S. purpuratus    ----------------------------------------------------------
SDR7-1C3 M. domestica     ----------------------------------------------------------
SDR7-1C1 B. malayi        ----------------------------------------------------------
SDR7-1C2 A. californica   PEDEPEATPNAEEAGEATA---EEEKKSQ-----------------------------
SDR7-1C3 A. californica   ----------------------------------------------------------
```

Figure S25. Alignment of the invertebrate orthologs of the human SDR7C family and of the human SDR40C1 variants. × and + symbols mark the structure consensus and the catalysis consensus respectively; the couples of amino acid symbols in red lowercase mark the splicing-site positions; * symbol marks the position of identical amino acid residues of the aligned SDR protein sequences. The splicing sites are progressively numbered and the phase (p.) type is indicated after the splicing-site number; the phase symbols, highlighted in a same color, belong to orthologous splicing sites.

```
SDR9C1   H. sapiens       ----------------------------------------MLATRL-------SRPLSRLP
SDR9C2   H. sapiens       ----------------MSTFF-SDTAWICLAVPTVLCGTVFCKYKKSSGQLWSWMVCLAG
SDR9C3   H. sapiens       ----------ME----RWPWP-SGGAWLLVAARALLQL--LRSDLRLGRPLLAALALLAA
SDR9C4   H. sapiens       ------------------------------------------------------------
SDR9C5   H. sapiens       ------------------------------------------------------------
SDR9C6   H. sapiens       ------------------------------------------------------------
SDR9C7   H. sapiens       ------------------------------------------------------------
SDR9C8   H. sapiens       ------------------------------------------------------------
SDR9-1C2 C. intestinalis  ------------------------------------------------------------
SDR9-1C2 S. purpuratus    ----------------------------------------------------------MH
SDR9-1C2 C. elegans       ------------------------------------------------------------
SDR9-1C4 C. elegans       ------------------------------------------------------------
SDR9-1C2 A. californica   MEIDIQNELNRDGIVFYSVLYSSVTVIFAFAVLS----KFLTNEFRFGFRSFLSLVTLFL
SDR9-1C5 A. californica   ------------------------------------------------------------

                                                                              ×     ×
SDR9C1   H. sapiens       GKTLSACDRENGArrP-LLLGST----SFIPIGRRTYASAAEpvGSKAVLVTGCDS--GF  1p.1 2p.0
SDR9C2   H. sapiens       LCAVCLLILSPFWGLILFSVSCFLMY-------TYLSGQELLPVDQKAVLVtgGDC--GL  1p.2
SDR9C3   H. sapiens       LDWLCQRLLPPPAALAVLAAAGWIAL-------SRLARPQRLPVATRAVLItgCDS--GF  1p.2
SDR9C4   H. sapiens       -----------MLFWVL---GLLILC-GFLW--TRKGKLKIEDITDKYIFITGCDS--GF
SDR9C5   H. sapiens       -------------MWLPLLLGA-LLW-AVLW--LLRDR-QSLPASNAFVFITGCDS--GF
SDR9C6   H. sapiens       -------------MWLYLAAFVGLYY-LLHW--YRERQ-VVSHLQDKYVFITGCDS--GF
SDR9C7   H. sapiens       -----------------MAALTDLSF-MYRW--FKNCN-LVGNLSEKYVFITGCDS--GF
SDR9C8   H. sapiens       -------------MWLYLAVFVGLYY-LLHW--YRERQ-VLSHLRDKYVFITGCDS--GF
SDR9-1C2 C. intestinalis  ----------------------------------------MAKSHQKNVVItgCDIPNSY  1p.2
SDR9-1C2 S. purpuratus    EAPlaKCIMSFLLTLVGLTVGVIVAR-MIEY--LIRRPHIDNITKDkyVFITGCDT--GF  1p.2 2p.1
SDR9-1C2 C. elegans       ---MVVCCLCTLFTWVLYFVAVVVVWNIVry--ILELIPEGGSLQDRAILITGCDT--GF  1p.1
SDR9-1C4 C. elegans       -----MLSASTIFAWVAIFGSYV-AYkvYNF--LI--KQRVDNYGKKTVAISGCDS--GF  1p.2
SDR9-1C2 A. californica   GEPLCQFLVEGPSGLVVFSLGCLIIY-------Y-ILPASHLPIKNRSVLItgCDS--GF  1p.2
SDR9-1C5 A. californica   -------------MWLMIAAIVTGIV-GYLFINRFLRSLDVGDYGNKYVFITGCDT--GF
                                                                              * *

                          ×
SDR9C1   H. sapiens       GFSLAKHLHSKGFLVFAGCLMkdKGHDGVKELDSL-NSDRLRTV-QLNVCSSEEVEKVVE  3p.0
SDR9C2   H. sapiens       GHALCKYLDELGFTVFAGVLNENGPG--AEELRRTC-SPRLSVL-QMDITKPVQIKDAYS
SDR9C3   H. sapiens       GKETAKKLDSMGFTVLATVLELNSPG--AIELRTCC-SPRLRLL-QMDLTKPGDISRVLE
SDR9C4   H. sapiens       GNLAARTFDKKGFHVIAACLTESGST-A---LKAET-SERLRTV-LLDVTDPENVKRTAQ
SDR9C5   H. sapiens       GRLLALQLDQRGFRVLASCLTPSGAE-D---LQRVA-SSRLHTT-LLDITDPQSVQQAAK
SDR9C6   H. sapiens       GNLLARQLDARGLRVLAACLTEKGAE-Q---LRGQT-SDRLETV-TLDVTKMESIAAATQ
SDR9C7   H. sapiens       GNLLAKQLVDRGMQVLAACFTEEGSQ-K---LQRDT-SYRLQTT-LLDVTKSESIKAAAQ
SDR9C8   H. sapiens       GKLLARQLDARGLRVLAACLTEKGAE-Q---LRGQT-SDRLETV-TLDVTKTESVAAAAQ
SDR9-1C2 C. intestinalis  GYELALCLDHQGYVVFAGCTDCNSVG--ARSLASRG-STRLAVM-QIDVTKQDEIDDATE
SDR9-1C2 S. purpuratus    GNKLAKQLDIMGVHVIAACLTEKGRT-D---LDDVT-SDRVKTL-ILDVTDHESVLRAYE
SDR9-1C2 C. elegans       GRELAKKCAKNGFLVFAGCLTTeaAK-T---LESESANPRLRTV-PLDVSKDESVEKTVE  2p.0
SDR9-1C4 C. elegans       GRLLAVRLVKQGVPVIAGVLKKenGE-S---LKKEVNTPSLLTFGILNVANDESVKEFVK  2p.0
SDR9-1C2 A. californica   GHALTIKLDSLGIQVFAGCLEDRGPG-AL-ELKNRC-SDrlHIL-KLDVTNSKDIAEASN  2p.1
SDR9-1C5 A. californica   GHRLSirLNKMGFHVISGCLTQKGAD-T---LTQLC-PDRLSAL-LLNVADSDSIQKAYK  1p.1
                          *          *  *                *                           .

SDR9C1   H. sapiens       IVRSSLKDPEkgMWGLVNNAGISTF-GEVEFTSLETYKQVAEVNLWGTVRMTKSFLPLIR  4p.2
SDR9C2   H. sapiens       KVAAML--QDrgLWAVINNAGVLGFPTDGELLLMTDYKQCMAVNFFGTVEVTKTFLPLLR  2p.2
SDR9C3   H. sapiens       FTKAHT--TStgLWGLVNNAGHNEVVADAELSPVATFRSCMEVNFFGALELTKGLLPLLR  2p.2
SDR9C4   H. sapiens       WVKNQV--GEkgLWGLINNAGVPGVLAPTDWLTLEDYREPIEVNLFGLISVTLNMLPLVK  1p.2
SDR9C5   H. sapiens       WVEMHV--KEagLFGLVNNAGVAGIIGPTPWLTRDDFQRVLNVNTMGPIGVTLALLPLLQ  1p.2
SDR9C6   H. sapiens       WVKEHV--GDrgLWGLVNNAGILTPITLCEWLNTEDSMNMLKVNLIGVIQVTLSMLPLVR  1p.2
SDR9C7   H. sapiens       WVRDKV--GEqgLWALVNNAGVGLPSGPNEWLTKDDFVKVINVNLVGLIEVTLHMLPMVK  1p.2
SDR9C8   H. sapiens       WVKECV--RDkgLWGLVNNAGISLPTAPNELLTKQDFVTILDVNLLGVIDVTLSLLPLVR  1p.2
SDR9-1C2 C. intestinalis  MIQDMI--GDegLWAVVNCAEFYTV-AEADWCSVEDYEQSMQVNYLGTVRVTKSLLHLIR  2p.2
```

```
SDR9-1C2 S. purpuratus    EVKKIIP-HKaaLWGVVNNAGISNLAGYLEWTRREDYEKVLAVNLFGVVDVTLTFLPLLK  3p.2
SDR9-1C2 C. elegans       FVKKSL--GNHKLWGVVNNAGIFSCYGPDDWCRMNDYKLALDVNCLGVIRVTQAFKKLVK
SDR9-1C4 C. elegans       LVEKTV--ENRGLWGVVANAGILGNSGPDDWLSAQDYIntMQVNTFGVMRFIQGLKKFVK  3p.0
SDR9-1C2 A. californica   YIKSRV--GEegLWGLVNNAGVWCF-AELGMTSETIVQKVMDVNLFGAVRMTRAVLPLIK  2p.2
SDR9-1C5 A. californica   HVQDLLP-AGkgLWAVVNNAGISGNTSRIEMCTKQDFVDAIDINLFGPIEVSRTFLPLLR  2p.2
                                           *                           *  *

                                                  +   +
SDR9C1   H. sapiens       RAkgRVVNISSMLGRMANPARSPYCITKFGVEAFSDCLRYEMYPLGV--KVSVVEPGNFI  5p.2
SDR9C2   H. sapiens       KSKGRLVNVSSMggGAPMERLASYGSSKAAVTMFSSVMRLELSKWGI--KVASIQPGGFL  3p.2
SDR9C3   H. sapiens       SSRGRIVTVGSPagDMPYPCLGAYGTSKAAVALLMDTFSCELLPWGV--KVSIIQPGCFK  3p.2
SDR9C4   H. sapiens       KAQGRVINVSSVGGRLAI-VGGGYTPSKYAVEGFNDSLrrDMKAFGV--HVSCIEPGLFK          2p.1
SDR9C5   H. sapiens       QARGRVINITSVLGRLAA-NGGGYCVSKFGLEAFSDSLrrDVAHFGI--RVSIVEPGFFR          2p.1
SDR9C6   H. sapiens       RARGRIVNVSSILGRVAF-FVGGYCVSKYGVEAFSDILrrEIQHFGV--KISIVEPGYFR          2p.1
SDR9C7   H. sapiens       RARGRVVNMSSSGGRVAV-IGGGYCVSKFGVEAFSDSIrrELYYFGV--KVCIIEPGNYR          2p.1
SDR9C8   H. sapiens       RARGRVVNVSSVMGRVSL-FGGGYCISKYGVEAFSDSLrrELSYFGV--KVAMIEPGYFK     2p.1
SDR9-1C2 C. intestinalis  RGRGRIVNLSSMagCLARAGQSAYSASKFALEAFTDSLRQEMLKWGV--YVTLVEPAYFp  3p.2 4p.2
SDR9-1C2 S. purpuratus    ESSGRLVNVASSAGRVSA-LPGGYCESKFAVEAFSDAVrrERKHFGYLFKVSIIEPGFFA     4p.1
SDR9-1C2 C. elegans       AAKGRIVTVTSVNGRLSTPAAGPYVVSKFGAAAYMDCIrqELYNFGV--KVSILEPGIFR     3p.1
SDR9-1C4 C. elegans       KQEGrvVIISSISGRTPRPTVGPYCVSKHAVEAYADVIrhELIDFNV--SVHLLEPGFFT  4p.1 5p.1
SDR9-1C2 A. californica   QAQGRIVNVSSLlgRISVEGCGAYSMSKHALVAFTNTLRMEMKKWGV--CVSLVEPTGYF  4p.2
SDR9-1C5 A. californica   QTRGRVVCITSILGRYPS-GPAPYCTSKCGLEAFCDVLrrEVYLQGI--KVSILEPGVFR     3p.1
                              **      *  *         *   *                           *

SDR9C2   H. sapiens       --tnIAGTSDKWEKLEKDILDHLPAEVQEDYGQDYILAQRNFLLL-INSL----------  4p.2
SDR9C3   H. sapiens       --teSVRNVGQWEKRKQLLLANLPQELLQAYGKDYIEHLHGQFLH-SLRL----------  4p.2
SDR9C1   H. sapiens       AATSLYS-PESIQAIAKKMWEELPEVVRKDYGKKYFDEKIAKMET-YCSS----------
SDR9C4   H. sapiens       --TNLAD-PVKVIEKKLAIWEQLSPDIKQQYGEGYIEksLDKLKG-NKSY----------          3p.2
SDR9C5   H. sapiens       --TPVTN-LESLEKTLQACWARLPPATQAHYGGAFLTkyLKMQQRIMNLI----------          3p.2
SDR9C6   H. sapiens       --TGMTN-MTQSLERMKQSWKEAPKHIKETYGQQYFDalYNIMKE-GLLN----------          3p.2
SDR9C7   H. sapiens       --TAILG-KENLESRMRKLWERLPQETRDSYGEDYFRiyTDKLKN-IMQV----------          3p.2
SDR9C8   H. sapiens       --TAVTS-KERFLKSFLEIWDRSSPEVKEAYGEKFVAdyKKSAEQ-MEQK----------     3p.2
SDR9-1C2 C. intestinalis  eGSLVLS-----EPQTSVRLKRVPDHVKEVYGEDYFVDFINSVSRNDPTTQEEGKSKSTE
SDR9-1C2 S. purpuratus    --TPLAS-KDNLSRSVTAAWERLSDVQKKEFPESMLHefVDEKSSLISSM----------     5p.2
SDR9-1C2 C. elegans       --TPLLD-EQAMLKRVDHVWTQIDDETRTEYGETFKNyfAKMWNKTYISM----------     4p.2
SDR9-1C4 C. elegans       --TNITQ---TATNDLDAVWERLDDETKKEYGKEFYDdyKNSRF-SRLSH----------     6p.2
SDR9-1C2 A. californica   --tgVMQ-EHSLQRKKEELWSALDEETRqiYGKEYFDSAYAHILT-HAQS----------  5p.2 6p.2
SDR9-1C5 A. californica   --TNIMD-FESLKSKCESSFDQASDEVKTAYGPDYVKnyVTLVSE-TMKG----------     4p.2

SDR9C1   H. sapiens       ------------------------------------------------------------
SDR9C2   H. sapiens       ------------------------------------------------------------
SDR9C3   H. sapiens       ------------------------------------------------------------
SDR9C4   H. sapiens       ------------------------------------------------------------
SDR9C5   H. sapiens       ------------------------------------------------------------
SDR9C6   H. sapiens       ------------------------------------------------------------
SDR9C7   H. sapiens       ------------------------------------------------------------
SDR9C8   H. sapiens       ------------------------------------------------------------
SDR9-1C2 C. intestinalis  TLLPKSSSSTLTSGYSSLSSTSASLKKKMPSGVMANPGDYDPETQKEEKKRRSLSLGGGA
SDR9-1C2 S. purpuratus    ------------------------------------------------------------
SDR9-1C2 C. elegans       ------------------------------------------------------------
SDR9-1C4 C. elegans       ------------------------------------------------------------
SDR9-1C2 A. californica   ------------------------------------------------------------
SDR9-1C5 A. californica   ------------------------------------------------------------

SDR9C1   H. sapiens       --------GSTDTSPVIDAVTHALTATTPYTRYHPMDY--YWWLRMQIMTHLPGAISDMI
SDR9C2   H. sapiens       --------ASKDFSPVLRDIQHAILAKSPFAYYTPGKGAYLWIC---LAHYLPIGIYDYF
```

```
SDR9C3   H. sapiens       --------AMSDLTPVVDAITDALLAARPRRRYYPGQGLGLMYF---IHYYLPEGLRRRF
SDR9C4   H. sapiens       --------VNMDLSPVVECMDHALTSLFPKTHYAAGKDAKIFWI---PLSHMPAALQDFL
SDR9C5   H. sapiens       --------CDPDLTKVSRCLEHALTARHPRTRYSPGWDAKLLWL---PASYLPASLVDAV
SDR9C6   H. sapiens       --------CSTNLNLVTDCMEHALTSVHPRTRYSAGWDAKFFFI---PLSYLPTSLADYI
SDR9C7   H. sapiens       --------AEPRVRDVINSMEHAIVSRSPRIRYNPGLDAKLLYI---PLAKLPTPVTDFI
SDR9C8   H. sapiens       --------CTQDLSLVTNCMEHALIACHPRTRYSAGWDAKLLYL---PMSYMPTFLVDAI
SDR9-1C2 C. intestinalis  SSTLKSSPDFGNTNRVLHALSEAVTAYSPKVRYFVGSFQDRMVK--SASAFLPTVVMDTY
SDR9-1C2 S. purpuratus    --------ATTDLSKVTDVIEHALFSKWPKTRYAVGWDAKMVWV---PMSYMPSWIQDRL
SDR9-1C2 C. elegans       --------STTKIHYVVDNYYHALTAKYPRHRYYCGWDAIFVYV---PLSLLPTWWADYV
SDR9-1C4 C. elegans       --------CADDLNPVIDAYDHALLGKYPKTRYWVGWDTILFYI---PLATLPTFAQDWF
SDR9-1C2 A. californica   --------YPRDLSPVIRCLRSALLSKRPRERFPCGGGAEILMC---VYPLLPVWMADCV
SDR9-1C5 A. californica   --------ASTDIDQVVDAYVHAITSKFPRTRYVVGNDAKFIYI---PLSLVPDWCGDWI
                                      *         *     *                       *

SDR9C1   H. sapiens       YIR------------------------------------------
SDR9C2   H. sapiens       AKRHFGQDK-PMPRALRMP--------------NYKKKAT-----
SDR9C3   H. sapiens       LQAFFIS-H-CLPRALQPGQPGTTPPQDAAQDPNLSPGPSPAVAR
SDR9C4   H. sapiens       LLKQKAEL--ANPKAV-----------------------------
SDR9C5   H. sapiens       LTWVLPKP--AQAVY------------------------------
SDR9C6   H. sapiens       LTRSWPKP--AQAV-------------------------------
SDR9C7   H. sapiens       LSRYLPRP--ADSV-------------------------------
SDR9C8   H. sapiens       MYWVSPSP--AKAL-------------------------------
SDR9-1C2 C. intestinalis  LTSGSLSK--CVPKVIKEKQE------------------------
SDR9-1C2 S. purpuratus    CP-------------------------------------------
SDR9-1C2 C. elegans       IRSLGKQE--VVPACLEDSMK----------KKN-----------
SDR9-1C4 C. elegans       ISFQRCSR--PVPASMKNHH-------------------------
SDR9-1C2 A. californica   SSSINLVPKQLRPQALSGMTT----------R-------------
SDR9-1C5 A. californica   VRSLINYT--ISKKR------------------------------
```

Figure S26. Alignment of the invertebrate orthologs of the human SDR9C family variants. For further details see Fig. S25 and Table S28.

* symbol marks the position of identical amino acid residues of the aligned SDR protein sequences.

```
SDR10E1 H. sapiens         ------------------------------------------------------------
SDR10E2 H. sapiens         ------------------------------------------------------------
SDR10-1E1 C. intestinalis  ------------------------------------------------------------
SDR10-2E1 C. intestinalis  ------------------------------------------------------------
SDR10-1E1 S. purpuratus    ------------------------------------------------------------
SDR10-1E1 D. melanogaster  MSHAVANKTETEAAPNSSLKQSAAPQPANSH------------------DAKLLNGTLAR
SDR10-1E1 A. gambiae       ------------------------------------------------------------
SDR10-1E1 A. mellifera     ------------------------------------------------------------
SDR10-1E1 C elegans        ------------------------------------------------------------
SDR10-1E1 A. californica   MSSEDCEQG------GGEMTVSSPQQPSKTNQSSSIVKDLIMKTTMVPEKEVVVNGFHNT


SDR10E1 H. sapiens         ------------------------------------------------------------
SDR10E2 H. sapiens         ------------------------------------------------------------
SDR10-1E1 C. intestinalis  ------------------------------------------------------------
SDR10-2E1 C. intestinalis  ------------------------------------------------------------
SDR10-1E1 S. purpuratus    ----------------------------MPCF--DDNGPICAPLDG--------------
SDR10-1E1 D. melanogaster  TNGL----------------------------------------THAASVATSSSGSYGS
SDR10-1E1 A. gambiae       ------------------------------------------------------------
SDR10-1E1 A. mellifera     ------------------------------------------------------------
SDR10-1E1 C. elegans       ------------------------------------------------------------
SDR10-1E1 A. californica   LNGAGEDQETLRKPGDSNTFTNSNGSNKIPTSMDPVTSPLSSPLTQVASTLAPSLKQYED


SDR10E1 H. sapiens         -----------------------------------------------------------M
SDR10E2 H. sapiens         -----------------------------------------------------------M
SDR10-1E1 C. intestinalis  -----------------------------------------------MA--DSQEIPVNV
SDR10-2E1 C. intestinalis  -------------------------------------------------MDYSDVLPkkY  1p.0
SDR10-1E1 S. purpuratus    ----EHSSGSGGV-----------------------VDVTDEAFA---mkLNNLPASMAY  1p.0
SDR10-1E1 D. melanogaster  SSAAGSNAGSGGPTSSASLSIAAGVASSTALPLPPSSNGLQMPYE------RFRADDTSY
SDR10-1E1 A. gambiae       ------------------------------MDDEQQRSMATTVVQREMMATTTTTTAAAA
SDR10-1E1 A. mellifera     ---------------------------------------------------MSTISDNQC
SDR10-1E1 C. elegans       ----------------------------------------------------------MA
SDR10-1E1 A. californica   FLPKTTQDGVGSPSRTTP-----------------EMSILHEHYEHRHATSPKLAVSAGE

                                          ×  ×  ×
SDR10E1 H. sapiens         VSIPEYYEGKNVLLTGATGFLGKVLLEKLLRSCPKVNSVYVLVRQKAGQTPQERVEEVLS
SDR10E2 H. sapiens         STIAAFYGGKSILITGATGFLGKVLMEKLFRTSPDLKVIYILVRPKAGQTLQQRVFQILD
SDR10-1E1 C. intestinalis  PSIAEFFSGKSVAITGATGFIGTCLVEKILRCCPGVLKIFVFIREKKDKSATERLAHLTQ
SDR10-2E1 C. intestinalis  PTIADFYAGKTVAMTGGTGFLGQGITEKLLRCCPDINKIILFIRHKRNCDPKERLQQLAQ
SDR10-1E1 S. purpuratus    CSIGEFYAGKTLMITGATGFIGKVMLEKLMRCCPDIKKVFLLIRPKSGQRAAARIQEITA
SDR10-1E1 D. melanogaster  VPIAQFYAGRSVFITGGTGFMGkvLVEKLLRSCPEIRNIYLLIRPKRGQEVSARLTELLN  1p.0
SDR10-1E1 A. gambiae       ASIAESYAGRSIFITGATGFMGKIMVEKLLRDCGELRCIYLLIRAKKGVDPAQRKEEYVK
SDR10-1E1 A. mellifera     TSVRDFYKDRSIFITGGTGFMGKVLVEKLLRSCPGIKNIYILMRPKKSQDIQQRLQKLLD
SDR10-1E1 C. elegans       FSVRDVYAGSSVLLTGGTGFLGKVIVEKLLWTIDDIQNIYLMIRTRKGKNPQERLSGLLh  1p 0
SDR10-1E1 A. californica   LTVPLYYAGKSVLLTGGTGFVGKVFIEKLLRSCPELDSLYCLIRPKNKQSIEARLNEITS
                                         ** *** *    **               *         *

SDR10E1 H. sapiens         GklFDRLRDENPDFREKIIAINSELTQPKLALSEEDKEVIIDST----------------  1p.0
SDR10E2 H. sapiens         SklFEKVKEVCPNVHEKIRAIYADLNQNDFAISKEDMQELLSCT----------------  1p.0
SDR10-1E1 C. intestinalis  LqvFDTVRTQQPDFQLKLVAVPCDLEKEGFDLSEESQTQIQNEV----------------  1p.0
SDR10-2E1 C. intestinalis  KpaFDILRKTQPNFAEKLAFVSCDLEADDLGLSKEDRKTLQNEV----------------  2p.0
SDR10-1E1 S. purpuratus    GllFDKVREAQPNFQSKLIAIDCDLTEPDLALKEEDIKTLQEETELAFHVAATVRFDEKL  2p.0
SDR10-1E1 D. melanogaster  AplFESLRQEKPKELSKVIPISGDITSEELGISEKDqnLLCRNV----------------  2p.0 3p.0
SDR10-1E1 A. gambiae       NIVFDHVRERYSERLGKIRLIRGDILSPGLGLSDDDRRELTENV----------------
SDR10-1E1 A. mellifera     VplFDKLRRDTPDELLKIIPIAGDVTEHELGISEADQNVIIRDV----------------  1p.0
SDR10-1E1 C. elegans       dPLFNRIRQEKPEAFDKLKAIGGDMMVENLGMDPEDVMLIRDnv----------------       2p.0
```

```
SDR10-1E1 A. californica   TklFDRLRKERPDFASKLRPIHGDMLLPDLGLSPEDRAELEQKI----------------  1p.0
                              *            *

SDR10E1 H. sapiens         ------------------------------------------------------------
SDR10E2 H. sapiens         ------------------------------------------------------------
SDR10-1E1 S. purpuratus    slSLHLNVYATKKILQLAQGMKKLllFDKVREAQPNFQSKLIAIDCDLTEPDLALKEEDI  3p.1 4p.0
SDR10-1E1 C. intestinalis  ------------------------------------------------------------
SDR10-2E1 C. intestinalis  ------------------------------------------------------------
SDR10-1E1 D. melanogaster  ------------------------------------------------------------
SDR10-1E1 A. gambiae       ------------------------------------------------------------
SDR10-1E1 A. mellifera     ------------------------------------------------------------
SDR10-1E1 C. elegans       ------------------------------------------------------------
SDR10-1E1 A. californica   ------------------------------------------------------------


SDR10E1 H. sapiens         -------NIIFHCAATVRFNENLrdAVQLNVIATRQLILLAQQMKNLEVFMHVSTAYAYC  2p.1
SDR10E2 H. sapiens         -------NIIFHCAATVRFDDTLrhAVQLNVTATRQLLLMASQMPKLEAFIHISTAYSNC  2p.1
SDR10-1E1 C. intestinalis  -------NIFIHSAATLRFNEHIrlSYQINTLGVRTMLKLCRTIKNLVSIVHIStaYSFC  2p.1 3p.2
SDR10-2E1 C. intestinalis  -------NVFYHSAATLKFNEHLriSFDINVQCVRKLLKLR--WDNLQAFVHIStaYAHT  3p.1 4p.2
SDR10-1E1 S. purpuratus    KTLQEETELAFHVAATVRFDEKLSLSLHLNVYATKKILQLAQGMKKLlvFQHVSTAYANC  5p.0
SDR10-1E1 D. melanogaster  -------SVVFHSAATVKFDEKLKLSVTINMLGTKRLVELCHRMLSLdaLIHVSTAYCNC  4p.0
SDR10-1E1 A. gambiae       -------ELVFHCAANVRFDQHIRQAVDINLNGTIRVLGLAEQMRRLVSFVHVSTAYCQC
SDR10-1E1 A. mellifera     -------SIVFHSAATVKFDEPLKRSVHINMIGTKQLLNLCHRMHNLeaLIHVSTAYCNC  2p.0
SDR10-1E1 C. elegans       -------NVVIHSAATVKFDEHLRAAVTMNVIGTKRIIDLCHQIKDLkvLVHVSTAYANC  3p.0
SDR10-1E1 A. californica   -------NIVFHSAATIRFDEPLRVAVEMNVVAVRKMLALARKLEHLeaFVHVSTAYANC  2p.0
                                      * **   *          *                *    * ****

                                                                        +   +
SDR10E1 H. sapiens         NRKHIDEVVYPPPVDPKKLIDSLewMDDGLVNDITPKLIGDRPNTYIYTKALAEYVVQQE  3p.1
SDR10E2 H. sapiens         NLKHIDEVIYPCPVEPKKIIDSLewLDDAIIDEITPKLIRDWPNIYTYTKALGEMVVQQE  3p.1
SDR10-1E1 C. intestinalis  DRKDIGEEVYKTGWNFNKLHDTMQWMNDDMLTKLTPDILRDrpNTYTLTKAFGEEVIVKE  4p.1
SDR10-2E1 C. intestinalis  DRKFVGEEFYDCGVDYLDLEAMFKWMDDDTVEQLTPSLLKSrpNTYTLTKALAEDAVCRE  5p.1
SDR10-1E1 S. purpuratus    DRSRIEEVVYPPPVDPYKMLDAVewMSEDMIQTLTPKLLGKRPNTYTFTKALAEYVVMEE  6p.1
SDR10-1E1 D. melanogaster  DRTDVSEVIYAPPYNPDDIISLINWLPEDILDqlTPRLIGKRPNTYTFTKALAEHMLLKE  5p.0
SDR10-1E1 A. gambiae       NEAVLEEKHYPAPQNPEGISKMVGLLDDDLLDIITPrlLNNLPNTYAYTKALTEDMVYQY  1p.1
SDR10-1E1 A. mellifera     DRYDVAEEIYPVSAEPEEIMALTKLMDSQMIDNITPTLIGNRPNTYTFTKALTERMLQSE
SDR10-1E1 C. elegans       DRFETTEkiYKSPMAPQKLVDALSWMDDETLTkiTPKILGLRPNTYTLTKALAESTIETE  4p.0 5p.0
SDR10-1E1 A. californica   DRPYIEETVYPPPVEPQKIIDVLewMDDEMLELFTPKLMGEKPNTYTYTKQLAEHLLITE  3p.1
                                 *  *                        **      ** *  **   *

SDR10E1 H. sapiens         GAKLNVAIVRPSIVGASWKEPFpgWIDNFNGPSGLFIaaGKGILRTIRASNNALADLVPV  4p.0 5p.0
SDR10E2 H. sapiens         SRNLNIAIIRPSIVGATWQEPFpgWVDNINGPNGIIIatGKGFLRAIKATPMAVADVIPV  4p.0 5p.0
SDR10-1E1 C. intestinalis  GEGLPVCIVRPSIVGATYSDPVagWCSNFNGATGLFIAYGKGLMRSLYVKRDICMDIIPA  5p.0
SDR10-2E1 C. intestinalis  SGNLPICIVRPSMIIPAWREPMpgWCTNVYGPTAFIIAYGKGVLRCCLADRKINADNIP   6p.0
SDR10-1E1 S. purpuratus    GKGMPICITRPSIVGASWKEPFpgWIDNFNGPSGVFIavGKGLLRTMIGDADAVVDISPV  7p.0 8p.0
SDR10-1E1 D. melanogaster  AGNLPVAIVRPSivTASLNEPFAGWVDNFNGPTGLVSALAKGMFRTMMCEKNYVADMVPV  6p.0
SDR10-1E1 A. gambiae       RGKLPIAIARPSIVTAAMKEPLPGWGEGTNGPTGLLIGAGRGVIRSMHCNGEYLADFMPV
SDR10-1E1 A. mellifera     CGHLPIAIVRPSIVLSSFREPVSGWVDNLNGPTGIVAAAGKGFFrsMLCQKNMVADLVPV  3p.1
SDR10-1E1 C. elegans       AKDIPVIIIRPSIVGAMWQGPLPGWTDNINGPTGIFAAVGRGVLTNMCGSSESKADIIPV
SDR10-1E1 A. californica   GAGLPLAIVRPSIVGAAWKEPFpgWIDNYNGPSGIYIAAGRGILRSMKGESKAIADIVPV  4p.2
                                  * ***        *  **     *          *             *  *

SDR10E1   H. sapiens       DVVVNMSLAAAWYS-GVN------------------------------------------
SDR10E2   H. sapiens       DTVVNLMLAVGWYT-AVH------------------------------------------
SDR10-1E1 C. intestinalis  DLVVNGTIAAAWRNAVCHNPVSANSVLSTSPLidRRSNSTFSSASDLSEL------RTLG  6p.2
SDR10-2E1 C. intestinalis  DFIVNGAVSAGMKT-ACD-------------FVHRRRSSGYNSSDGEESLDDGVEELSSG
SDR10-1E1 S. purpuratus    DFVVNAMIGATWHT-GVH------------------------------------------
```

```
SDR10-1E1 D. melanogaster  DIVINLMIAAAWRT-ATR------------------------------------------
SDR10-1E1 A. gambiae       DVTMNAIIAIGTERMANA------------------------------------------
SDR10-1E1 A. mellifera     DIVINLMICTAWRT-ATN------------------------------------------
SDR10-1E1 C. elegans       DIVANMIIASASYR-TSI------------------------------------------
SDR10-1E1 A. californica   DIPVNVMIVVAWYT-AVA------------------------------------------
                           *   *

SDR10E1    H. sapiens       ---------------------------rpRNIMVYNCTTGSTNPFHWGEveYHVISTFKR  6p.1  7p.2
SDR10E2    H. sapiens       ---------------------------rpKSTLVYHITSGNMNPCNWHKmgVQVLATFEK  6p.1  7p.2
SDR10-1E1 C. intestinalis R---------------EREEESPEIELRRKTLPIYNLVASSSNPVLMREwnDIMTSSYTN        7p.2
SDR10-2E1 C. intestinalis TERSDSPGSsvFAESAVVEIPSSEAARKSTKIPIYNINSSTTNPLLLHDleIYTKETIDV  7p.2  8p.2
SDR10-1E1 S. purpuratus   ---------------------------kpANIPIYNLVTSPVNPNRWGDveII-PDLYNR  9p.1 10p.2
SDR10-1E1 D. melanogaster ---------------------------KSNNLLIYNCCTGQRNPIIWSEFVKHAMTSVRK
SDR10-1E1 A. gambiae      ---------------------------RKEDVMYYNLTSSADNPISWGEVLEMGRGILNQ
SDR10-1E1 A. mellifera    ---------------------------RTKTIPIYHCCTGQQNPITWQQFVELILKYNRM
SDR10-1E1 C. elegans      ---------------------------ntTEIPVIHCSSGELNPLYWGHIVLFLEQFYKk  6p.1  7p.0
SDR10-1E1 A. californica  ---------------------------KPGQVLTYHATTGGCNPYTWGDisGIVNKSYKQ        5p.2
                                                                  **

SDR10E1    H. sapiens       NPLEQA-FRRPNVNLTSNHLLYHYWIAVSHKAPAFLYDIYLRMTGRSPrmMKTITRLHKA        8p.1
SDR10E2    H. sapiens       IPFERP-FRRPNANFTSNSFTSQYWNAVSHRAPAIIYDCYLRLTGRKPrmTKLMNRLLRT        8p.1
SDR10-1E1 C. intestinalis YPLDA--LMSPDLKVASNklMFRVFLFFKQYIPAYIFDAGLILIGKKPqlLRWTQRVSGT  8p.1  9p.1
SDR10-2E1 C. intestinalis YPMKSASFFPPSCILTTNqkVMDYATFALQTVPAHIIDTILALTKQKRqmVKWDGKIRSG  9p.1 10p.1
SDR10-1E1 S. purpuratus   IPLEKV-FRRPRAALTKYplAFEYVNLVSAKIPAYIMDCALRLQGKKPqmMKVNSKITKM 11p.1 12p.1
SDR10-1E1 D. melanogaster HPleGC-LWYPTGDLRMNRPMNTLNCIAKHFLPAYILDGVARIMGKKPfvVNVQNKIAKA  7p.2  8p.1
SDR10-1E1 A. gambiae      NPFCFA-LWYPDGSIKSNYLYHLLCVVLFHYLPAYLIDFLMVLLRRKPflVKVQKRISAG        2p.1
SDR10-1E1 A. mellifera    HPPNDT-IWWPDGKCHTFAIVNNVCKLFQHLLPAHILDFIFRLRGKPAIMVGLHEKIDKA
SDR10-1E1 C. elegans      yPMEQC-FAVPSTYFHKSRSLFLINYYIKHHIPAAISDISARLIGKRKnnVKLYSKVWKM        8p.1
SDR10-1E1 A. californica  IPLEGC-FRRPNVAIVSNnfLHEYWTCVSHLIPAYMIDLAYRMMGKKPprMVRLYNKLHRS  6p.1  7p.1
                           *        *                      **    *

SDR10E1    H. sapiens       MVFLEYFTSNSWVWNTENVNMLMNQLNPEDKktFNIDVRQLHWAEYIENYCLGTKKYVLN  9p.0
SDR10E2    H. sapiens       VSMLEYFINRSWEWSTYNTEMLMSELSPEDQrvFNFDVRQLNWLEYIENYVLGVKKYLLK  9p.0
SDR10-1E1 C. intestinalis IGVLQFFLTSQWNWSNNSIQKLQREMNEQDRkqFNMDAAVVDWEQYMNNYAKGTKKFILK 10p.0
SDR10-2E1 C. intestinalis MEVMGFFFTNGFVWNRDETDRLHNCLHPKDQktFTLDARAFVWREYFNDYILGAKKFILK 11p.0
SDR10-1E1 S. purpuratus   VHTLKYFTNNTWEWTNQNTIALSAAMSEEDRkvYFTDVRPLHWPTYLEAYCLGTKKYVLK 13p.0
SDR10-1E1 D. melanogaster VECLEYFATRQWRFKDDNVHALLHTLSPKDREIFVFDVRHINWDKYVERYVLGFREFLFK
SDR10-1E1 A. gambiae      LTILQYYTTKEWVFRCDNTKSLYQRLSPDDRKRFYFDVNEINYKTYLYDFILGARQYILK
SDR10-1E1 A. mellifera    VKCLEYFTMQQWNFRDDNVRQLSGELSPEDRQIFMFDVKQIDWPSYLEQYILGIRQFIIK
SDR10-1E1 C. elegans      IETLHFFTTRGWSFNARGLPEFFEKMTPADQKEYNFDVRQVDWNSYLFDYVMGIKKFLLK
SDR10-1E1 A. californica  IDVLKFFTCNEWQWSSTNHDMLKHQLTPLDQkmFYFDPKPLHWPTYIENYCMGAKKYVLN  8p.0
                                                       *      *        *      *

SDR10E1    H. sapiens       EEMSGLPAARKHLNklRNIRYGFNTILV---IL--------------IWRIFIARSQM-- 10p.1
SDR10E2    H. sapiens       EDMAGIPKAKQRLKrlRNIHYLFNTALF---LI--------------AWRLLIARSQM-- 10p.1
SDR10-1E1 C. intestinalis EDPMNYPAARRHVQrlRILGYCSQLLVF---MV--------------VWRLLVPRSGI-- 11p.1
SDR10-2E1 C. intestinalis ENIEDFSKEQKAMQrmKTLSKVGLQTIAGVSALGLASAVGLTSSLWELSSYVLG------ 12p.1
SDR10-1E1 S. purpuratus   EDMNDIPAARSHLKmlRNIRWTFNTVLL---VV--------------FWRVLIARSQL-- 14p.1
SDR10-1E1 D. melanogaster QRPESLPASRKRMLrlYYLHQLTKLVAV---LL--------------TWRFLMSRSKR--  9p.1
SDR10-1E1 A. gambiae      EAPETLPKARKLLRklYIMDKIVQIGLY---LF----------GLWLAWTYLEVVT----  3p.1
SDR10-1E1 A. mellifera    DSPETLPAARSHIKklYWIQKVVEFGML---LV--------------VLRFLLLRIPM--  4p.1
SDR10-1E1 C. elegans      ENLENLNRSRAHLLkmRIRRQVIAAIVY---AG--------------FISTI-GRKWKKT  9p.1
SDR10-1E1 A. californica  EDLSGLPAARAHVRklRIIRYLFNMSVA---VV--------------LWRVLIAKSQF--  9p.1
```

```
SDR10E1    H. sapiens       -ARNIWYFVVSLCYK--FLSYFRASSTMRY----------------
SDR10E2    H. sapiens       -ARNVWFFIVSFCYK--FLSYFRASSTLKV----------------
SDR10-1E1 C. intestinalis  -AKNLWHLFMSLWFK--FLGFFQISSTLHRSNFFSR-----ILSSS
SDR10-2E1 C. intestinalis  -DGLSWFV--------------------------------------
SDR10-1E1 S. purpuratus    -ARNLWAFVINIFFR--FLRYCRVTSTIAR---TH-----------
SDR10-1E1 D. melanogaster  -LNDLWSSFLENALR--M---ARLIPFL------------------
SDR10-1E1 A. gambiae       --G-----SIQFVFD-------TAIESLRG--TTVRQGL-------
SDR10-1E1 A. mellifera     -AQSACFTLLSAILR--M---CRMIV--------------------
SDR10-1E1 C. elegans       TQYLTWFGAMLATYTYTEVSFRRHIPLKSLKDYTQTSDYSRYLQKS
SDR10-1E1 A. californica   -ARNLWFFIVGLVSK--FVQYFRITSTMHK----------------
```

Figure S27. Alignment of the invertebrate orthologs of the human SDR10E1 family variants. For further details see Fig. S25 and Table S29.

* symbol marks the position of identical amino acid residues of the aligned SDR protein sequences.

```
                                        x  x  x
SDR11E1   H. sapiens     ----MAMTGWSCLVTGAGGFLGQRIIRLLV---KEKELKEIRVLDKAFGPELREEFskLQ  1p.2
SDR11E2   H. sapiens     -------MGWSCLVTGAGGLLGQRIVRLLV---EEKELKEIRALDKAFRPELREEFskLQ  1p.2
SDR11E3   H. sapiens     MADSAQAQKLVYLVTGGCGFLGEHVVRMLLQ--REPRLGELRVFDQHLGPWLEELKtg--  1p.2
SDR11-1E1 S. purpuratus  ---MPGTEGEVVMVTGASGFLGQHILKQLLEQ-GEFLIKEVRTFDLQPFTWCPEL-evHN  1p.2
SDR11-1E1 M. domestica   -----MASGEVVLVTGGSGFIGQHIIRRLLKDKSELGIKEVRSVDLNAFKNTI---dd--  1p.2
SDR11-1E1 C. briggsae    --------MYTVAVTGGAGLVGRYVVQRLLE---NEQIAEVRVIDRQSTS------re--  1p.2
                                      ***  *  *      *          * *  *

SDR11E1   H. sapiens     NKTKLTVLEGDILDEPFLKRACQDVSVIIHTACII----DVFGVTHRESIMNVNVkgTQL  2p.2
SDR11E2   H. sapiens     NRTKLTVLEGDILDEPFLKRACQDVSVVIHTACII----DVFGVTHRESIMNVNVkgTQL  2p.2
SDR11E3   H. sapiens     -PVRVTAIQGDVTQAHEVAAAVAGAHVVIHTAGLV----DVFGRASPKTIHEVNVqgTRN  2p.2
SDR11-1E1 S. purpuratus  PTTQLSHIKGDLLCMEEVRRAFKGVTVVIHTAGVI----DVSPVPDAELLRSVNIqgSEN  2p.2
SDR11-1E1 M. domestica   EKLDIKTFVGDLCEPDSVEDAFKGVDTVFHCAGKM----SLQYPPKYDELNRNNIeaTQS  2p.2
SDR11-1E1 C. briggsae    ESRKLKLYLMDLNDRKPLENALLGCDGVIHCAHAPFPILYSKNKAEIDQMWRDNLdaCES  2p.2
                                   *         *        * *                     *

SDR11E1   H. sapiens     LLEACVQASVPVFIYTSSIEVAGPNSYKEI---IQNGHEEEP-------------LENTW
SDR11E2   H. sapiens     LLEACVQASVPVFIYTSSIEVAGPNSYKEI---IQNGHEEEP-------------LENTW
SDR11E3   H. sapiens     VIEACVQTGTRFLVYTSSMEVVGPNTKGHP---FYrgNEDTP-------------YEAVH  3p.1
SDR11-1E1 S. purpuratus  VLQACIHHNIQYLVYTSTVDVVIGQ---EP---ITAGTETIL-----------GIPQHHH
SDR11-1E1 M. domestica   VVDLCLKHNVQRLIYTSCGSVCMVPFKGSSSTVVINQTESKAATPMFDAKKPEEFDKQFI
SDR11-1E1 C. briggsae    VVDTMISMNIRNLVNIGCAYSPIPNEDNYG-----------LAQDV-----FldYPRNYM  3p.1

                            +   +
SDR11E1   H. sapiens     PAPYPHSKKLAEKAVLAANGWNLKNGG-TLYTCALRPMYIYGEGSRFLSA-SINEALNNN
SDR11E2   H. sapiens     PTPYPYSKKLAEKAVLAANGWNLKNGD-TLYTCALRPTYIYGEGGPFLSA-SINEALNNN
SDR11E3   H. sapiens     RHPYPCSKALAEWLVLEANGRkvRGGL-PLVTCALRPTGIYGEGHQIMRD-FYRQGLRLG  4p.0
SDR11-1E1 S. purpuratus  FGLYATTKYEAEKIVLKASNLILKNGK-RLQTCALRPTPVYGEGDTYNRD-VLRQACHYK
SDR11-1E1 M. domestica   IRGYSSSKLKAEQIVLSAHGKTLKNGTGQLTTAAIRPPLTYGEGDTFFMPDMLRYLSRHS
SDR11-1E1 C. briggsae    LDKYGESRTRGEMYARKAA------KRGSLNALFLRPTFVYGQGKSRKIE-AIKELVQNA
                            *       *     *           *     **   ** *

SDR11E1   H. sapiens     GILS-SVGKFSTVNPVYVGNVAWAHILALRALQDPKKAPSIRGQFYYISDDTPHQSYDNL
SDR11E2   H. sapiens     GILS-SVGKFSTVNPVYVGNVAWAHILALRALRDPKKAPSVRGQFYYISDDTPHQSYDNL
SDR11E3   H. sapiens     GWLFRAIPASVEHGRVYvgNVAWMHVLAARELEQ--RATLMGGQVYFCYDGSPYRSYEDF  5p.2
SDR11-1E1 S. purpuratus  MMV-RMGSESSRYQCTYAGNIAWGHVLAVKELLKPTTNESPAGQAFFLTDETPVSKVSDF
SDR11-1E1 M. domestica   YVYPKIAGAGGKQQLCYagNVAWGHMCAYKSLKA--APKSVGGLPVFITDDTPLYDTSRF  3p.2
SDR11-1E1 C. briggsae    ALPFVTGERRGMHQfiYAGNLAAIAEKSFFCLLQ--DQKKFNGEIVFCMDENCAHSIRdf  4p.0 5p.0
                                         * ** *         *          *      *

SDR11E1   H. sapiens     NYTLSKEFG-LRLDSRW-SFPLSLMYWIGFLLEIVSFLLRPIYTYRPPFN---RHIVTLS
SDR11E2   H. sapiens     NYILSKEFG-LRLDSRW-SLPLTLMYWIGFLLEVVSFLLSPIYSYQPPFN---RHTVTLS
SDR11E3   H. sapiens     NMEFLGPCG-LRLVGARPLLPYWLLVFLAALNALLQWLLRPLVLYAPLLN---PYTLAVA
SDR11-1E1 S. purpuratus  FTPFVIGVD-AK--MSSFSLPFWLLYSIAVVIEICAWLLQPIYKVKFFLT---TATVTYA
SDR11-1E1 M. domestica   IQKIGLVSGKYKARHSSWSIPHFIFYFFAMLTELFIDLLEPVKKITLKYSI--RSLSSYA
SDR11-1E1 C. briggsae    FENRFLNPN-Y-------SNDVAVNYWPSFVSSYTTYWKNQLGLpvPDKSLNHVSFRLFF  6p.0

SDR11E1   H. sapiens     NSVFTFSYKKAQRDLAYKPLYSWEEAKQKTVEWVGSLVDRHKE----TL--KSKTQ
SDR11E2   H. sapiens     NSVFTFSYKKAQRDLAYKPLYSWEEAKQKTVEWVGSLVDRHKE----TL--KSKTQ
SDR11E3   H. sapiens     NTTFTVSTDKAQRHFGYEPLFSWEDSRTRTILWVQAATGSAQ--------------
SDR11-1E1 S. purpuratus  YGVYYFQCEGAERCLGYEPLYTYDDAVERSLVYYRRECGLS---------------
SDR11-1E1 M. domestica   SSIIFFNRLRASIHMDYVPLIDEETSVGNSAKWYGKWWEENIGKGQRNGVAKSKNN
SDR11-1E1 C. briggsae    EKTVGFPNKKLRLLLDSKPEISQEEALANYANPKSSFTTDSTKRSAVKG-------
                                           *
```

Figure S28. Alignment of the invertebrate orthologs of the human SDR11E family variants. For further details see Fig. S25 and Table S30.

* symbol marks the position of identical amino acid residues of the aligned SDR protein sequences.

```
SDR12C1     H. sapiens      -------------------------ME----------SALPAAGFLYWVGAGTVA--YLA
SDR12C2     H. sapiens      --------------------------------------MGDVLEQFFILTGLLVCLACLA
SDR12C3     H. sapiens      ----------------MAAVDSFYLLYREIA---RSCN--CYMEALALVGAWYT------
SDR12C-1C1 C. intestinalis  ----------------MAAVDSVKLLVNQMTSDLQNYSSF-AWNLLSVIGAWYTLKYSVK
SDR12C-2C1 C. intestinalis  -------------------------ME---------------CWLFTYIGIAT-C-SIVA
SDR12C-1C1 S. purpuratus    ---------------MAAVVDGFDLLFRQVS---NGFH--IYSEVLSLIGAYYV------
SDR12C-2C1 S. purpuratus    ------------------------------------------------------------
SDR12C-1C1 D. arizonae      -------------MQPALELSIYTLLRMAF-------IWQLISVAIYLVGTLAIAAYLYD
SDR12C-1C1 D. ananassae     -------------------------MD----------SEL--LNILSVISISV-------
SDR12C-1C1 A. gambiae       --------------------------M----------VTT--SSVLGSTSAVL-------
SDR12C-2C1 A. gambiae       ---------------------------MLI-------ASQLALWGLALIGVWALAWWMYD
SDR12C-3C1 A. gambiae       ----------------------------------------MFCSLAACLGVYVAAVWLYE
SDR12C-1C1 A. mellifera     MMFHLRFFIGVHDDLPSLPVPTFQPFFSATKFKDRDYIQFIwkMILEIV--FWLF----F-  1p.1
SDR12C-1C1 C. elegans       -----------------MESSDNLHDIDNL--------EngNMACQCFLVGAGYVALAAV-  1p.2
SDR12C-1C1 C. remanei       -----------------------------------------MDLQWFAAGAGAVALLFI-
SDR12C-1C1 A. californica   -------------------------MIRQ--------YLGDSVDLFSVIGAGVTV--FVA
SDR12C-2C1 A. californica   -MLSNRWFGS------------FRTFSLQLT-PSRILHFSDFIDVFAFLGAYYFVKKCLK

                                                                            ×   × ×
SDR12C1     H. sapiens      LRISYSLFTALRVWGVGNEAG-------------VGPGL--GEWavVTGSTDGIGKSYAE  1p.2
SDR12C2     H. sapiens      KCVRFSRCVLLNYWKVL---------P-----KSFLRSM--GQWavITGAGDGIGKAYSF  1p.2
SDR12C3     H. sapiens      ARKSI----TVICDFYSLIRLHFI--PRLGSRADLIKQY--GRWAVVsgATDGIGKAYAE  1p.1
SDR12C-1C1 C. intestinalis  ----------FCCWFISCCK----CAHKYWRKRNFVKEY--GRWAVVtgCSSGIGKQFMH  1p.2
SDR12C-2C1 C. intestinalis  FKFVSKIVHALR--------------IYVFPTVPKFSKY--GKWtvVTGCTSGIGKSIAK  1p.2
SDR12C-1C1 S. purpuratus    TKKVI----VTCYRVQYVIRMHLY--PKLRPKRDLVKEF--GEWavVTGSTDGIGKAYAM  1p.2
SDR12C-2C1 S. purpuratus    -----------------------------------MHR-WSGRVALVTGASEGIGEAISR
SDR12C-1C1 D. arizonae      NFKSL------ICI-IKALLEPYF-QPQ--LPQTLLDKF--GKWavITGATDGIGKEYAK  1p.2
SDR12C-1C1 D. ananassae     --VGFQVVRKVLPWIYANVVG-----PKVFGSSVNLAKM--GEWavVTGSTDGIGKAYAK  1p.2
SDR12C-1C1 A. gambiae       --VSLFLLRKVFPWIYQNLIG-----PKVFGCRINLKKT--GEWalITGATDGIGKAYAQ  1p.2
SDR12C-2C1 A. gambiae       NLKSL------AQI-GVALLAPYF-APA--EHKTLTERY--GKWavITGSTDGIGKQYAF  1p.2
SDR12C-3C1 A. gambiae       NFRTPVLLV------LQTL-------KQLVFRQKLSQRY--GQWavITGASDGIGKGYAH  1p.2
SDR12C-1C1 A. mellifera     TLLLISLLLTYIFNMGTTLWEIFV-SLINTKTVDLRTKF--GEWavVTGSTDGIGKAYAK  1p.2
SDR12C-1C1 C. elegans       ------AYRLL--TIFSNILG-----PYVLLSPIDLKKRAGASWavVTGATDGIGKAYAF  1p.2
SDR12C-1C1 C. remanei       ------FCHFI--RVLLNILG-----PYVFSQPIDLKKKAGAPWavVTGATDGIGKSYSF  1p.2
SDR12C-1C1 A. californica   FKVAISLFRFITTHFLA----------STLGLPANLKNA--GSWavITGCTDGIGKAYAE  1p.2
SDR12C-2C1 A. californica   TAALL----------TDTVNAHVLSRLRPYSNEWWMESF--GPWavVTGSSEGIGQAYAY  1p.2
                                                                            *   ***

SDR12C1     H. sapiens      elAKHGMKVVLISRSKDKLDQVSSEikE-KF-KVETRTIAVDFAS---EDIY---DKIKT  2p.0 3p.2
SDR12C2     H. sapiens      elAKRGLNVVLISRTLEKLEAIATEieR-TT-GRSVKIIQADFTK---DDIY---EHIKE  2p.0 3p.2
SDR12C3     H. sapiens      ELASRGLNIILISRNEEKLQVVAKDIAD-TY-KVETDIIVADFSSG--REIY---LPIRE
SDR12C-1C1 C. intestinalis  KLAEHGLNIILVSRNKDCLEEEAkfIET-AY-GVQTLLVVQDLENL-TPEIT---QKIQD  2p.1
SDR12C-2C1 C. intestinalis  alAARGQNIALISRNPEKLKTVATEleT-KY-NVQTKYLVIDFTQD--ESIY---EKIEE  2p.0 3p.2
SDR12C-1C1 S. purpuratus    ELAHHGVNIILISRSNEKLRKVASEieS-FY-GVKTHVIKADFSLG--SEIY---EDIST       2p.2
SDR12C-2C1 S. purpuratus    LLVKHDMKVVGCARNLDKLkaIDGELKK-EE-GGSFFPIQCDLTQR--DQIYAMFEKIKT  1p.0
SDR12C-1C1 D. arizonae      ELARQGLNLVLISRSKEKLIAVTNEieS-EH-KVKTKWIAVDFGKG--REAY---EQIEK       2p.2
SDR12C-1C1 D. ananassae     elARRGLKLVLISRSLEKLNAVAKEigD-KY-GVEVRVIDVDFTGG--AEIY---DKIQE  2p.0 3p.2
SDR12C-1C1 A. gambiae       alARKGLNIILVSRTLSKLEDVAKEieT-EF-KVRTMVIAADFTKG--AEIY---EQIQR  2p.0 3p.2
SDR12C-2C1 A. gambiae       QLASRGLNVVLVSRSTDKLVAVAAEIES-KY-SVKTKWIAVDFSSG--REIY---DHLRR
SDR12C-3C1 A. gambiae       YLAAKGMAIVLVARNAAKLNKVADEIRA-KH-GVETKVLVADFSKG--AEIY---PQLEK
SDR12C-1C1 A. mellifera     ELATRKINLVLISRSLEKLEKTRNEIMQENP-TVEIKIIVADFSKG--KEIF---EKLAE
SDR12C-1C1 C. elegans       ELARRGFNVLLVSRTQSKLDETKKEILE-KYSSIEVRTAAFDFTNA-APSAY---KDLLA
SDR12C-1C1 C. remanei       ELARRGFNTYLVSRTQSKLEQTKKEILQ-QYSNVEVRFATYDFTNP-SPADY---QKLLD
SDR12C-1C1 A. californica   qlAAKGLNVVLISRTLSKLEDMAKEskS-KY-KVETKVIAADFSR---EDIY---DGIKL  2p.0 3p.2
SDR12C-2C1 A. californica   ELARRGLNVVLISENERRLRSTQVLleG-RF-AVKVEYIVVDFASADQTEVY---NRIWE       2p.2
                             *                *                      *
```

```
SDR12C1    H. sapiens       GLAGLEIGIlvNNVGMSYE-YPEYFLDVPDLD-nvIKKMININILSVCkmT----QLVLP  4p.2  5p.0   6p.0
SDR12C2    H. sapiens       KLAGLEIGIlvNNVGMLPNLLPSHFLNA-PDEIqsL---IHCNITSVVkmT----QLILK  4p.2  5p.0   6p.0
SDR12C3    H. sapiens       ALKDKDVGILVNNVGVFYP-YPQYFTQLSE---DKLWDIINVNIAAASLMV----HVVLP
SDR12C-1C1 C. intestinalis  RINELDIGILINNAGLH-E-SPKSFTevEI---SSLHAMVQVNMNAVVAMT----AAVLP  3p.1
SDR12C-2C1 C. intestinalis  FLQGMDIGTLVNNVGMASP--LAFYLDTKNL-SQILPAIMKVNVMSVFkmT----QIVLP               4p.0
SDR12C-1C1 S. purpuratus    KLQGMQIGIlvNNVGAM-D-YPQLFLEMDA---DRIRQLININIGAATmmT----KLVLP  4p.2         4p.0
SDR12C-2C1 S. purpuratus    HHGGV--DVCINNAGMAYP--SSLLDGTP----EEWQKSLdlNVIALCLCT----KLSVQ  2p.0
SDR12C-1C1 D. arizonae      ELAGIPVGIlvNNVGRMYD-YPETLDLVPE---DTIWDILTINIGAVTMLT----RKIVP  2p.2
SDR12C-1C1 D. ananassae     KTAGLDIGVLVNNVGISYS-HPEYFLDCYKADPQFLRNIVAANIHSVTHMS----ALFLP
SDR12C-1C1 A. gambiae       QIENMEIGVLVNNVGMSYS-NPEYLLGLPDY-EKLINNLLSCNILSVTRMC----QLVMP
SDR12C-2C1 A. gambiae       ELESVPVGIlvNNVGANVD-YPDDLDHIPE---DKLWQLININVGAVTMLT----RTVLP  2p.2
SDR12C-3C1 A. gambiae       ALVPLDVGIlvNNVGVSHD-TPMYVDEVPQ---QTLWDLIHVNVAAATLLC----NILAP  2p.2
SDR12C-1C1 A. mellifera     QLKDIPIGIlvNNVGMQYS-HPMYLEEVPE---DDLWDIININIGATTLMT----RIVIG  2p.2
SDR12C-1C1 C. elegans       TLNQVEIGVLINNVGMSYE-YPDVLHKVDGG-IERLANITTINTLPPTlfLTQLSAGILP               3p.0
SDR12C-1C1 C. remanei       KLNEVNIGILINNVGMFFE-YPDVLHEIQGG-IETLANVAVVNILPPTllS----AGILP               3p.0
SDR12C-1C1 A. californica   KMNGLDIGVLVNNVGCSYE-YPEYFGEVEEA--dfSQKMIHMNCTSVAmmT----EIVLP        4p.0   5p.0
SDR12C-2C1 A. californica   VLEDKDIGLlvNNVGVSYR-CPDVLLSISR---EDLWRLIYVNIGAATmmT----HMLLP  3p.2         4p.0
                                       ** *                           *
                                                                +   +
SDR12C1    H. sapiens       GMVErsK--GAILNISSGS---GMLPVPLLTIYSATktFVDFFSQCLHEEYRSKGV----  7p.1  8p.0
SDR12C2    H. sapiens       HMESrqK--GLILNISSGI---ALFPWPLYSMYSASkaFVCAFSKALQEEYKAKEV----  7p.1  8p.0
SDR12C3    H. sapiens       GMVERKK--GAIVTISSGS---CCKPTPQLAAFSASkaYLDHFSRALQYEYASKGI----        2p.0
SDR12C-1C1 C. intestinalis  GMLSRQR--GLIVNMSSGG---GMFPVPLISLYSSTkaFVDHFSQALHYEVASKNI----        4p.0
SDR12C-2C1 C. intestinalis  GMMERKR--GLILNISSAS---SLVPVNGFSVYGATKALVNYFSKCISRECEGHGI----
SDR12C-1C1 S. purpuratus    QMVERKC--GVVVNVSSGT---SIHPSPQLALYSACktYVDVFSQALEYEYKDYGI----        5p.0
SDR12C-2C1 S. purpuratus    QMKERGVDDGHIILLNsmGGHRLIQGSNYLHFYAGTKHMVKALTEGYRDELRQKNS---K  3p.1
SDR12C-1C1 D. arizonae      QMKAARR--GAIVNIGSGS---ELQPMPNMAVYAASKKYVSYFTQALEQELAEHNV----
SDR12C-1C1 D. ananassae     GMIAKRK--GVIINLSSTA---GVIPNPLLSVYSSTkaFVNKFSDDLQTEYKEHGI----        4p.0
SDR12C-1C1 A. gambiae       GMVKRHA--GVVINISSLS---AVIPAPLLTVYAASKAFMDKFSEDLASEYAKHNI----
SDR12C-2C1 A. gambiae       GMKKRGQ--GAIVNISSGS---ELQPLPYMTVYAATKVTVGRHPAHHGASkcHKRLVKIL  3p.2
SDR12C-3C1 A. gambiae       SMKRRQR--GLIINVSSIA---SVGPSPCMATYAASKAYMTSFSIALRDELRPFGV----
SDR12C-1C1 A. mellifera     QMQKRGK--GAIVNVSSAS---GFIPLPLMTVYSATKVYIISFTEALRAEYSKFGL----
SDR12C-1C1 C. elegans       QMVARKA--GVIVNVGSSA---GANQMALWAVYSATkkYVSWLTAILRKEYEHQGI----        4p.0
SDR12C-1C1 C. remanei       QMVSRKA--GIIVNIGSAA---GAVPMAKWSVYSASkkYVSWLTATLRKEYGHQGI----        3p.0
SDR12C-1C1 A. californica   AMVAKKR--GYVINIGSSA---GFKPAPLLALYSGTkgYVEMFTRSLYHEYIGRGV----        6p.0
SDR12C-2C1 A. californica   GMVKQGR--GGLVVVSAGS---STQITPQMTVYSATkrYLDYFIQGVAYEYRHSGL----        5p.0
                             *       *                          *

SDR12C1    H. sapiens       -FVqsVLPYFVATKLAKIRK------PTLDKPSPETFVKSAIKTVGLQSRTNGYLIHALm  9p.0  10p.0
SDR12C2    H. sapiens       -IIqvLTPYAVSTAMTKYLN------TNVITKTADEFVKESLNYVTIGGETCGCLAHEIl  9p.0  10p.0
SDR12C3    H. sapiens       -FVQSLIPFYVATSMTAPSNFLHR--CSWLVPSPKVYAHHAVSTLGISKRTTGYWSHSIq         3p.0
SDR12C-1C1 C. intestinalis  -HVQSLTPMYISTRMTDYSTTINS--NKFFTPSVETYVKHALPTLGRFRSNTGYFPHTIq         5p.0
SDR12C-2C1 C. intestinalis  -TVqsVKPFFVSTNMVNNVK------PNMLVMDADYYVNSLLGTIGKERESDGCWQHGLq  5p.0   6p.0
SDR12C-1C1 S. purpuratus    -IVQTLLPSYVATKMADFGETMPR--SRFLIPSAAVYAKHAVASIGIANRTAGYWPHAVq         6p.0
SDR12C-2C1 S. purpuratus    IRVsaLSPGLVESEFVV----------RLFGDDPDKGR--------KVLQTTpcLKREDV  4p.0  5p.0
SDR12C-1C1 D. arizonae      -TVQLVMPMFVITKMNEYSDSVMR--GGFLIPNARSFARSAVFTLGKTSMTTGFWTHGLq
SDR12C-1C1 D. ananassae     -LIQSVQPGFVATNMSKIRK------ASVFAPSPETYVRSALSTLGIATQTAGYLPHALL
SDR12C-1C1 A. gambiae       -VVQSVLPGPVATNMSKIRK------SSWMACSPKVFANSAISTLGHTRKTTGYFPHALL
SDR12C-2C1 A. gambiae       VVQGSNQTMPRFAvmNNFSTTIME--GGLFIPNAEMYAKFATFTLGKTKQTTGYWSHGIQ  4p.0
SDR12C-3C1 A. gambiae       -EVQTVRPSFVHTNMTDFLVTGKEKWSKNMMVRVDNFMAYAGCTIGKVDMTSGHWSHGLQ
SDR12C-1C1 A. mellifera     -TIQHLSPFFVNTKMNAFSNWLqv--SNILVPSATTYAKNAVNTLGKIDSSTGYWSHGIQ  4p.0
SDR12C-1C1 C. elegans       -TVQTIAPMMVATKMSKVKR------TSFFTPDGAVFAKSALNTVGNTSDTTGYITHQLQ
SDR12C-1C1 C. remanei       -IFQTITPLMVATKMAGSPD------TSFFCPTSDAFAKSALNTIGNSSDTTGYITHQLE
SDR12C-1C1 A. californica   -TFqyVAPYFVVSKLSKFRR------TSLMVPSPNAFVRNALKTVGVQAYTTGYWAHDVq  7p.0  8p.0
SDR12C-2C1 A. californica   -TFQCLMPFYVNTRMTGFSDTLTR--SRGFFPSAKQYASNAILTLGRASLTTGYFYHTLq        6p.0
```

```
SDR12C1     H. sapiens       ----gSIISNLPSWI----YLKIVMNMNKSTRAHYLKKTKK--N----------------------
SDR12C2     H. sapiens       ----aGFLSLIPAWAFYS---GAFQRLLLTHYVAYLKLNTK--VR---------------------
SDR12C3     H. sapiens       ----fLFAQYMPEWLWVW----GANILNRSLRKEALSCTA--------------------------
SDR12C-1C1  C. intestinalis  ----cYFAMLCPRFLVVK----FSHRMQLNLQKEAASIQKKAK-----------------------
SDR12C-2C1  C. intestinalis  ----gFFVRHMTEGTFNALMKR-RMKTMQRNL--SLAGKAA--KKD----N---------------
SDR12C-1C1  S. purpuratus    ----sWIARQIPRNTWMW----GANILNSALRRQAEVRRKLKIVKSSSKGKLASKSPTAESASKSV
SDR12C-2C1  S. purpuratus    AELVVMVMQQPPNVqiHD----ILVRDTDQVK----------------------------------  6p.0
SDR12C-1C1  D. arizonae      ----yFFMKMAPQHLRML----IGHQLTRRLRLEGLSQKKLT------------------------
SDR12C-1C1  D. ananassae     ----QLIIHFTEAVFGDQFARNLVLKNILGTRKRALRRLAK--EQ---------------------
SDR12C-1C1  A. gambiae       ----ELSINALSFV-SPRLAEKITINTMQNIRARAMKKSTA--RSSAPADSTAVLTA--SS-----
SDR12C-2C1  A. gambiae       ----YGVMKLVPEWIRTM----IGGMMNKQFRKEYYDQQQKPAQAS--------------------
SDR12C-3C1  A. gambiae       ----LAGLSLVPEVVRVY----IFGLINKKLREQFHVQNNNKKR----------------------
SDR12C-1C1  A. mellifera     ----KIIVLLVPIEIRTK----LAMILNMIFRKEYFKQKRNI------------------------
SDR12C-1C1  C. elegans       ----LELMDLIPTFIRDK----ILTNMSVGTRAAALRKKER--EAKSQ------------------
SDR12C-1C1  C. remanei       ----YEMMNLMPEFIIDK----AVIRSSAKLREAALAKKEE--KLLS-------------------
SDR12C-1C1  A. californica   ----rFVHSFMPAS--------MSMSMMQGARKKALKKKAA--AKSQ-------------------
SDR12C-2C1  A. californica   ----lWLAWMLPSGLWMW----GSEKLNNALRREAQNRALRRVHKSASDETMVSVAMTPA------
```

Figure S29. Alignment of the invertebrate orthologs of the human SDR12C family variants. For further details see Fig. S25 and Table S31.

* symbol marks the position of identical amino acid residues of the aligned SDR protein sequences.

```
SDR16C1    H. sapiens      ------------------------------------------------MVWKRLGALVMF
SDR16C2    H. sapiens      --------------------------------------------------MKFLLDILLL
SDR16C3    H. sapiens      --------------------------------------------------MNIILEILLL
SDR16C4    H. sapiens      --------------------------------------------------MNIVVEFFVV
SDR16C5    H. sapiens      ----------------------------------------------MSFNLQSSKKLFIF
SDR16C-1C1 C. intestinalis ------------------------------------------------MDFQDVGNLLWA
SDR16C-1C1 S. purpuratus   -----------------------------------------------MIFIDFLLDLVWM
SDR16C-1C2 S. purpuratus   ----------------------------------------------MENPIVIFFLLLWT
SDR16C-1C3 S. purpuratus   ---------------------MSDA-------------SDREHSSTAMSILEFVGELMIL
SDR16C-1C1 D. melanogaster MEKNNNELSAEDAQILANGHAHPNANGVVHQKTEPVAPAADGWRQVLWNTWDAFADVAWF
SDR16C-1C1 A. gambiae     ------------------------------------------------------MDVIVF
SDR16C-1C1 A. mellifera   -------------------------------------MQLAEWIILIY-------DFLLF
SDR16C-1C1 C. elegans     -------------------------------------------------MFDIILEIVIL
SDR16C-1C2 C. elegans     ----------------------------------------------MQDI----LESVVV
SDR16C-1C3 C. elegans     ----------------------------------------------MDRA----LDFVKM
SDR16C-1C1 A. californica ----------------------------------------------MSLF----LEFLVV
SDR16C-1C2 A. californica ------------------------------------------------MDMQIIFDGITA

                                                             ×   × ×
SDR16C1   H. sapiens      PLQMIYLVVKAAVGLVLP---AKLRDLSRENVLITGGGRGIGRQLAREFAERGARkiVLW  1p.0
SDR16C5   H. sapiens      LGKSLFSLLEAMIFALLP---KPRKNVAGEIVLITGAGSGLGRLLALQFARLGSV-LVLW
SDR16C2   H. sapiens      LPLLIVCSLESFVKLFIP---KRRKSVTGEIVLITGAGHGIGRLTAYEFAKLKSK-LVLW
SDR16C3   H. sapiens      LITIIYSYLESLVKFFIP---QRRKSVAGEIVLITGAGHGIGRQTTYEFAKRQSI-LVLW
SDR16C4   H. sapiens      TFKVLWAFVLAAARWLVR---PKEKSVAGQVCLITGAGSGLGRLFALEFARRRAL-LVLW
SDR16-1C1 C. intestinalis LLVSLYYCLEAIVMFFVP---TGRKVVRGQTVLITGAGSGigQRLSVEFAKLGCT-IVGV  1p.0
SDR16-1C1 S. purpuratus   LLSVLYYIGEGLVLCLVPTSF-RKNSIEGELVLITGAGSGIGRLQAIKFAAAGCD-VVLW
SDR16-1C2 S. purpuratus   IAKICFYNFVGFIDNIIPGSWKPQKDVSKEIVLVTGGGMGIGRLMSLTFAKLGAT-VIIW
SDR16-1C3 S. purpuratus   HLQVLLVSMEAFLKWLIPSSL-RSKSLEGETMLITGAGSGIGRLFARKFAAlgVR-VVLW  1p.2
SDR16 1C1 D. melanogaster IICCIGYILqd--LYYIAFGY-PEKELNTDIALITGGGNGLGRLLAERLGKMGTK-VVIW  1p.1
SDR16-1C1 A. gambiae     LLTSLGYILqa--FYYQIFGV-PKKNLNGEIALVTGGGGGLGRLLALRLIKLGAK-VVLW  1p.0
SDR16-1C1 A. mellifera   MGMALIYISEAIILTFIPKRY-RIKSVKGEIALVTGGAGGIGRLIAIKLAKLGAH-VVIW
SDR16-1C1 C. elegans     LFNLLIQNLISLIKYALPYSLLPKKDLYRKKVLITGAGNGLGKLLAQKFAARGAT-LILW
SDR16-1C2 C. elegans     TLHAICLLIISTIRNCFPMGWLLRKDVrgQTVLITGSGSGLGRLMAFEFGKLGAR-LVLW  1p.2
SDR16-1C3 C. elegans     VVGTLFFIVLNFFKNFLPNGVLPRKSVEGKKVLITGSGSGIGRLMALEFAKLGAE-VVIW
SDR16-1C1 A. californica LVNAIWLWLVAIYKALVPVSMQWTKDMSKDTVLVTGAGSGIGRLMSLKFAEMGCR-LVIW
SDR16-1C2 A. californica VRNIFAAFFLSWIRFFFS-G-YKKKSVTEEIVLITGAGSGMGQGMAMEFAKLGAV-VVLW
                                                        * **   * *

SDR16C1   H. sapiens      GRTEKCLKETTEEIRQ-------------------------MGTECHYFICDVGNREEVY
SDR16C2   H. sapiens      DINkhGLEETAAKCKG-------------------------LGAKVHTFVVDCSNREDIY  1p.0
SDR16C3   H. sapiens      DINkrGVEETAAECRK-------------------------LGVTAHAYVVDCSNREEIY  1p.0
SDR16C4   H. sapiens      DINTQSNEETAGMVRHIYRDLEAADAAALqaGNGEEEILPHCNLQVFTYTCDVGKRENVY  1p.2
SDR16C5   H. sapiens      DINKEGNEETCKMA-REA-----------------------GATRVHAYTCDCSQKEGVY
SDR16-1C1 C. intestinalis DISIVGLGETKKKLEDLN-----------------------MKVKYHCYKCDLSDREQIY
SDR16-1C1 S. purpuratus   DINTkgIEETAQLVRK-------------------------TGRKAWYCICDVSKREKVY  1p.2
SDR16-1C2 S. purpuratus   DINKETAQGVVKEIre-------------------------AGGKAYSYVVDCCDNEAVY  1p.1
SDR16-1C3 S. purpuratus   DINASDVEETAKLVRV-------------------------NGGKAWWYVCDVTEMAKVN
SDR16-1C1 D. melanogaster DINKkgIAETVQIVEE-------------------------AGGYCKGYVVDISKKEEVY  2p.2
SDR16-1C1 A. gambiae     DINQegLDDSVKLIQS-------------------------LGGLCKGYKVDISNKEEVY  2p.2
SDR16-1C1 A. mellifera   DINRIglESTVQEIRH-------------------------NGGKCWGYYCDITNKEEIY  1p.2
SDR16-1C1 C. elegans     DINLQSVDELKNEIRG-------------------------NQGeaHSYEVNLCDPGKIA  1p.0
SDR16-1C2 C. elegans     DINEQGNKETLKELEA-------------------------MGVeaKAYTVDLSEYKEIN  2p.0
SDR16-1C3 C. elegans     DVNKDGAEETKNQVVK-------------------------AGGKASTFVVDLSQYKDIH
SDR16-1C1 A. californica DINEEGNLETARQIEA-------------------------KGSKVKAYTVDLSNKESIY
SDR16-1C2 A. californica DVNNKGLEETVKMVKA-------------------------VEGVCHTYICDVsdRAAVR  1p.1
```

```
SDR16C1   H. sapiens      QTAKAVREkvGDITILVNNAAVVHGKSLMDSDDDALLKSQHINTLGQFwtTKAFLPRMLE  2p.0 3p.0
SDR16C2   H. sapiens      SSAKkvKAEIGDVSILVNNAGVVYTSDLFATQDPQIEKTFEVNVLAHFwtTKAFLPAMTK  2p.0 3p.0
SDR16C3   H. sapiens      RSLNqvKKEVGDVTIVVNNAGTVYPADLLSTKDEEITKTFEVNILGHFwiTKALLPSMME  2p.0 3p.0
SDR16C4   H. sapiens      LTAERVRKEVGEVSVLVNNAGVVSGHHLLECPDELIERTMMVNCHAHFwtTKAFLPTMLE       2p.0
SDR16C5   H. sapiens      RVADqvKKEVGDVSILINNAGIVTGKKFLDCPDELMEKSFDVNFKAHLwtYKAFLPAMIA  1p.0 2p.0
SDR16-1C1 C. intestinalis DVADkvKSDVGDVDILINNAGIVTGKRLMDCPDKLMIKTMDVNAVAHFwtIKAFLPSMLE  2p.0 3p.0
SDR16-1C1 S. purpuratus   EAAAKVKREAGEVTILVNNAGIIAGHKFINLTDDAIQKTMEVNALAHAwtLKAFLPDMMK       2p.0
SDR16-1C3 S. purpuratus   ETAQRVREEVGDVTMLVNNAGIVTGKYFQDLNEEDFHKTLNVNSLAHVwtLKAFLPNMLE       2p.0
SDR16-1C2 S. purpuratus   RTADKVREDIGHVTILINNAGIVSGKKLLQCPDSLIKKTMDLNINAHFwtLKAFMPHMLE       2p.0
SDR16-1C1 D. melanogaster KAADVIRDEVGdiTLLINNAGVVSGLHLLDTPDHLIERSFNVNVMAHFwtTKAFLPKMIE  3p.0 4p.0
SDR16-1C1 A. gambiae      KYAKIIQEEIGdvTLLFNNAGVVSGRALLDTPDHLIERSFSVNVLAHFwtTKAFLPAMLK  3p.0 4p.0
SDR16-1C1 A. mellifera    RMAKIVQIEVGsvTLLINNAGYVSGKIFWELSDVEIDRTYKVNILSHYwiNKTFLKDMMK  2p.0 3p.0
SDR16-1C1 C. elegans      QVGQQVINDIGKVDILVNNAGIAtaKMILDSSENEINRSFDVNVKAHFytVQQFLPAMLK  2p.2 3p.0
SDR16-1C2 C. elegans      RTADLVKSEVGKVDILVNNAGIVtgKKLLQCPDELMVKTVSVNTNALFftTKNFLPGMLE  3p.2 4p.0
SDR16-1C3 C. elegans      KVAKETKEAVGDIDILINNAGIVtgKKLFDCPDELMEKTMAVNTNALFytAKNFLPSMLE  1p.2 2p.0
SDR16-1C1 A. californica  ETAAkvKADVGNVDVLVNNAGIVTGRKFLDCPDGLVEKTMEVNTNAHFwtTKSFLPEMME  1p.0 2p.0
SDR16-1C2 A. californica  ETADKVRSEVGDVTMLVNSAGVVAGRRLLDLTDQQIEHTFGVNLMGPIwtTKEFLPSMLE       2p.0
                                    *      * *                      *              *


                                                 +   +
SDR16C1   H. sapiens      LQNGHIVCLNSVLALSAIPGAIDYCTSKASAFAFMESLTLGLL--DCPGVSATTVLPFHT
SDR16C2   H. sapiens      NNHGHIVTVASAAGHVSVPFLLAYcsSKFAAVGFHKTLTDELAALQITGVKTTCLCPNFV  4p.1
SDR16C3   H. sapiens      RNHGHIVTVASVCGHEGIPYLIPYcsSKFAAVGFHRGLTSELQALGKTGIKTSCLCPVFV  4p.1
SDR16C4   H. sapiens      INHGHIVTVASSLGLFSTAGVedYCASKFGVVGFHESLSHELKAAEKDGIKTTLVCPYLV  3p.0
SDR16C5   H. sapiens      NDHGHLVCISSSAGLSGVNGLadYCASKFAAFGFAESVFVETFVQKQKGIKTTIVCPFFI  3p.2
SDR16-1C1 C. intestinalis KNCGHIVTIASGAGVFGLPALLDYCAskFAAVGLSEALDLELWQQRKDGIHVTVVCPYYI  4p.1
SDR16-1C1 S. purpuratus   RDHGHIVTIASIMGEISAAGMSEYCMSKFASVGLHEAVLRETRAAGKLGINFTLVNPYMI
SDR16-1C2 S. purpuratus   KNHGHIVTIASLAGHLGVSGLVDYCASKFAAVGLDDALYHELQYSGKTGVKCTVVCPFYI
SDR16-1C3 S. purpuratus   RNHGHIVTVASIMGEIVVPGLSDYCMSKFAAVALHESLLREARAQGKDGVHFTLINPYKI
SDR16-1C1 D. melanogaster NDRGHIATIASLAGHVGISKLVDYCASKfaAVGFDEALRLELEVLGHTNIRTTCICPFFI  5p.0
SDR16-1C1 A. gambiae      NDHGHIITIASLAGHVGISKLVDYCSSKFAavGFDEALRLELEHLRAQGVFTTVICPYFI  5p.0
SDR16-1C1 A. mellifera    NNHGHIVTVASVAGLLGTYKCTDYSATKFAAIGYHESLFTELktHGYDGIHATLICPYFI  4p.0
SDR16-1C1 C. elegans      DNNGHIVTIASAAGKMGSSGlaDYSSTKHAAVGFHDSLVAEIMESEKNGVKTTLVCPYYV  4p.0
SDR16-1C2 C. elegans      SNKGHIVTIASMAGKCGVAGlvDYCASKHGAVGFNDSLASELYALK-KDVKTTVVCPIYI  5p.0
SDR16-1C3 C. elegans      KDNGHLVTIASMAGKTGCVGLVDYCASKHGAIGCHDSIAMEILAQKKYGVNTTLVCPFFI
SDR16-1C1 A. californica  RNHGHIVTIASSAGLFGVATLADYCASKFGAVGFDESIrsEMLKYRKTGVHTTVVCPFFI  3p.1
SDR16-1C2 A. californica  NNQGHIVNMASSCGLIGLSHLTDYsaSKFGVVGFTQTLNYEIHFSGHDGVHTTLVCPSFV  3p.1
                             **      *            *   *                            *

SDR16C1   H. sapiens      S-TEMFQGMRVrf--PNLFPPLKPETVARRTVEAVQLNQALLLLPWTMHALVILKsiLPQ  4p.1 5p.1
SDR16C2   H. sapiens      N-TGFIKNPSTsl-----GPTLEPEEVVNRLMHGILTEQKMIFIPSSIAFLTTLEriLPE  5p.1 6p.1
SDR16C3   H. sapiens      N-TGFTKNPSTrl-----WPVLETDEVVRSLIDGILTNKKMIFVPSYINIFLRLQkfLPE  5p.1 6p.1
SDR16C4   H. sapiens      D-TGMFRGCRIrkEIEPFLPPLKPDYCVKQAMKAILTDQPMICTPRLMYIVTFMKsiLPF  4p.1 5p.1
SDR16C5   H. sapiens      K-TGMFEGCTTgc--PSLLPILEPKYAVEKIVEAILQEKMYLYMPKLLYFMMFLKsfLPL  4p.1 5p.1
SDR16-1C1 C. intestinalis D-TGMFKGVSTpi-----LPILKPEYAVNRMVDAILKNKRMLLLPGFSYVMYALKgfLPS  5p.1 6p.1
SDR16-1C1 S. purpuratus   N-TGMFAGTKIry--EMIVPTLEPEYVAGKVVEAVQTRTALVRTPVLLHLMVLLKdiLPQ  3p.1 4p.1
SDR16-1C2 S. purpuratus   K-TGMFDGVKAkr--SIMIDLMEPEYAVEQIILAIRTNQRVLILPKSLYFFPGLKNLWHP  3p.1
SDR16-1C3 S. purpuratus   D-TGMFNGATIKKsaQLLFPTLKPEFVADKVVEAVQTNRDVVRTPFIFNMVVVLTkmLPL  3p.1 4p.1
SDR16-1C1 D. melanogaster QATGMFDDVNArw-----VPTLNPNDVADRVIAAIRKNEKLAVIPGFLKVLLSFKwtFPW  6p.1 7p.1
SDR16-1C1 A. gambiae      QSTGMFDDVNSrw-----VPTLDSNDVADKIIEGVQKNEKYVIIPGYLRLMLAIKwmFPW  6p.1 7p.1
SDR16-1C1 A. mellifera    N-TGMFHGVEPrl-----MPMLEPEYVAQEVVSGILLNQVVVVLPGSVRFLLPLKclLPA  5p.1 6p.1
SDR16-1C1 C. elegans      H-TSMFDATGAATrfPWIFPILDTDYVVQKIFEAIETEQEFLVTPRAFYLVFAGIqiLPY  5p.1 6p.1
SDR16-1C2 C. elegans      N-TGMFDGIATK--WPTLLPILSPEYVVDCIMEAVLTDRAFLAIPKFSYIFIALAglLPT       6p.1
SDR16-1C3 C. elegans      D-TGMFHGVTTkc--PALFPILEANYAVECIVEAILTNRPLLCMPKASYLILALIGLLPI  3p.1
SDR16-1C1 A. californica  K-TGMFEGAKSrf--PWLLPLLEPEYAADKIMDAVRTNQPMLCAPRVLYLLYFLRgvFPS  4p.1 5p.1
SDR16-1C2 A. californica  T-TGMFQGCRMaf--PLLIPELEQNATVQRIMHAILTNQSEVYIPRLVYFMNSLKtiIPV  4p.1 5p.1
```

```
                            *                                         *

SDR16C1   H. sapiens      AALEEIHKFSGT---YTCMNTFKGRT----------------------------------
SDR16C2   H. sapiens      RFLAVLKRKISV---K--FDAVIGYKMKAQ------------------------------
SDR16C3   H. sapiens      RASAILNRMQNI---Q--FEAVVGHKIKMK------------------------------
SDR16C4   H. sapiens      EAVVCMYRFLGA---DKCMYPFIAQRKQATNNNEA----KNGI-----------------
SDR16C5   H. sapiens      KTGLLIADYLGI---LHAMDGFVDQKKKL-------------------------------
SDR16-1C1 C. intestinalis KAMYVMGEACGI---NTSMDTFIGRKKE--------------------------------
SDR16-1C1 S. purpuratus   KAIFSMEDFFET---AKAMDNFVGRRHVKSS-----------------------------
SDR16-1C2 S. purpuratus   KAVQAAYDMNGI---QDMMNTFTGRTKED-------------------------------
SDR16-1C3 S. purpuratus   KANLLLEDFLEN---EKVMKNFVGRRGQKAVEQTG----AKIE-----------------
SDR16-1C1 D. melanogaster GCVGGLLKRLVPDASPHGLPSSIAVPTVPL---NSNSKLTAADIAALDAELSSVTLPAKP
SDR16-1C1 A. gambiae      GCNSGFLRRLVPDAAPQHTITPLNNS----------------------------------
SDR16-1C1 A. mellifera    KLCWALMYNIIR--GPQGMMMFKGREEVQVLKNNNNITMSKGTIH---------------
SDR16-1C1 C. elegans      KAQAMVAQFFGL---VQNLERFHK------------------------------------
SDR16-1C2 C. elegans      EVLNLYGDHFGI---THSMDHFKGRQSRQA------------------------------
SDR16-1C3 C. elegans      ESQVMMADFFGT---NESMNDFKGRQKND-------------------------------
SDR16-1C1 A. californica  TVADVLAEFIGI---SASMDDFVGRTKPKTA-----------------------------
SDR16-1C2 A. californica  SAMLEVIRFFRA---DKFMHSFVGRGKDELSLPAK----EE-------------------


SDR16C1   H. sapiens      ----------------
SDR16C2   H. sapiens      ----------------
SDR16C3   H. sapiens      ----------------
SDR16C4   H. sapiens      ----------------
SDR16C5   H. sapiens      ----------------
SDR16-1C1 C. intestinalis ----------------
SDR16-1C1 S. purpuratus   ----------------
SDR16-1C2 S. purpuratus   ----------------
SDR16-1C3 S. purpuratus   ----------------
SDR16-1C1 D. melanogaster PSMLIQRTPSLGERVL
SDR16-1C1 A. gambiae      ----------------
SDR16-1C1 A. mellifera    ----------------
SDR16-1C1 C. elegans      ----------------
SDR16-1C2 C. elegans      ----------------
SDR16-1C3 C. elegans      ----------------
SDR16-1C1 A. californica  ----------------
SDR16-1C2 A. californica  ----------------
```

Figure S30. Alignment of the invertebrate orthologs of the human SDR16C family variants. For further details see Fig. S25 and Table S32.

* symbol marks the position of identical amino acid residues of the aligned SDR protein sequences.

```
                                              ×   × ×
SDR21C1    H. sapiens       MSSGIHVALVTGGNKGIGLAIVRDLCRLF--SGDVVLTARDVTRGQAAVQQLQAEGLSPR
SDR21C2    H. sapiens       MSSCSRVALVTGANRGIGLAIARELCRQF--SGDVVLTARDVARGQAAVQQLQAEGLSPR
SDR21-1C1 C. intestinalis   -MSTTRVAIVSGSNRGLGLAIVRGLCKDF--KGDVYLCSRSEASGKEAVKSLETEGLCPK
SDR21-1C1 S. purpuratus     MSSRKTIAlvTGANKGIGFGIVRALCKELGERGVVYLASRDEGRGEKAVQELKGEGLNPR  1p.0
SDR21-1C1 M. rotundata      ---MTRVAvvTGGNKGIGFAIVKALCKQF--DGVVYLTARDVNRGQNAVKQLEDQGLTPK  1p.2
SDR21-1C2 A. californica    -MSLKRVAvvTGSNKGIGLAIVRGLCKDF--QGDVVLtaRDEGRGRAAVQVLEKEGFSPK  1p.0 2p.2
                                   * * * * * *  *   **      * * *  *    *  **  *   *  *

SDR21C1    H. sapiens       FHQLDIDDLQSIRALRDFLRKEYGGLDVLVNNAGIAFkvADPTPFHIQAEVTMKTNFFGT  1p.2
SDR21C2    H. sapiens       FHQLDIDDLQSIRALRDFLRKEYGGLNVLVNNAAVAFksDDPMPFDIKAEMTLKTNFFAT  1p.2
SDR21-1C1 C. intestinalis   YHQLDICDENSVLSLKEFLVKNYGGLDVLVNNAGFAYKSASTEPFGKQARDTVDVNYYGT
SDR21-1C1 S. purpuratus     CIQLDICNNDHISKVADYFRDTYGGLDILVNNAGIAFkaAATEPDSIQAPVTVETNVFAT  2p.0
SDR21-1C1 M. rotundata      FHQLDVTDENSISTFRDYLQKTYGGLDILVNNAAIAFkmAATEPFSVQAEETVRVNYFAL  2p.0
SDR21-1C2 A. californica    FHLLDISDHQSIVKLRDFLKETYQGLDVLVNNAAILYneDSTLPFPDQARETIKVNYYNN  3p.0
                               **                   * **   *****          *    *  *   *

SDR21C1    H. sapiens       RDVCTELLPLIKPqgRVVNVSSIMSVRALKSCSPELQQKFRSETITEEELVGLMNKFVED  2p.2
SDR21C2    H. sapiens       RNMCNELLPIMKPhgRVVNISSLQCLRAFENCSEDLQERFHSETLTEGDLVDLMKKFVED  2p.2
SDR21-1C1 C. intestinalis   LKISNILLPIMKKGGRVVNVSSFVSLMSIKKCSEELQSIFRSQTITEEELSSKMEEFVAH
SDR21-1C1 S. purpuratus     LRLCRALIPLIRSHGrvVTVASQAGSSIYGRLGPDLQKRFKTVT-SEQGVIDLMNEFIsa  3p.1 4p.1
SDR21-1C1 M. rotundata      RKVCTLLYPLLKPHARVVHVSSSSGRLSL-IPSESLRKRFSDPNLTEEELDNIMHEFVnt       3p.1
SDR21-1C2 A. californica    LDVCEVLFPLLRPHArvCNMSSLANAIAFKVLSPELRTKFLNPQITMPEVSSLMTQFVda  4p.1 5p.1
                                  * *       **    *             *   *             *  *
                                                 +   +
SDR21C1    H. sapiens       TKKGVHQKEGWP---SSAYGVTKIGVTVLSRIHARKLSEQRKGDKILLNACCPGWVRTDM
SDR21C2    H. sapiens       TKNEVHEREGWP---NSPYGVSKLGVTVLSRILARRLDEKRKADRILVNACCPGPVKTDM
SDR21-1C1 C. intestinalis   ARAGDHVTHGWP---DTAYGVSKVGVSVMTWIQARQMR-MRGLDDVLINACCPGWVRTDM
SDR21-1C1 S. purpuratus     AKEEKKKELGWG---SSNYGVSKLGVIALTRIQGQDIIKDSSREDILINscCPGYVDTDM  5p.0
SDR21-1C1 M. rotundata      AKTNTHLENGWS---NSAYVASKVGVSALARVHQKMFN-SDSREDLAVNAVHPGYVDTDM
SDR21-1C2 A. californica    AQENKHEEQGFFLSRVSSYGISKIGLTLMSAIQQRDFD-AQGAVDIVVNscSPGYVNTDM  6p.0
                                     *        *   * *                      *   ** * ***

SDR21C1    H. sapiens       AGPKATKSPEEGAETPVYLALLPPDAEGPHGQFVSEKRVEQW----
SDR21C2    H. sapiens       DGKDSIRTVEEGAETPVYLALLPPDATEPQGQLVHDKVVQNW----
SDR21-1C1 C. intestinalis   AGPKATKSPDEGAITPLYCALLPEGAKEPHGKFLSDKTIKEW----
SDR21-1C1 S. purpuratus     SSHKGPLTIDQGAVTPVYLALLPGGCSHQGLFFYQKAVKDFW----
SDR21-1C1 M. rotundata      TSHKGTLTPDQGAVAPVFAALLPENTDIKGKYIWFDKSLVEWTKDA
SDR21-1C2 A. californica    TQGKGPLTVEegAVTPLYLCLLPPDVSGPRGLFIREKAIYDWTTT-   7p.2
                                      **  *    ***                  *
```

Figure S31. Alignment of the invertebrate orthologs of the human SDR21C family variants. For further details see Fig. S25 and Table S33.

* symbol marks the position of identical amino acid residues of the aligned SDR protein sequences.

```
SDR25C1   H. sapiens      -----------------------------MLSAVARGY-----QGWFHPCA--RLSVRMS
SDR25C2   H. sapiens      ----------------------------------------MHKAGLLGLCARAWNSVRMA
SDR25C1C1 S. purpuratus   ----------------------------------------MFSLTRLLSVGRSINHFRMA
SDR25-1C1 D. melanogaster MLHLSKQLVVRAPKIRLNASAVSGSGQSSSFDQNSNNYSRKLVGPNLNQCHKRLSSSS-Q
SDR25-1C1 A. mellifera    ----------------------------------------MLRS----SINRSMQQIN-R
SDR25-1C1 C. elegans      ------------------------------------------------------------
SDR25-1C1 A. californica  ------------------------------------------------------------

                                            ×   × ×
SDR25C1   H. sapiens      STGIDRKGVLANRVAVVTGSTSgiGFAIARRLARDGAHVVISSRKQQNVDRAMAKLQGEG  1p.1
SDR25C2   H. sapiens      SSGMTRRDPLANKVALVTASTDgiGFAIARRLAQDGAHVVVSSRKQQNVDQAVATLQGEG  1p.1
SDR25-1C1 S. purpuratus   SAAATGSRRLEGKVAVVTASTEGIGYAIAKRLGEEGAHVVISSRKQAHVDHALKTLKEAN
SDR25-1C1 D. melanogaster SSTAGTMKRLAGKVAVVTASTDgiGFAIAKRLAEDGAAVVISSRKQKNVDSALAELRKLN  1p.1
SDR25-1C1 A. mellifera    NLSEIKYKRLEGKVAIVTASTQgiGFAIAKRLAEEGAKVMISSRKEENVQNALKELKSKN  1p.1
SDR25-1C1 C. elegans      --MPSNCRRFEGKVAIVTAATKgiGLAIAERLLDEGASVVIGSRNQKNVDEAIEYLKNKG  1p.1
SDR25-1C1 A. californica  -----MAGKLAGKVAIVTASTDgiGFAIARRLAQDGAKVMVSSRKQNNVDNAVKALKQEN  1p.1
                                       ** **  * *** *** **   ** *   **    *  *   *

SDR25C1   H. sapiens      LS-VAGIVCHVGKAEDREQLVAkaLEHCGGVDFLVCSAGVNPLVGSTLGTSEQIWDkiLS  2p.0        3p.0
SDR25C2   H. sapiens      LS-VTGTVCHVGKAEDRERLVAtaVKLHGGIDILVSNAAVNPFFGSIMDVTEEVWDktLD  2p.0        3p.0
SDR25-1C1 S. purpuratus   LS-VSGLVCHVGKQADRAKLIEtaVKENGGLDILVSNAAANPHFGNILdcDEQAWDkiFD  1p.0  2p.0  3p.0
SDR25-1C1 D. melanogaster LN-VHGLKCHVSEPEDRKQLFEETISKFGKLNILVSNAATNPAVGGVLECDEKVWDKIFD
SDR25-1C1 A. mellifera    LN-VCGMTCHVGKNEDRKSLLEktIQEFHGLDILVLNAGINPSASTFFETSESVWDKIFE  2p.0
SDR25-1C1 C. elegans      LTKVAGIAGHIASTDDQKKLVDftLQKFGKINILVNNHGINPAFGHILEVSDQVWDKLFE  2p.0
SDR25-1C1 A. californica  LN-VEGLVCHVAKKEDRSKLFNetVKKFGGIDVLVSNAASSTYFGPTLntPEDSYDkmFD  2p.0  3p.0  4p.0
                          *  * *   *     *   *             **                    **

                                                                    +   +
SDR25C1   H. sapiens      VNVKSPALLLSQLLPYMENrr-GAVILVSSIAAYNPVvaLGVYNVSKTALLGLTRTLALE  4p.1  5p.0
SDR25C2   H. sapiens      INVKAPALMTKAVVPEMEKRggGSVVIVSSIAAFSPSpgFSPYNVSKTALLGLTKTLAIE  4p.1  5p.0
SDR25-1C1 S. purpuratus   INVKSTFLLVKESVPHMVSRggGSIVVVSSIAGYMPFELLGPYSVSKTALLGLTKALTPQ  4p.1
SDR25-1C1 D. melanogaster VNVKSSYLLAKEALPLLRQQKNSSIVFVSSIAGYDAFelLGAYSVSKTALIGLTKAAAKD        2p.0
SDR25-1C1 A. mellifera    INVKSTFLLLRDSLPFLRKSKSASVILLSSIVGYSPFdmLGVYSISKTTILGINQVAANI        3p.0
SDR25-1C1 C. elegans      VNVKAGFQMTKLVHPHIAKEGGGAIIFNASYSAYKSPPGIAAYGVTKTTLVGLTRALAMG
SDR25-1C1 A. californica  LNVKSSFMLCKEVVPEMEKRgsGSMILVSSIAAYTPMsmIGIYSLTKTALLGLNKVLAPE  5p.1  6p.0
                           ***          *              *            *   **   *

SDR25C1   H. sapiens      LAPKDIRVNCVVPGIIKTDFSKvfHGNESLWKNF---KEHHQLQriGESEDCAGIVSFLC  6p.0  7p.1
SDR25C2   H. sapiens      LAPRNIRVNCLAPGLIKTSFSRmlWMDKEKEESM---KETLRIRrlGEPEDCAGIVSFLC  6p.0  7p.1
SDR25-1C1 S. purpuratus   LSDMNIRVNCVAPGLVKTKFSSalLSSEESTKLA---LSGIPMKRAGTPDEISGIVSFLA  5p.0
SDR25-1C1 D. melanogaster LAPEGIRVNCLAPGVIRTKFSKALYENESANEAA---LSKIPMGRLGTSEEMAGVVSFLV
SDR25-1C1 A. mellifera    LAPEGIRVNCIAPGIIKTKFSQilYENET-GEMM---LSKIPMNKFGKSDNIGSVAAFLA  4p.0
SDR25-1C1 C. elegans      LAKDNIRVNGIAPGVIKTKMSQvlWDGGEDAEKELTDIQEIALGRLGVPDDCAGTVAYLA  3p.0
SDR25-1C1 A. californica  LAAHNIRINCIAPGIIKTKFSNalTQNEDALNVA---LSTIPLGrlGTPEECSGAASFLA  7p.0  8p.1
                          *    ** *   **   *  *                         *           *

SDR25C1   H. sapiens      SPDASYVNGENIAVAGYSTRL-
SDR252C   H. sapiens      SEDASYITGETVVVGGGTPSRL
SDR25-1C1 S. purpuratus   SDDASYITGENILVAGGAPSRL
SDR25-1C1 D. melanogaster SEDAGYITGESIVAGGGMTARL
SDR25C1C1 C. elegans      SDDSSYITGEMIIIAGGVQARL
SDR25C1C1 A. mellifera    SDDASYITGETIVVAGGMRSRL
SDR25-1C1 A. californica  SDDSAYMTGETIVVSGGMTGRL
                          * *  *  **     *
```

Figure S32. Alignment of the invertebrate orthologs of the human SDR25C family variants. For further details see Fig. S25 and Table S34.

* symbol marks the position of identical amino acid residues of the aligned SDR protein sequences.

```
SDR26C1   H. sapiens     -----------------------MAFMKKYLLPILGLFMAYYYYSANE----------EF
SDR26C2   H. sapiens     --------------------------MKVLLLTGLGAL--FFAYYWDD----------NF
SDR26-1C2 C.intestinalis -----------------------MSYCFKALFVIIVA-IVTYSFIQPD----------DY
SDR26-1C4 C.intestinalis -----------------------MGWLRVTAVVVIA--CILYSAVRES----------GY
SDR26-1C1 S. purpuratus  MALGTIYLIALCIAAVFILLSIRVLYFIALFLLVTFLICFFLSMVSDGDLTLMLYELIGN

                                      ×   × ×
SDR26C1   H. sapiens     RpeMLQGKKVIVTGASKGIGREMAYHLAKMGAHVVVTARSKETLQkvVSHCLELGAASAH  1p.2 2p.0
SDR26C2   H. sapiens     DpaSLQGARVLLTGANAGVGEELAYHYARLGSHLVLTAHTEALLQkvVGNCRKLGAPKVF  1p.2 2p.0
SDR26-1C2 C.intestinalis DtgILKNKRVLITGASQNIGEELATQYAQSGAKIVITARREDKLKevAAKCRKAGATSVD  1p.2 2p.0
SDR26-1C4 C.intestinalis NpdEVKGLRVVITGASSGIGEQIAYKFASMGAKLHITARRESILQkvAARAKELGAIESH  1p.2 2p.0
SDR26-1C1 S. purpuratus  KgdNMMGRVVWITGASSGIGEELAYQLARkgAKVVLSARRVNELERVRGNCVRFPEVREE  1p.2 2p.0
                                  *  ***    *   *   *  *      *     *  *

SDR26C1   H. sapiens     ---YIAGTMEDMTFAEQFVAQAGKLmgGLDMLILNHITNTSLNLFHDD----IHHVRKSM  3p.2
SDR26C2   H. sapiens     ---YIAADMASPEAPESVVQFALDKlgGLDYLVLNHIGGAPAGTRARS----PQATRWLM  3p.2
SDR26-1C2 C.intestinalis ---YIVADMADIEQVKNVVKDAVALlgGLDQLVLNHVTGLNHPGMWNNSQFNLDYAQnsA  3p.2  4p.1
SDR26-1C4 C.intestinalis ---YLTISLSSYENSTLAVKDALEKlgGIDVLILNHFTNNYLGSYRHE----AKDIEKLF  3p.2
SDR26-1C1 S. purpuratus  NILVLPMDATKYETHGQVAETIVDRYGkiDILINNSGRSQRAL--VQ--DCKIEVDKAIM  3p.0
                                                   *  * *  *
                                                                 +   +
SDR26C1   H. sapiens     EVNFLSYVVLTVAALPMLKQ-SNGSIVVVSSLagKVAYPMVAAYSASKFALDGFFSSIRK       4p.2
SDR26C2   H. sapiens     qvNFVSYVQLTSRALPSLTD-SKGSLVVVSSLlgRVPTSFSTPYSAAKFALDGFFGSLRR_ 4p.0 5p.2
SDR26-1C2 C.intestinalis IVNYVSYIHLSTHAIPHLTE-SNGNLIVVSSLagHVPTINTAIYGATKASLNHFYASLRL       5p.2
SDR26-1C4 C.intestinalis MPNMLTHAHLASVAMESLIE-NKGRIAATSSAvaLLGGAFTVPYTTTKFALGGFFRSLEH       4p.2
SDR26-1C1 S. purpuratus  DLNLMGPISLFKVALPYMLENNYGQIINISSATGIMPAPLSSAYCASKHGMhgFFNSVRS  4p.0
                           *      *   *         *     **            *   *     *  *

SDR26C1   H. sapiens     EYSVS-RVNVSITLCVLGLIDteTAMKAVSGIVHMQAAPKEECA-LEI------------  5p.2
SDR26C2   H. sapiens     ELDVQ-DVNVAITMCVLGLRDRASAAEAVrsSTSRPRQPEHRGVPLQSQTAMFLPP----  6p.2
SDR26-1C2 C.intestinalis EMMASERDPYSITICMMGPIETDRSRWS-----KfpDKVNMKGNPIDDTAAQI-------  6p.1
SDR26-1C4 C.intestinalis ELTIN-KTGVSVSLIQLGYvaTNSSKAIAD-KIKATGASKIECASE------I-------  5p.2
SDR26-1C1 S. purpuratus  ELSYR---NIAVTMVCPGPVVSNIVENALREDFTrpAAKPYDGTADRSYKVLMSTERCAA  5p.0
                         *                *

SDR26C1   H. sapiens     --IKGGALR------------------------QEEVYYDSSLWTTLL--IRNPCRKILE
SDR26C2   H. sapiens     -TVPGARTLTETPLRGWPQPKMKSSRQKSKTEKNDGHLEPVTAWEVQVPRVRRLCRGLAR
SDR26-1C2 C.intestinalis --IkaGTTRR--------------------------SYLFIPWWTKYASAVKAIPSQLVD  7p.2
SDR26-1C4 C.intestinalis --VRGIMTR--------------------------EKLIYFPGYVSWIPLMRALFPNYVD
SDR26-1C1 S. purpuratus  LTVVAIANK---LNEVWVARQP------I--LLFYYMAQYMPGFTKwmTPLIG--RGRV-  6p.1

SDR26C1   H. sapiens     FLYSTSYNMDRFINK
SDR26C2   H. sapiens     P---HLFGHD-----
SDR26-1C2 C.intestinalis RMFLKIFYNK-----
SDR26-1C4 C.intestinalis KMTREAYYEGAFD--
SDR26-1C1 S. purpuratus  ----KQYNQSQAQ--
```

Figure S33. Alignment of the invertebrate orthologs of the human SDR26C family variants. For further details see Fig. S25 and Table S35.

* symbol marks the position of identical amino acid residues of the aligned SDR protein sequences.

```
SDR28C1   H. sapiens     ------------------------------------------------------------
SDR28C2   H. sapiens     ------------------------------------------------------------
SDR28-1C2 C.intestinalis MVYVVHLTDEVTKSFLSQKRATYRNMFTGELGRSVFGATVFTFVFALGVPWIGSTIFYIL
SDR28-1C1 S. purpuratus  ------------------------------------------------------------
SDR28-1C2 S. purpuratus  ------------------------------------------------------------
SDR28-1C3 S. purpuratus  ------------------------------------------------------------
SDR28-1C1 A. californica -------------------------------------------------------MMW--

                                                                            ×   × ×
SDR28C1   H. sapiens      -----------------------------------------MARTVVLITGCSSGIGLHL
SDR28C2   H. sapiens      -------------------MNGQSQVLPGGGHESREGINMAAAPRTVLISGCSSGIGLEL
SDR28-1C2 C. intestinalis GLNYgcCTSTIAFFA-------TC-FLALLIFAVFPRGRLNPKDKAVLITGCDSGFGPLL  1p.2
SDR28-1C1 S. purpuratus   -----------------------------------------MAPLVVLISGCSTGIGLAL
SDR28-1C2 S. purpuratus   -----------------------------------------MAPLVVLISGCSTGIGLAL
SDR28-1C3 S. purpuratus   -----------------------------------------MAPLITLITGCSTGIGLTT
SDR28-1C1 A. californica  DLLLPCGLALAVLLAVLFAFADTDFLLAL--YEKFGKDCGSLSGKVVWITGASSGIGEHI
                                                                          * *   * *

SDR28C1   H. sapiens      AVRLASDPSQSFkvYATLRDLKTQGRLWEAARA----LACPPGSLETLQLDVRDSKSVAA  1p.2
SDR28C2   H. sapiens      AVQLAHDPKKRYqvVATMRDLGKKETLEAAAGE----AL--GQTLTVAQLDVCSDESVAQ  1p.2
SDR28-1C2 C. intestinalis AKQLHALGMHVFagC-LLKDKGGEGAKHL--------MKIQSSRFHVLQLDVTNDSEIKM  2p.2
SDR28-1C1 S. purpuratus   AVRLAQDPDKKYLVYATMRNLAKKEGLEKAASD----AL--DKTLFVRQLDVTVDDQVKS
SDR28-1C2 S. purpuratus   AVRLAQDPDKKYLVYATMRNLAKKEAIEKAAGD----AL--DKTLFVRQLDVTVDDQVKT
SDR28-1C3 S. purpuratus   AVKMAKD-AKKYIVYATMRNLAKKGDLEQAAGS----AL--NDTLFVRQLDITEEESIVA
SDR28-1C1 A. californica  AYCLAKSGCRLVL---TARRVEELERVKQACISQGPNSAALEKNILVLPLDVVHFHKHRE
                          *                                                **

SDR28C1   H. sapiens      ARERV--T---EGRVDVlv-CNAGLGLLGPLEALGEDAVASVLDVNVVGTVRMLQAFLPD  2p.2
SDR28C2   H. sapiens      CLSCI------QGEVDVlv-NNAGMGLVGPLEGLSLAAMQNVFDTNFFGAVRLVKAVLPG  2p.2
SDR28-1C2 C. intestinalis AVQYIGMHIPPNQdgLWGIVNNAGASAFGEIEWSSIDLYKKVAEVNLWGVIRCTQACLPL  3p.2
SDR28-1C1 S. purpuratus   IFEFIMEK---HGRVDVli-NNAGFGFFGPLEAMSMEKAKNMFDTNYFGTVRLIRAALPI  1p.2
SDR28-1C2 S. purpuratus   IFEFIMGHGRVDV----lvKNNAGFGFFGALEAMSMEKAKNMFDTNYFGTVRLIRAALPI  1p.2
SDR28-1C3 S. purpuratus   IVKQIKEK----HGKIDILVNNAAFGWMGPLEEMTMTHMRNMSETNIVGTFRMTQEVLPP
SDR28-1C1 A. californica  AVQTVLEH----FGqiDILVNNAARGQFAEWIKVELDVDRELFETNVLGPVSLSQEVVPH  1p.0
                                               **                      *  *         *

                                                    +   +
SDR28C1   H. sapiens      MKRRGSGRVLVTGSVGGLmgLPFNDVYCASKFALEGLCESLAVLLLPFGVhlSLIECGPV  3p.2 4p.1
SDR28C2   H. sapiens      MKRRRQGHIVVISSVMGLqgVIFNDVYAASKFALEGFFESLAIQLLQFNIfiSLVEPGPV  3p.2 4p.1
SDR28-1C2 C. intestinalis I-RraKGRVVNMASGFGRMIAGSRSVYGITKWAVEGFSDCLRYEMRRWGVkvSIIEPGNY  4p.1 5p.0
SDR28-1C1 S. purpuratus   MKKQKSGRIVNISSMVGHlaLPYMDMYNASKFAMEGLSESLLPQLKNFGIsiSTVQPGPV  2p.2 3p.1
SDR28-1C2 S. purpuratus   MKKQKSGRIVNISSVVGHlaLPYMDMYNASKFAMEGLSESLLPQLKNFGIsiSTVQPGPV  2p.2 3p.1
SDR28-1C3 S. purpuratus   MKQQKSGRIVNISSLAGIngFPFSAVYSASKFALEGFTESLHPELKCFNIkiSTVCPGPV  1p.2 2p.1
SDR28-1C1 A. californica  MIKRGGGQIVVTSSMAGKmgLPGARSYTGSKHAIhgYFESLRTEMAGKNIEVTLICPGPT  2p.2 3p.0
                                *      *  *         *   * *  *    *                *

SDR28C1   H. sapiens      HTAFM------EKVLGSPE--EVLDRTDIHTFHRFYQ-YLAHSKQVFREAAQN-PEEVAe  5p.0
SDR28C2   H. sapiens      VTEFE------GKLLAQVSMAEFPG-TDPETLHYFRDLYLPASRKLFCSVGQN-PQDVVq  5p.0
SDR28-1C2 C. intestinalis IAGTSIFTEDGVKKLGDSMWEKMPDNVKEDYGKKLFDLrvAE-MQTYCSKGMDDLRPVLN  6p.0
SDR28-1C1 S. purpuratus   ATNFG------EALAANAGTEEDNKDVSEETVKQMTAFRLLL-HKPDLFAAQQ-TEPVID
SDR28-1C2 S. purpuratus   ATNFG------EALVANAGTEEDNKDVSEETVkqMTAFRLLL-SKPDLFTAQE-TGPVID  4p.0
SDR28-1C3 S. purpuratus   ITSFK------DNMSANQDPEAKSSDAENPTKklFTGAIAAM-MPHMKSAAQT-SDEIAD  3p.0
SDR28-1C1 A. californica  FSQLY------LTAATER-LGetLGLRMEKTQKRMSTS-----RCAYLSCVAM-ANQVSE  4p.0

SDR28C1   H. sapiens      vFL--------TALRAPKPTLRYFTTERFLPLLRMRLDDPSGSNYVTAMHREVFGDVPAK
SDR28C2   H. sapiens      aIV--------NVISSTRPPLRRQTNIRYSPLTTLKTVDSSGSLYVRTTHRLLFRCPRLL
SDR28-1C2 C. intestinalis AYE--DALLSRIPLIRYQPMEAYWTIRSFVFTHFPSFIADylY---------IYRKAKK-  7p.1
```

```
SDR28-1C1 S. purpuratus   VIV--------ECVEAEKPKLRYPTSESVRHLI---------------------------
SDR28-1C2 S. purpuratus   VIV--------ECVQAEKPKLRYPTSENVRSWVAKRYREEGVT-GLESLFE---------
SDR28-1C3 S. purpuratus   VII--------DCIKAEDPALRYGTNEGTSKRVTDRFGGGIGDAAAQNWYKMLEPASKI-
SDR28-1C1 A. californica  AWIMQQPVLLTAYIIQYAPVLGRWLLKKFGPKQVQKIREGK-------------------
                                            *

SDR28C1   H. sapiens      AEAGAE-AGGGAGPGAEDEAGRGAVGDPELGDPPAAPQ
SDR28C2   H. sapiens      -NLGLQCLSCGCLPTRVRPR------------------
SDR28-1C2 C. intestinalis --------------------------------------
SDR28-1C1 S. purpuratus   --------------------------------------
SDR28-1C2 S. purpuratus   --------------------------------------
SDR28-1C3 S. purpuratus   --------------------------------------
SDR28-1C1 A. californica  --------------------------------------
```

Figure S34. Alignment of the invertebrate orthologs of the human SDR28C family variants. For further details see Fig. S25 and Table S36.

* symbol marks the position of identical amino acid residues of the aligned SDR protein sequences.

```
SDR32C1   H. sapiens      MVSPATrkSLPKVKAMDFITS----TAILPLLFGCLG----VFGLFRLLQWVRGKAYLRN  1p.1
SDR32C2   H. sapiens      ----------------------MGVMAMLMLPLLLLGISGLLFI-YQEVSRLWSKSAVQN
SDR32-1C2 C. intestinalis ------------------------------------------------------MEEMKN
SDR32-1C1 S. purpuratus   ---------------MSSASSMNSLTVATAAIFGSLG----LLWLYKKLRSQRRAGLKD
SDR32-1C1 D. busckii      -------MKLHEMEKCAPSSDWNVLYWVIGTILMPIALPLALINIWQRFRAQNYRNQLPG
SDR32-1C1 A. gambiae      ------------MKNLAERSAGSLYWWLLATLFLPIAIPGLVLKLLTMMKEQRNARHLNG
SDR32-1C1 A. dorsata      -------------MTEESLKSWHLIWWLFKLFGFPITIPWLIYHFLDIMQQKRRKKTLNG
SDR32-1C1 H. robusta      ---------------------MSVLLF--SVGVGLLSVAGTV---YYFFCYKKCSSSLKG
SDR32-1C1 A. californica  -----------------MGIRANAVWAVLGLPFSLLS----VFYYIYVY-CCKEKLMLKD

                                ×   × ×
SDR32C1   H. sapiens      AVVVITGATSGLGkeCAKVFYAAGAKLVLCGRNGGALEELIRELTASHATkv-----QTH       2p.2 3p.0
SDR32C2   H. sapiens      KVVVITDAISGLGkeCARVFHTGGARLVLCGKNWERLENLYDALISVADPSkq-----TF       1p.2 2p.0
SDR32-1C2 C. intestinalis KVVLItgASSGIGEGLACAFARQGASLSLCGRNVENLKKVAEKCVSEGAAkt--------  1p.2      2p.0
SDR32-1C1 S. purpuratus   KVVLITGASSGVGeaCAIAFYRLGCKVILCARRAPELERVKKELMGPEigSRD---TSTH       1p.2 2p.2
SDR32-1C1 D. busckii      kvVLITGASSGLGESLAHVFYRAGCKVILAARRVQELERVKKDLLALD--vq-PA----Y  1p.0      2p.0
SDR32-1C1 A. gambiae      kvVLITGASSGLGEALAHSFFLAGCKVVLAARRKDELERVRKDLLELHatVPT------H  1p.0      2p.0
SDR32-1C1 A. dorsata      kvVIITGASSGLGEALAHVFYACGCKIILISRRKEELDRVKNTLMNTHvtVPT------Y  1p.0      2p.0
SDR32-1C1 H. robusta      KVVLIVGASSGIGeaCAHIFYKENCKLILCAKDLPDLERVKKEVEESYRKTDENCESTSE       1p.2
SDR32-1C1 A. californica  KVVFITGASSGLGaaCAEAFYTAGCRLVLSGRNVEKLNLLKVRMEKSKegTGF------H       1p.2 2p.0
                           ** *  * ** *   *  *        *       *

SDR32C1   H. sapiens      KPYLVTFDLTDSGAIVAAAAEILQCFGYVDILVNNAGISYRGTIMDTTVDVDKRVMETNY
SDR32C2   H. sapiens      TPKLVLLDLSDISCVPDVAKEVLDCYGCVDILINNASVKVKGPAHKISLELDKKIMDANY
SDR32-1C2 C. intestinalis --IQVVADVKNVKDMERMVDETVAKLGQIDVLINNAGYGCKGGIEngTLEQFDEVFSVNL  3p.0
SDR32-1C1 S. purpuratus   TPC-HTLRPRKTRRVTDKAKEALALHGHVDILINNGGMSGRGSVAETVLSVHQKIMNVNH
SDR32-1C1 D. busckii      PPTVLALDLSELNSIPQFAKRVLEVYNQVDILINNGGISVRADVVSTAVDVDLKVMIVNY
SDR32-1C1 A. gambiae      PPIILPLDLSDLNSIGGKVQSVLEIHGAIDILVNNGGISVRGDALSTAIDVDIRIMLVNY
SDR32-1C1 A. dorsata      PPVILPVDITNINNLQTEITKIIDIHGRIDILINNAGISYRGEIINTNMDVDIKVMLTNY
SDR32-1C1 H. robusta      EVTIFQLDISNLNETTKKISECIQIHGHIDILLGIAGISFRGKIIETELSVDQLIMSVNY
SDR32-1C1 A. californica  SPAIVTMDLEDLPSIKEKVKEVLAAFGHVDILINNAGKSYRGVALHTKLEVDKMLMDVNY
                                                       * *                           *

                                                                 +   +
SDR32C1   H. sapiens      FGPVALTkaLLPSMIKRRQGHIVAISSIQGKMSIPFRSayAASKHATQAFFDCLRAEMEQ  4p.2 5p.2
SDR32C2   H. sapiens      FGPITLTkaLLPNMISRRTGQIVLVNNIQGKFGIPFRTtyAASKHAALGFFDCLRAEVEE  3p.2 4p.2
SDR32-1C2 C. intestinalis KAPYYLMQLCLPHLK-KTkgCVINLSSCYTNMFVPDAVHYSMTKVGLDHLTKCAAieLGK  4p.2 5p.2
SDR32-1C1 S. purpuratus   FGSLVLTsaILPNMISRRSGHILAVSSIQGRIAIPFRSaySASKHAMQAFFDSLRAEVAE  3p.2 4p.2
SDR32-1C1 D. busckii      FGTVALTkaLLPSMIKRKNGHICFISSVQGKFAIPQRAAYSASKHAMQAFADSLRAEMAN  3p.2
SDR32-1C1 A. gambiae      FGSVALTKACLPSMMARKEGRIVSISSVQGKFAIPHRSAYSASKHAMQAFCDSLRAEVAK
SDR32-1C1 A. dorsata      FAQIALAkvILPYMIKQQSGHIVCISSIQGKISIPYrsAYAASKYALQAWCDCCRAELHD  3p.2 4p.1
SDR32-1C1 H. robusta      FGHVAATkaVLKSMVERRSGHIVYMSSLQGKLSIPFRSAysASKHAIQAFADCLRAEVAq  2p.2 3p.1 4p.2
SDR32-1C1 A. californica  FAHVEVTraILPQMVERKTGQIVAIGSVQSRIAIPHRTsyAASKHAMQAYFDVLRSEVAQ  3p.2 4p.2
                                    *        *              *    *   *            *

SDR32C1   H. sapiens      YEIEVTVISPGYIHTNLSVNAI-TADGSRYgvM-DTT---TAQGRSPVEVAQDVL-----  6p.2
SDR32C2   H. sapiens      YDVVISTVSPTFIRSYHVYPEQGNWEASIWkfF-FRK---LTYGVHPVEVAEEVM-----  5p.2
SDR32-1C2 C. intestinalis YGIRVNNVnpGLVVTEMVKANWKPDELEAFCKDFLSNTPLENESLSIQEVADVVIFLTSS  6p.0
SDR32-1C1 S. purpuratus   HNIQVTVLSPSYIKTNISLSAL-DGDGSVHakM-DST---TAGGMSTVYVADKVV-----  5p.2
SDR32-1C1 D. busckii      KNIYVSCVSPSYIRTQLSMNAL-TGAGSSYgkM-DET---TAKGMSPDKLAERIL-----  4p.2
SDR32-1C1 A. gambiae      DNIKVTLISPGYINTALSLNAL-TGTGASYgkM-DAA---TAGGASPQDTASSIL-----  3p.2
SDR32-1C1 A. dorsata      QNIKITIVSPGYIKTSLSLNAL-TGNGQIYgvM-DKT---IQEGYYPKYVAERIL-----  5p.2
SDR32-1C1 H. robusta      fNVRVSVVSPAYVRTNLSLNALTSSGSLHGkm--DEN---QKSGMSAEEVGQAVV-----  5p.2
```

```
SDR32-1C1 A. californica  YNIGVSVVNPYYIATNLSTNAV-SADGSAYgkL-DAN---TKAGLQPEYVASKVV-----  5p.2
                                   *

SDR32C1   H. sapiens      -------AAVGKKKKDVILADLLPSLAVYLRTLAPGLFFSLMASRARKERKSKNS--
SDR32C2   H. sapiens      -------RTVRRKKQEVFMANPIPKAAVYVRTFFPEFFFAVVACGVKEKLNVPEEG-
SDR32-1C2 C. intestinalis AAKSITGTCLPVEKGRILMGN------------------------------------
SDR32-1C1 S. purpuratus   -------DAVAKQQRDVVLGPFTHGAAIYIRALVPNLYFWIMAGRARKGTKMED---
SDR32-1C1 D. busckii      -------QCILRKEPDVIVSDLQAKIAYYLRHILPSAYFWIMAKRALKLERAEKKKD
SDR32-1C1 A. gambiae      -------KAIARDEKDVMLAPIAPRAAYWLRHLAPSVYFWIMKKRAEKLNST-----
SDR32-1C1 A. dorsata      -------KAVLKEEKDILITPFIPKAAMYLRTLCPSLYFWIMQKRAKNTKEKE----
SDR32-1C1 H. robusta      -------QAVLLDKKEVLLANCSYKMIVYFRNFLSDIYFMAMARRARSS--------
SDR32-1C1 A. californica  -------SCVVNRTSELTVAPPHIDLAIALRAMSPWLFFKIMERRAKKEQREMNKDR
```

Figure S35. Alignment of the invertebrate orthologs of the human SDR32C family variants. For further details see Fig. S25 and Table S37.

* symbol marks the position of identical amino acid residues of the aligned SDR protein sequences.

```
SDR42E1    H. sapiens       ------------------------------------------------------------
SDR42E2    H. sapiens       MKSNPPRSSLEACKAAGQGEKSCPVCQACGEVSGPRSGSGSESRPAPKPGAIPGPGLGPK
SDR42-1E1  C. intestinalis  ------------------------------------------------------------
SDR42-1E1  S. purpuratus    ------------------------------------------------------------
SDR42-1E2  C. elegans       ------------------------------------------------------------
SDR42-2E2  C. elegans       ------------------------------------------------------------
SDR42-1E2  C. remanei       ------------------------------------------------------------
SDR42-1E1  A. californica   ------------------------------------------------------------


SDR42E1    H. sapiens       ------------------------------------------------------------
SDR42E2    H. sapiens       AIPGPQAGSGTVPRPGAISGTGPGLGPGPGAGSVPGPGAGSVPGLGARSVPGPGAGSVPG
SDR42-1E1  C. intestinalis  ------------------------------------------------------------
SDR42-1E1  S. purpuratus    ------------------------------------------------------------
SDR42-1E2  C. elegans       ------------------------------------------------------------
SDR42-2E2  C. elegans       ------------------------------------------------------------
SDR42-1E2  C. remanei       ------------------------------------------------------------
SDR42-1E1  A. californica   ------------------------------------------------------------


SDR42E1    H. sapiens       ------------------------------------------------------------
SDR42E2    H. sapiens       PGAGSVPGPGAGSVPGPGAGSGPGLGGGLGPGVGAGPGAGSVPGPGAGSVPGPGAGSVPG
SDR42-1E1  C. intestinalis  ------------------------------------------------------------
SDR42-1E1  S. purpuratus    ------------------------------------------------------------
SDR42-1E2  C. elegans       ------------------------------------------------------------
SDR42-2E2  C. elegans       ------------------------------------------------------------
SDR42-1E2  C. remanei       ------------------------------------------------------------
SDR42-1E1  A. californica   ------------------------------------------------------------


SDR42E1    H. sapiens       -------------------------------------------------MDPKRSQKESV
SDR42E2    H. sapiens       PGAGSVPGAGAGSTPEPELGPGLRQGSGTGPRPSESTTTPTPAPQQKTQAKPTKAARQKV
SDR42-1E1  C. intestinalis  ----------------------------MDSVSDLTDTLGLPENFKLKTGTNGHsrRLKF  1p.1
SDR42-1E1  S. purpuratus    ------------------------------------------------------------
SDR42-1E2  C. elegans       -------------MSIKRLSMRLKKGIHRSWNRMTSLEAGLEEEKEIKIVEEpePRPWKV  1p.2
SDR42-2E2  C. elegans       --------------------------------------------------------MGHY
SDR42-1E2  C. remanei       --------------------------------------------------------MKHY
SDR42-1E1  A. californica   --------------------------------------------MVQTRSSAQSRDKEIH


                               ×  ×  ×
SDR42E1    H. sapiens       LITGGSGYFGFrlGCALNQNGVHVILFDISSPA----------------------Q---T  1p.1
SDR42E2    H. sapiens       LVTGGGGYLGFSLGSHLAKSGTSVILLDRRRPQ----------------------W---E
SDR42-1E1  C. intestinalis  LVTGGCGYFGYKLGKVLHTLGADVTLLDIQLPQ----------------------KIQES
SDR42-1E1  S. purpuratus    ------------------------------------------------------------
SDR42-1E2  C. elegans       LITGGAGHLAENLVAKLEEMTRDSIRPKIREMLEKEMPAVISTKVDKEVEKRLPMYIQIV
SDR42-2E2  C. elegans       VIVGGGGFLGAHVISALQKIGCKERIIVVDPCPQ-E---FKTIKID----KSNISY----
SDR42-1E2  C. remanei       VITGGAGLLGSQLISSLQKNGDFTKCVVIDPTPYRP---FDTIKLD----TSFVEYIQVS
SDR42-1E1  A. californica   VVTGGGGFPGFSLAKYLARKGHHVKLIDVKEPV----------------------W---D


SDR42E1    H. sapiens       ------------IPEGIKFIQGDIRHLSDVEKAFQDADVTCVFHIASYGMSGREQLN--R
SDR42E2    H. sapiens       ------------LSPETKFIqaDVRDEEALYRA--FEGVDCVFHVASYGMSGAEklQ--K  1p.0  2p.0
SDR42-1E1  C. intestinalis  ------------LDDHLKFIrgDIRDYDSVLKS--SAGMDCVYHIASFGMSGKDqlY--K  2p.0  3p.0
SDR42-1E1  S. purpuratus    ---------------MTKVQRGDIRDRELLDKL--CEGVDCVFHIASYGMSGREmmKA-S        1p.0
SDR42-1E2  C. elegans       LVDVLEPRGRVL-KHHVAFVkcSFDDECTMKTA--LEQVDTVYHLAAVGMTGQYarDR--  2p.0  3p.0
```

```
SDR42-2E2 C. elegans       -------------------IkaSFLDDKVLENI--LNGASAVVHLAAVGHTGLIagDR--  1p.0  2p.0
SDR42-1E2 C. remanei       --------SKIDSVSYTYSIQGSFLDETVLNRV--LSNCVTVFHLCAIGHTGRFgaQKYK        1p.0
SDR42-1E1 A. californica   ------------LEANMEFIKGSICDDNQLQSV--VEGASAVYHMASYGMSGREQLN--K
                                                                    * *    *  *

SDR42E1   H. sapiens       NLIKEVNVRGTDNILQVCQRRRVPRLVYTSTFNVIFGGQVIRNGDE-SLPYLPLHLPDH
SDR42E2   H. sapiens       EQIESINVGGTKLVIdvCVRRRVPRLIYTSTVNVAFGGKPIEQGDEDSVPYFPLDehVDH  3p.2  4p.0
SDR42-1E1 C. intestinalis  DLTEAINVDGTRNVIkaCQMNNVQRLIYTSSYNVVLEGNPIIGGDE-TLPYADPNkiVDY  4p.2  5p.0
SDR42-1E1 S. purpuratus    DFIESINVGGTENIVeaCIKQNVPRLVYTSTHNVVFAGQDIENGDE-TLPLLPLSahKDD  2p.2  3p.0
SDR42-1E2 C. elegans       KACMDINAVGTMNLLIWARNSGVQRFIYTSSVGVVFSGEPMYNATE-EVGYPD--dfYNY        4p.0
SDR42-2E2 C. elegans       KSVHNFNVNGTKQLIKQCKALGVKRFLYASSVAVSFIGEPLDNVTE-DDPLPDPKkyLDF        3p.0
SDR42-1E2 C. remanei       SRAHQFNVLGTIQLIKKCKENGVQRFIYSSSIAVVFTGKPLYSCNE-AEPYPKQSeyLDI        2p.0
SDR42-1E1 A. californica   KLIEKVNIGGTEAVLkaCLSQDVTRLVYTSTYNVVFGGQEILDGDE-RLPYLTDDkfTDH  1p.2  2p.0
                                 *  **           * *  * *   *   *       *

                           +   +
SDR42E1   H. sapiens       YSRTKSIAEQKVLEANATPLDRGDGVLRTCALRPAGIYGPGEQRHLPRIVSYIEKGLFKF
SDR42E2   H. sapiens       YSRTKAIADQLTLMANGMP-LpgGGTLRTCVLRPPGIYGPEEQRHLPRVagHIKKRLFMF  5p.2  6p.0
SDR42-1E1 C. intestinalis  YSKSKLKADKLILEANNTE-LndGRQLKTCCLRPAGIYGPGERRHMQRIakFIDWGIIIL  6p.2  7p.0
SDR42-1E1 S. purpuratus    YSRTKSMAEQLAMKSNGRETKkd-SILNVCVIRPVAIYGAGEQRHFPRIvkNMEQGLLCM  4p.2  5p.0
SDR42-1E2 C. elegans       YCESKAHAERIVQKASGHR-------MRTTVLRFNGIYGPGEKRVTERVvkFMLTGMWIA        5p.0
SDR42-2E2 C. elegans       YSASKAEAETYVLSQSTPD-------FKTVCLRFRGIYGPEDPNVTLKVanLIKNGLFIG        4p.0
SDR42-1E2 C. remanei       YSSTKAEAESFILSQSCLD-------FKTTCLRFRAIYGPQDVSVAEKVvnMVRKNLFMV        3p.0
SDR42-1E1 A. californica   YSKTKMLAEKKVLASNGAK-TSGGATLRCCVLRLAGVYGPGELRHLPRIvsYLEQGLVKV        3p.0
                           *   *  *                        *    **

SDR42E1   H. sapiens       VYG--DPRSLVEFVHVDNLVQAHILASEALRADKGHIASGQPYFISDGRPVNNFEFFRPL
SDR42E2   H. sapiens       RFG--DHKARMNWVHVHNLVQAHVLAAEALTTAKGYVasGQAYYINDGESVNLFEWMAPl  7p.0  8p.0
SDR42-1E1 C. intestinalis  RIG--D--AVVDWTHVNNLIQAHLLAIPGLSKTSGYVaaGKAYFISDNRPLRVFEFLRPl  8p.0  9p.0
SDR42-1E1 S. purpuratus    KIG--SSHIKVPWVHVDNLVNGHILAAEGLSSHKEHIaaGQVYFIADKAPVNQFEFLRPL  6p.0
SDR42-1E2 C. elegans       TCKPNGVEAQTQLSSVANCIQGLVKAELALRWSDT--PHGQIYNIMDKTPVGTFSFWTPL
SDR42-2E2 C. elegans       MVSAHGRESVSCASSGVNCAKAFALADQMLQNPDG--LHGraYYILDGENVGQFQFWTPL  5p.0
SDR42-1E2 C. remanei       KISRHDHESISNMSSAENCGQAFHLANQVLAEQNG--PHGqaYFITDGETVGQYEVWSPL  4p.0
SDR42-1E1 A. californica   TYG--PRESLVDFLHVDNLVQAHALAAEGLTEGRSYVaaGRAYFISDDKPVNNFEFFRPl  4p.0  5p.0
                                            *       *   *         *  * * *           **

SDR42E1   H. sapiens       VEGLGYTFPSTRLPLTLVYCFAFLTEMVHFILGRLYNFQPFLTRTEVYKTGVTHYFSLEK
SDR42E2   H. sapiens       fEKLGYSQPWIQVPTSWVYLtaAVMERLHLALRPICSLPPLLTRSevRSVAVTHTFQIAK  9p.2  10p.0
SDR42-1E1 C. intestinalis  fNGLGQEVPQYKCPYTIVYIlaILMEMLHYILKPVLVIEPLLTRNeiMKMGIDHHHKMDQ  10p.2 11p.0
SDR42-1E1 S. purpuratus    ITGLGYRYPSLIVPVWFMYIvaLLSEWLHTILKPVINFQPLMVRTelFQVAVTHHFSIEK  7p.2  8p.0
SDR42-1E2 C. elegans       NIALGFSSSMITVPATPIRLFAY-L---SQIIADRMRIDPIVSVLEVDLLLVNNTFNIEK
SDR42-2E2 C. elegans       VLALGKQPPSFYTPYDFIKAVVPYF---QNVCYGVFKLPPLLTKFELSILAVDNTYSIER
SDR42-1E2 C. remanei       IRALGKTPPVHSVPYPIVSAFVSIS---SFFCYEIFHSSPPMTRFELETLVTDNTYSIEK
SDR42-1E1 A. californica   fEGLGYGYPAVNLPFLPVFYfaWLTEIVHYIVSTVYNFQPILTRTevYKTGVTHYFSVQR  6p.2  7p.0
                              **        *                         *     *

SDR42E1   H. sapiens       AKKELGYKAQ-PFDLQEAVEWFKAHGHGRSSGSRDSE--CFVWDGLLVFLLIIAVL----
SDR42E2   H. sapiens       ARAQLGYAPD-KFRFADAVELYVQSTTRRPRGSTARTLLRLLLRLLLFLGLLALAL----
SDR42-1E1 C. intestinalis  AINDLGYKPM-FYDFADTVALYLKERGsvSEKSKI--AFHPLWTSVLFTIVTVIIT---- 12p.2
SDR42-1E1 S. purpuratus    AKKQLGYEPV-ERDLTDMVQYFLERGHRKKSSSIGA----FLFTVILAVLFVVLLM----
SDR42-1E2 C. elegans       AERDLGYEPS-VSAIPeiIEHYLHRLPPDVVRPKGRSD-FYVKVAVLVLGTILIFVAVFS  6p.0
SDR42-2E2 C. elegans       ARRELGYEPE-PCVMTdvAKYYQDLEENEVATA--TTE-WKSAISILIVFIS--------  6p.0
SDR42-1E2 C. remanei       AEKELGYVPG-KNHFKktVDYYRIQPIQSTTHN--FGS-WFNFDTLLFMILI--------  5p.0
SDR42-1E1 A. californica   ARTDLGYSPTVQNDLSQVVTYYKKLGRVKGHQSMPLL--FYFVNAIIIFIIYSIFM----
                           *   ***

SDR42E1   H. sapiens       ----MWLPSSVILSL----
```

```
SDR42E2   H. sapiens       ----HFLGLQPLHAAVERL
SDR42-1E1 C. intestinalis  ----ISLCL----------
SDR42-1E1 S. purpuratus    ----SLLPLAE--------
SDR42-1E2 C. elegans       FTFWMYLIFQRLSRWNP-F
SDR42-2E2 C. elegans       IAFLFLFNLN---------
SDR42-1E2 C. remanei       -IFWTIISF----------
SDR42-1E1 A. californica   ----HVLPRVTTD------
```

Figure S36. Alignment of the invertebrate orthologs of the human SDR42E family variants. For further details see Fig. S25 and Table S38.

* symbol marks the position of identical amino acid residues of the aligned SDR protein sequences.

Species	Gene symbol	Gene ID	Chr	Variant symbol	Exons n.	Phase formula	aa	Structure consensus	Catalytic consensus
Homo sapiens	RDH11	51109	14	SDR7C1	7	122221	318	GANTGIG	YCHSK
	RDH12	145226		SDR7C2			316		
	RDH13	112724	19	SDR7C3	7	122222	331		
	RDH14	57665	12	SDR7C4	2	0	336	GANSGLG	YSRSK
	DHRS13	147015	17	SDR7C5	5	2022	377	GANSGIG	YADTK
	DHRS12	79758	13	SDR40C1	10	120020022	317	GGNSGIG	YAQNK
Ciona intestinalis	LOC100177351	100177351	4	SDR7-1C1	6	22212	372	GATDGVG	YSRSK
	LOC100179350	100179350	3	SDR7-1C2	6	02021	313	GANAGIG	YCRSK
	LOC100176936	100176936	6	SDR7-3C2	7	100022	320	GANSGLG	YAQQK
	LOC100182258	100182258	7	SDR7-3C3	7	202021	321	GGNTGVG	YSNTK
	LOC100178007	100178007	6	SDR7-3C4	6	02021	322	GANTGIG	YGRSK
Strongylocentrotus purpuratus	LOC764557	764557	-	SDR7-1C1	5	2221	283	GCNTGIG	YGQSK
	LOC589120	589120	-	SDR7-1C3	4	222	355	GANTGIG	YARSK
	LOC585181	585181	-	SDR7-2C2	5	2221	357	GANAGIG	YNRSK
Musca domestica	LOC101892171	101892171	-	SDR7-1C3	6	10102	328	GSNTGIG	YNQSK
Brugia malayi	Bm1_24195	6099738	-	SDR7-1C1	8	2022001	340	GASAGIG	YSRSK
Aplysia californica	LOC101848777	101848777	-	SDR7-1C1	7	212222	382	GGTSGIG	YTRSK
	LOC101852273	101852273	-	SDR7-1C3	7	122221	344	GANCGIG	YGQSK

Table S27. Genetic and molecular data of the invertebrate orthologs of the human SDR7C family and of the SDR40C1 variants. Chr, chromosome; phase formula contains phase type symbols aligned according to the sequence of their relative splicing sites; aa n., number of the variant amino acids.

Species	Gene symbol	Gene ID:	Chr	Variant symbol	Exons n.	Phase formula	aa n.	Structure consensus	Catalysis consensus
Homo sapiens	BDH1	5622	3	SDR9C1	6	10022	343	GCDSGFG	YCITK
	HSD17B2	3294	16	SDR9C2	5	2222	387	GGDCGLG	YGSSK
	HSD11B2	3291		SDR9C3			405	GCDSGFG	YGTSK
	DHRS9	10170	2	SDR9C4	4	212	319	GCDSGFG	YTPSK
	RDH5	5959	12	SDR9C5			317		YCVSK
	HSD17B6	8630		SDR9C6					
	SDR9C7	121214		SDR9C7			313		
	RDH16	8608		SDR9C8			317		YCISK
Ciona intestinalis	LOC100175276	100175276	-	SDR9-1C2	5	2222	383	GCDIPNSYG	YSASK
Strongylocentrotus purpuratus	LOC581727	581727	-	SDR9-1C2	6	21212	327	GCDTGFG	YCESK
Caenorhabditis elegans	dhs-20	17959	V	SDR9-1C2	5	1012	342	GCDTGFG	YVVSK
	drd-5	186315	X	SDR9-1C4	7	200112	331	GCDSGFG	YCVSK
Aplysia californica	LOC101862944	101862944	-	SDR9-1C2	7	212222	394	GCDSGFG	YSMSK
	LOC101861767	101861767	-	SDR9-1C5	5	1212	322	GCDTGFG	YCTSK

Table S28. Genetic and molecular data of the invertebrate orthologs of the human SDR9C family variants. The atypical structure consensus of *Ciona intestinalis* is highlighted in turquoise. For further details see Table S27.

Species	Gene symbol	Gene ID	Chr	Variant symbol	Exons n.	Phase formula	aa n.	Structure consensus	Catalysis consensus
Homo sapiens	FAR1	84188	11	SDR10E1	11	0110012101	515	GATGFLG	YIYTK
	FAR2	55711	12	SDR10E2					YTYTK
Ciona intestinalis	LOC100177355	100177355	12	SDR10-1E1	12	0121022110	584	GATGFIG	YTLTK
	LOC100179698	100179698	12	SDR10-2E1	12	00121022110	573	GGTGFLG	YTLTK
Strongylocentrotus purpuratus	LOC584999	584999	-	SDR10-1E1	11	0010010012	644	GATGFIG	YTFTK
Drosophila melanogaster	CG5065	36860	2R	SDR10-1E1	9	00000021	625	GGTGFMG	YTFTK
Anopheles gambiae	AgaP_AGAP002279	1269279	2R	SDR10-1E1	4	111	543	GATGFMG	YAYTK
Apis mellifera	FAR1	551233	-	SDR10-1E1	5	0011	516	GGTGFMG	YTFTK
Caenorhabditis elegans	fard-1	180582	X	SDR10-1E1	11	0000010111	536	GGTGFLG	YTLTK
Aplysia californica	LOC101853321	101853321	-	SDR10-1E1	10	001221101	671	GGTGFVG	YTYTK

Table S29. Genetic and molecular data of the invertebrate orthologs of the human SDR10E family variants. For further details see Table S27.

Species	Gene Symbol	Gene ID	Chr	Variant symbol	Exons n.	Phase formula	aa n.	Structure consensus	Catalysis consensus
Homo sapiens	HSD3B1	3283	1	SDR11E1	3	22	375	GAGGFLG	YPHSK
	HSD3B2	3284		SDR11E2			372	GAGGLLG	YPYSK
	HSD3B7	80270	16	SDR11E3	6	22102	369	GGCGFLG	YPCSK
Strongylocentrotus purpuratus	LOC592634	592634	-	SDR11-1E1	3	22	366	GASGFLG	YATTK
Drosophila arizonae	LOC108612818	108612818	4	SDR11-1E1	3	22	380	GGSGFLG	YSSSK
Caenorhabditis briggsae	CBG02636	8572428	II	SDR11-1E1	7	221000	357	GGAGLVG	YPRNY

Table S30. Genetic and molecular data of the invertebrate orthologs of the human SDR11E family variants. For further details see Table S27. The atypical tyrosine (Y) of the *Caenorhabditis briggsae* is highlighted in turquoise.

Species	Gene Symbol	Gene ID	Chr	Variant symbol	Exons n.	Phase formula	aa n.	Structure consensus	Catalysis consensus
Homo sapiens	HSD17B12	51144	11	SDR12C1	11	2022001000	312	GSTDGIG	YSATK
	HSD17B3	3293	9	SDR12C2			310	GAGDGIG	YSASK
	HSDL1	83693	16	SDR12C3	4	100	330	GATDGIG	FSASK
Ciona intestinalis	LOC100179389	100179389	14	SDR12-1C1	6	21100	335	GCSSGIG	YSSTK
	LOC100176329	100176329	3	SDR12-2C1	7	202000	310	GCTSGIG	YGATK
Strongylocentrotus purpuratus	LOC583990	583990	-	SDR12-1C1	7	222000	356	GSTDGIG	YSACK
	LOC574750	574750	-	SDR12-2C1	7	001000	242	GASEGIG	YAGTK
Drosophila arizonae	LOC108610536	108610536	3	SDR12-1C1	5	2220	335	GATDGIG	YAASK
Drosophila ananassae	Dana\GF21190	6503875	-	SDR12-1C1	5	2020	318	GSTDGIG	YSSTK
Anopheles gambiae	AgaP_AGAP004532	1274678	2R	SDR12-1C1	4	202	329	GATDGIG	YAASK
	AgaP_AGAP008570	1275433	3R	SDR12-2C1	4	220	330	GSTDGIG	YAATK

	AgaP_AGAP007879	4578212	3R	SDR12-3C1	3	22	316	GASDGIG	YAASK
Apis mellifera	LOC410227	410227	-	SDR12-1C1	5	1220	359	GSTDGIG	YSATK
Caenorhabditis elegans	let-767	175895	III	SDR12-1C1	5	2200	337	GATDGIG	YSATK
Caenorhabditis remanei	CRE_05435	9824494	-	SDR12-1C1	4	200	215	GATDGIG	YSASK
Aplysia californica	LOC101847547	101847547	-	SDR12-1C1	9	20200000	315	GCTDGIG	YSGTK
	LOC101853549	101853549	-	SDR12-2C1	6	22200	361	GSSEGIG	YSATK

Table S31. Genetic and molecular data of the invertebrate orthologs of the human SDR12C family variants. The atypical phenylalanine (F) of the human catalysis consensus is highlighted in turquoise. For further details see Tables S27 and Consensuses in Online Resources 1.

Species	Gene Symbol	Gene ID	Chr	Variant symbol	Exons n.	Phase formula	aa n.	Structure consensus	Catalysis consensus
Homo sapiens	SDR16C1	9249	1	SDR16C1	6	00011	302	GGGRGIG	YCTSK
	SDR16C2	51170	4	SDR16C2	7	000111	300	GAGHGIG	YCSSK
	SDR16C3	345275		SDR16C3					
	SDR16C4	157506	8	SDR16C4	6	20011	341	GAGSGLG	YCASK
	SDR16C5	195814		SDR16C5		00211	309		
Ciona intestinalis	LOC100185899	100185899	-	SDR16-1C1	7	000111	304	GAGSGIG	YCASK
Strongylocentrotus purpuratus	LOC755823	755823	-	SDR16-1C1	5	2011	311	GAGSGIG	YCMSK
	LOC581999	581999	-	SDR16-1C2	4	101	311	GGGMGIG	YCASK
	LOC589689	589689	-	SDR16-1C3	5	2011	334	GAGSGIG	YCMSK
Drosophila melanogaster	CG9265	35369	2L	SDR16-1C1	8	1200011	399	GGGNGLG	YCASK
Anopheles gambiae	AgaP_AGAP010232	1279655	3R	SDR16-1C1	8	0200011	298	GGGGGLG	YCSSK
Apis mellifera	LOC552689	552689	-	SDR16-1C1	7	220011	326	GGAGGIG	YSATK
Caenorhabditis elegans	dhs-4	172810	I	SDR16-1C1	7	020011	305	GAGNGLG	YSSTK
	dhs-3	172603	I	SDR16-1C2	7	202001	307	GSGSGLG	YCASK
	dhs-19	179578	V	SDR16-1C3	4	201	307	GSGSGIG	YCASK
Aplysia californica	LOC101846838	101846838	-	SDR16-1C1	6	00111	309	GAGSGIG	YCASK
	LOC101853580	101853580	-	SDR16-1C2	6	10111	315	GAGSGMG	YSASK

Table S32. Genetic and molecular data of the invertebrate orthologs of the human SDR16C family variants. For further details see Table S27.

Species	Gene symbol	Gene ID	Chr	Variant symbol	Exons n.	Phase formula	aa n.	Structure consensus	Catalysis Consensus
Homo sapiens	CBR1	873	21	SDR21C1	3	22	277	GGNKGIG	YGVTK
	CBR3	874		SDR21C2				GANRGIG	YGVSK
Ciona intestinalis	LOC100182286	100182286	-	SDR21-1C1	1	-	275	GSNRGLG	YGVSK
Strongylocentrotus purpuratus	LOC755162	755162	-	SDR21-1C1	6	00110	278	GANKGIG	YGVSK
Megachile rotundata	LOC100877141	100877141	-	SDR21-1C1	4	201	276	GGNKGIG	YVASK
Aplysia californica	LOC101846277	101846277	-	SDR21-1C2	8	0201102	281	GSNKGIG	YGISK

Table S33. Genetic and molecular data of the invertebrate orthologs of the human SDR21C family variants. For further details see Table S27.

Species	Gene symbol	Gene ID	Chr	Variant symbol	Exons n.	Phase formula	aa n.	Structure consensus	Catalysis consensus
Homo sapiens	DHRS2	10202	14	SDR25C1	8	1001001	280	GSTSGIG	YNVSK
	DHRS4	10901		SDR25C2			278	ASTDGIG	
Strongylocentrotus purpuratus	LOC764565	764565	-	SDR25-1C1	6	00010	278	ASTEGIG	YSVSK
Drosophila melanogaster	CG10672	38598	3L	SDR25-1C1	3	10	317	ASTDGIG	YSVSK
Apis mellifera	DHRS4	412304	-	SDR25-1C1	5	1000	272	ASTQGIG	YSISK
Caenorhabditis elegans	dhrs-4	179772	V	SDR25-1C1	3	100	260	AATKGIG	YGVTK
Aplysia californica	LOC101862311	101862311	-	SDR25-1C1	8	2000100	253	ASTDGIG	YSLTK

Table S34. Genetic and molecular data of the invertebrate orthologs of the human SDR25C family variants. For further details see Table S27 and Consensuses in Online Resources 1.

Species	Gene symbol	Gene ID	Chr	Variant symbol	Exons n.	Phase formula	aa n.	Structure consensus	Catalysis Consensus
Homo sapiens	SDR26C1	3290	1	SDR26C1	6	20222	292	GASKGIG	YSASK
	SDR26C2	374875	19	SDR26C2	7	202022	315	GANAGVG	YSAAK
Ciona intestinalis	LOC100176102	100176102	7	SDR26-1C2	8	2021212	292	GASQNIG	YGATK
	LOC100179203	100179203	9	SDR26-1C4	6	20222	287	GASSGIG	YTTTK
Strongylocentrotus purpuratus	LOC580541	580541	-	SDR26-1C1	7	200001	348	GASSGIG	YCASK

Table S35. Genetic and molecular data of the invertebrate orthologs of the human SDR26C family variants. The atypical asparagine (N) of Ciona intestinalis structure consensus is highlighted in turquoise. For further details see Table S27.

Species	Gene symbol	Gene ID	Chr	Variant symbol	Exons n.	Phase formula	aa n.	Structure consensus	Catalysis Consensus
Homo sapiens	HSD17B1	3292	17	SDR28C1	6	22210	328	GCSSGIG	YCASK
	RDH8	50700	19	SDR28C2			331		YAASK
Ciona intestinalis	LOC100176396	100176396	1	SDR28-1C2	8	2221001	389	GCDSGFG	YGITK
Strongylocentrotus purpuratus	LOC762727	762727	-	SDR28-1C1	4	221	266	GCSTGIG	YNASK
	LOC592953	592953	-	SDR28-1C2	5	2210	283	GCSTGIG	YNASK
	LOC577205	577205	-	SDR28-1C3	4	210	291	GCSTGIG	YSASK
Aplysia californica	LOC101854946	101854946	-	SDR28-1C1	5	0200	322	GASSGIG	YTGSK

Table S36. Genetic and molecular data of the invertebrate orthologs of the human SDR28C family variants. For further details see Table S27.

Species	Gene symbol	Gene ID	Chr	Variant symbol	Exons n.	Phase formula	aa n.	Structure consensus	Catalysis consensus
Homo sapiens	DHRS7B	25979	17	SDR32C1	6	120222	325	GATSGLG	YAASK
	DHRS7C	201140		SDR32C2	11	20222	312	DAISGLG	
Ciona intestinalis	LOC104266214	104266214	11	SDR32C-1C2	7	200220	256	GASSGIG	YSMTK
Strongylocentrotus purpuratus	LOC588555	588555	-	SDR32C-1C1	6	22222	314	GASSGVG	YSASK
Drosophila busckii	LOC108603035	108603035	3R	SDR32C-1C1	5	0022	326	GASSGLG	YSASK
Anopheles gambiae	DHRS7_ANOGA	1276215	2L	SDR32C-1C1	4	002	317	GASSGLG	YSASK
Apis dorsata	LOC102677319	102677319	-	SDR32C-1C1	6	00212	317	GASSGLG	YAASK
Helobdella robusta	HELRODRAFT_ 194864	20213175	-	SDR32C-1C1	6	22122	306	GASSGIG	YSASK
Aplysia californica	LOC101858248	101858248	-	SDR32C-1C1	6	20222	312	GASSGLG	YAASK

Table S37. Genetic and molecular data of the invertebrate orthologs of the human SDR32C family variants. The atypical aspartate (N) of *Ciona intestinalis* structure consensus is highlighted in turquoise. For further details see Table S27 and Consensuses in Online Resources 1.

Species	Gene symbol	Gene ID	Chr	Variant symbol	Exons N.	Phase formula	aa n.	Structure consensus	Catalysis consensus
Homo Sapiens	SDR42E1	93517	16	SDR42E1	2	1	393	GGSGYFG	YSRTK
	SDR42E2	100288072		SDR42E2	12	0020200020	626	GGGGYLG	
Ciona intestinalis	LOC100186801	100186801	-	SDR42E-1E1	13	100202000202	406	GGCGYFG	YSKSK
Strongylocentrotus purpuratus	LOC575739	575739	-	SDR42E-1E1	9	02020020	334	-	YSRTK
Caenorhabditis elegans	hsd-1	189371	I	SDR42E-1E1	6	20000	462	GGAGHLA	YCESK
	hsd-2	191049	X	SDR42E-1E2	7	000000	374	GGGGFLG	YSASK
Caenorhabditis remanei	Cre-hsd-3	9824419	-	SDR42E-1E1	6	00000	390	GGAGLLG	YSSTK
Aplysia californica	LOC101864466	101864466	-	SDR42E-1E1	8	2000020	394	GGGGFPG	YSKTK

Table S38. Genetic and molecular data of the invertebrate orthologs of the human SDR42E family variants. The atypical (A) alanine of *Caenorhabditis elegans* structure consensus is highlighted in turquoise. For further details see Table S27.

Species	Variants	Phase formula	Splicing-site phases																																			
Homo sapiens	SDR7C1	122221				1		2										2					2							2									1
	SDR7C2					1		2										2					2							2									1
	SDR7C3	122222				1		2										2					2							2			2						
	SDR7C4	0																0																					
	SDR7C5	2022					2						0								2										2								
	SDR40C1	120020022		1				2						0					0				2			0		0				2							2
Ciona intestinalis	SDR7C-1C1	22212					2		2												2				1						2								
	SDR7C-1C2	02021									0										2				0					2									1
	SDR7C-3C2	100022		1										0												0											2		2
	SDR7C-3C3	202021					2								0						2						0				2				1				
	SDR7C-3C4	02021									0										2				0					2									1
Strongylocentrotus purpuratus	SDR7C-1C1	2221						2													2									2									1
	SDR7C-1C3	122221				1		2										2					2							2									1
	SDR7C-2C2	222					2														2										2								
Musca domestica	SDR7C-1C3	10102				1				0							1								0					2									
Brugia malayi	SDR7C-1C1	2022001			2								0					2					2						0							0			1
Aplysia californica	SDR7C-1C2	212222					2					1										2		2							2							2	
	SDR7C-1C3	122221	1					2										2					2							2									1

Table S39a. Splicing site organization of the human SDR7C family, of the SDR40C1 variants, and of their respective invertebrate orthologs. The phase type symbols are aligned according to the sequence of their relative splicing sites. Phase symbols, in a same column and highlighted in green or pink, mark the orthologous splicing sites. Note, this figure is identical to Table S2a in the main text.

Species	Variants	% identity					
		SDR7C1	SDR7C2	SDR7C3	SDR7C4	SDR7C5H	SDR40C1
Homo sapiens	SDR7C2	71.66					
	SDR7C3	49.20	48.89				
	SDR7C4	45.31	46.75	48.72			
	SDR7C5H	45.87	47.21	42.01	45.39		
	SDR40C1	30.20	31.00	28.05	28.72	26.33	
Ciona	SDR7C-1C1	37.26	38.78	34.46	36.31	35.62	26.40
	SDR7C-1C2	46.05	48.11	47.68	46.08	36.79	24.64
	SDR7C-3C2	26.00	27.48	25.25	26.51	25.83	54.57
	SDR7C-3C3	36.69	35.48	33.02	32.57	34.50	25.33
	SDR7C-3C4	46.25	46.93	44.73	46.91	39.54	23.67
Strongylocentrotus	SDR7C-1C1	54.12	53.76	53.36	50.53	44.04	30.71
	SDR7C-1C3	47.17	47.78	59.09	48.90	40.30	29.61
	SDR7C-2C2	42.49	42.77	42.86	42.44	45.02	27.63
Musca domestica	SDR7C-1C3	49.37	51.91	53.92	47.78	44.52	31.25
Brugia malayi	SDR7C-1C1	36.77	39.48	38.54	41.40	33.66	27.67
Aplysia californica	SDR7C-1C2	39.81	41.04	41.38	36.86	33.13	29.24
	SDR7C-1C3	49.05	46.47	52.05	47.34	40.26	28.57

Table S39b. Percent identity values of the human SDR7C family, of the SDR40C1 variants and of their invertebrate orthologs. Note, this figure is identical to Table S2b in the main text.

Species	Variants	Phase formula	Splicing-site phases																				
Homo sapiens	SDR9C1	10022		1			0				0			2		2							
	SDR9C2	2222							2					2				2			2		
	SDR9C3								2					2				2			2		
	SDR9C4	212												2					1				2
	SDR9C5													2					1				2
	SDR9C6													2					1				2
	SDR9C7													2					1				2
	SDR9C8													2					1				2
Ciona intestinalis	SDR9-1C2	2222							2					2				2		2			
Strongylocentrotus purpuratus	SDR9-1C2	21212	2					1						2					1				2
Caenorhabditis elegans	SDR9-1C2	1012				1						0							1				2
	SDR9-1C4	200112			2							0			0		1		1				2
Aplysia californica	SDR9-1C2	212222							2				1	2				2			2	2	
	SDR9-1C5	1212								1				2					1				2

Table S40a. Splicing site organization of the human SDR9C family variants and of their invertebrate orthologs. For further details see Table S39a.

Species	Variants	% identity							
		SDR9C1	SDR9C2	SDR9C3	SDR9C4	SDR9C5	SDR9C6	SDR9C7	SDR9C8
Homo sapiens	SDR9C2	31.50							
	SDR9C3	31.29	38.54						
	SDR9C4	36.33	30.57	30.03					
	SDR9C5	35.12	29.17	30.87	43.63				
	SDR9C6	38.67	31.09	31.83	44.90	49.37			
	SDR9C7	36.36	32.47	30.29	44.19	47.76	50.80		
	SDR9C8	37.67	32.05	32.48	46.18	51.58	66.25	51.76	
Ciona intestinalis	SDR9-1C2	34.63	32.66	31.21	28.28	29.31	35.07	31.94	32.99
Strongylocentrotus purpuratus	SDR9-1C2	35.76	31.76	28.62	43.65	41.37	43.32	40.92	46.25
Caenorhabditis elegans	SDR9-1C2	35.14	26.67	29.13	34.17	34.91	37.54	34.82	37.54
	SDR9-1C4	32.03	27.73	26.17	34.82	33.33	35.37	35.50	36.01
Aplysia californica	SDR9-1C2	35.89	31.72	32.89	34.50	34.41	34.08	33.88	33.12
	SDR9-1C5	34.54	30.89	27.16	34.49	36.59	39.12	38.98	39.43

Table S40b. Percent identity values of the human SDR9C family variants and of their invertebrate orthologs.

Species	Variants	Phase formula	Splicing-site phase																															
H. sapiens	SDR10E1	0110012101					0					1				1					0	0				1	2					1	0	1
	SDR10E2						0					1				1					0	0				1	2					1	0	1
Ciona intestinalis	SDR10-1E1	0121022110					0					1		2					1		0			2			2			1		1	0	1
	SDR10-2E1	00121022110		0			0					1		2					1		0				2		2			1		1	0	1
Strongylocentrotus purpuratus	SDR10-1E1	0010010012	0				0			1	0		0			1					0	0				1	2			1		1	0	1
Drosophila melanogaster	SDR10-1E1	00000021			0		0	0					0				0			0									2			1		1
Anopheles gambiae	SDR10-1E1	111					0											1														1		1
Apis mellifera	SDR10-1E1	0011					0						0										1											1
Caenorhabditis elegans	SDR10-1E1	0000010111				0			0				0		0		0									1		0				1		1
Aplysia californica	SDR10-1E1	001221101					0						0			1					0						2			1	1		0	1

Table S41a. Splicing site organization of the human SDR10E family variants and of their invertebrate orthologs. For further details see Table S39a.

Species	Variants	% identity	
		SDR10E1	SDR10E2
Homo sapiens	SDR10E2	59.03	
Ciona intestinalis	SDR10-1E1	37.74	34.63
	SDR10-2E1	35.58	34.36
Strongylocentrotus purpuratus	SDR10-1E1	52.72	47.47
Drosophila melanogaster	SDR10-1E1	38.82	38.43
Anopheles gambiae	SDR10-1E1	36.65	36.06
Caenorhabditis elegans	SDR10-1E1	38.13	34.05
Apis mellifera	SDR10-1E1	38.58	39.17
Aplysia californica	SDR10-1E1	53.79	48.54

Table S41b. Percent identity values of the human SDR10E family variants and of their invertebrate orthologs.

Species	Variants	Phase formula	Splicing site phases										
Homo sapiens	SDR11E1	22	2		2								
	SDR11E2		2		2								
	SDR11E3	22102	2		2	1		0			2		
Strongylocentrotus purpuratus	SDR11-1E1	22	2		2								
Musca domestica	SDR11-1E1	222	2		2						2		
Caenorhabditis briggsae	SDR11-1E1	221000	2		2		1			0		0	0

Table S42a. Splicing site organization of the human SDR11E family variants and of their invertebrate orthologs. For further details see Table S39a.

Species	Variants	% identity		
		SDR11E1	SDR11E2	SDR11E3
Homo sapiens	SDR11E2	93.55		
	SDR11E3	38.40	39.00	
Strongylocentrotus purpuratus	SDR11-1E1	32.03	32.87	33.98
Musca domestica	SDR11-1E1	26.98	26.85	26.04
Caenorhabditis briggsae	SDR11-1E1	18.21	19.10	19.52

Table S42b. Percent identity values of the human SDR11E family variants and of their invertebrate orthologs.

Species	Variants	Phase formula	Splicing-site phases																					
Homo sapiens	SDR12C1	2022001000			2		0			2	2		0		0	1		0		0				0
	SDR12C2				2		0			2	2		0		0	1		0		0				0
	SDR12C3	100				1												0						0
Ciona intestinalis	SDR12C-1C1	21100				2			1			1						0						0
	SDR12C-2C1	202000			2		0			2					0					0				0
Strongylocentrotus purpuratus	SDR12C-1C1	222000			2					2	2				0			0						0
	SDR12C-2C1	001000						0						0			2			0			0	0
Drosophila arizonae	SDR12C-1C1	2220			2					2	2													
Drosophila ananassae	SDR12C-1C1	2020			2		0			2								0						
Anopheles gambiae	SDR12C-1C1	202			2		0			2														
	SDR12C-2C1	220			2						2								0		0			
	SDR12C-3C1	22			2						2													
Apis mellifera	SDR12C-1C1	1220		1	2						2											0		
Caenorhabditis elegans	SDR12C-1C1	2200	2		2										0			0						
Caenorhabditis remanei	SDR12C-1C1	200			2										0			0						
Aplysia californica	SDR12C-1C1	20200000			2		0			2			0		0			0		0				0
	SDR12C-2C1	22200			2					2	2				0			0						0

Table S43a. Splicing site organization of the human SDR12C family variants and of their invertebrate orthologs. For further details see Table S39a.

Species	Variants	% identity		
		SDR12C1	SDR12C2	SDR12C3
Homo sapiens	SDR12C2	40.47		
	SDR12C3	41.02	34.25	
Ciona intestinalis	SDR12C-1C1	35.84	32.42	41.19
	SDR12C-2C1	37.67	33.33	34.14
Strongylocentrotus purpuratus	SDR12C-1C1	41.89	35.71	49.54
	SDR12C-2C1	27.23	27.43	19.67
Drosophila arizonae	SDR12C-1C1	37.79	32.55	39.56
Drosophila ananassae	SDR12C-1C1	46.86	38.93	38.74
Anopheles gambiae	SDR12C-1C1	44.33	34.68	39.00
	SDR12C-2C1	37.25	29.67	39.03
	SDR12C-3C1	31.97	30.54	37.00
Apis mellifera	SDR12C-1C1	37.75	33.78	42.86
Caenorhabditis elegans	SDR12C-1C1	39.46	31.44	32.48
Caenorhabditis remanei	SDR12C-1C1	35.40	34.01	34.01
Aplysia californica	SDR12C-1C1	45.54	36.33	38.13
	SDR12C-2C1	33.00	27.27	41.51

Table S43b. Percent identity values of the human SDR12C family variants and of their invertebrate orthologs.

Species	Variants	Phase formula	Splicing-site phases																											
Homo sapiens	SDR16C1	00011					0									0			0									1		1
	SDR16C2	000111						0							0				0			1						1		1
	SDR16C3							0							0				0			1						1		1
	SDR16C4	20011										2							0		0							1		1
	SDR16C5	00211													0				0		2							1		1
Ciona intestinalis	SDR16-1C1	000111			0										0				0				1					1		1
Strongylocentrotus purpuratus	SDR16-1C1	2011							2										0									1		1
	SDR16-1C2	101									1								0									1		
	SDR16-1C3	2011				2													0										1	1
Drosophila melanogaster	SDR16-1C1	1200011	1						2								0		0					0				1		1
Anopheles gambiae	SDR16-1C1	0200011	0						2								0		0						0			1		1
Apis mellifera	SDR16-1C1	220011								2							2		0								0	1		1
Caenorhabditis elegans	SDR16-1C1	020011											0					2	0	0									1	1
	SDR16-1C2	202001		2									0					2	0	0										1
	SDR16-1C3	201																2	0									1		
Aplysia californica	SDR16-1C1	00111													0				0							1		1		1
	SDR16-1C2	10111												1					0			1						1		1

Table S44a. Splicing site organization of the human SDR16C family variants and of their invertebrate orthologs. For further details see Table S39a.

Species	Variants	% Identity				
Homo sapiens		SDR16C1	SDR16C2	SDR16C3	SDR16C4	SDR16C5
	SDR16C2	33.67				
	SDR16C3	32.31	63.67			
	SDR16C4	36.45	40.67	39.67		
	SDR16C5	34.67	41.95	40.27	45.25	
Ciona intestinalis	SDR16-1C1	37.92	42.42	41.08	42.05	48.84
Strongylocentrotus purpuratus	SDR16-1C1	37.67	40.13	38.13	42.95	40.98
	SDR16-1C2	33.22	37.46	37.79	38.16	43.97
	SDR16-1C3	38.67	38.80	38.46	42.54	41.18
Drosophila melanogaster	SDR16-1C1	32.20	38.72	36.36	35.41	38.54
Apis mellifera	SDR16-1C1	34.14	40.48	39.46	37.05	38.85
Anophele gambiae	SDR16-1C1	33.22	41.52	37.02	35.40	42.07
Caenorhabditis elegans	SDR16-1C1	30.20	35.71	35.71	36.54	38.46
	SDR16-1C2	37.16	40.68	40.34	43.67	47.68
	SDR16-1C3	35.35	39.32	35.93	40.33	48.18

Table S44b. Percent identity values of the human SDR16C family variants and of their invertebrate orthologs.

Species	Variants	Phase formula	Splicing-site phases							
Homo sapiens	SDR21C1	22			2	2				
	SDR21C2				2	2				
Ciona intestinalis	SDR21C-1C1	-	No splicing sites							
Strongylocentrotus purpuratus	SDR21C-1C1	00110	0		0		1	1	0	
Megachile rotundata	SDR21C-1C1	201	2		0			1		
Aplysia californica	SDR21C-1C2	0201102	0	2	0		1	1	0	2

Table S45a. Splicing site organization of the human SDR21C family variants and of their invertebrate orthologs. For further details see Table S39a.

Specie	Variants	% identity	
Homo sapiens		SDR21C1	SDR21C2
	SDR21C2	71.84	
Ciona intestinalis	SDR21C-1C1	55.64	49.45
Strongylocentrotus purpuratus	SDR21C-1C1	46.01	44.20
Megachile rotundata	SDR21C-1C1	48.16	45.96
Aplysia californica	SDR21C-1C2	50.91	48.73

Table S45b. Percent identity values of the human SDR21C family variants and of their invertebrate orthologs.

Species	Variants	Phase formula	Splicing-site phases									
Homo sapiens	SDR25C1	1001001		1	0		0	1		0	0	1
	SDR25C2			1	0		0		1	0	0	1
Strongylocentrotus purpuratus	SDR25-1C1	00010			0	0	0		1		0	
Drosophila melanogaster	SDR25-1C1	10		1						0		
Apis mellifera	SDR25-1C1	1000		1	0					0	0	
Caenorhabditis elegans	SDR25-1C1	100		1	0						0	
Aplysia californica	SDR25-1C1	2000100	2		0	0	0		1	0	0	1

Table S46a. Splicing site organization of the human SDR25C family variants and of their invertebrate orthologs. For further details see Table S39a.

Species	Variants	% Identity	
Homo sapiens		SDR25C1	SDR25C2
	SDR25C2	59.78	
Strongylocentrotus purpuratus	SDR25-1C1	47.97	56.12
Drosophila melanogaster	SDR25-1C1	46.59	49.46
Apis mellifera	SDR25-1C1	44.15	43.38
Caenorhabditis elegans	SDR25-1C1	40.55	46.09
Aplysia californica	SDR25-1C1	49.80	56.92

Table S46b. Percent identity values of the human SDR25C family variants and of their invertebrate orthologs.

Species	Variants	Phase formula	Splicing-site phases														
Homo sapiens	SDR26C1	20222	2		0	2				2			2				
	SDR26C2	202022	2		0	2			0	2				2			
Ciona intestinalis	SDR26-1C2	2021212	2		0	2		1		2					1	2	
	SDR26-1C4	20222	2		0	2				2		2					
Strongylocentrotus purpuratus	SDR26-1C1	200001	2	0			0				0				0		1

Table S47a. Splicing site organization of the human SDR26C family variants and of their invertebrate orthologs. For further details see Table S39a.

Species	Variants	% identity	
Homo sapiens		SDR26C1	SDR26C2
	SDR26C2	41.22	
Ciona intestinalis	SDR26-1C2	32.37	34.41
	SDR26-1C4	28.62	28.26
Strongylocentrotus purpuratus	SDR26-1C1	24.10	25.08

Table S47b. Percent identity values of the human SDR26C family variants and of their invertebrate orthologs.

Species	Variants	Phase formula	Splicing-site phases													
Homo sapiens	SDR28C1	22210		2			2		2		1				0	
	SDR28C2			2			2		2		1				0	
Ciona intestinalis	SDR28-1C2	2221001	2	2	2			1			0			0		1
Strongylocentrotus purpuratus	SDR28-1C1	221					2		2		1					
	SDR28-1C2	2210					2		2		1		0			
	SDR28-1C3	210							2		1		0			
Aplysia californica	SDR28-1C1	0200				0			2	0		0				

Table S48a. Splicing site organization of the human SDR28C family variants and of their invertebrate orthologs. For further details see Table S39a.

Species	Variants	% identity	
Homo sapiens		SDR28C1	SDR28C2
	SDR28C2	44.59	
Ciona intestinalis	SDR28-1C2	25.91	23.71
Strongylocentrotus purpuratus	SDR28-1C1	42.53	47.71
	SDR28-1C2	39.21	44.80
	SDR28-1C3	35.31	40.42
Aplysia californica	SDR28-1C1	27.76	26.95

Table S48b. Percent identity values of the human SDR28C family variants and of their invertebrate orthologs.

Species	Variants	Phase formula	Splicing-site phases																
Homo sapiens	SDR32C1	120222	1			2		0			2			2					2
	SDR32C2	20222				2			0		2			2					2
Ciona intestinalis	SDR32-1C2	200220			2			0		0		2				2		0	
Strongylocentrotus purpuratus	SDR32-1C1	22222				2	2				2			2					2
Drosophila busckii	SDR32-1C1	0022		0				0			2								2
Anopheles gambiae	SDR32-1C1	002		0			0												2
Apis dorsata	SDR32-1C1	00212		0			0				2		1						2
Helobdella robusta	SDR32-1C1	22122				2					2				1		2		2
Aplysia californica	SDR32-1C1	20222				2	0				2			2					2

Table S49a. Splicing site organization of the human SDR32C family variants and of their invertebrate orthologs. For further details see Table S39a.

Species	Variants	% Identity	
Homo sapiens		SDR32C1	SDR32C2
	SDR32C2	43.19	
Ciona intestinalis	SDR32-1C2	26.36	24.58
Strongylocentrotus purpuratus	SDR32-1C1	46.43	33.66
Drosophila busckii	SDR32-1C1	43.49	32.35
Anopheles gambiae	SDR32-1C1	47.08	30.69
Apis dorsata	SDR32-1C1	44.81	30.26
Helobdella robusta	SDR32-1C1	43.34	31.67
Aplysia californica	SDR32-1C1	45.90	37.09

Table S49b. Percent identity values of the human SDR32C family variants and of their invertebrate orthologs.

Species	Variants	Phase formula	Splicing-site phases															
Homo sapiens	SDR42E1	1			1													
	SDR42E2	0020200020				0	0	2	0	2	0	0		0	2	0		
Ciona intestinalis	SDR42-1E1	100202000202		1		0	0	2	0	2	0	0		0	2	0		2
Strongylocentrotus purpuratus	SDR42-1E1	02020020					0	2	0	2	0	0			2	0		
Caenorhabditis elegans	SDR42-1E1	20000	2			0	0		0		0						0	
	SDR42-2E1	000000				0	0		0		0		0				0	
Caenorhabditis remanei	SDR42-1E2	00000					0		0		0		0				0	
Aplysia californica	SDR42-1E1	2000020						2	0		0	0		0	2	0		

Table S50a. Splicing site organization of the human SDR42E family variants and of their invertebrate orthologs. For further details see Table S39a.

Species	Variants	% identity	
Homo sapiens		SDR42E1	SDR42E2
		47.18	
Ciona intestinalis	SDR42-1E1	41.58	40.69
Strongylocentrotus purpuratus	SDR42-1E1	49.55	43.88
Caenorhabditis elegans	SDR42-1E1	27.01	27.54
	SDR42-2E1	24.72	29.46
Caenorhabditis remanei	SDR42-1E2	28.33	31.58
Aplysia californica	SDR42-1E1	48.97	39.69

Table S50b. Percent identity values of the human SDR42E family variants and of their invertebrate orthologs. Note, this figure is identical to Table S4b in the main text.
