# Supplementary material for: Gene Structure Evolution of the Short-Chain Dehydrogenase/Reductase (SDR) Family
Source: Genes (Basel). 2022 Dec 30;14(1):110. doi: 10.3390/genes14010110 (PMC9859523; doi:10.3390/genes14010110)
Supplement: Supplementary file 1 [file genes-14-00110-s001.zip › Online Resources 5/Online Resources 5.pdf]

PHYLOGENETIC ANALYSES

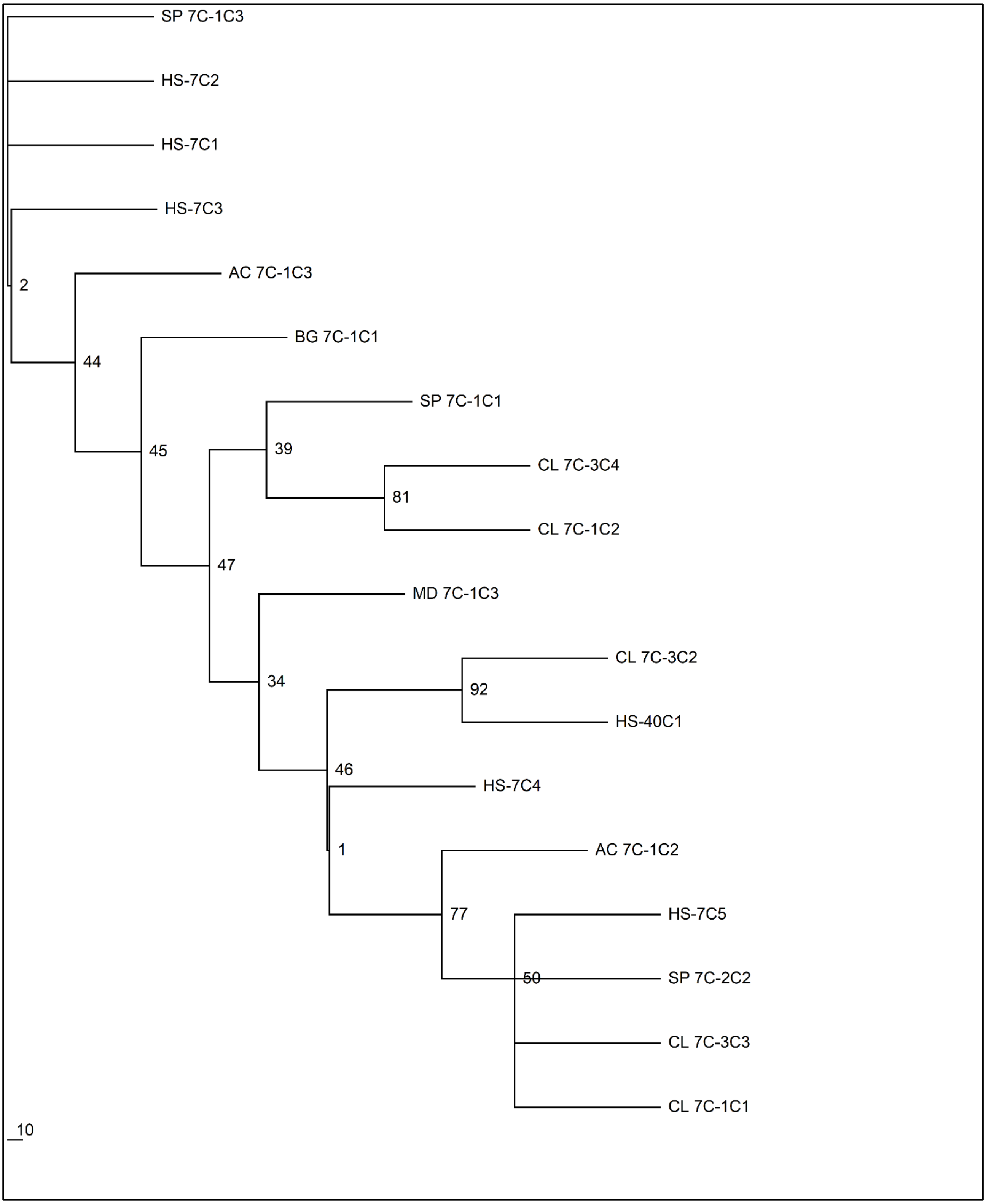

Figure S37. Phylogram of SDR7C gene structures. Splicing-site phase data are from Table S39a (Online Resources 4). *Homo sapiens* (HS), *Ciona intestinalis* (CL), *Strongylocentrotus purpuratus* (SP), *Musca domestica* (MD), *Brugia malayi* (BG), *Aplysia californica* (AC).

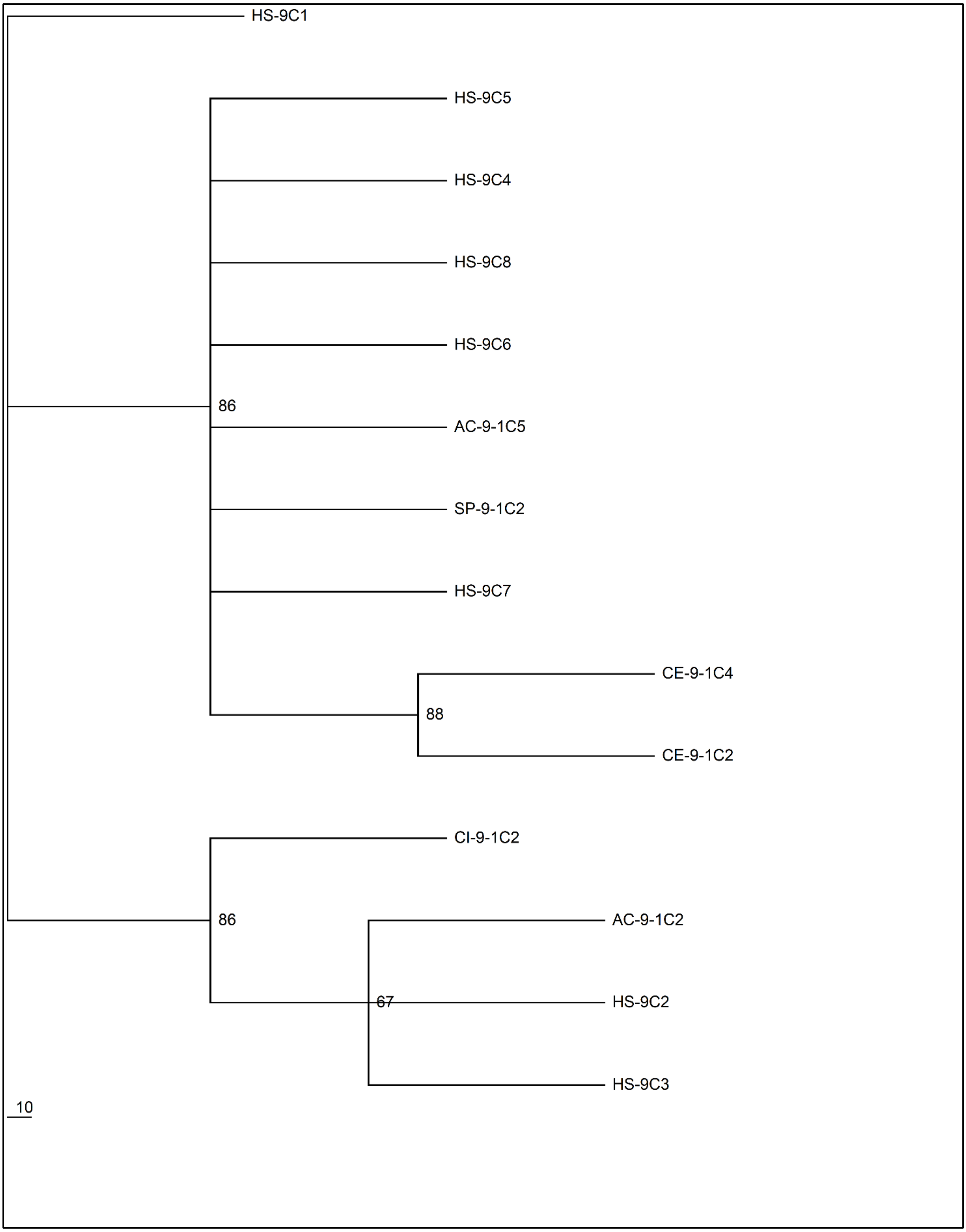

Figure S38. Phylogram of SDR9C gene structures. Splicing-site phase data are from Table S40a (Online Resources 4). *Homo sapiens* (HS), *Ciona intestinalis* (CI), *Strongylocentrotus purpuratus* (SP), *Caenorhabditis elegans* (CE), *Aplysia californica* (AC).

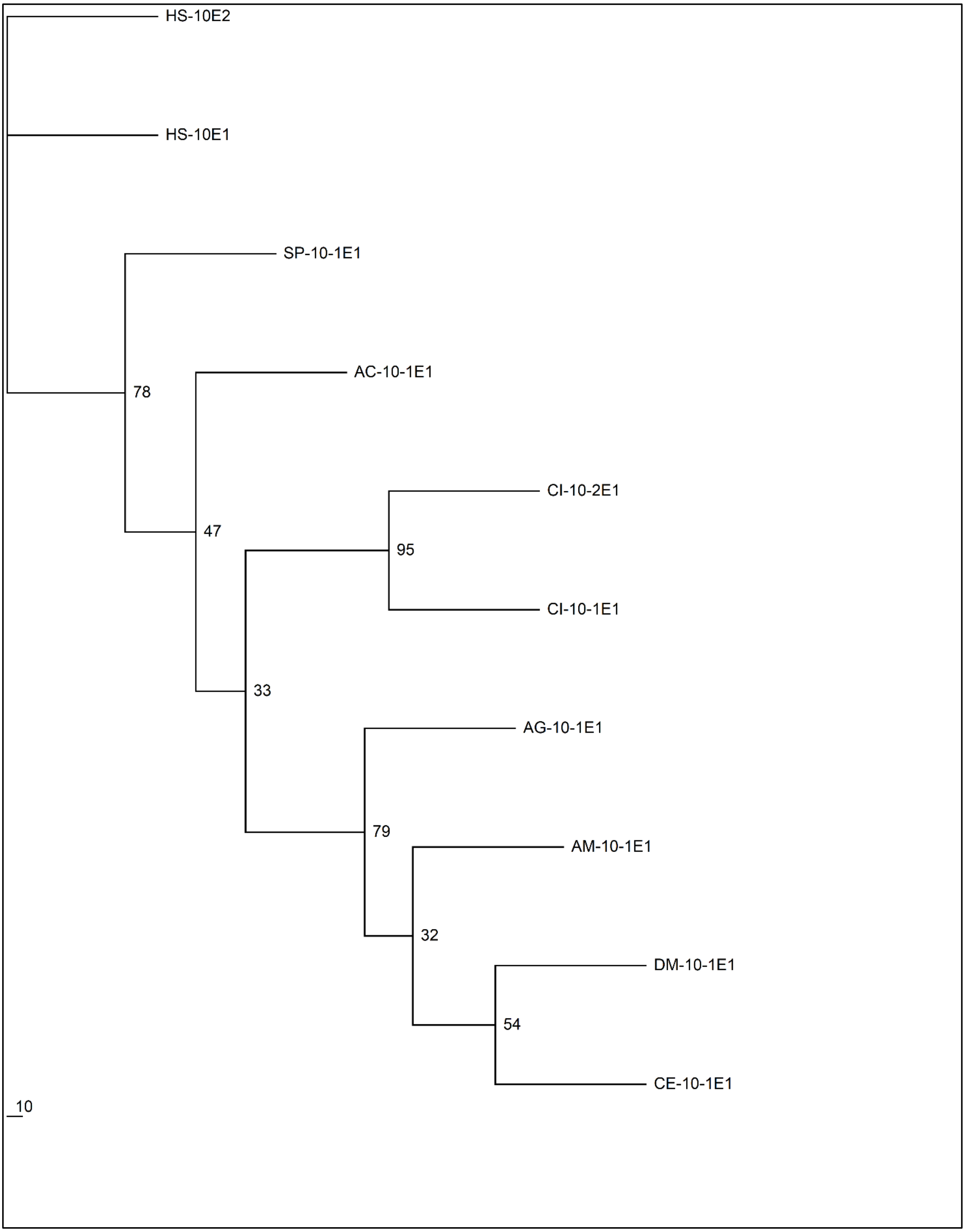

Figure S39. Phylogram of SDR10E gene structures. Splicing-site phase data are from Table S41a (Online Resources 4). *Homo sapiens* (HS), *Ciona intestinalis* (CI), *Strongylocentrotus purpuratus* (SP), *Drosophila melanogaster* (DM), *Anopheles gambiae* (AG*), Apis mellifera* (AM), *Caenorhabditis elegans* (CE)*, Aplysia californica* (AC).

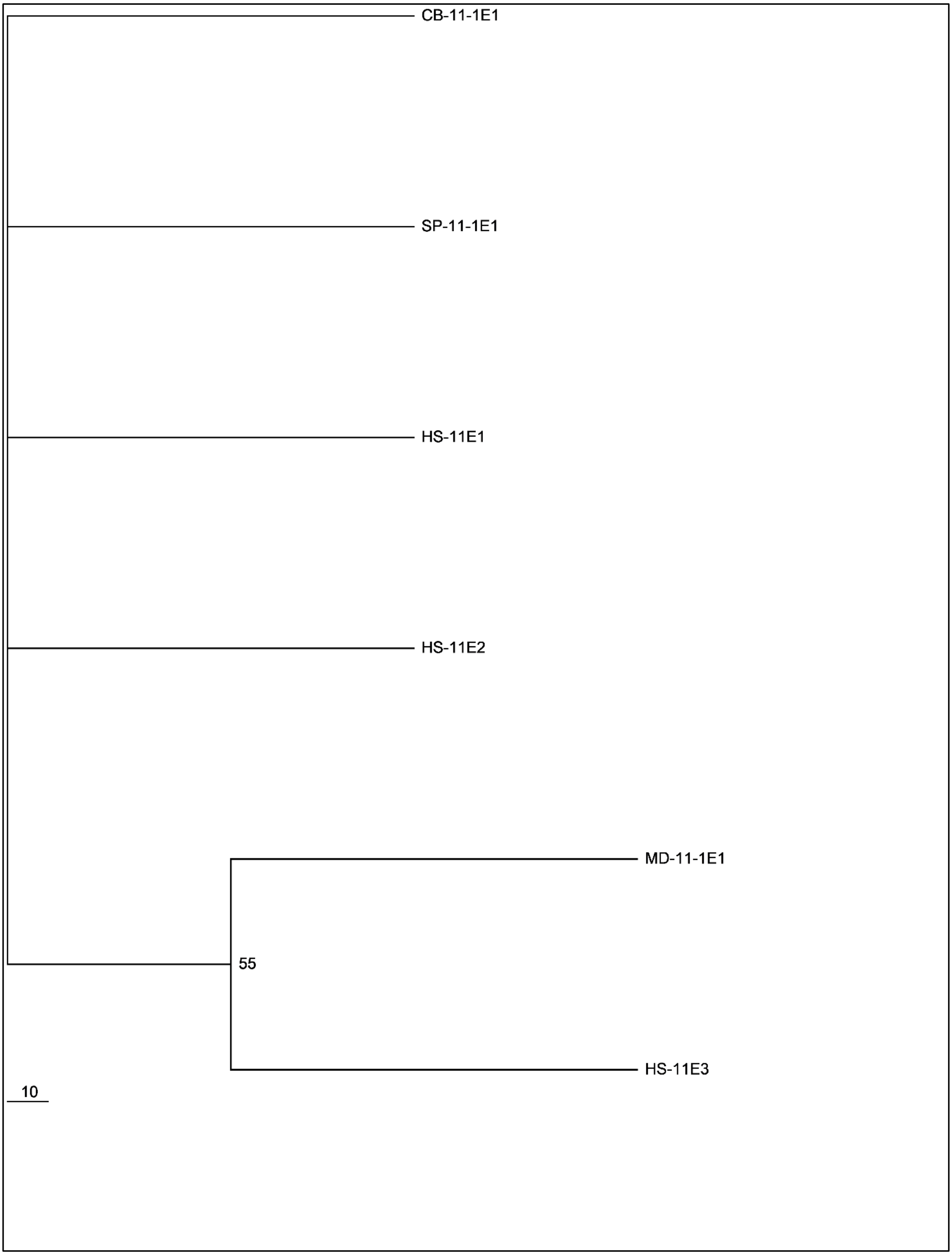

Figure S40. Phylogram of SDR11E gene structures. Splicing-site phase data are from Table S42a (Online Resources 4). *Homo sapiens* (HS), *Strongylocentrotus purpuratus* (SP), *Musca domestica* (MD), *Caenorhabditis briggsae* (CB).

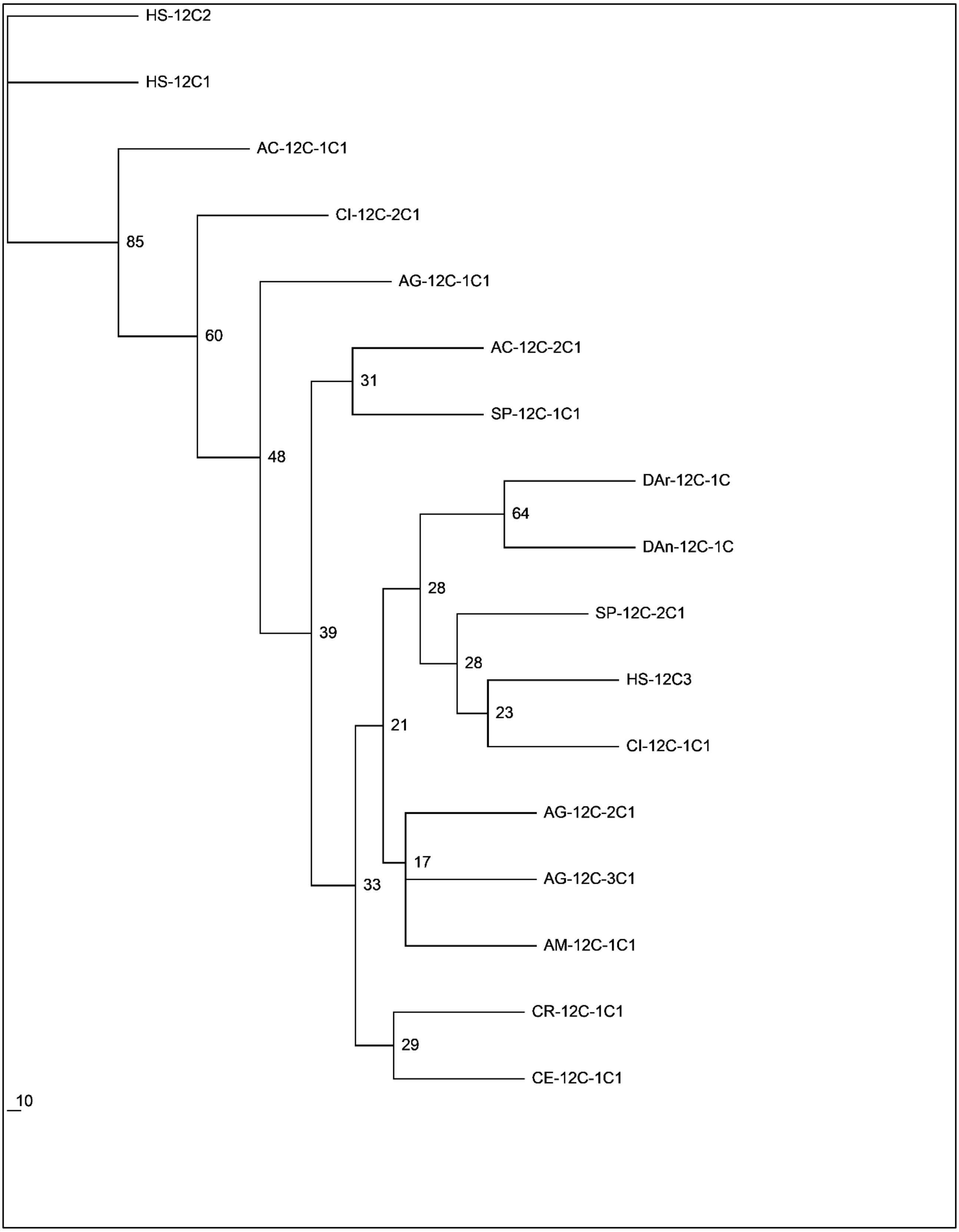

Figure S41. Phylogram of SDR12C gene structures. Splicing-site phase data are from Table S43a (Online Resources 4). *Homo sapiens* (HS), *Ciona intestinalis* (CI), *Strongylocentrotus purpuratus* (SP), *Drosophila arizonae* (DAr), *Drosophila ananassae* (Dan), *Anopheles gambiae* (AG), *Apis mellifera* (AM), *Caenorhabditis elegans* (CE), *Caenorhabditis remanei* (CR), *Aplysia californica* (AC).

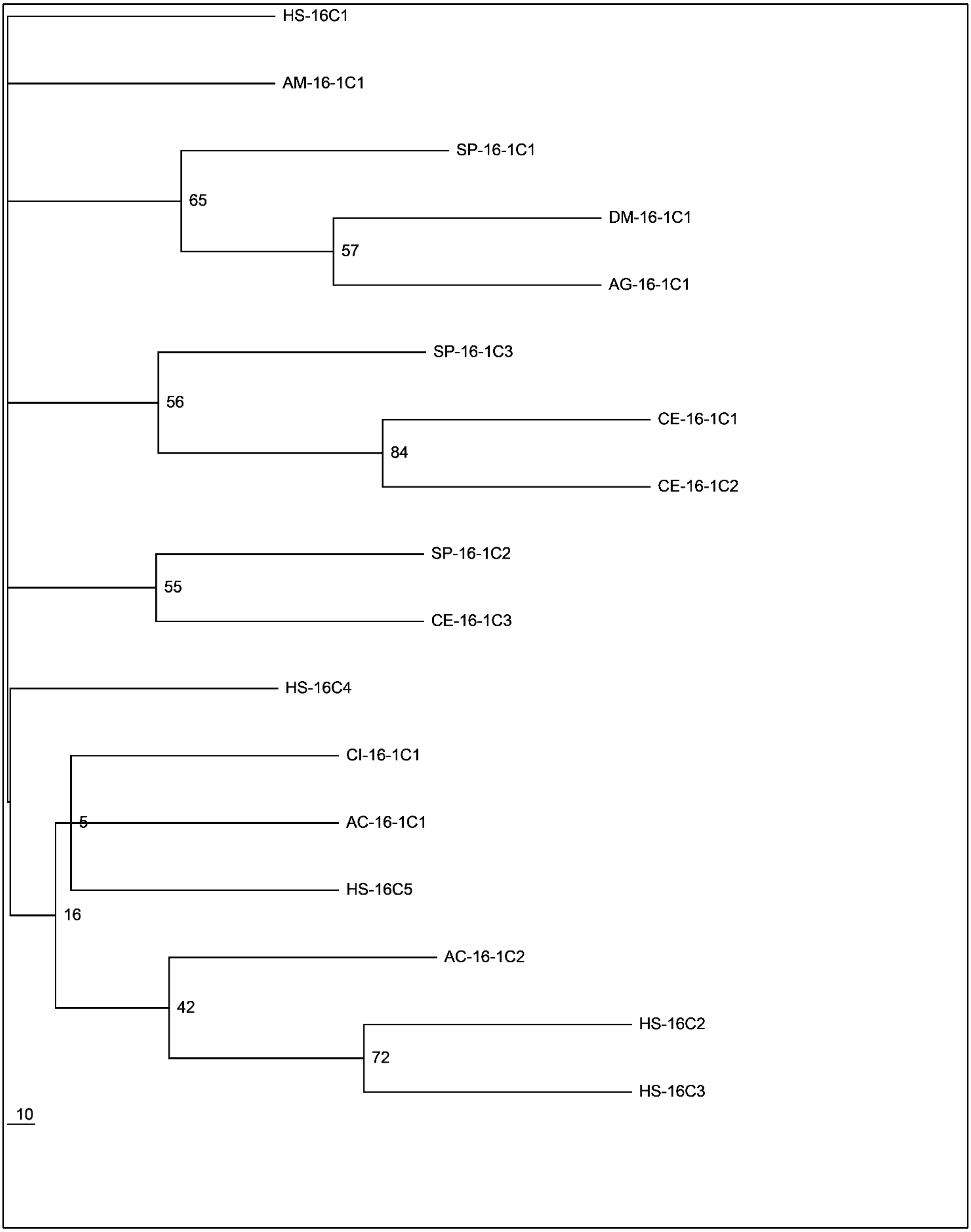

Figure S42. Phylogram of SDR16C gene structures. Splicing-site phase data are from Table S44a (Online Resources 4). *Homo sapiens* (HS), *Ciona intestinalis* (CI), *Strongylocentrotus purpuratus* (SP), *Drosophila melanogaster* (DM), *Anopheles gambiae* (AG*), Apis mellifera* (AM), *Caenorhabditis elegans* (CE)*, Aplysia californica* (AC).

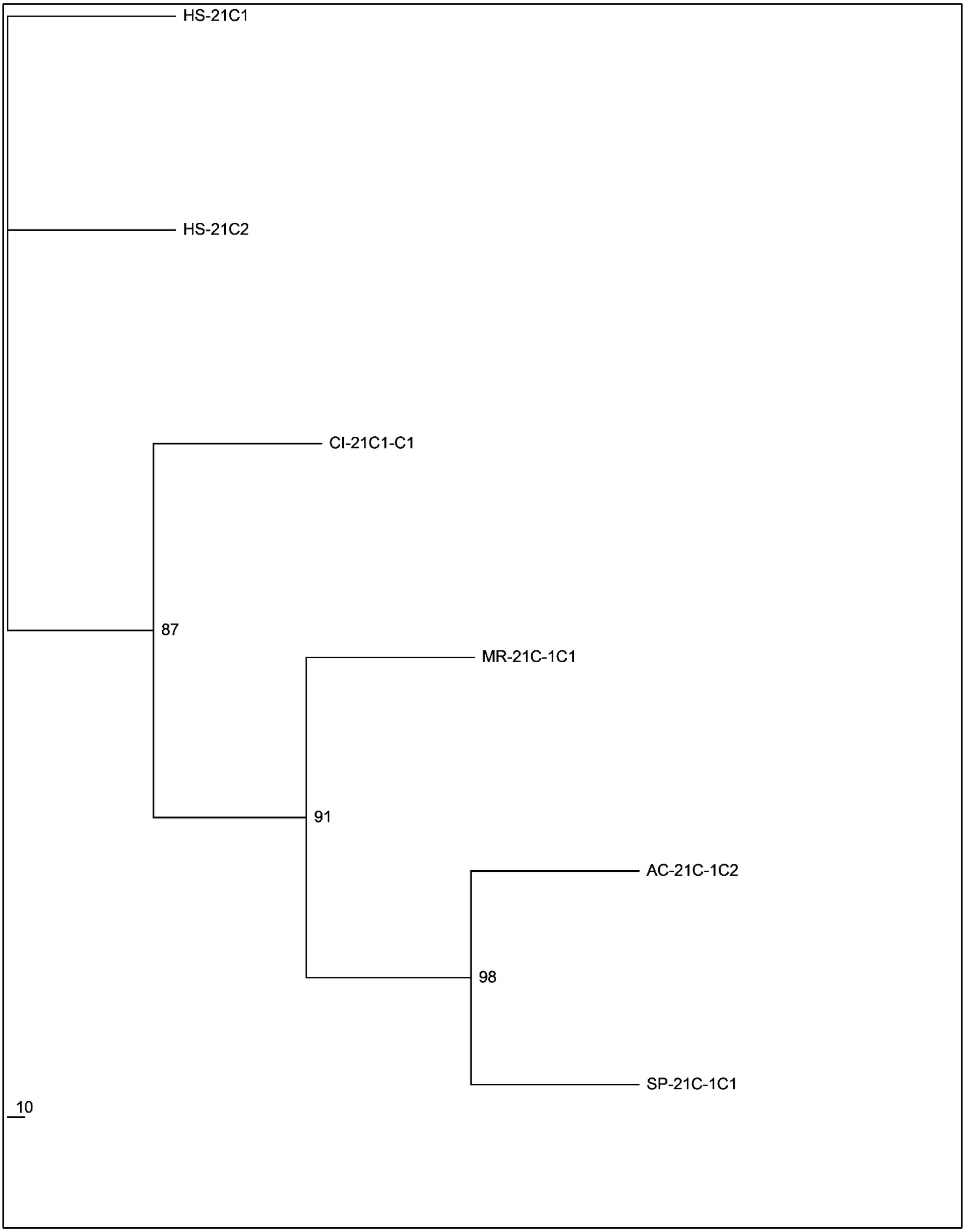

Figure S43. Phylogram of SDR21C gene structures. Splicing-site phase data are from Table S45a (Online Resources 4). *Homo sapiens* (HS), *Ciona intestinalis* (CI), *Strongylocentrotus purpuratus* (SP), *Megachile rotundata* (MR), *Aplysia californica* (AC).

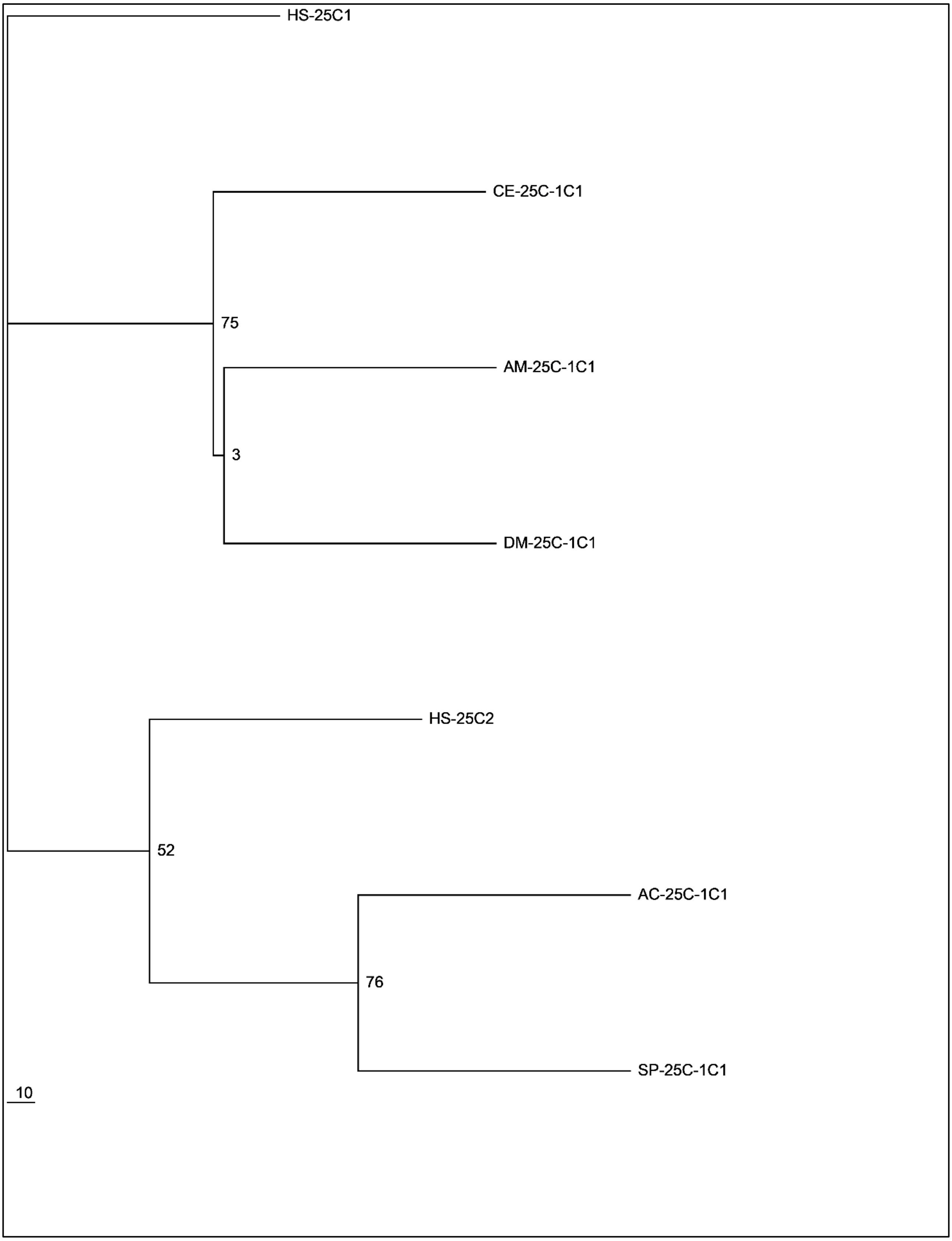

Figure S44. Phylogram of SDR25C gene structures. Splicing-site phase data are from Table S46a (Online Resources 4). *Homo sapiens* (HS), *Strongylocentrotus purpuratus* (SP), *Drosophila melanogaster* (DM), *Apis mellifera* (AM), *Caenorhabditis elegans* (CE), *Aplysia californica* (AC).

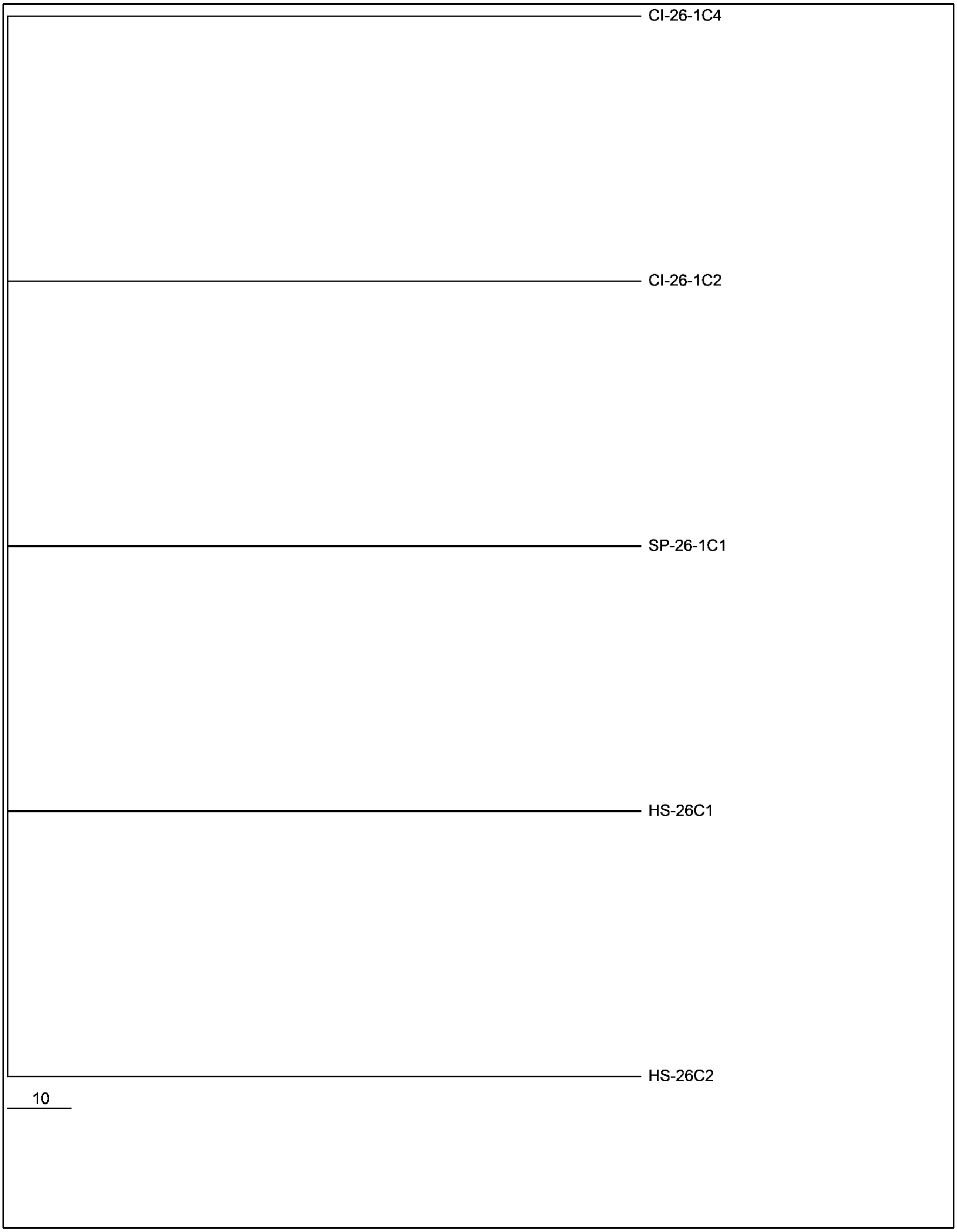

Figure S45. Phylogram of SDR26C gene structures. Splicing-site phase data are from Table S47a (Online Resources 4). *Homo sapiens* (HS), *Ciona intestinalis* (CI), *Strongylocentrotus purpuratus* (SP).

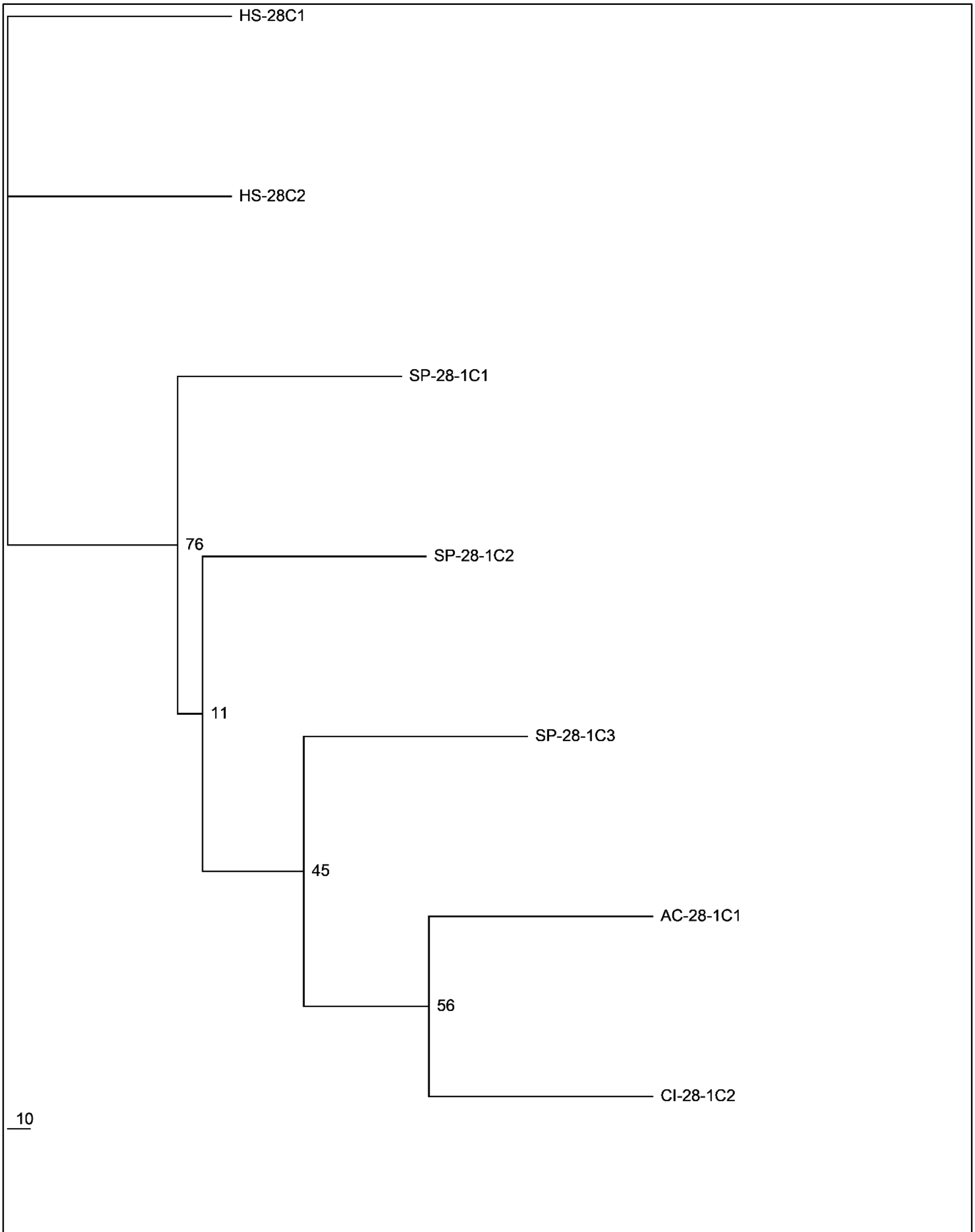

Figure S46. Phylogram of SDR28C gene structures. Splicing-site phase data are from Table S48a (Online Resources 4). *Homo sapiens* (HS), *Ciona intestinalis* (CI), *Strongylocentrotus purpuratus* (SP), *Aplysia californica* (AC).

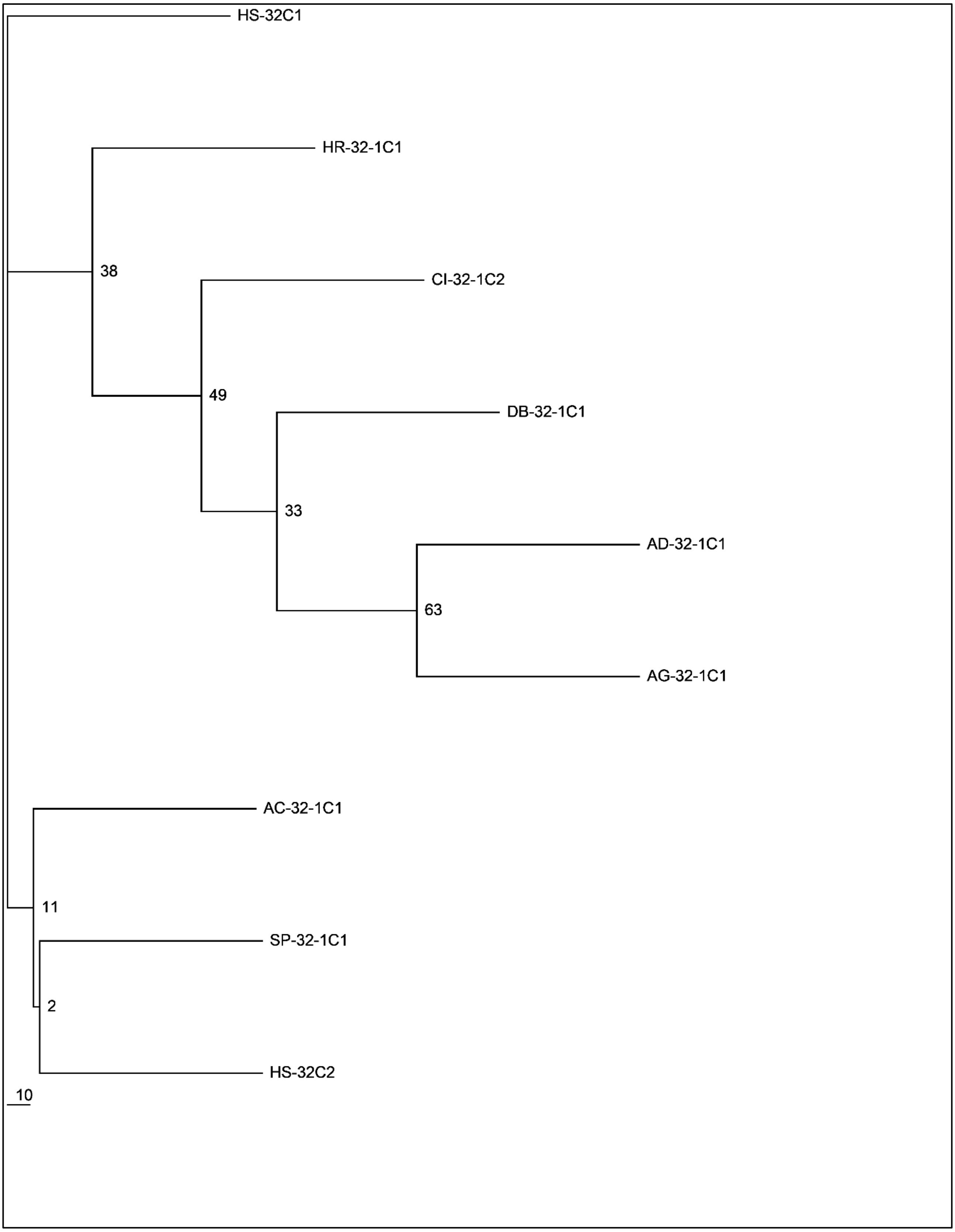

Figure S47. Phylogram of SDR32C gene structures. Splicing-site phase data are from Table S49a (Online Resources 4). *Homo sapiens* (HS), *Ciona intestinalis* (CI), *Strongylocentrotus purpuratus* (SP), *Drosophila busckii* (DB), *Anopheles gambiae* (AG), *Apis dorsata* (AD), *Helobdella robusta* (HR), *Aplysia californica* (AC).

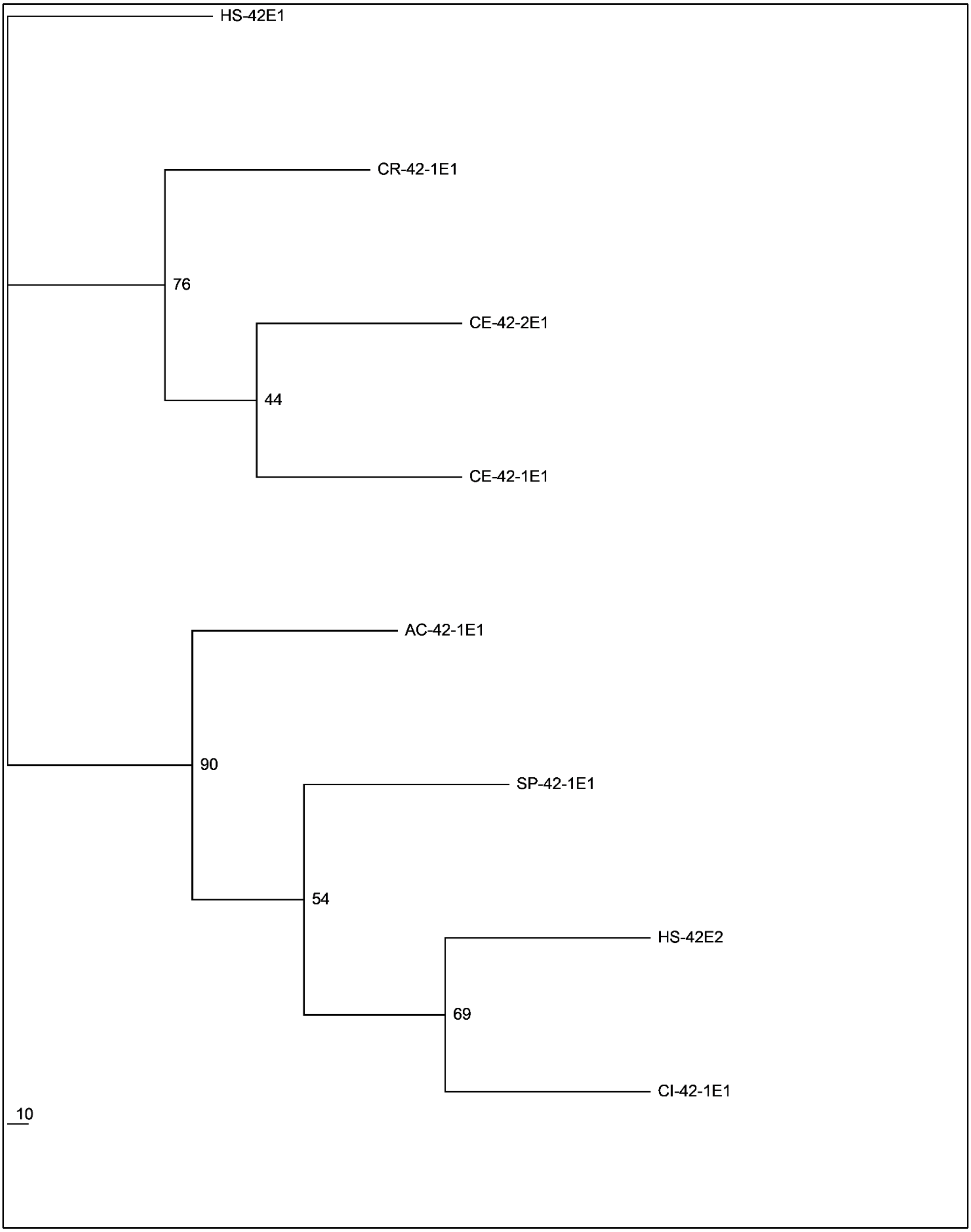

Figure S48. Phylogram of SDR42E gene structures. Splicing-site phase data are from Table S50a (Online Resources 4). *Homo sapiens* (HS), *Ciona intestinalis* (CI), *Strongylocentrotus purpuratus* (SP), *Caenorhabditis elegans* (CE), *Caenorhabditis remanei* (CR), *Aplysia californica* (AC).
