# Supplementary material for: Gene Structure Evolution of the Short-Chain Dehydrogenase/Reductase (SDR) Family
Source: Genes (Basel). 2022 Dec 30;14(1):110. doi: 10.3390/genes14010110 (PMC9859523; doi:10.3390/genes14010110)
Supplement: Supplementary file 1 [file genes-14-00110-s001.zip › Online Resources 6/Online Resources 6.pdf]

PRIMATE SDR42E VARIANTS

```
                                      x  x  x
SDR42E1 H. sapiens      MDPKRSQKESVLITGGSGYFGFrlGCALNQNGVHVILFDISSPAQTIPEGIKFIQGDIRH  1p.1
SDR42E1 P. troglodytes  MDPKRSQKETVLITGGSGYFGFrlGCALNQKGVHVILFDISSPAQTIPEGIKFIQGDIRH  1p.1
SDR42E1 P. paniscus     MDPKRSQKETVLITGGSGYFGFrlGCALNQKGVHVILFDISSPAQTIPEGIKFIQGDIRH  1p.1
SDR42E1 N. leucogenys   MDPQRPQKETVLITGGSGYFGFrlGCALNQKGVHVILFDISSPAQTIPEGIKFIRGDIRH  1p.1
SDR42E1 M. mulatta      MDPKRSQKETVLITGGGGYFGFrlGCALNQKGVHVILFDISSPAETIPEGIKFIQGDICH  1p.1
SDR42E1 P. anubis       MDPKRSQQETVLITGGGGYFGFrlGCALNQKGVHVILFDISSPAETIPEGIKFIQGDIRH  1p.1
SDR42E1 C. jacchus      MDPKRSPKETVLITGGGGYFGFrlGCALNQKGVHVILFDINSPAQTVPEGIKFIQGDIRH  1p.1
SDR42E1 M. murinus      MDAEKSPAETVLITGGGGYFGF-lGCALNQQGVHVILFDINSPAETIPEGIKFARGDIRH  1p.1
                        **      * ****** ***** ******* ********* *** * ******  *** *

SDR42E1 H. sapiens      LSDVEKAFQDADVTCVFHIASYGMSGREQLNRNLIKEVNVRGTDNILQVCQRRRVPRLVY
SDR42E1 P. troglodytes  LSDVEKAFQDADVTCVFHIASYGMSGREQLNRNLIEEVNVRGTDNILQVCQRRSVPRLVY
SDR42E1 P. paniscus     LSDVEKAFQDADVTCVFHIASYGMSGREQLNRNLIEEVNVRGTDNILQVCQRRSVPGLVY
SDR42E1 N. leucogenys   LSDVDKAFQDADVTCVFHIASYGMSGREQLNRNLIEEVNIRGTDNILQACQRRRVPRLVY
SDR42E1 M. mulatta      LSDIEKAFQDADITCVFHIASYGMSGREQLNRNLIEEVNIGGTDNILQACQRRRVPRLVY
SDR42E1 P. anubis       LSDIEKAFQDADITCVFHIASYGMSGREQLNRNLIEEVNIGGTDNILQACQRRRVPRLVY
SDR42E1 C. jacchus      VSDVEKAFQDADVTCVFHIASYGMSGWEQLSRNPIEEVNIGGTDNILQACQRRMVPRLVY
SDR42E1 M. murinus      LSDVENAFQDANVTCVFHIASYGMSGREQLNGNLIEEVNVGGTDNVLQACRRRGVPRLVY
                         **   *****  ************* ***  * * ***  **** ** * ** ** ***

                                                       +   +
SDR42E1 H. sapiens      TSTFNVIFGGQVIRNGDESLPYLPLHLHPDHYSRTKSIAEQKVLEANATPLDRGDGVLRT
SDR42E1 P. troglodytes  TSTFNVIFGGQVIRNGDESLPYLPLHLHPDHYSRTKSIAEQKVLEANATPLDRGDGVLRT
SDR42E1 P. paniscus     TSTFNVIFGGQVIRNGDESLPYLPLHLHPDHYSRTKSIAEQKVLEANATPLDRGDGVLRT
SDR42E1 N. leucogenys   TSTFNVIFGGQVIRNGDESLPYLPLHLHPDHYSRTKSIAEQKVLEANGTPLDRGDGVLRT
SDR42E1 M. mulatta      TSTFNVIFGGQVIRNGDESLPYLPLHLHPDHYSRTKSIAEKKVLEANGTPLDRGDGVLRT
SDR42E1 P. anubis       TSTFNVIFGGQVIRNGDESLPYLPLHLHPDHYSRTKSIAEKKVLEANGTPLDRGNGVLRT
SDR42E1 C. jacchus      TSTFNVIFGGQVIRNGDESLPYLPLHLHPDHYSRTKSIAEKKVLEANGTPLNGGGDVLRT
SDR42E1 M. murinus      TSTFNVIFGGQVIRNGDESLPYLPLHLHPDHYSRTKSIAERKVLEANGTLLETGDGVLRT
                        **************************************** ****** * *  *  ****

SDR42E1 H. sapiens      CALRPAGIYGPGEQRHLPRIVSYIEKGLFKFVYGDPRSLVEFVHVDNLVQAHILASEALR
SDR42E1 P. troglodytes  CALRPAGIYGPGEQRHLPRIVSYIEKGLFKFVYGDPRSLVEFVHVDNLVQAHILASEALR
SDR42E1 P. paniscus     CALRPAGIYGPGEQRHLPRIVSYIEKGLFKFVYGDPRSLVEFVHVDNLVQAHILASEALR
SDR42E1 N. leucogenys   CALRPAGIYGPGEQRHLPRIVSYIEKGLFKFVYGDPRSLVEFVHVDNLVQAHILASEALR
SDR42E1 M. mulatta      CALRPAGIYGPGEQRHLPRIVSYIEKGLFKFVYGDPRSLVEFVHVDNLVQAHILASEALR
SDR42E1 P. anubis       CALRPAGIYGPGEQRHLPRIVSYIEKGLFKFVYGDPRSLVEFVHVDNLVQAHILASEALR
SDR42E1 C. jacchus      CALRPAGIYGPGEQRHLPRIVSYIEKGLFKFVYGDPGSLVEFVHVDNLVQAHILALEALR
SDR42E1 M. murinus      CALRPAGIYGPGERRHLPRIVSYIERGLFKFVYGDPGSLVEFVHVDNLVQAHILASEALK
                        ************* *********** ********** ****************** ***

SDR42E1 H. sapiens      ADKGHIASGQPYFISDGRPVNNFEFFRPLVEGLGYTFPSTRLPLTLVYCFAFLTEMVHFI
SDR42E1 P. troglodytes  ADKGHIASGQPYFISDGRPVNNFEFFRPLVEGLGYTFPSTRLPLTLVYCFAFLTEMVHFI
SDR42E1 P. paniscus     ADKGHIASGQPYFISDGRPVNNFEFFRPLVEGLGYTFPSTRLPLTLVYCFAFLTEMVHFI
SDR42E1 N. leucogenys   ADKGHIASGQPYFISDGRPVNNFEFFRPLVEGLGYTFPSTRLPLTLVYCFAFLTEMVHFI
SDR42E1 M. mulatta      ADKGHIASGQPYFISDGRPVNNFEFFRPLVEGLGYTFPSTRLPLTLVYCFAFLTEMVHFI
SDR42E1 P. anubis       ADKGHIASGQPYFISDGRPVNNFEFFRPLVEGLGYTFPSTRLPLTLVYCFAFLTEMVHFI
SDR42E1 C. jacchus      ADKGHIASGQSYFISDGRPVNNFEFFRPLVEGLGYTFPSTRLPLTLVYCFAFLTEMVHFI
```

```
SDR42E1 M. murinus      ADKGYIASGQPYFISDGRPVNNFEFFRPLVEGLGYTFPSTRLPLYLIYCFAFLTEMAHFL
                        **** ***** ********************************* * ********* **

SDR42E1 H. sapiens      LGRLYNFQPFLTRTEVYKTGVTHYFSLEKAKKELGYKAQPFDLQEAVEWFKAHGHGRSSG
SDR42E1 P. troglodytes  LGRLYNFQPFLTRTEVYKTGVTHYFSLEKAKKELGYKAQPFDLQEAVEWFKAHGHGRSSG
SDR42E1 P. paniscus     LGRLYNFQPFLTRTEVYKTGVTHYFSLEKAKKELGYKAQPFDLQEAVEWFKAHGHGRSSG
SDR42E1 N. leucogenys   LGRLYNFQPFLTRTEVYKTGVTHYFSLEKAKKELGYKAQPFDLQEAVEWFKAHGHGRSSG
SDR42E1 M. mulatta      LGRLYNFQPFLTRTEVYKTGVTHYFSLEKAKKELGYKAQPFDLQEAVEWFKAHGHGRSSG
SDR42E1 P. anubis       LGRLYNFQPFLTRTEVYKTGVTHYFSLEKAKKELGYKAQPFDLQEAVEWFKAHGHGRSSG
SDR42E1 C. jacchus      LGRLYNFQPFLTRTEVYKTGVTHYFSLEKAKKELGYKAQPFDLQEVVEWFKAHGHGRSSG
SDR42E1 M. murinus      LGRLYNFQPFLTRTEVYKTGVTHYFSLEKAKKELGYEAQPFDLQEVVEWFKAHGHGRSPG
                        ************************************ ******** ************ *

SDR42E1 H. sapiens      SRDSECFVWDGLLVFLLIIAVLMWLPSSVILSL
SDR42E1 P. troglodytes  SRDSECFVWDGLLVFLLIIAVLMWLPSSVILSL
SDR42E1 P. paniscus     SRDSECFVWDGLLVFLLIIAVLMWLPSSVILSL
SDR42E1 N. leucogenys   SSDLECFVWDGLLVFLLIIAVLMWLPPSVILSL
SDR42E1 M. mulatta      SRDSECFIWDGLLVFLLIIAVLIWLPSSVILSL
SDR42E1 P. anubis       SRDSECFIWDGLLVFLLIIAVLIWLPSSVILSL
SDR42E1 C. jacchus      SRDSDCFVWDGLLVFLLIIATLTWLLSVILLL-
SDR42E1 M. murinus      SHGSEYHVWRGLLVFFLVIAVLTWLLPSVILLL
                        *       * ***** * ** * **     *
```

Figure S49. Alignment of the primate SDR42E protein variants. For further details see Online Resources 2 Fig. S3.

* symbol marks the position of identical amino acid residues of the aligned SDR protein sequences.

```
SDR42E2 H. sapiens      MKSNPPRSSLEACKAAGQGEKSCPVCQACGEVSGPRSGSGSESRPAPKPGAIPGPGLGPK
SDR42E2 P. troglodytes  MKSNPPRSSLEACKAAGQGEKSCPVCQACGEVSGPRSGSGSESRPAPKPGAIPGPGLGPK
SDR42E2 P. paniscus     ------------------------------------------------------------
SDR42E2 N. leucogenys   ------------------------------------------------------------
SDR42E2 M. mulatta      MKSNPPGSSPEACKAAGQGEKSCPVCQACGEVSGPRSGLGSESGPAPKPGAIPGPGLGPK
SDR42E2 P. anubis       MKSNPPGSSPEACKFAAQGEKSCPVCQACGEVSGPRSGLGSESGPAPKPGAIPGPGLGPK
SDR42E2 C. jacchus      MKPNPPGSSPEACKAAGQGEKSCPVCQACGEVSGPRSGLGSESGPVPKPGAIPGPGLGPK
SDR42E2 M. murinus      MKPNPTG------KAADQGEKSCPVCQACGGVSGPKSGLGPGPGP----NSIPGQGAGPG

SDR42E2 H. sapiens      AIPGPQAGSGTVPRPGAISGTGPGLGPGPGAGSVPGPGAGSVPGLGARSVPGPGAGSVPG
SDR42E2 P. troglodytes  AIPGPQAGSDTVPRPGAISGTGPSLGPGPGAGSVPGPGTGSVP--------GPGAGSVPG
SDR42E2 P. paniscus     ------------------------------------------------------------
SDR42E2 N. leucogenys   ------------------------------------------------------------
SDR42E2 M. mulatta      AIPGPQAGSGVAPRPGAISGTGPGLGPGPGARSVPGPGAESIPGLGAGSVPGPGTGSIPG
SDR42E2 P. anubis       AIPGPQAGSGVALRPGAISGTGLGLGPGPGAGSVPGPGVGSIPGLGAGSVPGPGTGSIPG
SDR42E2 C. jacchus      AIPEPQVGSGAGLRPDTVSGTGPGLGPGPGSGSVPGPGARSVPAPGARLAPAPGARSAPG
SDR42E2 M. murinus      SVPGPGAVPGRGA----------------GSGAVPGRGAG--------------------

SDR42E2 H. sapiens      PGAGSVPGPGAGSVPGPGAGSGPGLGGGLGPGVGAGPGAGSVPGPGAGSVPGPGAGSVPG
SDR42E2 P. troglodytes  LGAGSVPGPGAGSVPGPGAGSGPGLGAGLGPGLGAGPGAGSVPGPGAGSVPG--------
SDR42E2 P. paniscus     ------------------------------------------------------------
SDR42E2 N. leucogenys   ------------------------------------------------------------
SDR42E2 M. mulatta      LGVGSIPGPGAGSILGPGAGSGPGLGAGSAPGPGSGSVPGLGAGLGHGLGAGPEAGSVPG
SDR42E2 P. anubis       LGVGSIPGPGAGSVLGPGAGSGPGLGAGSVPGPGAGSVSGLGAGLGHGLGAGPEAGSVPG
SDR42E2 C. jacchus      LGAESVPEPGAGLVPGPGSGSAPGLGAGSVPVIGAGSVPG------------PGAGSVPG
SDR42E2 M. murinus      --PGAVPGRGAG----------------------PGAVPGRGAGPGAVPGPESGPGAVLG

SDR42E2 H. sapiens      PGAGSVPGAGAGSTPEPELGPGLRQGSGTGPRPSESTTTPTPAPQQKTQAKPTKAARQKV
SDR42E2 P. troglodytes  --------PGAGSTPEPELGPGLRQGSGTGPKPSESTTTPTPAPQQKTQAKPTKAARQKV
SDR42E2 P. paniscus     ------------------------MKSNPPRSSLEACKAAGqaPQQKTQAKPTKAARQKV  1p.2
SDR42E2 N. leucogenys   -----------------------MKSNPPGS-SPDACKAVGqaPQQKTQAKPTKAARQKV  1p.2
SDR42E2 M. mulatta      PGAGSVPGPGAGSTPEPELGPGPRQGSGTGPKPKESTTTPTPAPQQKTQAKPTKVARQKV
SDR42E2 P. anubis       PGAGSVPGPGAGSTPEPELGPGLRQGSGTGPKPKESTTTPTPAPQQKTQAKPTKVARQKV
SDR42E2 C. jacchus      LGSGSAPGPGAGSTPEPELGSGLTQGSVTGPKLRESTATPTPAPQQKTQAKPTKAARQKV
SDR42E2 M. murinus      PGPGLGPGPGAGSLPRPEVGPGPRKGSGTGLKPSESMTTPAPAPQQKTQAKPEKAPRQKV
                                                                  ********** *  ****

SDR42E2 H. sapiens      LVTGGGGYLGFSLGSHLAKSGTSVILLDRRRPQWELSPETKFIqaDVRDEEALYRAFEGV  1p.0
SDR42E2 P. troglodytes  LVTGGGGYLGFSLGSHLAKSGTSVILLDCRRPQWELSPETKFIqaDVRDEEALYRAFEGV  1p.0
SDR42E2 P. paniscus     LVTGGGGYLGFSLGSHLAKSGTSVILLDRRRPQWELSPETKFIqaDVRDEEALYRAFEGV  2p.0
SDR42E2 N. leucogenys   LVTGGGGYLGFSLGSHLAKTGTSVILLDRRRPQWELSPETKFIqaDVRDEEALYRAFEGV  2p.0
SDR42E2 M. mulatta      LVTGGGGYLGFSLGSHLAKSGTPVILLDRRRPQWELSPETEFIqaDVRDEEALYRAFKGV  1p.0
SDR42E2 P. anubis       LVTGGGGYLGFSLGSHLAKSGTPVILLDRRRPQWELCPETEFIqaDVRDEEALYRAFKGV  1p.0
SDR42E2 C. jacchus      LVTGGGGYLGFSLGSHLAKSGTSVILLDRRRPQWELSPETEFIqaDVRDEAALYRAFEGV  1p.0
SDR42E2 M. murinus      LVTGGGGYLGFTLGSSLARSGTSVILLDLHRPQWTLPSGTEFIqaDVRDEETLYRAFEGV  1p.0
                        *********** *** **  ** *****  **** *   * *********  ***** **
```

```
SDR42E2 H. sapiens       DCVFHVASYGMSGAEklQK-EQIESINVGGTKLVIdvCVRRRVPRLIYTSTVNVAFGGKP  2p.0 3p.2
SDR42E2 P. troglodytes   DCVFHVASYGISGAEklQK-EQIESINVGGTKLVIdvCVRRRVPRLIYTSTVNVAFGGKP  2p.0 3p.2
SDR42E2 P. paniscus      DCVFHVASYGMSGAEklQK-EQIESINVGGTKLVIdvCVRRRVPRLIYTSTVNVAFGGKP  3p.0 4p.2
SDR42E2 N. leucogenys    DCVFHVASYGMSGAEklQK-EQIESINVGGTKLVIdvCVRRRVPRLIYTSTVNVAFGGKP  3p.0 4p.2
SDR42E2 M. mulatta       DCVFHMASYGMSGAEklQK-EQIESINVGGTKLVIdvCVRQRVPRLIYTSTVNVAFGGKP  2p.0 3p.2
SDR42E2 P. anubis        DCVFHMASYGLSGAEklQEMRQMESINVGGTKLVIdvCVRQRVPRLIYTSTVNVAFGGKP  2p.0 3p.2
SDR42E2 C. jacchus       DCVFHVASYGMSGAEklQK-EQIESINVGGTKLVIdvCVRRRVPRLIYTSTVNVAFGGKP  2p.0 3p.2
SDR42E2 M. murinus       DCVFHMASYGMSGEEklQK-EQIESINVGGTKLVIdvCVRRRVPRLIYTSTVNVAFGGKP  2p.0 3p.2
                         ***** **** ** ****   * ***************** *******************

SDR42E2 H. sapiens       IEQGDEDSVPYFPLDehVDHYSRTKAIADQLTLMANGMPLpgGGTLRTCVLRPPGIYGPE  4p.0 5p.2
SDR42E2 P. troglodytes   IEQGDEDSVPYFPLDehVDHYSRTKAIADQLTLMANGTPLpgGGTLRTCVLRPPGIYGPE  4p.0 5p.2
SDR42E2 P. paniscus      IEQGDEDSVPYFPLDehVDHYSRTKAIADQLTLMANGTPLpgGGTLRTCVLRPPGIYGPE  5p.0 6p.2
SDR42E2 N. leucogenys    IEQGDEDSVPYFPLDehIDHYSRTKAIADQLTLMANGTPLpgGGTLRTCVLRPPGIYGPE  5p.0 6p.2
SDR42E2 M. mulatta       IEQGDEDSVPYFPLDehIDHYSRTKAIADQLTLMANGTPLpgGGALRTCVLRPPGIYGPE  4p.0 5p.2
SDR42E2 P. anubis        IEQGDEDSVPYFPLDkhIDHYSRTKAIADQLTLMANGTPLpgGGALRTCVLRPPGIYGPE  4p.0 5p.2
SDR42E2 C. jacchus       IQQGDEDSVPYFPLDehIDHYSRTKAIADQLTLMANGTPLpgGGILRTCVLRPPGIYGPE  4p.0 5p.2
SDR42E2 M. murinus       IEQGDEDSVPYFPLDkhVDHYSRTKAIADQLTLMANGTPLpgGGTLRTCVLRPPGIYGPE  4p.0 5p.2
                         * ************* * ******************* ****** ***************

SDR42E2 H. sapiens       EQRHLPRVagHIKKRLFMFRFGDHKARMNWVHVHNLVQAHVLAAEALTTAKGYVasGQAY  6p.0 7p.0
SDR42E2 P. troglodytes   EQRHLPRVagHIKKRLFMFRFGDRKARMNWVHVHNLVQAHVLAAEALTTAKGYVasGQAY  6p.0 7p.0
SDR42E2 P. paniscus      EQRHLPRVagHIKKRLFMFRFGDRKARMNWVHVHNLVQAHVLAAEALTTAKGYVasGQAY  7p.0 8p.0
SDR42E2 N. leucogenys    EQRHLPRVagHIKKRLFMFRFGDRKARMNWVHVHNLVQAHMLASEALTAAKGYVasGQAY  7p.0 8p.0
SDR42E2 M. mulatta       EQRHLPRVagHIKKRLFMFRFGDRKARMNWVHVHNLVQAHVLAAEALTAAKGYVasGQAY  6p.0 7p.0
SDR42E2 P. anubis        EQRHLPRVagHIKKRLFMFRFGDRKARMNWVHVHNLVQAHVLAAEALTAAKGYVanGQAY  6p.0 7p.0
SDR42E2 C. jacchus       EQRHLPRVagHIKKRLFSFRFGDRKAQMNWVHVHNVVQAHVLAAKALTVGKGYVasGQAY  6p.0 7p.0
SDR42E2 M. murinus       EQRHLPRVvsHLKKMLFMVRFGDRRTRMNWVHVHNLVQAHMLAAEALTAAKGYVasGQAY  6p.0 7p.0
                         ********  * ** **  ****    ********* **** **  *** ***** ****

SDR42E2 H. sapiens       YINDGESVNLFEWMAPlfEKLGYSQPWIQVPTSWVYLtaAVMERLHLALRPICSLPPLLT  8p.0  9p.2
SDR42E2 P. troglodytes   YINDGESVNLFEWMAPlfEKLGYSQPWIQVPTSWVYLtaAVMERLHLALRPICSLPPLLT  8p.0  9p.2
SDR42E2 P. paniscus      YINDGESVNLFEWMAPlfEKLGYSQPWIQVPTSWVYLtaAVMERLHLALRPICSLPPLLT  9p.0 10p.2
SDR42E2 N. leucogenys    YINDGESVNLFEWMAPlfEKLGYSQPWIQVPTSWVYLtaAVMERLHLALRPICSLPPLLT  9p.0 10p.2
SDR42E2 M. mulatta       YINDGESVNLFEWMAPlfEKLGYSQPWIQVPTSWVYLtaAVMEHLHLALRPICCLPPLLT  8p.0  9p.2
SDR42E2 P. anubis        YINDGESVNLFEWMAPlfEKLGYSQPWIQVPTSWVYLtaAVMERLHLALRPICCLPPLLT  8p.0  9p.2
SDR42E2 C. jacchus       YINDGESVNLFEWMAPlfEKLGYSQPRIQVPTSWVYLtaAVMEHLHLALRPICNLPLLLT  8p.0  9p.2
SDR42E2 M. murinus       YINDGDSINLFEWIAPlfEKLGYSQPWIQVPTSWIYLtaSVMEHVHLALRPICSVPLLLT  8p.0  9p.2
                         ***** * ***** ************ ******* **** ***  ********  * ***

SDR42E2 H. sapiens       RSevRSVAVTHTFQIAKARAQLGYAPDKFRFADAVELYVQSTTRRPRGSTARTLLRLLLR  10p.0
SDR42E2 P. troglodytes   RSevRSVAVTHTFQIAKARAQLGYAPDKFRFADAVELYVQSTTRRPRGSTARTLLRLLLG  10p.0
SDR42E2 P. paniscus      RSevRSVAVTHTFQIAKARAQLGYAPDKFRFADAVELYVQSTTRRPRGSTARTLLRLLLG  11p.0
SDR42E2 N. leucogenys    RSevRSVAVTHTFQIAKARAQLGYAPDKFRFADAVELYVQSTTWRPRGSTARTLLRLLLG  11p.0
SDR42E2 M. mulatta       RSevRSVAVTHTFQIAKARAQLGYAPDKFKFADAVELYVQSTTRRPRGSTARTLLRLLLG  10p.0
SDR42E2 P. anubis        RSevRSVAVTHTFQIAKARAQLGYAPDKFRFADAVELYVQSTTRRPRGSTARTLLRLLLG  10p.0
SDR42E2 C. jacchus       RSevCSVAVTHTFQIAKARAQLGYAPDKFSLADVVERYVQSTSPRPRSSTAQTLLRLLLG  10p.0
SDR42E2 M. murinus       RSevCSVAVTHTFQIAKARAQLGYAPDKFSFSDAVERYMQSSAPRQRGSTTRALLRLLLG  10p.0
                         **** ************************   * ** * **   * * **   ******

SDR42E2 H. sapiens       LLLFLGLLALALHFLGLQPLHAAVERL
```

```
SDR42E2 P. troglodytes  LLLFLGLLALALHFLGLQPLHAAVERL
SDR42E2 P. paniscus     LLLFLGLLALALHFLGLQPLHAAVERL
SDR42E2 N. leucogenys   LLLFLGLLALALHFLGLQPLHAAVERL
SDR42E2 M. mulatta      LLLFLGLLALALHFLGLQPLHSSVERL
SDR42E2 P. anubis       LLLFLGLLALALHFLGLQPLQASVERL
SDR42E2 C. jacchus      LLLLLGLLALALHFLGLQPLQAAMKRL
SDR42E2 M. murinus      LL-LLGLLALALHYLCLQPLRAATERL
                        **  ********* * ****     **
```

Figure S50. Alignment of the primate SDR42E2 proteins variants. For further details see Online Resources 2 Fig. S3.

* symbol marks the position of identical amino acid residues of the aligned SDR protein sequences.

Species	Gene symbol	Gene ID	Chr	Enzyme symbol	Exons n.	Phases formula	% identity	aa n.	Structure consensus	Catalysis Consensus
Homo sapiens	SDR42E1	93517	16	SDR42E1	2	1	100.00	393	GGSGYFG	YSRTK
Pan troglodytes		468047	16				98.98			
Pan paniscus		100984124	16				98.73			
Nomascus leucogenys		100588195	2				96.69			
Macaca mulatta		714229	20				96.18		GGGGYFG	
Papio anubis		101004680	20				95.93			
Callithrix jacchus		100393984	20				91.58	392		
Microcebus murinus		105859480	20				87.50	393		
Homo sapiens	SDR42E2	100288072	16	SDR42E2	11	0020200020	100.00	626	GGGGYLG	YSRTK
Pan troglodytes		100615511	16				97.84	602		
Pan paniscus		100971052	16		12	20020200020	95.50	422		
Nomascus leucogenys		100592297	2				93.84			
Macaca mulatta		106994972	20		11	0020200020	91.21	626		
Papio anubis		101004328	20				90.42	627		
Callithrix jacchus		100895632	12				87.13	614		
Microcebus murinus		105885292	17				80.00	555		

Table S51a. Genetic and molecular data of primate SDR42E family variants. For further details see Online Resources Table S39a.

Enzyme	Species	% identity	Enzyme	Species	% identity
SDR42E1	*Homo sapiens*	100.00	SDR42E2	*Homo sapiens*	100.00
	Pan troglodytes	98.98		*Pan troglodytes*	97.84
	Pan paniscus	98.73		*Pan paniscus*	95.50
	Nomascus leucogenys	96.69		*Nomascus leucogenys*	93.84
	Macaca mulatta	96.18		*Macaca mulatta*	91.21
	Papio anubis	95.93		*Papio anubis*	90.42
	Callithrix jacchus	91.58		*Callithrix jacchus*	87.13
	Microcebus murinus	87.50		*Microcebus murinus*	80.00
SDR42E2	*Homo sapiens*	47.95	SDR42E1	*Homo sapiens*	47.95
	Pan troglodytes	48.21		*Pan troglodytes*	47.95
	Pan paniscus	48.46		*Pan paniscus*	47.69
	Nomascus leucogenys	48.97		*Nomascus leucogenys*	47.69
	Macaca mulatta	48.21		*Macaca mulatta*	47.69
	Papio anubis	47.44		*Papio anubis*	47.95
	Callithrix jacchus	47.18		*Callithrix jacchus*	48.07
	Microcebus murinus	48.84		*Microcebus murinus*	47.04

Table S51b. Relative percent identity of primate SDR42E family protein variants.
